# Copper-Catalyzed Cyclization and Alkene Transposition Cascade Enables a Modular Synthesis of Complex Spirocyclic Ethers

**DOI:** 10.1021/jacs.4c14418

**Published:** 2024-12-20

**Authors:** Wan-Xu Wei, Yangjin Kuang, Martin Tomanik

**Affiliations:** Department of Chemistry, New York University, New York, New York 10003, United States

## Abstract

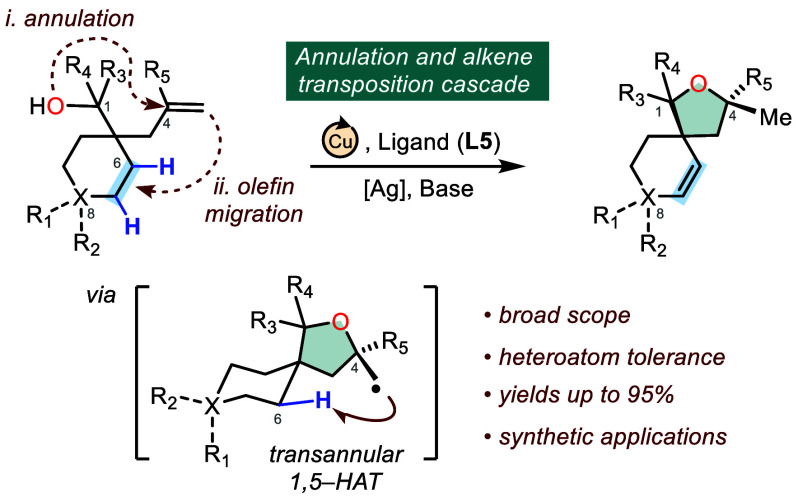

Complexity-generating reactions that access three-dimensional products from simple starting materials offer substantial value for drug discovery. While oxygen-containing heterocycles frequently feature unique, nonaromatic architectures such as spirocyclic rings, exploration of these chemical spaces is limited by conventional synthetic approaches. Herein, we report a copper-catalyzed annulation and alkene transposition cascade reaction that enables a modular preparation of complex, spirocyclic ethers from readily available alkenol substrates via a copper-catalyzed annulation and transannular 1,5-hydrogen atom transfer-mediated C–H functionalization. Our transformation displays a broad substrate scope, shows excellent heteroatom compatibility, and readily constructs spirocycles of varying ring sizes. The wider synthetic utility of this method is highlighted by numerous product diversifications and a short synthesis of the all-carbon framework of spirotenuipesine A. We anticipate that this transformation can significantly streamline access to a privileged class of three-dimensional oxygen-containing heterocycles and will find broad application in natural product synthesis.

## Introduction

**Figure 1:**
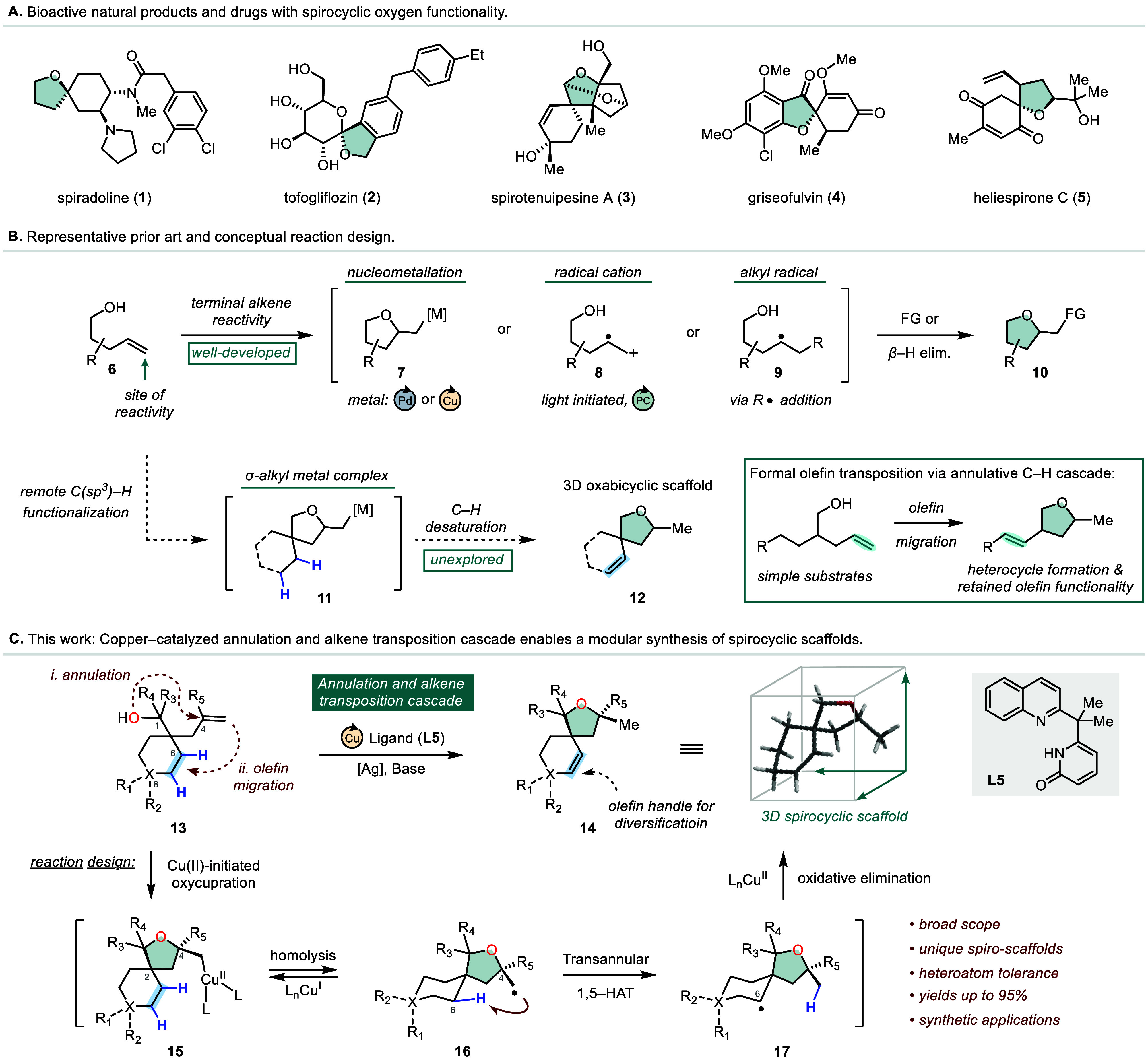
Copper-catalyzed annulation and alkene transposition cascade for the synthesis of 3D desaturated oxabicyclic scaffolds.

Oxygen-containing heterocycles are the second-most common structural motif in FDA-approved therapeutics and are primarily derived from commercially available pyranoses and furanoses.^1^ Beyond carbohydrates, they exist predominantly as nonaromatic compounds, often with complex architectures such as spiro centers in several notable drugs and natural products (**1**–**5**, Figure 1A).^2^ This prevalence across bioactive molecules suggests that exploring saturated versions of heterocyclic oxygen scaffolds could offer new and useful compounds.^3^ Inspired by the challenges inherent to preparing such complex molecules and the demand for increasing access to diverse three-dimensional motifs, we sought to develop a modular annulation reaction capable of streamlining synthetic access to a privileged class of oxygen-containing heterocycles. Existing strategies to prepare oxo-containing spirocyclic compounds often employ bifunctional intermediates; however, harsh reaction conditions are typically needed to displace leaving groups.^4^ Other notable cyclization strategies have utilized γ-hydroxyalkenes to provide access to such heterocycles (Figure 1B). In these approaches, key reactive intermediates can be accessed via transition metal-mediated nucleometallation (**7**),^5^ photocatalytic olefin oxidation (**8**),^6^ or radical-mediated olefin addition (**9**).^7^ While highly enabling transformations, these methods suffer from several notable pitfalls such as regioselectivity issues, limited coupling partner scope, and competitive oxidative pathways. Moreover, the site of reactivity in these transformations is often restricted to the terminal position of the original olefinic residue, thereby limiting the further derivatization of the resulting carbocyclic scaffolds.

We recognized that a more compelling strategy could leverage the in situ generated σ-alkyl complex **11**, formed via a regioselective nucleometallation event, toward an unprecedented remote C–H dehydrogenation pathway (**11** → **12**, Figure 1B). Such a transformation, which would constitute a formal annulation and olefin transposition cascade, would not only provide efficient synthetic access to a family of complex three-dimensional spirocyclic ethers but also retain the synthetically useful olefin functionality present in the starting material. Importantly, this olefinic residue would be reintroduced at an arguably more desirable location within the carbocycle, adding considerable value for medicinal chemistry applications due to the capability to further explore chemical space through well-established alkene transformations. Unlike conventional transformations that swap one functional group for another, remote functional group transposition reactions are far less explored, and have primarily focused on cyano^8^ or aryl group^9^ migrations. Precedence for our proposed distal alkene translocation has been limited to metal chain walking strategies,^10^ and a recent conceptually related example employing photo/cobalt dual catalysis protocol.^11^ However, we hypothesized that this type of tandem annulation–olefin migration reaction might be possible via an intramolecular radical hydrogen atom transfer mediated C–H functionalization approach through the judicious selection of an appropriate transition metal catalyst and ligand (Figure 1C).

To realize this transformation, we envisioned that a highly reactive σ-alkyl copper intermediate **15**, accessed via a regioselective oxycupration reaction between a copper catalyst and a readily accessible alkenol starting material (**13**) could undergo homolysis to provide the corresponding primary carbon-centered radical (**16**) that could be utilized toward a transannular 1,5-HAT-mediated C(sp^3^)–H functionalization.^12^ The resulting electron-rich secondary radical **17** could then be intercepted with a copper(II) catalyst to oxidatively regenerate an alkene residue embedded within the oxabicyclic scaffold by selectively removing two vicinal C–H bonds. Additionally, this transformation would be highly diastereoselective as a consequence of the stereochemical arrangement between the primary carbon-centered radical at C4 and the key C6 hydrogen atom of **16** positioned on the same side of the formed oxabicycle. Herein, we report a successful realization of a complexity-generating annulation and alkene transposition cascade that enables a modular preparation of three-dimensional oxabicyclic spirocyclic ethers in a single step utilizing an inexpensive copper catalyst in combination with a bidentate quinoline-pyridone ligand (**L5**).

## Results and Discussion

We began our investigation by selecting alkenol **13a** as our model substrate, which was easily accessed in two steps from commercially available materials. Inspired by seminal reports that demonstrated that copper salts can promote intramolecular addition of heteroatoms to alkenes,^13,5c^ we hypothesized that a judicious combination of catalyst, oxidant, and ligand tuning could promote the desired remote C(sp^3^)–H functionalization via an efficient 1,5-HAT process. Our initial studies found that employing copper(II) acetate, 1,10-phenanthroline (**L1**), and manganese dioxide (MnO_2_) in 1,2-dichloroethane provided the desired, spirocyclic ether **14a** possessing a transposed alkene in 10% yield as a single detectable diastereomer (Table 1). Analysis of the product mixture revealed that the undesired saturated cyclic ether **18** was obtained as the major product in 50% yield, suggesting the presence of a competitive radical quenching mechanism likely arising from an inefficient alkyl radical oxidation. Guided by these findings, we surveyed several commercially available Cu(II) and Cu(I) salts in our reaction, but these efforts did not result in an increase of the desired product **14a**. However, we found that the choice of the reaction oxidant had a prominent effect on this transformation. Specifically, the use of silver carbonate resulted in a substantial increase in the formation of **14a** (40%, entry 5).^14^ This outcome could be further improved by using dimethyl sulfoxide (DMSO) as the reaction solvent, providing the sought-after desaturated product in 62% yield. Among the examined reaction additives, we identified that catalytic amounts of the bulky pivalic acid could further increase the formation of **14a** to 74% (entry 11). While this transformation generally had a clean reaction profile with no detectable major side products besides **18**, we sought to further favor product formation by examining additional ligand scaffolds aimed at converting the unreacted starting material **13a** to the desired desaturated oxabicycle and minimizing the formation of **18**. Various substituted bipyridines known to enable a range of copper-catalyzed reactions failed to significantly improve the reaction outcome (entries 12 and 13).^15^ Kochi and co-workers showed that Cu(II) salts can oxidize alkyl radicals to generate olefins through a process termed “oxidative elimination”.^16^ Importantly, they found carboxylate X-type ligands were critical for this observed reactivity, which was later corroborated by Su,^15b^ Hartwig,^17^ and others.^18^ We reasoned that a related oxidative elimination process could be operative in our desaturation step, and we attempted to change the bidentate L,L-type bipyridine scaffold to the L,X-type quinoline–pyridone where the 2-hydroxy subunit is known to mimic a carboxylate moiety in palladium catalysis.^19^ Using the five-membered chelate **L4** ligand did not improve the reaction yield, but notably the formation of the undesired byproduct **18** was reduced. However, the use of the six-membered chelate quinoline–pyridone **L5**(20) that benefits from a favorable Thorpe–Ingold effect exhibited excellent reactivity in our transformation and gave the desired product **14a** in 93% yield with minute quantities of **18** and no detectable starting material (entry 15). We also attempted our transformation in the absence of the silver carbonate oxidant with increased loading of the copper catalyst and observed the formation of **14a** albeit in diminished yield (66%, entry 17).^21^

**Table 1:**
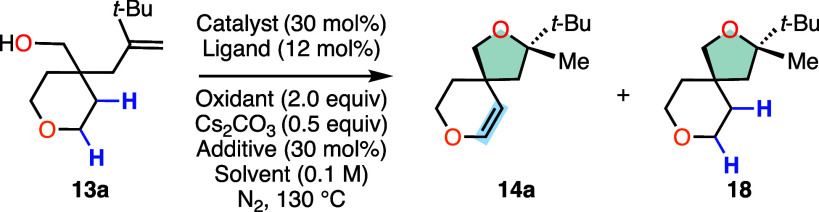

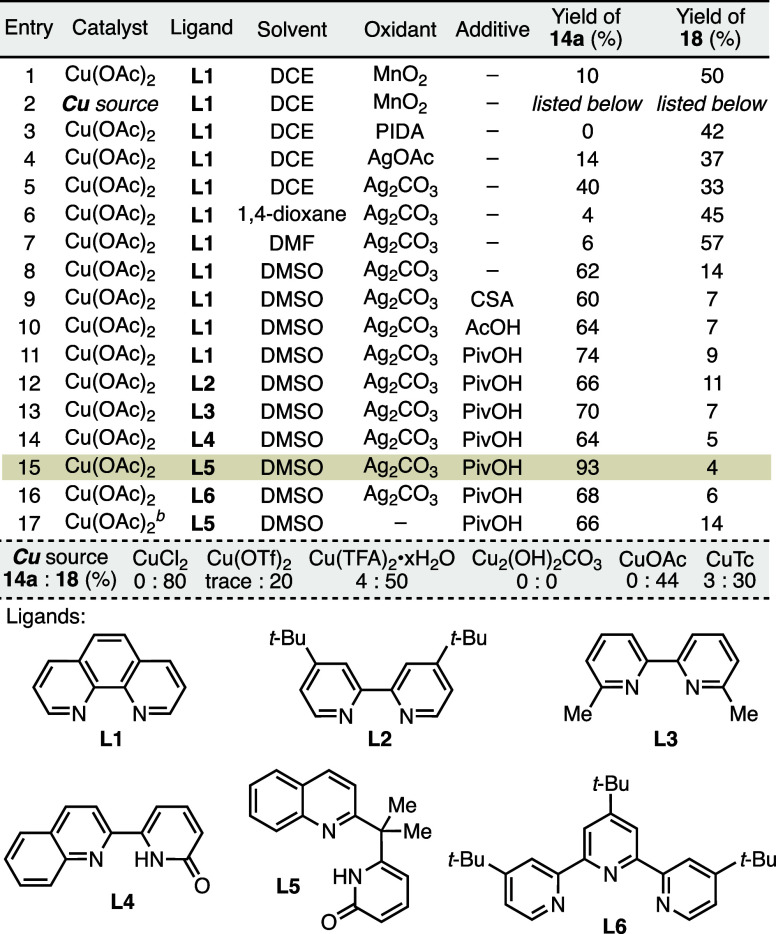
Reaction Discovery and Optimization of Reaction Conditions^a^^,^^b^^,^^c^

a Conditions: **13a** (0.10 mmol), catalyst (30 mol %), ligand (12 mol %), oxidant (2.0 equiv), Cs_2_CO_3_ (0.5 equiv), additive (30 mol %), solvent (1.0 mL), N_2_, 130 °C, 12h.

b Cu(OAc)_2_ (2.0 equiv).

c Yields were determined by ^1^H NMR analysis using CH_2_Br_2_ as an internal standard.

**Scheme 1:**
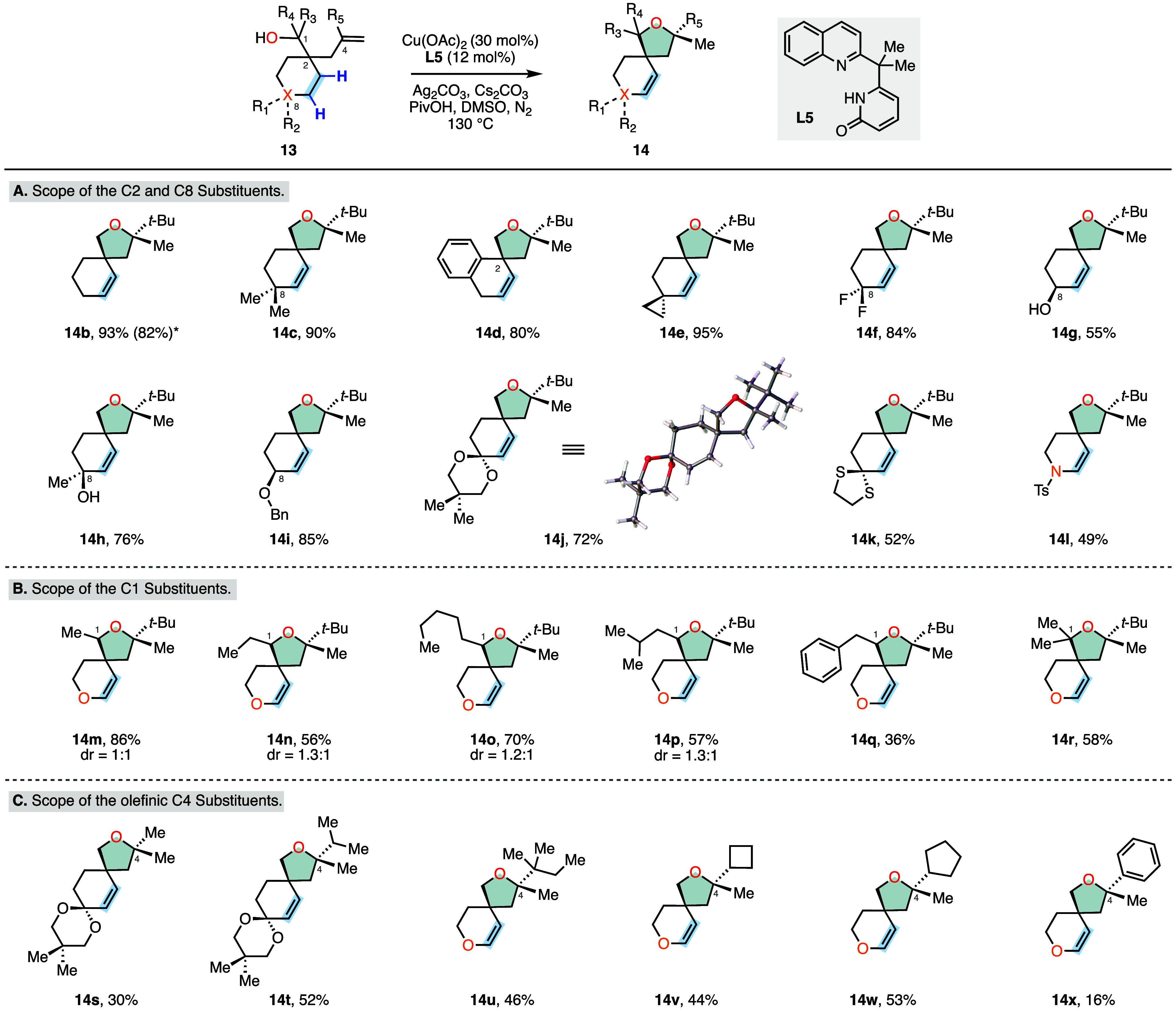
Substrate Scope of the Cu-Catalyzed Cyclization and Alkene Transposition Cascade^,^ Conditions: **13** (0.10 mmol), Cu(OAc)_2_ (30 mol %), **L5** (12 mol %), Ag_2_CO_3_ (2.0 equiv), Cs_2_CO_3_ (0.5 equiv), PivOH (30 mol %), DMSO (1.0 mL), N_2_, 130 °C, 12 h. Isolated yields are reported. *Isolated yield on 4.0 mmol scale.

Having identified the optimal reaction conditions, we studied the substrate scope of our transformation (Scheme 1). First, the scope with respect to the C2 and C8 substituents was investigated (Scheme 1A). Using a simple alkylation and reduction sequence (Supporting Information), we were able to prepare a wide range of annulation precursors. We found that simple alkyl substituents (**14b**–**14d**) provided the desired spirocyclic ethers in excellent yields ranging from 80 to 93%. The exocyclic cyclopropane (**14e**) and *gem*-difluoro (**14f**) substrates were also converted to products in high yields (95 and 84%, respectively). Notably, the unprotected secondary and tertiary hydroxyl groups that can competitively chelate with the copper catalyst were well tolerated and gave rise to products **14g** and **14h** in 55 and 76% yields. Additional C8 functional groups such as benzyl ether (**14i**), ketal (**14j**), and thioketal (**14k**) exhibited good reactivity and provided the desired ether products in 52–85% yields. Having developed our cascade reaction using substrate **13a** possessing a C8 oxygen, we also evaluated the corresponding tosyl-protected C8 nitrogen and observed formation of the synthetically useful enamide product **14l** in 49% yield. Additionally, we investigated the scalability of our transformation and isolated the product **14b** in 82% yield on a 4.0 mmol scale.

Our reaction protocol was also compatible with the incorporation of substituents at the C1 position of our annulation precursor (Scheme 1B). These substrates were prepared as inconsequential mixtures of diastereomers via nucleophilic addition to the corresponding C1 aldehydes. We found that simple aliphatic alkyl groups such as methyl (**14m**), ethyl (**14n**), and pentyl (**14o**) as well as the sterically demanding *iso*-butyl groups (**14p**) were well tolerated and afforded the desired bicyclic products in 56–86% yield. However, when a C1 benzyl substrate was subjected to our standard reaction conditions, we observed product formation in a reduced yield (**14q**, 36%) as a single isolable C1 diastereomer. We attributed this result to a preferential addition of the primary radical **16** to the nearby aryl group when these two substituents are positioned on the same face of the tetrahydrofuran ring over the desired 1,5-HAT step.^5c^ The addition of a second aliphatic substituent to the C1 position showed good reactivity, as demonstrated by the generation of the *gem*-dimethyl-containing product **14r** in 58%. Next, we examined the substrate scope with respect to the C4 olefinic residue (Scheme 1C). While the sterically demanding *tert*-butyl group provided the highest yields likely due to a favorable substrate preorganization for the transannular HAT step, our transformation proved to be compatible with an array of differentially substituted cyclic and acyclic olefinic residues. For example, the C4 methyl (**14s**), *iso*-propyl (**14t**), and trisubstituted **14u** olefins gave rise to the desired products in 30%, 52%, and 46% yields, respectively. The C4 cyclobutane (**14v**) and cyclopentane (**14w**) annulation precursors furnished the annulated products in 44% and 53% yields. The phenyl substituted substrate generated the bicyclic product **14x** in a modest 16% yield, presumably due to a competitive side reaction analogous to the substrate **14q** where the primary radical can add into the proximal phenyl group. It is also worth noting that substrates **14u**–**14w** possess additional accessible hydrogens that could undergo a competitive abstraction, potentially contributing to the decreased efficiency of the desired alkene transposition pathway.

**Scheme 2:**
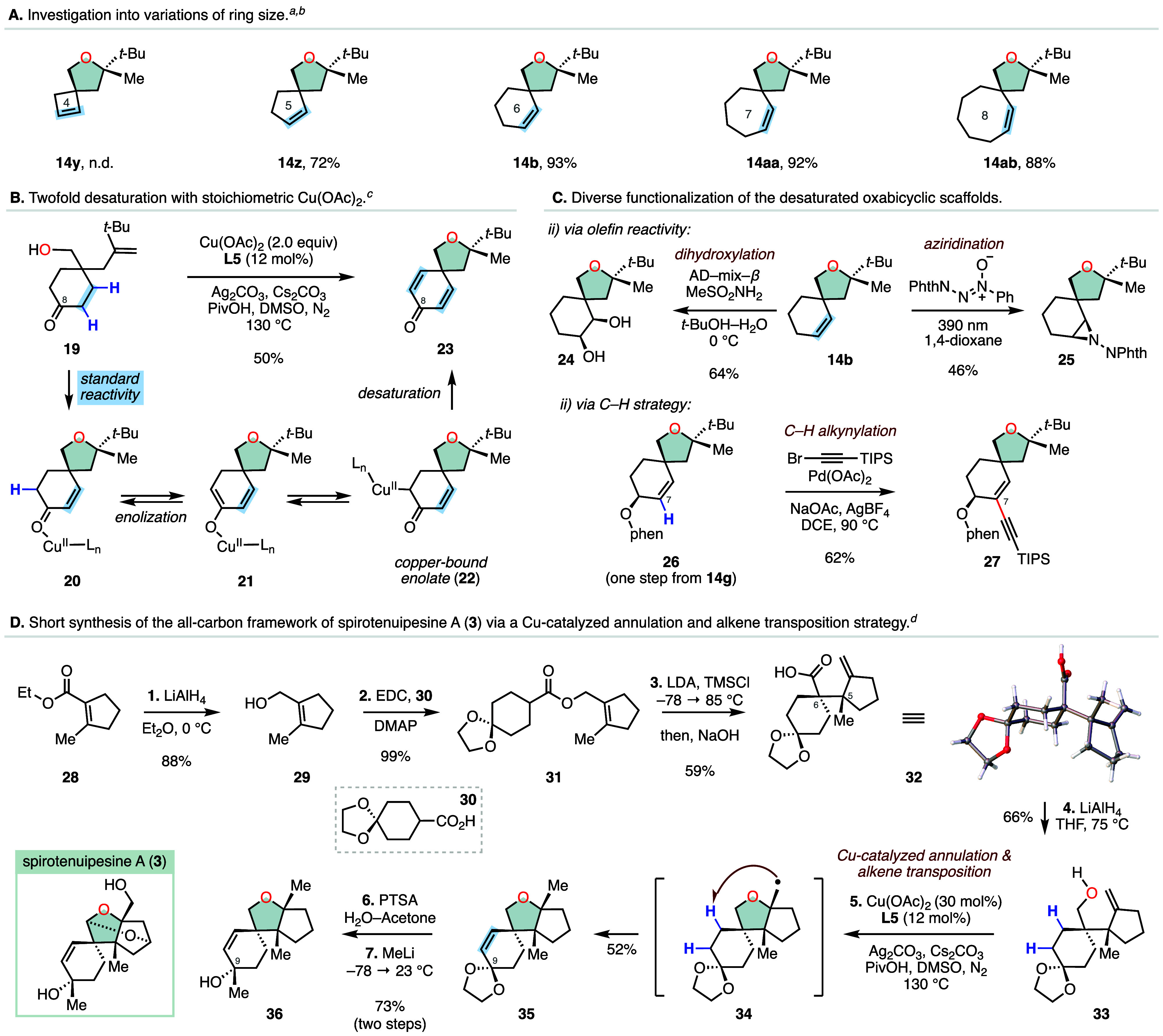
Synthetic Applications and a Short Total Synthesis of the All-Carbon Framework of Spirotenuipesine A^,^^,^^,^ Conditions: substrate (0.10 mmol), Cu(OAc)_2_ (30 mol %), **L5** (12 mol %), Ag_2_CO_3_ (2.0 equiv), Cs_2_CO_3_ (0.5 equiv), PivOH (30 mol %), DMSO (1.0 mL), N_2_, 130 °C, 12 h. Isolated yields are reported. Cu(OAc)_2_ (2.0 equiv). (1) LiAlH_4_ (1.1 equiv), Et_2_O, 0 °C; (2) EDC (2.0 equiv), DMAP (2.0 equiv), **30** (1.0 equiv), DCM; (3) LDA (2.05 equiv), TMSCl (2.10 equiv), THF, −78 → 85 °C, then NaOH (4.0 equiv), MeOH; (4) LiAlH_4_ (1.30 equiv), THF, 75 °C; (5) Cu(OAc)_2_ (30 mol %), **L5** (12 mol %), Ag_2_CO_3_ (2.0 equiv), Cs_2_CO_3_ (0.5 equiv), PivOH (30 mol %), DMSO, N_2_, 130 °C; (6) PTSA (0.3 equiv), H_2_O–Acetone (1:10 v/v); (7) MeLi (2.0 equiv), THF, −78 °C.

We were further interested in determining whether our transformation could be amenable to the preparation of desaturated bicyclic ethers with a varying number of carbon atoms in the central ring system (Scheme 2A). To this end, we synthesized the corresponding annulation precursors possessing four-, five-, seven-, and eight-membered central rings and subjected them to our optimized reaction conditions. With the cyclobutane substrate, we did not observe the formation of the desired product **14y**. We attribute this to the distortion of the requisite geometry from the four-membered ring following the initial cyclization, preventing an efficient 1,5-HAT step. Pleasingly, we found that all other ring-sizes were well tolerated and provided the corresponding desaturated bicyclic ethers in 72–93% yield, further demonstrating the effectiveness of this method for the preparation of medium-sized ring systems.

We also investigated if a second desaturation event could be triggered using a substrate containing a C8 carbonyl group to provide synthetically useful bis-desaturated oxabicycle **23** (Scheme 2B). Specifically, we envisioned that after the expected reaction sequence, the generated *α,β*-unsaturated ketone **20** could undergo a base-mediated enolization with copper acetate acting as a Lewis acid to form enolate **21**. The resulting copper-bound enolate is known to exhibit characteristic radical reactivity and could undergo a second desaturation event driven by another equivalent of copper acetate (**22** → **23**).^22a,18c,22b^ Our attempts to enable this transformation with catalytic amounts of copper were unsuccessful; however, we found that increasing the loading to two equivalents resulted in formation of the doubly desaturated oxabicycle **23** in 50% yield. Next, we turned our attention to derivatization of our desaturated scaffold through the functionalization of the alkene moiety. As demonstrated in Scheme 2C, several structurally diverse products were readily generated. For example, a dihydroxylation reaction (AD–mix−β) of **14b** provided diol **24** in 64% yield. A photoinduced aziridination reaction with 1-phenyl-2-phthalimidodiazene-1-oxide delivered the phthalimido-protected aziridine **25** in 46% yield.^23^ We also sought to derivatize the olefin residue via a carbon–carbon bond forming C–H functionalization strategy. To this end, the C7 position of **26** could be activated with a palladium catalyst and reacted with a (bromoethynyl)triisopropylsilane electrophile to synthesize the C–H alkynylated product **27** (64%).^24^

To further showcase the synthetic potential of our cyclization and remote dehydrogenation cascade, we applied this method to the synthesis of tertiary alcohol **36** that constitutes the all-carbon framework of the bioactive natural product spirotenuipesine A (**3**, Scheme 2D). Spirotenuipesine A was isolated from the fungus *Paecilomyces tenuipes* in 2004 and displays potent activity in promoting neurotrophic factor biosynthesis in the 1321N1 human astrocytoma cell line.^25^ Our synthesis began with the readily available ethyl ester **28** that was reduced with lithium aluminum hydride (LiAlH_4_) to the allylic alcohol **29** (88%). Coupling of **29** with carboxylic acid **30** provided ester **31**. Next, deprotonation and trapping of the resulting enolate with trimethylsilyl chloride gave a silylketene acetal (not isolated) that upon heating (85 °C) underwent an Ireland–Claisen rearrangement to furnish the crystalline carboxylic acid **32** (59%). Reduction of the hindered carboxylic acid residue required heating **32** with excess LiAlH_4_ and formed the annulation precursor **33** for our key step in 66% yield. We found that exposure of the complex primary alcohol **33** to our developed Cu-catalyzed cyclization and remote dehydrogenation protocol gave the expected tetracycle **35** in 52%. The all-carbon-framework of **3** could then be completed in two additional steps via an acid-catalyzed deprotection of the ketal moiety (*p*-toluenesulfonic acid) and a pro-axial methyl lithium addition to the C9 carbonyl providing the tertiary alcohol **36** in 73% over two steps.^26^ Our work toward this complex synthetic framework highlights the utility of our annulation to provide efficient access to structurally complex building blocks and indicates that this powerful transformation may find use in the synthesis of other natural products.

**Scheme 3:**
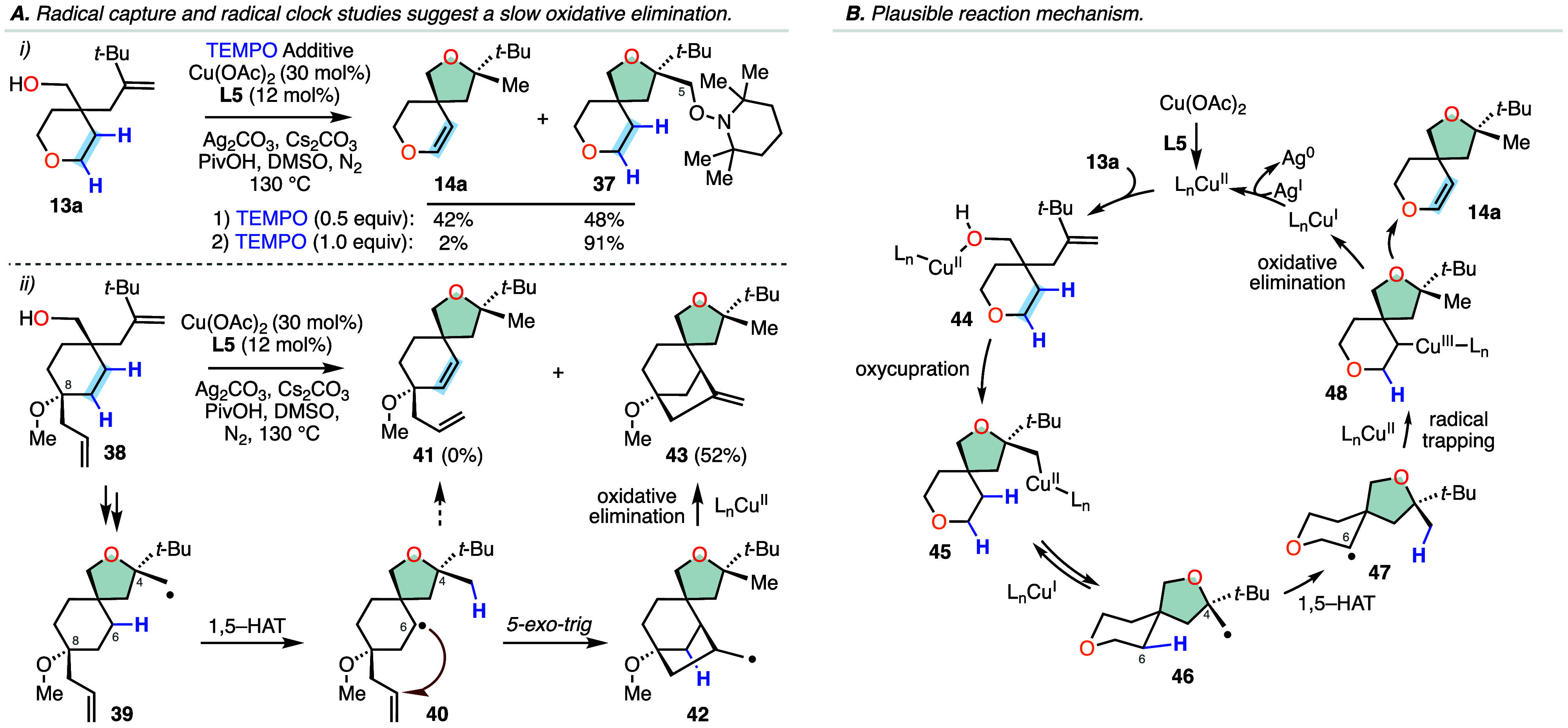
Radical Probes and Plausible Reaction Mechanism

A series of experiments were devised to increase our mechanistic understanding of the annulation and remote dehydrogenation cascade (Scheme 3). First, we found that employing radical traps such as the persistent radical 2,2,6,6-tetramethyl-1-piperidinyloxy (TEMPO) with our standard reaction conditions inhibited our transformation, confirming a radical-based mechanism (Scheme 3A). Specifically, employing 0.5 equivalents of TEMPO resulted in formation of 48% of the TEMPO-adduct **37** while 1.0 equivalent provided 91% of **37**. In both instances, the TEMPO addition occurred exclusively at the primary C5 position and no adducts derived from addition of TEMPO to the translocated secondary C-centered radical were detected. Next, a radical clock experiment was performed to investigate the rate of the second C–H bond cleavage step from the translocated radical **17** (Scheme 3A, part (ii). To this end, we synthesized substrate **38** possessing a C8 allyl substituent (see Supporting Information) and subjected it to our optimized reaction conditions. Using the allylic substrate **38**, we did not detect any formation of the standard product **41** but rather observed the formation of the tricyclic product **43** possessing an exocyclic olefin in 52% yield. This result indicates that the translocated secondary C-centered radical undergoes a fast 5-*exo*-trig radical addition to the pendant olefin forming new primary radical **42**, which then readily desaturates to provide the exocyclic olefin product. It is known that 5-hexenyl radical cyclizations proceed with a rate faster than 10^5^ s^–1^,^27^ indicating that by comparison the cleavage of the second C–H bond proceeds at a slower rate in our reaction system. This is consistent with observations reported by Nagib and co-workers in their C–H desaturation of amines.^28^ Based on these experiments, we propose that our transformation proceeds via the catalytic cycle shown in Scheme 3B. Presumably, after coordination of Cu(OAc)_2_ with ligand **L5**, the active catalyst formed performs a copper(II)-catalyzed alkene oxycupration reaction to provide a σ-alkyl copper complex **45**. Homolysis of the weak carbon–copper bond provides a Cu(I) species and a key primary alkyl radical intermediate **46** that undergoes a transannular 1,5-HAT-mediated C(sp^3^)–H functionalization step to form secondary radical **47**. Recombination of the radical **47** with a Cu(II) species generates an alkyl copper(III) intermediate **48** that can undergo a concerted β-oxidative elimination as proposed by Kochi^16a^ and others^18a,18c,28,29^ cleaving the second vicinal C–H bond and thereby transposing the alkene residue within the tethered carbocycle furnishing the desired product **14a**. Finally, the oxidation of the generated Cu(I) species with silver carbonate regenerates the active catalyst and completes the catalytic cycle. While we do not believe that silver plays any additional role beyond reoxidation of the copper catalyst in our cascade, it is evident that the use of catalytic copper conditions improves the yield of our transformation and results in an overall cleaner reaction profile with minimal impurities.

## Conclusion

In summary, we have developed a novel and efficient synthetic strategy to synthesize three-dimensional and structurally complex spirocyclic ether scaffolds via a copper-catalyzed cyclization cascade. This transformation, which constitutes a formal annulation–olefin migration sequence, transforms readily available alkenol substrates into privileged oxygen-containing heterocycles via a transannular 1,5-HAT-mediated C(sp^3^)–H functionalization and organocopper(III)-mediated oxidative elimination sequence. Our method utilizes a combination of an inexpensive copper(II) acetate catalyst and tautomeric L,X-type quinoline–pyridone ligand **L5** without the need for any exogenous directing groups to deliver the desired annulation products in efficient yields. Moreover, this annulation possesses a broad substrate scope, shows excellent functional group and heteroatom compatibility, and can be readily applied to the preparation of spirocyclic ethers with a varying ring size. We also demonstrate the synthetic utility of the transposed alkene residue with a broad derivatization effort to highlight the potential for a rapid and valuable scaffold diversification. Additionally, our short and direct synthesis of the all-carbon framework of the bioactive natural product spirotenuipesine A (**3**) using this strategy underscores the utility of this method for complex molecule synthesis. Lastly, our new method addresses the increasing demands for streamlining the preparation of three-dimensional molecular scaffolds and provides a compelling strategy for rapidly generating libraries of structurally unique compounds with bioactive potential.

## Supporting Information Available

General information; substrate structures; characterization of substrates and products; crystallographic analysis of **14j** and **32**; catalogue of ^1^H NMR and ^13^C NMR spectra (PDF)

## Abbreviations

DCE: 1,2-dichloroethane
DMAP: 4-(dimethylamino)pyridine
DMF: dimethylformamide
CSA: camphorsulfonic acid
Phen: phenanthroline
PIDA: (diacetoxyiodo)benzene
PivOH: pivalic acid
Pthth: phthaloyl
PTSA: p-toluenesulfonic acid
TEMPO: 2,2,6,6-tetrmethylpiperidine
TFA: trifluoroacetic acid
Ts: p-toluenesulfonyl.
