# Supplementary material for: Copper-Catalyzed Cyclization and Alkene Transposition Cascade Enables a Modular Synthesis of Complex Spirocyclic Ethers
Source: J Am Chem Soc. 2024 Dec 20;147(1):1034–41. doi: 10.1021/jacs.4c14418 (PMC11726577; doi:10.1021/jacs.4c14418)

**Copper–Catalyzed Cyclization and Alkene Transposition Cascade Enables a Modular Synthesis of Complex Spirocyclic Ethers.**

Wan-Xu Wei,[1] Yangjin Kuang,[1] and Martin Tomanik[1,*]

[1]Department of Chemistry, New York University, New York, New York, 10003, United States.

Supporting Information

**Table of Contents:**

General Information ........ S2
Substrate Structures ........ S3
Experimental Section ........ S4
General Procedure A: Preparation of Substrates **13a–13f**, **13i–13l**, and **13y–13ab** ........ S3
General Procedure B: Preparation of Substrates **13g** and **13h** ........ S6
General Procedure C: Preparation of Substrates **13m–13q** ........ S9
Synthetic Procedure for the Preparation of Substrate **13r** ........ S10
General Procedure D: Preparation of Substrates **13s–13x** ........ S11
General Procedure E: Copper–Catalyzed Cyclization and Remote Dehydrogenation ... S12
Synthetic Procedure for the Preparation of Substrate **19** ........ S13
Synthesis of the Doubly Desaturated Product **23** ........ S14
List of Unsuccessful or Limited Reactivity Substrates ........ S15
Reaction Optimization Tables ........ S16
Synthetic Procedure for Preparation of **24**, **25**, and **27** ........ S17
Synthesis of **36** Possessing the All-carbon Framework of Spirotenuipesines A (**3**) ........ S20
Radical Trapping Experiments with TEMPO ........ S27
Synthesis of the Radical Clock Substrate **38** ........ S28
Radical Clock Experiment with Substrate **38** ........ S31
Synthetic Procedure for Preparation of ligand **L5** ........ S32
Characterization of Substrates and Products ........ S34
Crystallographic Analysis of **14j** and **32** ........ S54
References ........ S58
Catalogue of $^{1}$H NMR and $^{13}$C NMR Spectra ........ S59

**General Information.**

Unless otherwise stated, all reagents were purchased from commercial suppliers and used without further purification. Anhydrous solvents were obtained from the solvent purification system produced by *JC* Meyer Solvent Systems. Analytical thin-layer chromatography (TLC) was performed on Merck Millipore precoated (0.25 mm thickness) silica gel plates with F254 fluorescent indicator. TLC plates were visualized by exposure to ultraviolet light (UV) and/or submersion in aqueous potassium permanganate solution ($KMnO_4$), ceric ammonium molybdate solution (CAM), *para*-anisaldehyde (PAA) and followed by brief heating on a hot plate (120 °C, 10–15 s). Flash-column chromatography was performed employing silica gel (32-63 µm particle size) supplied by Dynamic Adsorbents. Proton nuclear magnetic resonance spectra ($^1$H NMR) were recorded on a Bruker instrument (400, 500, or 600 MHz). Chemical shifts are expressed in parts per million (ppm, δ scale) downfield from tetramethylsilane and are referenced to residual protium in the NMR solvent ($CHCl_3$, δ 7.26; $C_6D_5H$, δ 7.16). Data are represented as follows: chemical shift, multiplicity (s = singlet, d = doublet, t = triplet, q = quartet, m = multiplet and/or multiple resonances, b = broad, app = apparent), coupling constant, *J*, in Hertz (Hz) AND integration. Proton-decoupled carbon nuclear magnetic resonance spectra ($^{13}$C NMR) were recorded on a Bruker instrument (101, 126, or 151 MHz). Chemical shifts are expressed in parts per million (ppm, δ scale) downfield from tetramethylsilane and are referenced to the carbon resonances of the solvent ($CDCl_3$, δ 77.2; Benzene-$d_6$, δ 128.1). High-resolution mass spectra (HRMS) were recorded on an Agilent Mass spectrometer using ESI-TOF (electrospray ionization-time of flight).

## Substrate Structures.

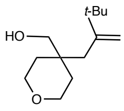


**13a**

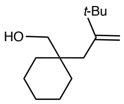


**13b**

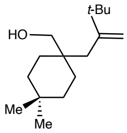


**13c**

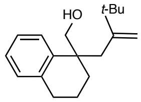


**13d**

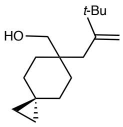


**13e**

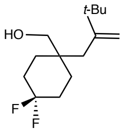


**13f**

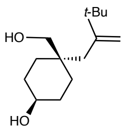


**13g**

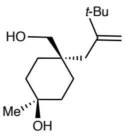


**13h**

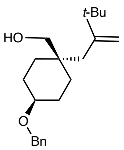


**13i**

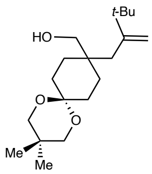


**13j**

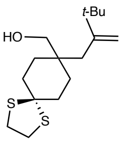


**13k**

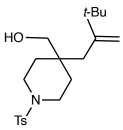


**13l**

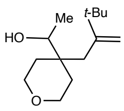


**13m**

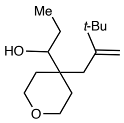


**13n**

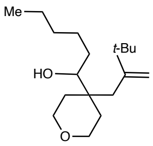


**13o**

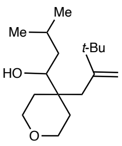


**13p**

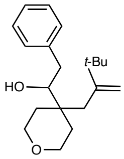


**13q**

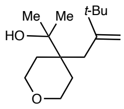


**13r**

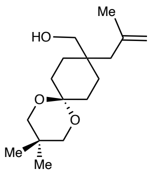


**13s**

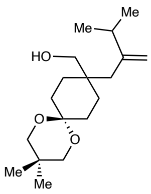


**13t**

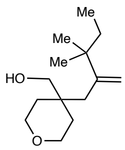


**13u**

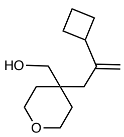


**13v**

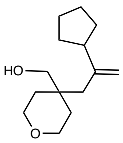


**13w**

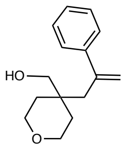


**13x**

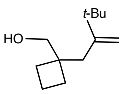


**13y**

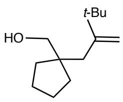


**13z**

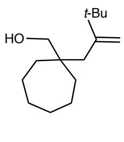


**13aa**

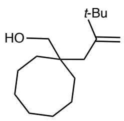


**13ab**

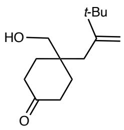


**19**

## Experimental Section.

### General Procedure A: Preparation of Substrates 13a–13f, 13i–13l, and 13y–13ab.

*Synthesis of the esters **S3**:*

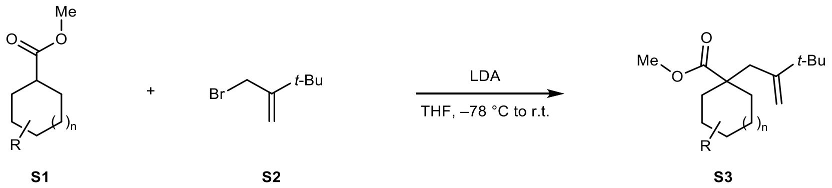

A solution of n-butyllithium in hexanes (2.50 M, 1.20 equiv.) was added dropwise via syringe over 30 min to a solution of diisopropylamine (1.25 equiv.) in tetrahydrofuran (0.2 M) at –78 °C. The resulting solution was stirred for 45 min at –78 °C. A solution of the ester **S1** (1.0 equiv.) in tetrahydrofuran was then added dropwise via syringe over 15 min at –78 °C. Upon completion of the addition, the reaction mixture was stirred for 1 hour at –78 °C. 2-(bromomethyl)-3,3-dimethylbut-1-ene (**S2**)[1] (1.2 equiv.) was then added dropwise via syringe at –78 °C. The reaction mixture was allowed to slowly warm to 23 °C overnight. The warmed product mixture was diluted sequentially with water and ethyl acetate. The resulting biphasic mixture was transferred to a separatory funnel and the layers that formed were separated. The aqueous layer was extracted with ethyl acetate. The organic layers were combined, and the combined organic layers were washed with saturated aqueous sodium chloride solution. The washed organic layer was dried over sodium sulfate. The dried solution was filtered and the filtrate was concentrated. The residue obtained was eluted over a short plug of silica gel with 20% ethyl acetate–hexanes to provide the corresponding alkylated ester **S3** as a colorless oil, which was used directly in the next step without any further purification.

*Synthesis of the alkenol **13a–13f, 13i-13l** and **13y–13ab** substrates:*

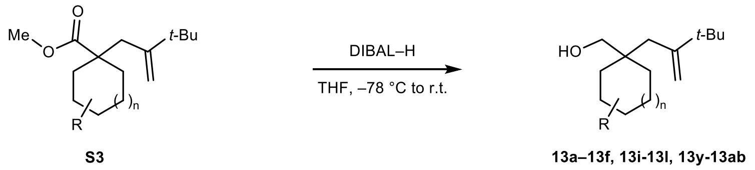

A solution of diisobutylaluminium hydride in toluene (1.00 M, 3.00 equiv) was added dropwise via syringe to a solution of the ester **S3** (1 equiv) in tetrahydrofuran (0.2 M) at –78 °C. The reaction mixture was allowed to slowly warm to 23 °C overnight. The warmed product mixture was diluted sequentially saturated aqueous potassium sodium tartrate tetrahydrate solution and ethyl acetate. The resulting biphasic mixture was allowed to stir for 1 hour at 23 °C. The product mixture was then transferred to a separatory funnel and the layers that formed were separated. The aqueous layer was extracted with ethyl acetate. The organic layers were combined, and the combined organic layers were washed with saturated aqueous sodium chloride solution. The washed organic layer was dried over sodium sulfate. The dried solution was filtered and the filtrate was

concentrated. The residue obtained was purified by flash-column chromatography to provide the corresponding alkenol substrates **13a–13f**, **13i-13l** and **13y–13ab** as colorless oils.

## General Procedure B: Preparation of Substrates 13g and 13h.

*Synthesis of the alcohols **S6** and **S7**:*

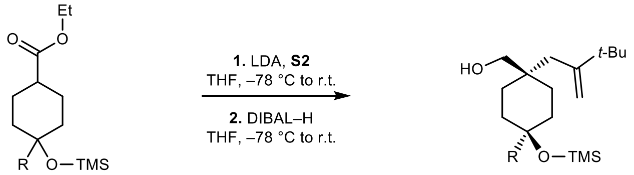


R = H (**S4**)
R = Me (**S5**)

R = H (**S6**)
R = Me (**S7**)

A solution of n-butyllithium in hexanes (2.50 M, 1.20 equiv.) was added dropwise via syringe over 30 min to a solution of diisopropylamine (1.25 equiv.) in tetrahydrofuran (0.2 M) at –78 °C. The resulting solution was stirred for 45 min at –78 °C. A solution of the known ester **S4**[2] or **S5**[3] (1.0 equiv.) in tetrahydrofuran was then added dropwise via syringe over 15 min at –78 °C. Upon completion of the addition, the reaction mixture was stirred for 1 hour at –78 °C. 2-(bromomethyl)-3,3-dimethylbut-1-ene (**S2**)[1] (1.2 equiv.) was then added dropwise via syringe at –78 °C. The reaction mixture was allowed to slowly warm to 23 °C overnight. The warmed product mixture was diluted sequentially with water and ethyl acetate. The resulting biphasic mixture was transferred to a separatory funnel and the layers that formed were separated. The aqueous layer was extracted with ethyl acetate. The organic layers were combined, and the combined organic layers were washed with saturated aqueous sodium chloride solution. The washed organic layer was dried over sodium sulfate. The dried solution was filtered, and the filtrate was concentrated. The residue obtained was eluted over a short plug of silica gel with 20% ethyl acetate–hexanes and was subsequently used directly in the next step. A solution of diisobutylaluminium hydride in toluene (1.00 M, 3.00 equiv) was added dropwise via syringe to a solution of the residue obtained (1 equiv) dissolved in tetrahydrofuran (0.2 M) at –78 °C. The reaction mixture was allowed to slowly warm to 23 °C overnight. The warmed product mixture was diluted sequentially saturated aqueous potassium sodium tartrate tetrahydrate solution and ethyl acetate. The resulting biphasic mixture was allowed to stir for 1 hour at 23 °C. The product mixture was then transferred to a separatory funnel and the layers that formed were separated. The aqueous layer was extracted with ethyl acetate. The organic layers were combined, and the combined organic layers were washed with saturated aqueous sodium chloride solution. The washed organic layer was dried over sodium sulfate. The dried solution was filtered, and the filtrate was concentrated. The residue obtained containing the corresponding primary alcohols **S6** or **S7** was used directly in the subsequent step without further purification.

*Synthesis of the alkenol substrates* ***13g*** *and* ***13h****:*

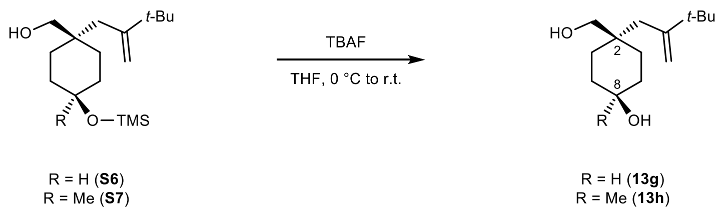


A solution of tetrabutylammonium fluoride (TBAF, 1.00M in THF, 2.00 equiv) was added dropwise via syringe over 10 min to a solution of the alcohol **S6** and **S7** (1 equiv) obtained in the previous step dissolved in tetrahydrofuran (0.2 M) at 0 °C. The reaction mixture was stirred for 5 hours at 0 °C. The cold product mixture was diluted sequentially with saturated aqueous ammonium chloride solution, saturated aqueous sodium chloride solution, and ethyl acetate. The diluted product mixture was then allowed to warm to 23 °C over 20 min. The warmed biphasic mixture was transferred to a separatory funnel and the layers that formed were separated. The aqueous layer was extracted with ethyl acetate. The organic layers were combined, and the combined organic layers were washed with saturated aqueous sodium chloride solution. The washed organic layer was dried over sodium sulfate. The dried solution was filtered and the filtrate was concentrated. The residue obtained was purified by flash-column chromatography to provide the corresponding secondary alkenol substrates **13g** and **13h** as a colorless oil and as a separable mixture of diastereomers. The relative stereochemistry of the products was assigned using NOE analysis and the shown diastereomer was used in our developed copper-catalysed annulation and dehydrogenation transformation.

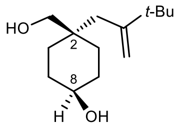


**13g**

$R_f$ = 0.4 (60% ethyl acetate–hexanes). $^1$H NMR (400 MHz, Benzene-$d_6$): δ 5.11 (d, $J$ = 1.1 Hz, 1H), 4.94 (d, $J$ = 1.1 Hz, 1H), 3.39 (s, 2H), 3.35 – 3.28 (m, 1H), 2.03 (d, $J$ = 1.1 Hz, 2H), 1.64 – 1.54 (m, 4H), 1.29 – 1.21 (m, 2H), 1.19 – 1.11 (m, 2H), 1.05 (s, 9H). $^{13}$C NMR (101 MHz, Benzene-$d_6$): δ 154.8, 110.2, 70.0, 64.6, 38.1, 38.1, 37.2, 31.3, 31.2, 29.8. HRMS-CI (m/z): [M + H]$^+$ calcd for $C_{14}H_{27}O_2$, 227.2011; found, 227.2012.

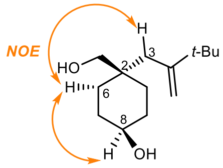


**13g**

Note: The relative stereochemistry at the C2 and C8 position was established by NOE analysis. Correlations between the hydrogen at C3 and the hydrogen at C6 as well as correlations between the hydrogen at C6 and the hydrogen at C8 support the relative assignment shown.

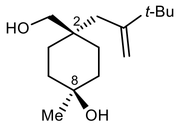


**13h**

$R_f$ = 0.60 (50% ethyl acetate–hexanes). $^{1}$H NMR (400 MHz, Benzene-$d_6$): δ 5.15 (s, 1H), 5.05 (s, 1H), 3.42 (s, 2H), 2.14 (s, 2H), 1.72 – 1.63 (m, 2H), 1.48 – 1.41 (m, 2H), 1.37 – 1.27 (m, 4H), 1.08 (s, 9H), 1.05 (s, 3H). $^{13}$C NMR (101 MHz, Benzene-$d_6$): δ 154.9, 110.2, 68.6, 64.3, 38.7, 38.1, 37.2, 35.1, 30.8, 29.8, 29.4. HRMS-CI (m/z): $[M + H]^+$ calcd for $C_{15}H_{29}O_2$, 241.2168; found, 241.2171.

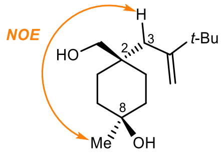


**13h**

Note: The relative stereochemistry at the C2 and C8 position was established by NOE analysis. Correlations between the hydrogen at C3 and the methyl group at C8 support the relative assignment shown.

## General Procedure C: Preparation of Substrates 13m–13q.

*Synthesis of the aldehyde **S8**:*

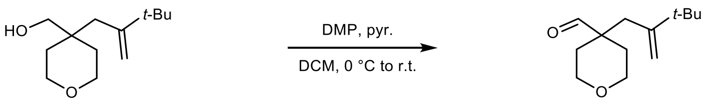


**13a** **S8**

The Dess–Martin periodinane (2.00 equiv) was added in three equal portions over 30 minutes to a solution of the primary alcohol **13a** (1 equiv) and pyridine (10.0 equiv) in dichloromethane (0.2 M) at 0 °C. Upon completion of the addition, the cooling bath was removed and the reaction mixture was warmed to 23 °C over 30 min. The warmed mixture was stirred for 2 h at 23 °C. The product mixture was diluted sequentially with dichloromethane, saturated aqueous sodium bicarbonate solution, and saturated aqueous sodium thiosulfate solution. The diluted product mixture was stirred for 30 minutes at 23 °C. The resulting biphasic mixture was transferred to a separatory funnel and the layers that formed were separated. The aqueous layer was extracted with dichloromethane. The organic layers were combined and the combined organic layers were washed with saturated aqueous sodium chloride solution. The washed organic layer was dried over sodium sulfate. The dried solution was filtered and the filtrate was concentrated. The residue obtained was eluted over a short plug of silica gel with 30% ethyl acetate–hexanes to provide the corresponding aldehyde **S8** as a colorless oil, which was used directly in the next step without any further purification

*Synthesis of the secondary alkenol substrates **13m–13q**:*

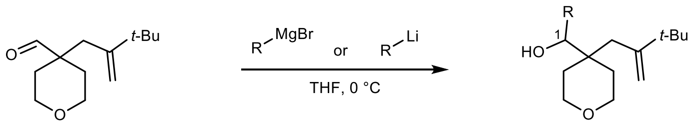


**S8** **13m–13q**

A solution of the corresponding Grignard reagent or alkyl lithium (1.50 equiv) was added dropwise via syringe over 10 min to a solution of the aldehyde **S8** (1 equiv) in tetrahydrofuran (0.2 M) at 0 °C. The reaction mixture was stirred for 2 hours at 0 °C. The cold product mixture was diluted sequentially with saturated aqueous ammonium chloride solution, saturated aqueous sodium chloride solution, and ethyl acetate. The diluted product mixture was then allowed to warm to 23 °C over 20 min. The warmed biphasic mixture was transferred to a separatory funnel and the layers that formed were separated. The aqueous layer was extracted with ethyl acetate. The organic layers were combined, and the combined organic layers were washed with saturated aqueous sodium chloride solution. The washed organic layer was dried over sodium sulfate. The dried solution was filtered and the filtrate was concentrated. The residue obtained was purified by flash-column chromatography to provide the corresponding secondary alkenol substrates **13m–13q** as colorless oils.

## Synthetic Procedure for the Preparation of Substrate 13r.

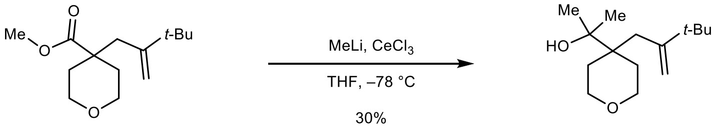


S9 → 13r

Cerium (III) chloride (862 mg, 3.50 mmol, 0.50 equiv) was added in one portion to a solution of the ester **S9** (nominally 7.0 mmol, 1 equiv) in tetrahydrofuran (35 mL) at 23 °C. The resulting solution was allowed to stir for 30 min at 23 °C and subsequently cooled to –78 °C. A solution of methyl lithium in tetrahydrofuran (1.00 M, 21.0 mL, 21.0 mmol, 3.0 equiv) was then added dropwise to the reaction mixture at –78 °C. Upon completion of the addition, the reaction mixture was stirred for 2 h at –78 °C. The cold product mixture was diluted sequentially with saturated aqueous ammonium chloride solution (30 mL), water (30 mL), and ethyl acetate (30 mL). The resulting biphasic mixture was transferred to a separatory funnel and the layers that formed were separated. The aqueous layer was extracted with ethyl acetate (3 × 30 mL). The organic layers were combined and the combined organic layers were washed with saturated aqueous sodium chloride solution (30 mL). The washed organic layer was dried over sodium sulfate. The dried solution was filtered, and the filtrate was concentrated. The residue obtained was purified by flash-column chromatography (eluting with 15% ethyl acetate–hexanes) to provide the tertiary alcohol **13r** as a colorless oil (500 mg, 30%).

$R_f$ = 0.50 (30% ethyl acetate–hexanes; PAA). $^1$H NMR (400 MHz, Benzene-$d_6$): δ 5.10 (s, 1H), 5.07 (s, 1H), 3.85 – 3.80 (m, 2H), 3.63 – 3.56 (m, 2H), 2.28 (s, 2H), 1.79 – 1.71 (m, 2H), 1.47 – 1.42 (m, 2H), 1.06 (s, 9H), 1.05 (s, 6H). $^{13}$C NMR (101 MHz, Benzene-$d_6$): δ 155.5, 109.8, 76.0, 64.4, 42.9, 38.1, 31.5, 31.4, 30.0, 26.7. HRMS-CI (m/z): $[M + H]^+$ calcd for $C_{15}H_{29}O_2$, 241.2168; found, 241.2165.

**General Procedure D: Preparation of Substrates 13s–13x.**

*Synthesis of the esters **S12**:*

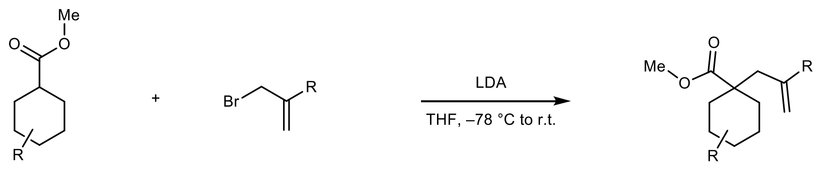


A solution of *n*-butyllithium in hexanes (2.50 M, 1.20 equiv.) was added dropwise via syringe over 30 min to a solution of diisopropylamine (1.25 equiv.) in tetrahydrofuran (0.2 M) at –78 °C. The resulting solution was stirred for 45 min at –78 °C. A solution of the ester **S10** (1.0 equiv.) in tetrahydrofuran was then added dropwise via syringe over 15 min at –78 °C. Upon completion of the addition, the reaction mixture was stirred for 1 hour at –78 °C. The corresponding alkyl bromide (**S11**)[4-7] (1.2 equiv.) was then added dropwise via syringe at –78 °C. The reaction mixture was allowed to slowly warm to 23 °C overnight. The warmed product mixture was diluted sequentially with water and ethyl acetate. The resulting biphasic mixture was transferred to a separatory funnel and the layers that formed were separated. The aqueous layer was extracted with ethyl acetate. The organic layers were combined, and the combined organic layers were washed with saturated aqueous sodium chloride solution. The washed organic layer was dried over sodium sulfate. The dried solution was filtered and the filtrate was concentrated. The residue obtained was eluted over a short plug of silica gel with 20% ethyl acetate–hexanes to provide the corresponding alkylated ester **S12** as a colorless oil, which was used directly in the next step without any further purification.

*Synthesis of the alkenol substrates **13s–13x**:*

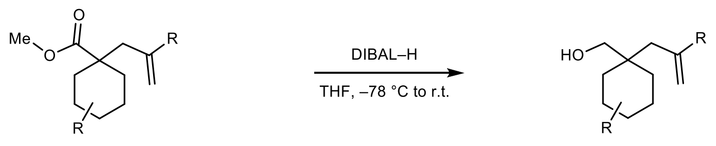


A solution of diisobutylaluminium hydride in toluene (1.00 M, 3.00 equiv) was added dropwise via syringe to a solution of the ester **S12** (1 equiv) in tetrahydrofuran (0.2 M) at –78 °C. The reaction mixture was allowed to slowly warm to 23 °C overnight. The warmed product mixture was diluted sequentially saturated aqueous potassium sodium tartrate tetrahydrate solution and ethyl acetate. The resulting biphasic mixture was allowed to stir for 1 hour at 23 °C. The product mixture was then transferred to a separatory funnel and the layers that formed were separated. The aqueous layer was extracted with ethyl acetate. The organic layers were combined, and the combined organic layers were washed with saturated aqueous sodium chloride solution. The washed organic layer was dried over sodium sulfate. The dried solution was filtered and the filtrate was concentrated. The residue obtained was purified by flash-column chromatography to provide the corresponding alkenol substrates **13s–13x** as colorless oils.

**General Procedure E: Copper–catalysed cyclization and remote dehydrogenation.**

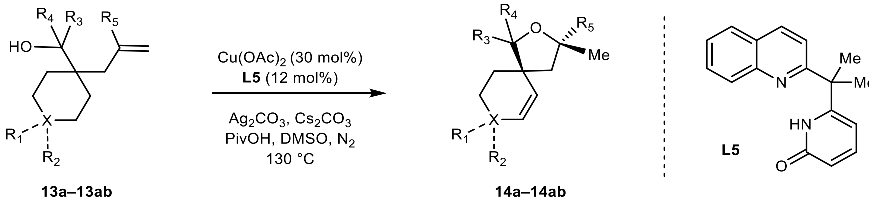


A screw-capped 16 x 125 mm culture tube was sequentially charged under air with the alkenol starting material **13a–13ab** (0.10 mmol, 1 equiv), $Cu(OAc)_2$ (5.45 mg, 30.0 μmol, 0.30 equiv), ligand (**L5**)[8] (3.17 mg, 12.0 μmol, 0.12 equiv), silver carbonate (55.2 mg, 0.20 mmol, 2.00 equiv), and cesium carbonate (16.3 mg, 50.0 μmol, 0.50 equiv). The reaction vessel was then sealed using a screw cap with a teflon coated septa. The reaction vessel was then placed under nitrogen atmosphere by using a vacuum manifold with a process of evacuating the headspace for 1.0 minute and venting with nitrogen for five times. Lastly, a pivalic acid (3.10 mg, 30.0 μmol, 0.30 equiv) dissolved in DMSO (1.0 mL) was added via a syringe to the reaction mixture at 23 °C. The reaction vessel was then placed into an oil bath that had been preheated to 130 °C. The reaction mixture was allowed to stir for 12 hours at 130 °C. After being allowed to cool to room temperature, the product mixture was diluted with ethyl acetate (10 mL) and water (10 mL). The resulting biphasic mixture was transferred to a separatory funnel and the layers that formed were separated. The aqueous layer was extracted with ethyl acetate (3 × 10 mL). The organic layers were combined, and the combined organic layers were washed with saturated aqueous sodium chloride solution (10 mL). The washed organic layer was dried over sodium sulfate. The dried solution was filtered and the filtrate was concentrated. The residue obtained was purified by flash-column or preparative thin-layer chromatography to provide the desired bicyclic ether products **14a–14ab**.

**Synthetic Procedure for the preparation of substrate 19.**

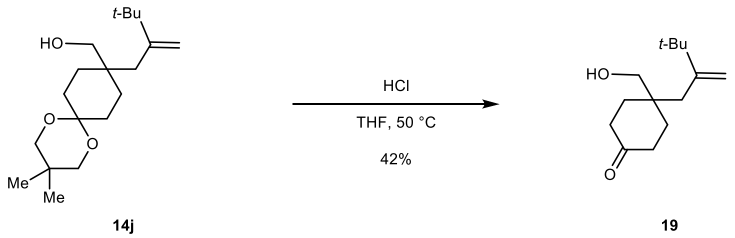

Hydrochloric acid (2M, 6.50 mg, 13.0 mmol, 16.0 equiv) was added to a solution of the primary alcohol **14j**, which was prepared according general procedure A (250 mg, 805 μmol, 1 equiv) in tetrahydrofuran (11.5 mL) at 23 °C. The reaction mixture was then placed into an oil bath that had been preheated to 50 °C. The reaction mixture was allowed to stir for 3 hours at 50 °C. After being allowed to cool to room temperature, the product mixture was diluted with ethyl acetate (20 mL) and water (20 mL). The resulting biphasic mixture was transferred to a separatory funnel and the layers that formed were separated. The aqueous layer was extracted with ethyl acetate (3 × 10 mL). The organic layers were combined and the combined organic layers were washed with saturated aqueous sodium chloride solution (20 mL). The washed organic layer was dried over sodium sulfate. The dried solution was filtered, and the filtrate was concentrated. The residue obtained was purified by flash-column chromatography (eluting with 20% ethyl acetate–hexanes) to provide the C7 ketone substrate **19** as a colorless oil (76.0 mg, 42%).

Note: Substrate **19** was unstable to prolonged storage even at low temperature and is recommended to be used in subsequent step immediately after preparation.

$R_f$ = 0.30 (35% ethyl acetate–hexanes; PAA). $^1$H NMR (500 MHz, Benzene-$d_6$): δ 5.04 (d, $J$ = 0.8 Hz, 1H), 4.76 (d, $J$ = 1.1 Hz, 1H), 3.19 (s, 2H), 2.10 – 2.02 (m, 4H), 1.97 (s, 2H), 1.50 – 1.46 (m, 2H), 1.41 – 1.38 (m, 2H), 0.99 (s, 9H). $^{13}$C NMR (126 MHz, Benzene-$d_6$): δ 209.2, 154.2, 110.1, 65.6, 38.0, 37.3, 37.2, 35.6, 32.7, 29.6. HRMS-CI (m/z): $[M + H]^+$ calcd for $C_{14}H_{25}O_2$, 225.1855; found, 225.1855.

**Synthesis of the doubly desaturated product 23.**

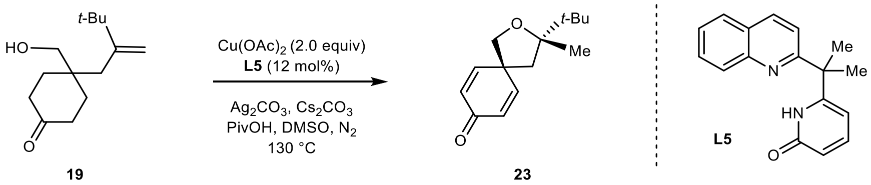


A screw-capped 16 x 125 mm culture tube was sequentially charged under air with the alkenol starting material **19** (22.4 mg, 0.10 mmol, 1 equiv), $Cu(OAc)_2$ (36.3 mg, 0.20 mmol, 2.00 equiv), ligand (**L5**) (3.17 mg, 12.0 µmol, 0.12 equiv), silver carbonate (27.6 mg, 0.10 mmol, 1.00 equiv), and cesium carbonate (97.7 mg, 0.30 mmol, 3.00 equiv). The reaction vessel was then sealed using a screw cap with a teflon coated septa. The reaction vessel was then placed under nitrogen atmosphere by using a vacuum manifold with a process of evacuating the headspace for 1.0 minute and venting with nitrogen for five times. Lastly, a pivalic acid (3.10 mg, 30.0 µmol, 0.30 equiv) dissolved in DMSO (1.0 mL) was added via a syringe to the reaction mixture at 23 °C. The reaction vessel was then placed into an oil bath that had been preheated to 130 °C. The reaction mixture was allowed to stir for 12 hours at 130 °C. After being allowed to cool to room temperature, the product mixture was diluted with ethyl acetate (10 mL) and water (10 mL). The resulting biphasic mixture was transferred to a separatory funnel and the layers that formed were separated. The aqueous layer was extracted with ethyl acetate (3 × 10 mL). The organic layers were combined, and the combined organic layers were washed with saturated aqueous sodium chloride solution (10 mL). The washed organic layer was dried over sodium sulfate. The dried solution was filtered and the filtrate was concentrated. The residue obtained was purified by flash-column chromatography (eluting with 15% ethyl acetate–hexanes) to provide the doubly desaturated ketone product **23** as a colorless oil (11.0 mg, 50%).

$R_f$ = 0.30 (10% ethyl acetate–hexanes; PAA). $^1$H NMR (500 MHz, $CDCl_3$): δ 7.05 (dd, $J$ = 9.9, 2.8 Hz, 1H), 6.98 (dd, $J$ = 9.9, 2.8 Hz, 1H), 6.30 – 6.21 (m, 2H), 3.94 (d, $J$ = 9.3 Hz, 1H), 3.86 (d, $J$ = 9.3 Hz, 1H), 2.31 (d, $J$ = 13.7 Hz, 1H), 1.77 (d, $J$ = 13.7 Hz, 1H), 1.35 (s, 3H), 1.01 (s, 9H).
$^{13}$C NMR (126 MHz, $CDCl_3$): δ 185.8, 152.5, 152.2, 128.5, 127.6, 89.9, 74.4, 49.8, 44.6, 38.0, 25.9, 22.5. HRMS-CI (m/z): $[M + H]^+$ calcd for $C_{14}H_{21}O_2$, 221.1542; found, 221.1543.

## List of Unsuccessful or Limited Reactivity Substrates. [a,b,c,d]

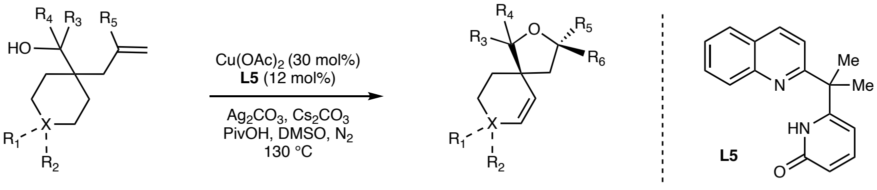


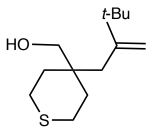

**S13**, n.d.

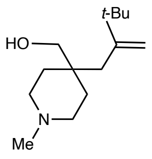

**S14**, n.d.

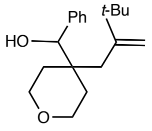

**S15**, 6%

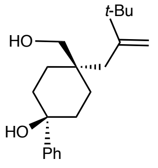

**S16**, 24%

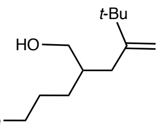

**S17**, 7%

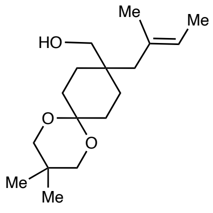

**S18**, n.d.

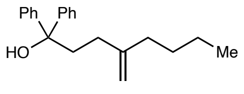

**S25**, n.d.[d]

[a]Conditions: substrate (0.1 mmol), $Cu(OAc)_2$ (30 mol%), **L5** (12 mol%), $Ag_2CO_3$ (2.0 equiv), $Cs_2CO_3$ (0.5 equiv), PivOH (30 mol%), DMSO (1.0 mL), $N_2$, 130 °C, 12h. [b]Yields were determined by $^1$H NMR analysis of an unpurified product mixture using $CH_2Br_2$ as an internal standard. [c]None detected. [d]We intended to trigger our transformation via an aryl migration pathway to form the desired carbon-centered radical but observed no productive reactivity with substrate **S25**.

## Reaction Optimization Tables.

Table S1. $Cu(OAc)_2$ and $Ag_2CO_3$ Equivalents Investigation.[a,b]

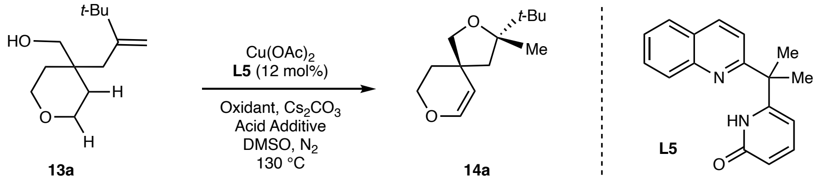


| Entry | $Cu(OAc)_2$ Loading | Oxidant | Yield of **14a** (%) |
|---|---|---|---|
| 1. | – | $Ag_2CO_3$ (2.0 equiv) | 0 |
| 2. | $Cu(OAc)_2$ (10 mol%) | $Ag_2CO_3$ (2.0 equiv) | 60 |
| 3. | $Cu(OAc)_2$ (20 mol%) | $Ag_2CO_3$ (2.0 equiv) | 70 |
| 4. | $Cu(OAc)_2$ (30 mol%) | $Ag_2CO_3$ (2.0 equiv) | 93 |
| 5. | $Cu(OAc)_2$ (1.0 equiv) | $Ag_2CO_3$ (1.0 equiv) | 90 |
| 6. | $Cu(OAc)_2$ (1.0 equiv) | – | 41 |
| 7. | $Cu(OAc)_2$ (2.0 equiv) | – | 66 |

[a]Reaction conditions: **13a** (0.10 mmol), **L5** (12 mol%), $Cs_2CO_3$ (0.5 equiv), DMSO (1.0 mL), $N_2$, 130 °C, 12h. [b]Yields were determined by $^1H$ NMR analysis using $CH_2Br_2$ as an internal standard

Table S2. Acid Additive Investigation.[a,b]

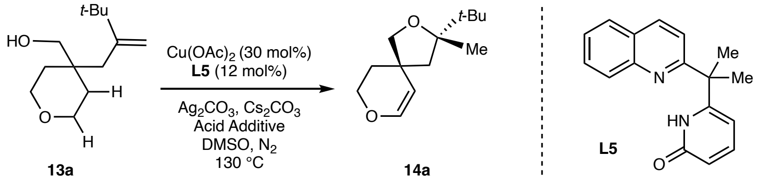


| Entry | Acid Additive | Yield of **14a** (%) |
|---|---|---|
| 1. | none | 80 |
| 2. | PivOH (30 mol%) | 93 |
| 3. | PivOH (50 mol%) | 84 |
| 4. | PivOH (1.0 equiv) | 75 |

[a]Reaction conditions: **13a** (0.10 mmol), $Cu(OAc)_2$ (30 mol%), **L5** (12 mol%), $Ag_2CO_3$ (2.0 equiv), $Cs_2CO_3$ (0.5 equiv), DMSO (1.0 mL), $N_2$, 130 °C, 12h. [b]Yields were determined by $^1H$ NMR analysis using $CH_2Br_2$ as an internal standard

## Synthetic Procedure for Preparation of 24, 25, and 27.

*Synthesis of the diol **24**:*

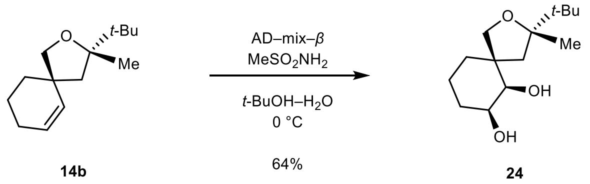


AD-mix-*β* (140 mg) was added to a mixture of the olefin **14b** (20.8 mg, 0.10 mmol, 1 equiv) and methanesulfonamide (19.0 mg, 0.20 mmol, 2.0 equiv) in tert-butanol–water (1:1, v/v, 1.0 mL) at 0 °C. The reaction mixture was stirred for 18 h at 0 °C. The product mixture was diluted with ethyl acetate (5.0 mL) and saturated aqueous sodium thiosulfate solution (5.0 mL). The diluted product mixture was stirred for 30 min at 23 °C. The resulting biphasic mixture was transferred to a separatory funnel, and the layers that formed were separated. The aqueous layer was extracted with ethyl acetate (10 mL). The organic layers were combined, and the combined organic layers were washed with saturated aqueous sodium chloride solution (10 mL). The washed organic layer was dried over sodium sulfate. The dried solution was filtered, and the filtrate was concentrated. The residue obtained was purified by flash column chromatography (eluting with 40% ethyl acetate–hexanes) to provide the diol **24** as a colorless oil (15.5 mg, 64%).

Note: No kinetic resolution was detected. The relative stereochemistry at the C5 and C6 position was established by NOE analysis. Correlations between the C4 methyl group and the hydrogen H5 support the relative assignment.

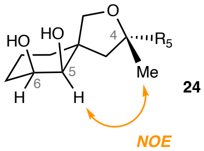


$R_f$ = 0.30 (50% ethyl acetate–hexanes; PAA). $^1$H NMR (400 MHz, $CDCl_3$) δ 3.96 (d, *J* = 9.4 Hz, 1H), 3.76 – 3.67 (m, 2H), 3.65 (d, *J* = 9.4 Hz, 1H), 1.80 (d, *J* = 13.1 Hz, 1H), 1.66 – 1.57 (m, 4H), 1.43 – 1.37 (m, 1H), 1.31 – 1.26 (m, 2H), 1.18 (s, 3H), 0.94 (s, 9H). $^{13}$C NMR (101 MHz, $CDCl_3$) δ 88.5, 77.4, 76.7, 70.5, 48.4, 42.0, 37.1, 29.9, 28.6, 25.9, 21.5, 19.8. HRMS-CI (m/z): [M + H]+ calcd for $C_{14}H_{27}O_3$, 243.1960; found, 243.1961.

*Synthesis of the aziridine **25**:*

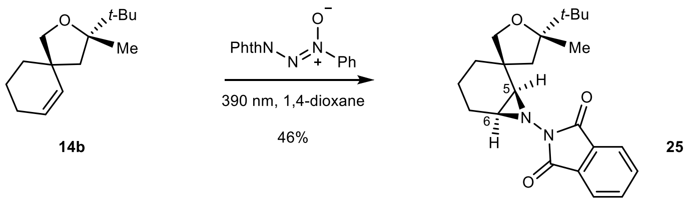

A 4-dram vial equipped with a stir-bar, was charged 1-phenyl-2-phthalimidodiazene 1-oxide[9] (66.8 mg, 0.25 mmol, 1.0 equiv.), the olefin **14b** (41.6 mg, 0.20 mmol, 2 equiv), and 1,4-dioxane (10.0 mL). The reaction vessel was then capped and left to stir at 1000 rpm and irradiated under 390 nm Kessil lamps with a cooling fan for 24 h. The product mixture was then concentrated, and the residue obtained was purified by flash column chromatography (eluting with 10% ethyl acetate–hexanes) to provide the aziridine **25** as a colorless oil (25.5 mg, 46%).

Note: The relative stereochemistry at the C5 and C6 position was established by NOE analysis. Correlations between the C1 methyl group and the hydrogen H5 support the relative assignment shown.

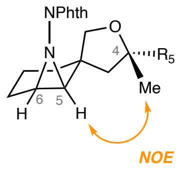

**25**

Rf = 0.40 (20% ethyl acetate–hexanes; PAA). $^1$H NMR (400 MHz, $CDCl_3$): δ 7.77 – 7.72 (m, 2H), 7.68 – 7.64 (m, 2H), 4.75 (d, J = 9.3 Hz, 1H), 3.69 (d, J = 9.3 Hz, 1H), 2.92 (d, J = 7.7 Hz, 1H), 2.73 – 2.67 (m, 1H), 2.34 – 2.23 (m, 1H), 1.98 (d, J = 13.2 Hz, 1H), 1.95 – 1.86 (m, 1H), 1.69 (d, J = 13.1 Hz, 1H), 1.62 – 1.59 (m, 1H), 1.43 – 1.32 (m, 5H), 1.19 – 1.12 (m, 1H), 0.98 (s, 9H). $^{13}$C NMR (101 MHz, $CDCl_3$): δ 165.4, 134.1, 130.6, 123.0, 88.5, 75.5, 51.6, 47.7, 44.9, 42.2, 37.3, 33.6, 25.9, 22.7, 21.8, 17.7. HRMS-CI (m/z): [M + H]+ calcd for $C_{22}H_{29}N_2O_3$ for 369.2178; found, 369.2182.

*Installation of the directing moiety to provide olefin **S17**:*

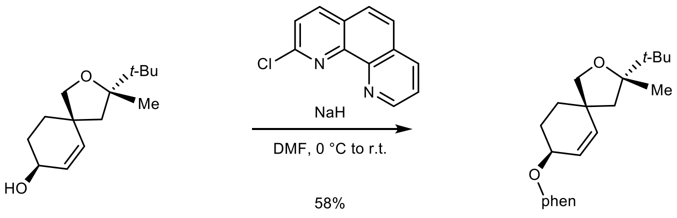


Sodium hydride (60% dispersion in mineral oil, 82.0 mg, 2.05 mmol, 2.00 equiv) was added in three equal portions to a solution of the allylic alcohol **14g** (230 mg, 1.03 mmol, 1 equiv) in dimethylformamide (2.0 mL) at 0 °C. The reaction mixture was allowed to slowly warm to 23 °C and stirred for 12 hours at 23 °C. The product mixture was sequentially diluted sequentially with ethyl acetate (10 mL) and water (10 mL). The resulting biphasic mixture was transferred to a separatory funnel and the layers that formed were separated. The aqueous layer was extracted with ethyl acetate (3 × 10 mL). The organic layers were combined, and the combined organic layers were washed with saturated aqueous sodium chloride solution (10 mL). The washed organic layer was dried over sodium sulfate. The dried solution was filtered and the filtrate was concentrated.

The residue obtained was purified by flash-column chromatography (eluting with 20% ethyl acetate–hexanes) to provide the tricyclic product **26** as a colorless oil (239 mg, 58%).

$R_f$ = 0.50 (35% ethyl acetate–hexanes; PAA). $^1$H NMR (400 MHz, $CDCl_3$): δ 9.16 (dd, *J* = 4.3, 1.8 Hz, 1H), 8.23 (dd, *J* = 8.1, 1.8 Hz, 1H), 8.10 (d, *J* = 8.6 Hz, 1H), 7.74 (d, *J* = 8.7 Hz, 1H), 7.65 (d, *J* = 8.7 Hz, 1H), 7.58 (dd, *J* = 8.1, 4.3 Hz, 1H), 7.09 (d, *J* = 8.6 Hz, 1H), 6.31 – 6.25 (m, 1H), 6.03 – 5.94 (m, 2H), 3.74 (s, 2H), 2.30 – 2.22 (m, 1H), 2.03 – 1.93 (m, 3H), 1.75 – 1.69 (m, 1H), 1.64 (d, *J* = 13.1 Hz, 1H), 1.25 (s, 3H), 0.97 (s, 9H). $^{13}$C NMR (101 MHz, $CDCl_3$): δ 162.4, 150.0, 145.4, 144.5, 139.1, 138.6, 136.4, 129.3, 126.6, 126.5, 124.9, 123.7, 122.5, 114.7, 88.8, 77.0, 69.0, 47.8, 44.8, 37.5, 31.9, 27.2, 25.9, 22.3. HRMS-CI (m/z): $[M + H]^+$ calcd for $C_{26}H_{31}N_2O_2$, 403.2386; found, 403.2394.

*Synthesis of the C–H alkynylation product **27**:*

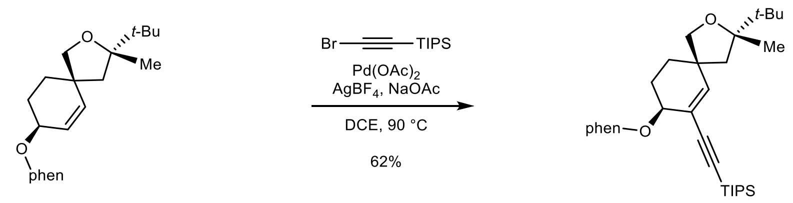


**26** **27**

A screw-capped 16 x 125 mm culture tube was sequentially charged under air with the olefin **26** (40.2 mg, 0.10 mmol, 1 equiv), $Pd(OAc)_2$ (2.25 mg, 10.0 µmol, 0.10 equiv), sodium acetate (8.20 mg, 0.10 mmol, 1.00 equiv), silver tetrafluorborate (19.5 mg, 0.10 mmol, 1.00 equiv), (bromoethynyl)triisopropylsilane (47.0 µL, 0.20 mmol, 2.00 equiv), and dichloroethane (1.0 mL). the reaction vessel was sealed and placed into an oil bath that had been preheated to 90 °C. The reaction mixture was allowed to stir for 16 hours at 90 °C. After being allowed to cool to room temperature, the product mixture was diluted with dichloroethane (10 mL) and water (10 mL). The resulting biphasic mixture was transferred to a separatory funnel and the layers that formed were separated. The aqueous layer was extracted with dichloroethane (3 × 10 mL). The organic layers were combined, and the combined organic layers were washed with saturated aqueous sodium chloride solution (10 mL). The washed organic layer was dried over sodium sulfate. The dried solution was filtered and the filtrate was concentrated. The residue obtained was purified by flash-column chromatography (eluting with 10% ethyl acetate–hexanes) to provide the C–H alkynylation product **27** as a colorless oil (36.0 mg, 62%).

$R_f$ = 0.30 (20% ethyl acetate–hexanes; PAA). $^1$H NMR (400 MHz, $CDCl_3$) δ 9.19 (s, 1H), 8.38 – 8.05 (m, 2H), 7.79 – 7.56 (m, 3H), 7.14 (d, *J* = 8.8 Hz, 1H), 6.54 (s, 1H), 6.36 (s, 1H), 3.80 – 3.73 (m, 2H), 2.37 (s, 1H), 2.06 – 1.93 (m, 3H), 1.84 – 1.68 (m, 2H), 1.26 (s, 3H), 0.97 (s, 9H), 0.81 – 0.78 (m, 21H). $^{13}$C NMR (101 MHz, $CDCl_3$) δ 162.7, 149.8, 149.5, 145.6, 139.2, 139.0, 129.3, 127.2, 126.6, 125.2, 123.3, 122.5, 122.1, 106.1, 90.1, 89.0, 77.4, 76.7, 47.6, 45.6, 37.5, 31.2, 26.9, 25.9, 22.3, 18.6, 11.2. HRMS-CI (m/z): $[M + H]^+$ calcd for $C_{37}H_{51}N_2O_2Si$, 583.3720; found, 583.3718.

**Synthesis of 36 Possessing the All-carbon Framework of Spirotenuipesines A (3).**

*Synthesis of the allylic alcohol **29**:*

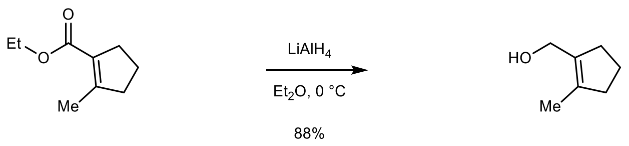


28 29

Lithium aluminum hydride (365 mg, 9.63 mmol, 1.10 equiv) was added in three equal portions to a solution of the ester **28**[10] (1.35 g, 8.750 mmol, 1 equiv) in diethyl ether (44 mL) at 0 °C. The reaction mixture was allowed to slowly warm to 23 °C overnight. The reaction mixture was stirred at 0 °C for 2 hours. The cold product mixture was sequentially diluted sequentially with saturated aqueous potassium sodium tartrate tetrahydrate (40 mL) and diethyl ether (40 mL) and allowed to stir at 23 °C for 2 hours. The resulting biphasic mixture was then transferred to a separatory funnel and the layers that formed were separated. The aqueous layer was extracted with ethyl acetate (3 × 40 mL). The organic layers were combined, and the combined organic layers were washed with saturated aqueous sodium chloride solution (40 mL). The washed organic layer was dried over sodium sulfate. The dried solution was filtered, and the filtrate was concentrated. The residue obtained was purified by flash column chromatography (eluting with 20% ethyl acetate–hexanes) to provide the allylic alcohol **29** as a colorless oil (860 mg, 88%).

The spectroscopic data for the corresponding allylic alcohol **29** prepared according to this general procedure matched the previously reported $^{1}$H NMR data.[11]

*Synthesis of the ester **31**:*

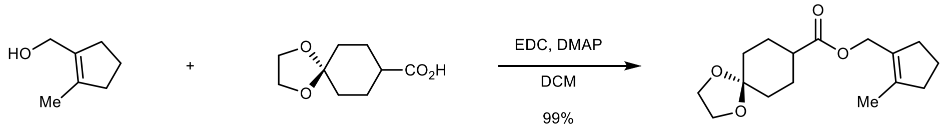


(4-Dimethylamino)pyridine (DMAP, 1.58 g, 12.9 mmol, 2.00 equiv), allylic alcohol **29** (795 mg, 7.09 mmol, 1.10 equiv), and EDC (2.47g, 12.9 mmol, 2.00 equiv) were added in sequence to a solution of the carboxylic acid **30**[12] (1.20 g, 6.44 mmol, 1 equiv) in dichloromethane (33 mL) at 23 °C. The reaction mixture was stirred for at 23 °C for 12 h. The product mixture was then diluted sequentially with dichloromethane (40 mL), water (40 mL), and saturated aqueous ammonium chloride solution (40 mL). The resulting biphasic mixture was transferred to a separatory funnel and the layers that formed were separated. The aqueous layer was extracted with dichloromethane (3 × 40 mL). The organic layers were combined and the combined organic layers were washed with saturated aqueous sodium chloride solution (40 mL). The washed organic layer was dried over sodium sulfate. The dried solution was filtered, and the filtrate was concentrated. The residue obtained was purified by flash-column chromatography (eluting with 5% ethyl acetate–hexanes) to provide the ester **31** as a colorless oil (1.79 g, 99%).

$R_f$ = 0.30 (10% ethyl acetate–hexanes; PAA). $^1$H NMR (400 MHz, $CDCl_3$): δ 4.63 (s, 2H), 3.94 (s, 4H), 2.42 – 2.17 (m, 5H), 1.99 – 1.89 (m, 2H), 1.86 – 1.74 (m, 6H), 1.69 (s, 3H), 1.58 – 1.50 (m, 2H). $^{13}$C NMR (100 MHz, $CDCl_3$): δ 175.2, 138.4, 129.8, 108.1, 64.3, 61.0, 41.7, 38.7, 34.4, 33.8, 26.3, 21.5, 13.9. HRMS-CI (m/z): $[M + H]^+$ calcd $C_{16}H_{25}O_4$ for 281.1753; found, 281.1757.

*Synthesis of the carboxylic acid **32**:*

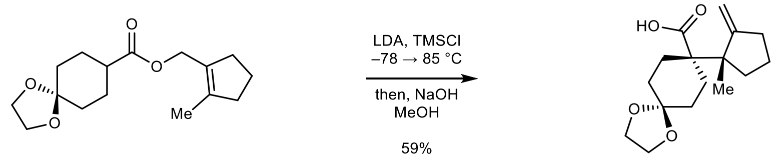


**31** **32**

In a round-bottomed flask fused to a Teflon-coated valve, a solution of n-butyllithium in hexanes (2.50 M, 4.28 mL, 10.7 mmol, 2.00 equiv.) was added dropwise via syringe over 30 min to a solution of diisopropylamine (1.55 mL, 11.0 mmol, 2.05 equiv.) in tetrahydrofuran (25 mL) at –78 °C. The resulting solution was stirred for 45 min at –78 °C. A solution of the ester **31** (1.50 g, 5.35 mmol, 1.0 equiv.) in tetrahydrofuran (5.0 mL) was then added dropwise via syringe over 15 min at –78 °C. Upon completion of the addition, the reaction mixture was stirred for 1 hour at –78 °C. Trimethylsilyl chloride (1.43 mL, 11.2 mmol, 2.10 equiv) was then added dropwise via syringe over 15 min at –78 °C. The reaction mixture was stirred 1 hour at –78 °C. The reaction mixture was then allowed to gradually warm up to 23 °C over the period of 1 hour. The reaction vessel was subsequently sealed with a Teflon-coated valve and placed into an oil bath that had been preheated to 85 °C. The reaction mixture was allowed to stir for 12 hours at 85 °C. After being allowed to cool to room temperature, a solution of aqueous sodium hydroxide (2 M, 5.35 mL, 21.4 mmol, 4 equiv) and methanol (5.35 mL) was then added, and the reaction mixture was stirred for 2 hours at 23 °C. The product mixture was then diluted sequentially with diethyl ether (40 mL) and hydrochloric acid 1N until the pH ~ 4. The resulting biphasic mixture was transferred to a separatory funnel and the layers that formed were separated. The aqueous layer was extracted with diethyl ether (3 × 25 mL). The organic layers were combined and the combined organic layers were washed with saturated aqueous sodium chloride solution (30mL). The washed organic layer was dried over sodium sulfate. The dried solution was filtered, and the filtrate was concentrated. The residue obtained was purified by flash-column chromatography (eluting with 20% ethyl acetate–hexanes) to provide the carboxylic acid **32** as a colorless oil (890 mg, 59%).

$R_f$ = 0.30 (40% ethyl acetate–hexanes; PAA). $^1$H NMR (400 MHz, Benzene-$d_6$): δ 5.06 (t, $J$ = 1.7 Hz, 1H), 4.94 (d, $J$ = 2.4 Hz, 1H), 3.54 – 3.49 (m, 2H), 3.48 – 3.43 (m, 2H), 2.36 – 2.12 (m, 5H), 2.06 – 1.86 (m, 3H), 1.85 – 1.70 (m, 3H), 1.51 – 1.46 (m, 1H), 1.37 – 1.28 (m, 2H), 1.23 (s, 3H). $^{13}$C NMR (101 MHz, Benzene-$d_6$): δ 180.3, 157.8, 108.6, 108.4, 64.3, 64.2, 53.1, 49.4, 38.5, 38.0, 33.3, 33.1, 27.6, 27.0, 25.3, 23.5. HRMS-CI (m/z): $[M + H]^+$ calcd $C_{16}H_{25}O_4$ for 281.1753; found, 281.1743.

*Synthesis of the primary alcohol **33**:*

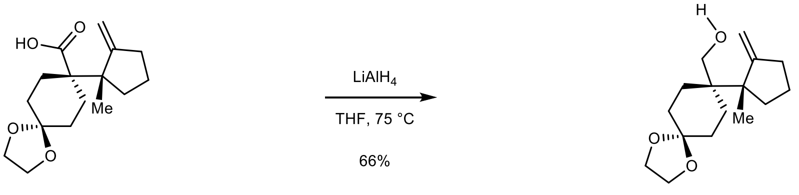


In a round-bottomed flask fused to a Teflon-coated valve, lithium aluminum hydride (111 mg, 2.78 mmol, 1.30 equiv) was added in three equal portions to a solution of the carboxylic acid **32** (600 mg, 2.14 mmol, 1 equiv) in tetrahydrofuran (20 mL) at 0 °C. The reaction mixture was allowed to warm to 23 °C. The reaction vessel was subsequently sealed with a Teflon-coated valve and placed into an oil bath that had been preheated to 75 °C. The reaction mixture was stirred at 75 °C for 12 hours. After being allowed to cool to room temperature, the product mixture was sequentially diluted sequentially with saturated aqueous potassium sodium tartrate tetrahydrate (20 mL) and diethyl ether (20 mL) and allowed to stir at 23 °C for 2 hours. The resulting biphasic mixture was then transferred to a separatory funnel and the layers that formed were separated. The aqueous layer was extracted with diethyl ether (3 × 20 mL). The organic layers were combined, and the combined organic layers were washed with saturated aqueous sodium chloride solution (30 mL). The washed organic layer was dried over sodium sulfate. The dried solution was filtered, and the filtrate was concentrated. The residue obtained was purified by flash column chromatography (eluting with 30% ethyl acetate–hexanes) to provide the allylic alcohol **33** as a colorless oil (379 mg, 66%).

$R_f$ = 0.50 (40% ethyl acetate–hexanes; PAA). $^{1}$H NMR (400 MHz, Benzene-$d_6$): δ 4.98 (d, $J$ = 1.6 Hz, 1H), 4.89 – 4.87 (m, 1H), 3.62 – 3.49 (m, 6H), 2.25 – 2.16 (m, 2H), 2.07 – 1.94 (m, 1H), 1.92 – 1.75 (m, 5H), 1.70 – 1.59 (m, 2H), 1.56 – 1.47 (m, 1H), 1.43 – 1.36 (m, 1H), 1.35 – 1.24 (m, 2H), 1.12 (s, 3H). $^{13}$C NMR (101 MHz, Benzene-$d_6$): δ 161.2, 109.0, 107.8, 64.3, 64.2, 62.1, 50.6, 41.8, 38.9, 37.8, 31.5, 31.5, 25.7, 25.5, 25.2, 23.4. HRMS-CI (m/z): [M + H]$^+$ calcd $C_{16}H_{27}O_3$ for 267.1960; found, 267.1960.

*Synthesis of the desaturated oxabicycle **35**:*

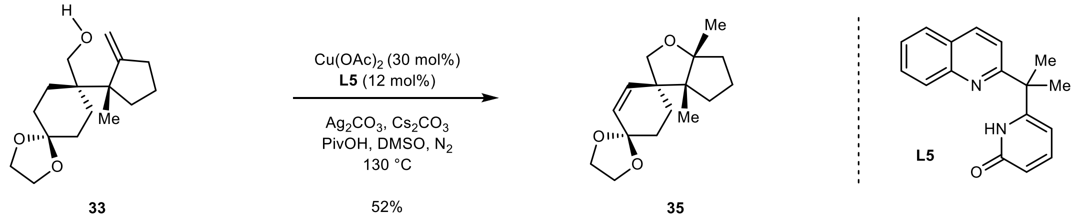

A round bottom-flask was sequentially charged with the primary alcohol **33** (319 mg, 1.20 mmol, 1 equiv), $Cu(OAc)_2$ (65.4 mg, 0.360 mmol, 0.30 equiv), ligand (**L5**) (38.0 mg, 144 µmol, 0.12 equiv), silver carbonate (662 mg, 2.40 mmol, 2.00 equiv), and cesium carbonate (195 mg, 600 µmol, 0.50 equiv). The reaction vessel was then sealed using a white rubber septa. The reaction vessel was then placed under nitrogen atmosphere by using a vacuum manifold with a process of evacuating the headspace for 1.0 minute and venting with nitrogen for five times. Lastly, a pivalic acid (36.8 mg, 360 µmol, 0.30 equiv) dissolved in DMSO (12 mL) was added via a syringe to the reaction mixture at 23 °C. The reaction vessel was then placed into an oil bath that had been preheated to 130 °C. The reaction mixture was allowed to stir for 12 hours at 130 °C. After being allowed to cool to room temperature, the product mixture was diluted with ethyl acetate (30 mL) and water (30 mL). The resulting biphasic mixture was transferred to a separatory funnel and the layers that formed were separated. The aqueous layer was extracted with ethyl acetate (3 × 20 mL). The organic layers were combined, and the combined organic layers were washed with saturated aqueous sodium chloride solution (30 mL). The washed organic layer was dried over sodium sulfate. The dried solution was filtered and the filtrate was concentrated. The residue obtained was purified by flash-column chromatography (eluting with 20% ethyl acetate–hexanes) to provide the desaturated oxabicycle **35** as a colorless oil (165 mg, 52%).

$R_f$ = 0.50 (20% ethyl acetate–hexanes; PAA). $^1$H NMR (600 MHz, Benzene-$d_6$): δ 6.04 (dd, $J$ = 10.4, 1.3 Hz, 1H), 5.74 (dd, $J$ = 10.4, 1.2 Hz, 1H), 3.69 (d, $J$ = 8.6 Hz, 1H), 3.59 – 3.56 (m, 3H), 3.53 – 3.47 (m, 1H), 3.41 (d, $J$ = 8.6 Hz, 1H), 1.91 – 1.87 (m, 1H), 1.86 – 1.80 (m, 2H), 1.74 (ddd, $J$ = 12.7, 11.6, 3.3 Hz, 1H), 1.65 – 1.61 (m, 1H), 1.61 – 1.57 (m, 1H), 1.50 – 1.39 (m, 3H), 1.22 (s, 3H), 1.03 (dddd, $J$ = 12.6, 5.7, 3.4, 1.8 Hz, 1H), 0.84 (s, 3H). $^{13}$C NMR (151 MHz, Benzene-$d_6$): δ 137.1, 127.4, 105.5, 92.4, 74.9, 64.5, 64.3, 55.1, 49.8, 43.6, 37.8, 32.6, 25.5, 25.4, 24.0, 19.6. HRMS-CI (m/z): $[M + H]^+$ calcd $C_{16}H_{25}O_3$ for 265.1804; found, 265.1803.

*Synthesis of the α,β-unsaturated ketone **S19**:*

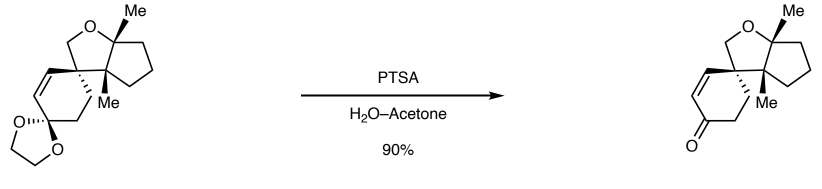


*p*-Toluenesulfonic acid (PTSA, 7.60 mg, 39.7 μmol, 0.30 equiv) was added to a solution of the desaturated oxabicycle **35** (35.0 mg, 132 μmol, 1 equiv) in acetone–water (10:1 v/v; 1.5 mL) at 23 °C. The reaction mixture was stirred for at 23 °C for 6 h. The product mixture was then diluted sequentially with ethyl acetate (5 mL), water (5 mL). The resulting biphasic mixture was transferred to a separatory funnel and the layers that formed were separated. The aqueous layer was extracted with ethyl acetate (3 × 5 mL). The organic layers were combined and the combined organic layers were washed with saturated aqueous sodium chloride solution (10 mL). The washed organic layer was dried over sodium sulfate. The dried solution was filtered, and the filtrate was concentrated. The residue obtained was purified by flash-column chromatography (eluting with 10% ethyl acetate–hexanes initially) to provide the *α,β*-unsaturated ketone **S19** as a colorless oil (26.2 mg, 90%).

$R_f$ = 0.30 (20% ethyl acetate–hexanes; PAA). $^1$H NMR (400 MHz, $CDCl_3$): δ 6.98 (dd, *J* = 10.4, 0.9 Hz, 1H), 6.02 (d, *J* = 10.4 Hz, 1H), 3.77 – 3.67 (m, 2H), 2.59 – 2.40 (m, 1H), 2.36 (ddd, *J* = 17.2, 8.5, 5.3 Hz, 1H), 2.04 (dddd, *J* = 13.5, 8.4, 5.0, 0.9 Hz, 1H), 1.96 – 1.77 (m, 4H), 1.75 – 1.66 (m, 1H), 1.55 – 1.37 (m, 2H), 1.30 (s, 3H), 0.98 (s, 3H). $^{13}$C NMR (101 MHz, $CDCl_3$): δ 198.9, 155.3, 128.2, 93.3, 73.9, 55.2, 50.2, 43.0, 38.0, 35.2, 26.3, 25.3, 23.9, 20.4. HRMS-CI (m/z): $[M + H]^+$ calcd $C_{14}H_{21}O_2$ for 221.1542; found, 221.1542.

*Synthesis of the tertiary alcohol **36**:*

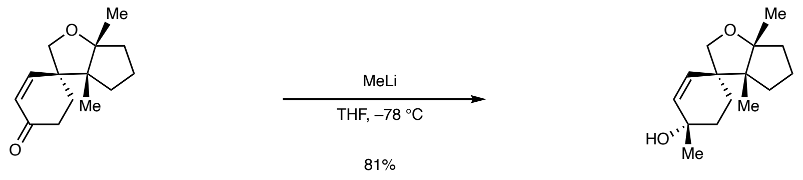


**S19** **36**

Methyl lithium (1.00 M, 136 µL, 136 µmol, 2.00 equiv) was added dropwise via a syringe to a solution of the α,β-unsaturated ketone **S19** (15.0 mg, 68.0 µmol, 1 equiv) in tetrahydrofuran (680 µL) at –78 °C. The reaction mixture was stirred for at –78 °C for 3 h. The product mixture was then allowed to gradually warm up to 23 °C over 30 minutes and was subsequently diluted sequentially with ethyl acetate (5 mL), water (5 mL). The resulting biphasic mixture was transferred to a separatory funnel and the layers that formed were separated. The aqueous layer was extracted with ethyl acetate (3 × 5 mL). The organic layers were combined and the combined organic layers were washed with saturated aqueous sodium chloride solution (10 mL). The washed organic layer was dried over sodium sulfate. The dried solution was filtered, and the filtrate was concentrated. The residue obtained was purified by flash-column chromatography (eluting with 30% ethyl acetate–hexanes initially) to provide the tertiary alcohol **36** as a colorless oil (13.0 mg, 81%).

$R_f$ = 0.30 (50% ethyl acetate–hexanes; PAA). $^1$H NMR (500 MHz, Benzene-$d_6$) δ 5.75 (d, $J$ = 10.3 Hz, 1H), 5.51 (d, $J$ = 10.3 Hz, 1H), 3.62 (d, $J$ = 8.6 Hz, 1H), 3.43 (d, $J$ = 8.6 Hz, 1H), 1.89 – 1.83 (m, 1H), 1.68 – 1.57 (m, 3H), 1.48 – 1.41 (m, 2H), 1.38 – 1.30 (m, 3H), 1.23 (s, 3H), 1.17 (s, 3H), 1.04 – 1.00 (m, 1H), 0.72 (s, 3H). $^{13}$C NMR (126 MHz, Benzene-$d_6$) δ 133.9, 133.0, 92.7, 75.6, 68.1, 54.9, 49.6, 43.6, 37.9, 36.8, 28.7, 25.5, 25.1, 24.2, 20.1. HRMS-CI (m/z): $[M + H]^+$ calcd $C_{15}H_{25}O_2$ for 237.1855; found, 237.1848.

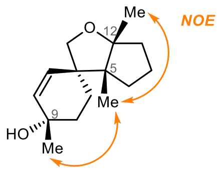


**36**

Note: The relative stereochemistry at the C9 position was established by NOE analysis. Correlations between the methyl group at C12 and the methyl group at C5 as well as correlations between the methyl group at C5 and the methyl group at C9 support the relative assignment shown.

**Radical Trapping Experiments with TEMPO:**

*Synthesis of the TEMPO adduct **37**:*

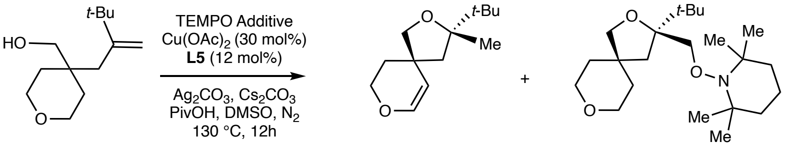


**13a**

| | **14a** | **37** |
|---|---|---|
| 1) TEMPO (0.5 equiv): | 42% | 48% |
| 2) TEMPO (1.0 equiv): | 2% | 91% |

A screw-capped 16 x 125 mm culture tube was sequentially charged under air with the alkenol starting material **13a** (21.2 mg, 0.10 mmol, 1 equiv), $Cu(OAc)_2$ (5.45 mg, 30.0 µmol, 0.30 equiv), ligand (**L5**) (3.17 mg, 12.0 µmol, 0.12 equiv), silver carbonate (55.2 mg, 0.20 mmol, 2.00 equiv), TEMPO (7.81 mg, 50.0 µmol, 0.5 equiv or 15.6 mg, 0.10 mmol, 1.00 equiv) and cesium carbonate (16.3 mg, 50.0 µmol, 0.50 equiv). The reaction vessel was then sealed using a screw cap with a teflon coated septa. The reaction vessel was then placed under nitrogen atmosphere by using a vacuum manifold with a process of evacuating the headspace for 1.0 minute and venting with nitrogen for five times. Lastly, a pivalic acid (3.10 mg, 30.0 µmol, 0.30 equiv) dissolved in DMSO (1.0 mL) was added via a syringe to the reaction mixture at 23 °C. The reaction vessel was then placed into an oil bath that had been preheated to 130 °C. The reaction mixture was allowed to stir for 12 hours at 130 °C. After being allowed to cool to room temperature, the product mixture was diluted with ethyl acetate (10 mL) and water (10 mL). The resulting biphasic mixture was transferred to a separatory funnel and the layers that formed were separated. The aqueous layer was extracted with ethyl acetate (3 × 10 mL). The organic layers were combined, and the combined organic layers were washed with saturated aqueous sodium chloride solution (10 mL). The washed organic layer was dried over sodium sulfate. The dried solution was filtered and the filtrate was concentrated. The residue obtained was purified by flash-column or preparative thin-layer chromatography to provide the TEMPO adduct **37** (17.6 mg, 48% with 0.5 equiv of TEMPO) or **37** (33.4 mg, 91% with 1.0 equiv of TEMPO).

$^{1}H$ NMR analysis of the unpurified reaction mixture with dibromomethane indicated the presence of 42% of **14a** with 0.5 equiv of TEMPO and 2% of **14a** with 1.0 equiv of TEMPO.

$R_f$ = 0.40 (10% ethyl acetate–hexanes; PAA). $^{1}H$ NMR (400 MHz, $CDCl_3$): δ 3.90 (d, $J$ = 14.8 Hz, 3H), 3.75 (d, $J$ = 8.3 Hz, 1H), 3.72 – 3.63 (m, 3H), 3.58 (ddd, $J$ = 11.4, 7.1, 3.6 Hz, 1H), 1.99 (d, $J$ = 13.2 Hz, 1H), 1.85 (ddd, $J$ = 13.3, 7.1, 3.6 Hz, 1H), 1.79 – 1.68 (m, 2H), 1.63 (t, $J$ = 5.3 Hz, 2H), 1.59 – 1.40 (m, 5H), 1.32 (dddd, $J$ = 9.5, 6.5, 4.7, 3.1 Hz, 1H), 1.22 (s, 3H), 1.16 (s, 3H), 1.11 (s, 6H), 0.93 (s, 9H). $^{13}C$ NMR (101 MHz, $CDCl_3$): δ 90.0, 81.4, 78.7, 66.1, 65.7, 59.9, 59.8, 41.9, 39.9, 39.9, 39.8, 37.5, 36.9, 36.3, 33.1, 32.9, 26.1, 21.2, 21.1, 17.0. HRMS-CI (m/z): $[M + H]^+$ calcd $C_{22}H_{42}NO_3$ for 368.3165; found, 368.3175.

**Synthesis of the Radical Clock Substrate 43.**

*Synthesis of the ketone **S21**:*

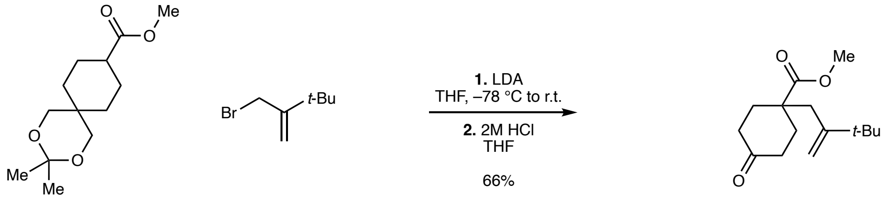


**S20** **S2** **S21**

A solution of *n*-butyllithium in hexanes (2.50 M, 3.96 mL, 9.91 mmol, 1.20 equiv) was added dropwise via syringe over 30 min to a solution of diisopropylamine (1.46 mL, 10.3 mmol, 1.25 equiv) in tetrahydrofuran (42 mL) at –78 °C. The resulting solution was stirred for 45 min at –78 °C. A solution of the ester **S20** (2.00 g, 8.26 mmol, 1.0 equiv.) in tetrahydrofuran was then added dropwise via syringe over 15 min at –78 °C. Upon completion of the addition, the reaction mixture was stirred for 1 hour at –78 °C. 2-(bromomethyl)-3,3-dimethylbut-1-ene (**S2**)[1] (1.74 mL, 9.91 mmol, 1.2 equiv.) was then added dropwise via syringe at –78 °C. The reaction mixture was allowed to slowly warm to 23 °C overnight. The warmed product mixture was diluted sequentially with water (40 mL) and ethyl acetate (40 mL). The resulting biphasic mixture was transferred to a separatory funnel and the layers that formed were separated. The aqueous layer was extracted with ethyl acetate (3 × 30 mL). The organic layers were combined, and the combined organic layers were washed with saturated aqueous sodium chloride solution. The washed organic layer was dried over sodium sulfate. The dried solution was filtered and the filtrate was concentrated. To the residue obtained was added tetrahydrofuran (30 mL) and hydrochloric acid (2.0 M, 41.3 mL, 82.6 mmol, 10.0 equiv.) at 23 °C and reaction mixture was stirred at this temperature for 4 hours. The product mixture was subsequently diluted sequentially with water (40 mL) and ethyl acetate (40 mL). The resulting biphasic mixture was transferred to a separatory funnel and the layers that formed were separated. The aqueous layer was extracted with ethyl acetate (3 × 30 mL). The organic layers were combined, and the combined organic layers were washed with saturated aqueous sodium chloride solution. The washed organic layer was dried over sodium sulfate. The dried solution was filtered, and the filtrate was concentrated. The residue obtained containing the ketone **S21** was used in the following step without further purification.

*Synthesis of the methyl ether* ***S22****:*

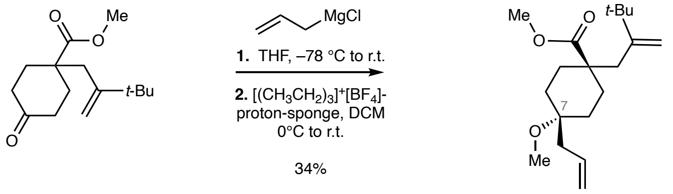


**S21** **S22**

Allylmagnesium chloride (2.00 M, 6.60 mL, 13.2 mmol, 1.60 equiv) was added to the residue obtained in the previous step (nominally, 8.26 mmol, 1.0 equiv) in tetrahydrofuran (42 mL) at –78 °C. The reaction was stirred at –78 °C for 1 hour and was subsequently allowed to warm to 23 °C over the period of 2 hours. The warmed product mixture was diluted sequentially with water (40 mL) and ethyl acetate (40 mL). The resulting biphasic mixture was transferred to a separatory funnel and the layers that formed were separated. The aqueous layer was extracted with ethyl acetate (3 × 30 mL). The organic layers were combined, and the combined organic layers were washed with saturated aqueous sodium chloride solution. The washed organic layer was dried over sodium sulfate. The dried solution was filtered and the filtrate was concentrated. The residue obtained was then dissolved dichloromethane (41 mL) and trimethyloxonium tetrafluoroborate (1.95 g, 13.2 mmol, 1.6 equiv.) and proton-sponge (3.00 g, 14.0 mmol, 1.7 equiv.) were added to the reaction mixture at 0°C. The reaction mixture was gradually allowed to warm up to 23 °C over the period of 2 hours and was subsequently stirred at 23 °C for 15 hours. The product mixture was subsequently diluted sequentially with water (40 mL) and dichloromethane (40 mL). The resulting biphasic mixture was transferred to a separatory funnel and the layers that formed were separated. The aqueous layer was extracted with dichloromethane (3 × 30 mL). The organic layers were combined, and the combined organic layers were washed with saturated aqueous sodium chloride solution. The washed organic layer was dried over sodium sulfate. The dried solution was filtered, and the filtrate was concentrated. The residue obtained was purified by flash-column chromatography (eluting with 10% ethyl acetate–hexanes initially) to provide the methyl ether **S22** as a colorless oil (860 mg, 34%). The relative stereochemistry at C7 was determined after the subsequent transformation.

$R_f$ = 0.40 (10% ethyl acetate–hexanes; PAA). $^1$H NMR (500 MHz, $CDCl_3$): δ 5.78 (ddt, $J$ = 17.4, 10.3, 7.2 Hz, 1H), 5.10 – 4.99 (m, 2H), 4.91 (s, 1H), 4.62 (d, $J$ = 1.4 Hz, 1H), 3.65 (s, 3H), 3.16 (s, 3H), 2.31 (s, 2H), 2.17 (dd, $J$ = 7.2, 1.3 Hz, 2H), 2.08 – 1.99 (m, 2H), 1.76 – 1.68 (m, 2H), 1.49 (td, $J$ = 13.3, 3.2 Hz, 2H), 1.38 (td, $J$ = 13.8, 3.4 Hz, 2H), 1.01 (s, 9H). $^{13}$C NMR (126 MHz, $CDCl_3$): δ 177.3, 153.2, 133.6, 117.4, 107.6, 74.1, 51.4, 48.3, 45.9, 42.0, 41.3, 36.5, 30.6, 30.4, 29.2. HRMS-CI (m/z): $[M + H]^+$ calcd $C_{19}H_{33}O_3$ for 309.2430; found, 309.2428.

*Synthesis of the primary alcohol **38**:*

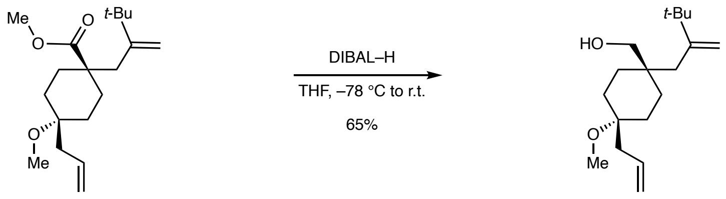


**S22** **38**

A solution of diisobutylaluminium hydride in toluene (1.00 M, 7.83 mL, 7.83 mmol, 3.00 equiv) was added dropwise via syringe to a solution of the methyl ether **S22** (800 mg, 2.61 mmol, 1 equiv) in tetrahydrofuran (13 mL) at –78 °C. The reaction mixture was allowed to slowly warm to 23 °C overnight. The warmed product mixture was diluted sequentially saturated aqueous potassium sodium tartrate tetrahydrate solution (10 mL) and ethyl acetate (10 mL). The resulting biphasic mixture was allowed to stir for 1 hour at 23 °C. The product mixture was then transferred to a separatory funnel and the layers that formed were separated. The aqueous layer was extracted with ethyl acetate (3 × 10 mL). The organic layers were combined, and the combined organic layers were washed with saturated aqueous sodium chloride solution. The washed organic layer was dried over sodium sulfate. The dried solution was filtered and the filtrate was concentrated. The residue obtained was purified by flash-column chromatography (eluting with 10% ethyl acetate–hexanes initially) to provide the alcohol **38** as a colorless oil (542 mg, 65%).

Note: The relative stereochemistry at the C7 position was established by NOE analysis. Correlations between the C1 hydrogen and the allylic substituent (C9) support the relative assignment shown.

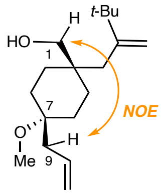


$R_f$ = 0.50 (20% ethyl acetate–hexanes; PAA). $^1$H NMR (400 MHz, Benzene-$d_6$): δ 5.84 (ddt, $J$ = 17.3, 10.2, 7.2 Hz, 1H), 5.25 – 4.90 (m, 4H), 3.45 (s, 2H), 2.97 (s, 3H), 2.14 (s, 2H), 2.05 (d, $J$ = 7.2 Hz, 2H), 1.68 (td, $J$ = 13.2, 4.0 Hz, 2H), 1.64 – 1.56 (m, 2H), 1.51 – 1.41 (m, 2H), 1.26 – 1.15 (m, 2H), 1.07 (s, 9H). $^{13}$C NMR (101 MHz, Benzene-$d_6$): δ 154.9, 134.6, 117.0, 110.0, 74.1, 64.1, 47.9, 41.6, 39.5, 38.0, 37.1, 29.7, 29.6, 28.8. HRMS-CI (m/z): [M + H]$^+$ calcd $C_{18}H_{33}O_2$ for 281.2481; found, 281.2485.

**Radical Clock Experiment with Substrate 38.**

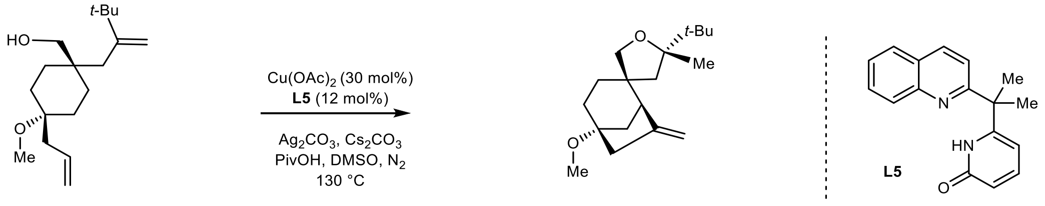


A screw-capped 16 x 125 mm culture tube was sequentially charged under air with the alkenol starting material **38** (27.8 mg, 0.10 mmol, 1 equiv), $Cu(OAc)_2$ (5.45 mg, 30.0 µmol, 0.30 equiv), ligand (**L5**) (3.17 mg, 12.0 µmol, 0.12 equiv), silver carbonate (55.2 mg, 0.20 mmol, 2.00 equiv), and cesium carbonate (16.3 mg, 50.0 µmol, 0.50 equiv). The reaction vessel was then sealed using a screw cap with a teflon coated septa. The reaction vessel was than placed under nitrogen atmosphere by using a vacuum manifold with a process of evacuating the headspace for 1.0 minute and venting with nitrogen for five times. Lastly, a pivalic acid (3.10 mg, 30.0 µmol, 0.30 equiv) dissolved in DMSO (1.0 mL) was added via a syringe to the reaction mixture at 23 °C. The reaction vessel was then placed into an oil bath that had been preheated to 130 °C. The reaction mixture was allowed to stir for 12 hours at 130 °C. After being allowed to cool to room temperature, the product mixture was diluted with ethyl acetate (10 mL) and water (10 mL). The resulting biphasic mixture was transferred to a separatory funnel and the layers that formed were separated. The aqueous layer was extracted with ethyl acetate (3 × 10 mL). The organic layers were combined, and the combined organic layers were washed with saturated aqueous sodium chloride solution (10 mL). The washed organic layer was dried over sodium sulfate. The dried solution was filtered and the filtrate was concentrated. The residue obtained was purified by flash-column chromatography (eluting with 5% ethyl acetate–hexanes) to provide the tricyclic product **43** as a colorless oil (14.5 mg, 52%).

Note: The relative stereochemistry at the C5 position was established by NOE analysis. Correlations between the C1 hydrogen and the vinyl hydrogen at C11 support the relative assignment shown.

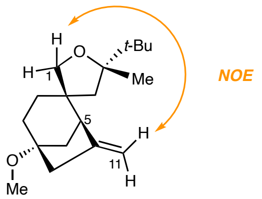


$R_f$ = 0.50 (10% ethyl acetate–hexanes; PAA). $^1$H NMR (400 MHz, $C_6D_6$): δ 4.79 (s, 1H), 4.71 (s, 1H), 3.91 (d, $J$ = 9.1 Hz, 1H), 3.52 (d, $J$ = 9.1 Hz, 1H), 3.07 (s, 3H), 2.44 (d, $J$ = 5.7 Hz, 1H), 2.40 – 2.30 (m, 1H), 2.20 – 2.10 (m, 1H), 1.79 (ddd, $J$ = 10.8, 5.7, 2.7 Hz, 1H), 1.72 (d, $J$ = 13.0 Hz, 1H), 1.63 – 1.53 (m, 3H), 1.39 – 1.25 (m, 2H), 1.15 (s, 3H), 1.01 (s, 9H). $^{13}$C NMR (101 MHz, $C_6D_6$): δ 151.2, 107.0, 88.6, 81.9, 78.0, 52.7, 50.6, 48.1, 43.5, 40.4, 39.8, 37.5, 32.6, 32.3, 25.9, 22.4. HRMS-CI (m/z): $[M + H]^+$ calcd $C_{18}H_{31}O_2$ for 279.2324; found, 279.2322.

## Synthetic Procedure for Preparation of ligand L5.

*Part 1: Synthesis of the quinoline–pyridine **S24**:*

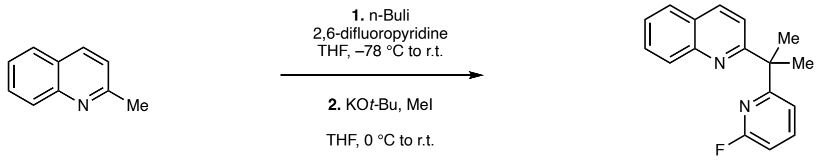


**S23** **S24**

A solution of *n*-butyllithium (2.5 M in hexanes, 38 mmol, 15.2 mL, 1.0 equiv.) was added dropwise via syringe to a solution of the quinaldine **S23** (5.40 g, 38.0 mmol, 1 equiv) in tetrahydrofuran (80 mL) at –78 °C. The reaction mixture was stirred at –78 °C for 1 hour before 2,6-difluoropyridine (3.50 mL, 38 mmol, 1.0 equiv.) was added to the reaction mixture at –78 °C. The resulting reaction mixture was allowed to gradually warm to 23 °C over the period of 3 hours. The product mixture was then diluted sequentially with saturated aqueous ammonium chloride solution (30 mL), water (50 mL), and ethyl acetate (60 mL). The resulting biphasic mixture was transferred to a separatory funnel and the layers that formed were separated. The aqueous layer was extracted with ethyl acetate (3 × 40 mL). The organic layers were combined and the combined organic layers were washed with saturated aqueous sodium chloride solution (50 mL). The washed organic layer was dried over sodium sulfate. The dried solution was filtered, and the filtrate was concentrated. The residue obtained was used in the subsequent step without further purification.

Potassium *tert*-butoxide (4.62 g, 38.0 mmol, 1.0 equiv.) was added in one portion to residue obtained from the previous step (nominally, 38 mmol, 1.0 equiv) dissolved in tetrahydrofuran (70 mL) at 0 °C. The reaction mixture was allowed to stir at 0 °C for 30 min before methyl iodide (4.38 mL, 38 mmol, 1.0 equiv.) was added dropwise via syringe over the period of 10 min. The resulting reaction mixture was allowed to warm to 0 °C for the period of 1 hour. Additional portion of potassium *tert*-butoxide (4.62 g, 38.0 mmol, 1.0 equiv.) was then added to the reaction mixture and allowed to stir at 0 °C for 30 min before an additional portion of methyl iodide (4.38 mL, 38 mmol, 1.0 equiv.) was added dropwise via syringe over the period of 10 min. The resulting reaction mixture was allowed to stir at 23 °C for 5 hours. The product mixture was then diluted sequentially with saturated aqueous ammonium chloride solution (30 mL), water (50 mL), and ethyl acetate (60 mL). The resulting biphasic mixture was transferred to a separatory funnel and the layers that formed were separated. The aqueous layer was extracted with ethyl acetate (3 × 40 mL). The organic layers were combined, and the combined organic layers were washed with saturated aqueous sodium chloride solution (50 mL). The washed organic layer was dried over sodium sulfate. The dried solution was filtered, and the filtrate was concentrated. The residue obtained was eluted over a short plug of silica gel with 30% ethyl acetate–hexanes to provide the corresponding alkylated ester **S24** as a yellow oil, which was used directly in the next step without any further purification.

*Part 2: Synthesis of the quinoline–pyridine ligand **L5**:*

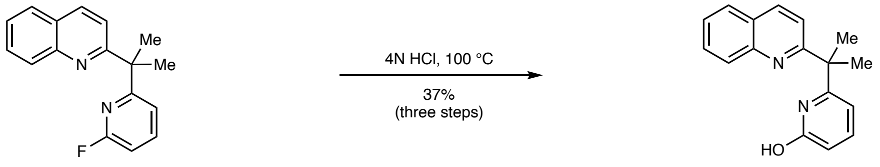


**S24** **L5**

Hydrochloric acid (4N, 76.0 mL, 304 mmol, 8.0 equiv) was added to the residue obtained from the previous step corresponding to **S24** (nominally, 38 mmol, 1.0 equiv). The resulting reaction mixture was placed into an oil bath that had been preheated to 100 °C. The reaction mixture was allowed to stir for 12 hours at 100 °C. After being allowed to cool to room temperature, a saturated solution of aqueous sodium bicarbonate (60 mL) was dropwise added to the product mixture. The product mixture was then diluted with dichloromethane (100 mL) and was transferred to a separatory funnel. The layers that formed were separated. The aqueous layer was extracted with dichloromethane (3 × 50 mL). The organic layers were combined, and the combined organic layers were washed with saturated aqueous sodium chloride solution (60 mL). The washed organic layer was dried over sodium sulfate. The dried solution was filtered, and the filtrate was concentrated. The residue obtained was purified by flash-column chromatography (eluting with 80% ethyl acetate–hexanes) to provide the ligand **L5** as an off-white solid. The obtained white solid was the recrystallized from hexane–ethyl acetate (1:2 v/v) to provide the ligand **L5** as a crystalline solid (3.70 g, 37%).

$R_f$ = 0.40 (100% ethyl acetate; PAA). $^1$H NMR (500 MHz, $CDCl_3$): δ 10.41 (s, 1H), 8.12 (d, $J$ = 8.5 Hz, 1H), 8.06 (d, $J$ = 8.6 Hz, 1H), 7.74 (d, $J$ = 8.1 Hz, 1H), 7.71 – 7.67 (m, 1H), 7.53 – 7.48 (m, 1H), 7.33 (d, $J$ = 8.6 Hz, 1H), 7.27 – 7.23 (m, 1H), 6.22 (d, $J$ = 9.2 Hz, 1H), 6.16 (d, $J$ = 7.0 Hz, 1H), 1.82 (s, 6H). $^{13}$C NMR (126 MHz, $CDCl_3$) δ 163.9, 163.4, 153.6, 147.3, 140.8, 137.1, 129.8, 129.6, 127.3, 126.8, 126.8, 118.5, 118.4, 102.0, 44.5, 27.6.

The spectroscopic data for the corresponding ligand **L5** prepared according to this procedure matched the previously reported $^1$H and $^{13}$C NMR data.[13,14]

**Characterization of Substrates and Products.**

Substrates:

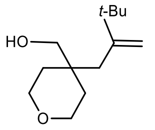

**13a**

(4-(3,3-dimethyl-2-methylenebutyl)tetrahydro-2*H*-pyran-4-yl)methanol (**13a**). $R_f$ = 0.40 (30% ethyl acetate–hexanes). Prepared according to general procedure A: $^1$H NMR (400 MHz, Benzene-$d_6$): δ 5.09 (d, $J$ = 1.0 Hz, 1H), 4.87 (d, $J$ = 1.1 Hz, 1H), 3.66 – 3.60 (m, 2H), 3.54 – 3.47 (m, 2H), 3.39 (d, $J$ = 4.6 Hz, 2H), 2.10 (d, $J$ = 1.2 Hz, 2H), 1.52 – 1.38 (m, 4H), 1.04 (s, 9H). $^{13}$C NMR (101 MHz, Benzene-$d_6$): δ 154.3, 110.2, 65.5, 63.9, 37.2, 37.1, 36.7, 33.9, 29.7. HRMS-CI (m/z): $[M + H]^+$ calcd for $C_{13}H_{25}O_2$, 213.1855; found, 213.1854.

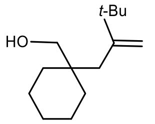

**13b**

(1-(3,3-dimethyl-2-methylenebutyl)cyclohexyl)methanol (**13b**). $R_f$ = 0.60 (20% ethyl acetate–hexanes). Prepared according to general procedure A: $^1$H NMR (400 MHz, Benzene-$d_6$): δ 5.12 (s, 1H), 4.99 (s, 1H), 3.43 (s, 2H), 2.12 (s, 2H), 1.48 – 1.31 (m, 10H), 1.07 (s, 9H). $^{13}$C NMR (101 MHz, Benzene-$d_6$): δ 155.0, 110.0, 66.7, 38.9, 37.2, 37.1, 33.9, 29.8, 26.7, 22.2. HRMS-CI (m/z): $[M + H]^+$ calcd for $C_{14}H_{27}O$, 211.2062; found, 211.2058.

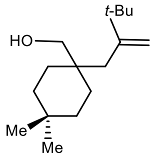

**13c**

(1-(3,3-dimethyl-2-methylenebutyl)-4,4-dimethylcyclohexyl)methanol (**13c**). $R_f$ = 0.60 (20% ethyl acetate–hexanes). Prepared according to general procedure A: $^1$H NMR (400 MHz, Benzene-$d_6$): δ 5.13 (d, $J$ = 1.1 Hz, 1H), 5.00 (d, $J$ = 1.0 Hz, 1H), 3.40 (d, $J$ = 5.3 Hz, 2H), 2.13 (d, $J$ = 1.1 Hz, 2H), 1.47 – 1.43 (m, 4H), 1.23 – 1.20 (m, 4H), 1.08 (s, 9H), 0.89 (s, 3H), 0.89 (s, 3H). $^{13}$C NMR (101 MHz, Benzene-$d_6$): δ 155.1, 110.0, 66.4, 38.6, 37.3, 36.9, 35.1, 30.0, 29.8, 29.5, 27.7. HRMS-CI (m/z): $[M + H]^+$ calcd for $C_{16}H_{31}O$, 239.2375; found, 239.2375.

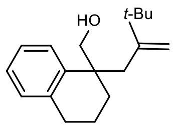

**13d**

(1-(3,3-dimethyl-2-methylenebutyl)-1,2,3,4-tetrahydronaphthalen-1-yl)methanol (**13d**). $R_f$ = 0.40 (20% ethyl acetate–hexanes). Prepared according to general procedure A: $^1$H NMR (400 MHz, Benzene-$d_6$): δ 7.25 – 7.21 (m, 1H), 7.08 – 6.96 (m, 3H), 4.88 – 4.85 (m, 1H), 4.37 – 4.34 (m, 1H), 3.43 (dd, *J* = 10.8, 4.6 Hz, 1H), 3.31 (dd, *J* = 10.9, 5.4 Hz, 1H), 2.74 (dt, *J* = 17.0, 2.1 Hz, 1H), 2.70 – 2.54 (m, 2H), 2.50 (d, *J* = 17.0 Hz, 1H), 2.11 – 1.98 (m, 1H), 1.82 – 1.61 (m, 3H), 1.03 (s, 9H). $^{13}$C NMR (101 MHz, Benzene-$d_6$): δ 153.2, 141.0, 138.5, 129.6, 127.4, 126.4, 126.1, 109.2, 72.1, 42.6, 37.0, 36.9, 31.0, 29.3, 28.5, 20.1. HRMS-CI (m/z): $[M + H]^+$ calcd for $C_{18}H_{27}O$, 259.2062; found, 259.3059.

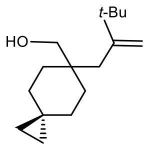

**13e**

(6-(3,3-dimethyl-2-methylenebutyl)spiro[2.5]octan-6-yl)methanol (**13e**). $R_f$ = 0.60 (20% ethyl acetate–hexanes). Prepared according to general procedure A: $^1$H NMR (400 MHz, Benzene-$d_6$): δ 5.14 (d, *J* = 1.1 Hz, 1H), 5.02 (d, *J* = 1.1 Hz, 1H), 3.47 (s, 2H), 2.17 (d, *J* = 1.1 Hz, 2H), 1.57 – 1.46 (m, 4H), 1.37 – 1.29 (m, 2H), 1.09 (s, 9H), 1.08 – 1.03 (m, 2H), 0.24 – 0.18 (m, 4H). $^{13}$C NMR (101 MHz, Benzene-$d_6$): δ 155.0, 110.0, 66.4, 38.7, 37.3, 36.7, 32.9, 31.4, 29.8, 18.9, 12.6, 12.4. HRMS-CI (m/z): $[M + H]^+$ calcd for $C_{16}H_{29}O$, 237.2218; found, 237.2218.

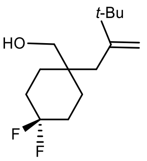

**13f**

(1-(3,3-dimethyl-2-methylenebutyl)-4,4-difluorocyclohexyl)methanol (**13f**). $R_f$ = 0.40 (25% ethyl acetate–hexanes). Prepared according to general procedure A: $^1$H NMR (400 MHz, Chloroform-*d*): δ 5.10 (s, 1H), 4.90 (s, 1H), 3.61 (s, 2H), 2.14 (d, *J* = 1.1 Hz, 2H), 1.97 – 1.84 (m, 4H), 1.73 – 1.64 (m, 4H), 1.08 (s, 9H). $^{13}$C NMR (101 MHz, Chloroform-*d*): δ 154.7, 123.7 (t, *J* = 240.8 Hz), 110.1, 66.2, 37.8, 37.3, 35.7, 30.0 (t, *J* = 24.24 Hz), 30.0 (t, *J* = 5.05 Hz), 29.6. $^{19}$F NMR (471 MHz, Benzene-$d_6$): δ -94.05 (d, *J* = 235.9 Hz), -97.63 (d, *J* = 236.2 Hz). HRMS-CI (m/z): $[M + H]^+$ calcd for $C_{14}H_{25}F_2O$, 247.1873; found, 247.1865.

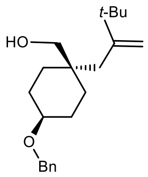

**13i**

(4-(benzyloxy)-1-(3,3-dimethyl-2-methylenebutyl)cyclohexyl)methanol (**13i**). $R_f$ = 0.40 (25% ethyl acetate–hexanes). Prepared according to general procedure A: $^1H$ NMR (400 MHz, Benzene-$d_6$): δ 7.40 – 7.36 (m, 2H), 7.24 – 7.20 (m, 2H), 7.15 – 7.10 (m, 1H), 5.13 (s, 1H), 4.95 (s, 1H), 4.41 (s, 2H), 3.41 (s, 2H), 3.24 – 3.16 (m, 1H), 2.07 (s, 2H), 1.78 – 1.65 (m, 4H), 1.53 – 1.44 (m, 2H), 1.21 – 1.13 (m, 2H), 1.07 (s, 9H). $^{13}C$ NMR (101 MHz, Benzene-$d_6$): δ 154.8, 140.2, 128.6, 127.6, 127.5, 110.2, 76.9, 69.9, 64.9, 38.4, 37.7, 37.2, 31.2, 29.8, 27.9. HRMS-CI (m/z): $[M + H]^+$ calcd for $C_{21}H_{33}O_2$, 317.2481; found, 317.2480.

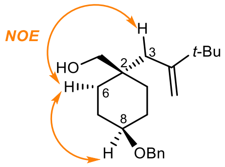


**13i**

Note: The relative stereochemistry at the C2 and C8 position was established by NOE analysis. Correlations between the hydrogen at C3 and the hydrogen at C6 as well as correlations between the hydrogen at C6 and the hydrogen at C8 support the relative assignment shown.

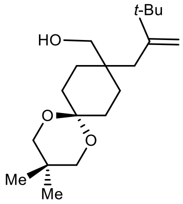

**13j**

(9-(3,3-dimethyl-2-methylenebutyl)-3,3-dimethyl-1,5-dioxaspiro[5.5]undecan-9-yl)methanol (**13j**). $R_f$ = 0.60 (30% ethyl acetate–hexanes). Prepared according to general procedure A: $^1H$ NMR (400 MHz, Chloroform-$d$): δ 5.06 (s, 1H), 4.91 (s, 1H), 3.60 (s, 2H), 3.49 (s, 4H), 2.12 (s, 2H), 1.90 – 1.83 (m, 2H), 1.75 – 1.68 (m, 2H), 1.56 – 1.52 (m, 4H), 1.06 (s, 9H), 0.96 (s, 6H). $^{13}C$ NMR (101 MHz, Chloroform-$d$): δ 155.2, 109.7, 97.7, 70.1, 70.1, 66.9, 38.3, 37.3, 36.2, 30.4, 29.8, 29.6, 28.2, 22.9. HRMS-CI (m/z): $[M + H]^+$ calcd for $C_{19}H_{35}O_3$, 311.2586; found, 311.2583.

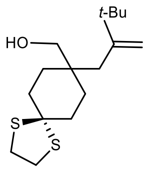

13k

(8-(3,3-dimethyl-2-methylenebutyl)-1,4-dithiaspiro[4.5]decan-8-yl)methanol (**13k**). $R_f$ = 0.40 (20% ethyl acetate–hexanes). Prepared according to general procedure A: $^1$H NMR (400 MHz, Benzene-$d_6$): δ 5.11 (d, $J$ = 1.0 Hz, 1H), 4.95 (d, $J$ = 1.1 Hz, 1H), 3.29 (s, 2H), 2.83 – 2.79 (m, 4H), 2.12 – 2.05 (m, 6H), 1.75 – 1.64 (m, 4H), 1.04 (s, 9H). $^{13}$C NMR (101 MHz, Benzene-$d_6$): δ 154.6, 110.2, 69.0, 65.7, 38.9, 38.5, 38.3, 37.7, 37.2, 33.3, 29.8. HRMS-CI (m/z): $[M + H]^+$ calcd for $C_{16}H_{29}OS_2$, 301.1660; found, 301.1658.

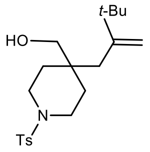

13l

(4-(3,3-dimethyl-2-methylenebutyl)-1-tosylpiperidin-4-yl)methanol (**13l**). $R_f$ = 0.40 (30% ethyl acetate–hexanes). Prepared according to general procedure A: $^1$H NMR (400 MHz, Benzene-$d_6$): δ 7.70 (d, $J$ = 8.3 Hz, 2H), 6.83 (d, $J$ = 7.7 Hz, 2H), 4.99 (d, $J$ = 0.9 Hz, 1H), 4.71 (s, 1H), 3.27 – 3.20 (m, 2H), 3.00 (s, 2H), 2.72 – 2.63 (m, 2H), 1.91 (s, 3H), 1.85 (s, 2H), 1.43 – 1.35 (m, 4H), 0.92 (s, 9H). $^{13}$C NMR (101 MHz, Benzene-$d_6$): δ 153.8, 142.9, 134.7, 129.6, 128.2, 110.4, 64.5, 42.5, 37.0, 36.5, 36.3, 32.2, 29.6, 21.1. HRMS-CI (m/z): $[M + H]^+$ calcd for $C_{20}H_{32}NO_3S$, 366.2103; found, 366.2105

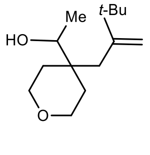

13m

1-(4-(3,3-dimethyl-2-methylenebutyl)tetrahydro-2*H*-pyran-4-yl)ethan-1-ol (**13m**). $R_f$ = 0.40 (30% ethyl acetate–hexanes). Prepared according to general procedure C: $^1$H NMR (400 MHz, Benzene-$d_6$): δ 5.11 (s, 1H), 4.89 (s, 1H), 3.96 (q, $J$ = 6.4 Hz, 1H), 3.69 – 3.57 (m, 4H), 2.43 (dd, $J$ = 16.2, 1.2 Hz, 1H), 1.97 (d, $J$ = 16.2 Hz, 1H), 1.73 – 1.61 (m, 2H), 1.47 – 1.39 (m, 2H), 1.06 (s, 9H), 0.90 (d, $J$ = 6.4 Hz, 3H). $^{13}$C NMR (101 MHz, Benzene-$d_6$): δ 154.2, 110.1, 68.9, 63.7, 63.6, 39.0, 37.4, 32.6, 32.0, 31.2, 29.9, 17.5. HRMS-CI (m/z): $[M + H]^+$ calcd for $C_{14}H_{27}O_2$, 227.2011; found, 227.2010.

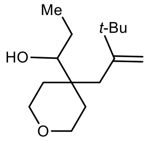

**13n**

1-(4-(3,3-dimethyl-2-methylenebutyl)tetrahydro-2*H*-pyran-4-yl)propan-1-ol (**13n**). $R_f$ = 0.60 (30% ethyl acetate–hexanes). Prepared according to general procedure C: $^{1}$H NMR (400 MHz, Benzene-$d_6$): δ 5.11 (s, 1H), 4.91 (s, 1H), 3.70 – 3.59 (m, 5H), 2.43 (dd, $J$ = 16.2, 1.2 Hz, 1H), 1.98 (d, $J$ = 16.3 Hz, 1H), 1.78 – 1.64 (m, 2H), 1.51 – 1.42 (m, 2H), 1.36 – 1.29 (m, 1H), 1.21 – 1.15 (m, 1H), 1.06 (s, 9H), 0.91 (t, $J$ = 7.3 Hz, 3H). $^{13}$C NMR (101 MHz, Benzene-$d_6$): δ 154.2, 110.1, 75.3, 63.8, 63.7, 39.3, 37.4, 33.0, 32.3, 31.5, 29.9, 23.8, 11.9. HRMS-CI (m/z): $[M + H]^+$ calcd for $C_{15}H_{29}O_2$, 241.2168; found, 241.2168.

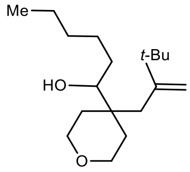

**13o**

1-(4-(3,3-dimethyl-2-methylenebutyl)tetrahydro-2*H*-pyran-4-yl)hexan-1-ol (**13o**). $R_f$ = 0.40 (25% ethyl acetate–hexanes). Prepared according to general procedure C: $^{1}$H NMR (400 MHz, Chloroform-*d*): δ 5.08 (s, 1H), 4.90 (s, 1H), 3.98 – 3.91 (m, 1H), 3.78 – 3.64 (m, 4H), 2.42 (d, $J$ = 16.1 Hz, 1H), 2.13 (d, $J$ = 16.5 Hz, 1H), 1.85 – 1.76 (m, 1H), 1.76 – 1.64 (m, 2H), 1.61 – 1.47 (m, 4H), 1.35 – 1.28 (m, 5H), 1.08 (s, 9H), 0.90 (t, $J$ = 6.8 Hz, 3H). $^{13}$C NMR (101 MHz, Chloroform-*d*): δ 154.2, 109.9, 74.3, 63.8, 63.7, 39.0, 37.5, 32.7, 32.1, 32.0, 31.4, 30.9, 29.8, 27.0, 22.8, 14.2. HRMS-CI (m/z): $[M + H]^+$ calcd for $C_{18}H_{35}O_2$, 283.2637; found, 283.2634.

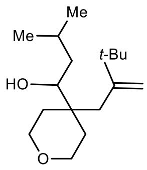

**13p**

1-(4-(3,3-dimethyl-2-methylenebutyl)tetrahydro-2*H*-pyran-4-yl)-3-methylbutan-1-ol (**13p**). $R_f$ = 0.40 (25% ethyl acetate–hexanes). Prepared according to general procedure C: $^{1}$H NMR (400 MHz, Benzene-$d_6$): δ 5.12 (s, 1H), 4.93 (s, 1H), 3.96 – 3.91 (m, 1H), 3.73 – 3.60 (m, 4H), 2.45 (dd, $J$ = 16.3, 1.2 Hz, 1H), 1.97 (d, $J$ = 16.3 Hz, 1H), 1.76 – 1.64 (m, 3H), 1.52 – 1.40 (m, 2H), 1.32 – 1.25 (m, 1H), 1.06 (s, 9H), 0.94 – 0.90 (m, 4H), 0.84 (d, $J$ = 6.6 Hz, 3H). $^{13}$C NMR (101 MHz, Benzene-$d_6$): δ 154.1, 110.1, 71.1, 63.7, 63.6, 40.1, 39.1, 37.4, 32.9, 32.2, 31.5, 29.9, 25.3, 24.5, 21.6. HRMS-CI (m/z): $[M + H]^+$ calcd for $C_{17}H_{33}O_2$, 269.2481; found, 269.2479.

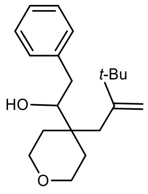


**13q**

1-(4-(3,3-dimethyl-2-methylenebutyl)tetrahydro-2*H*-pyran-4-yl)-2-phenylethan-1-ol (**13q**). $R_f$ = 0.40 (25% ethyl acetate–hexanes). Prepared according to general procedure C: $^1$H NMR (400 MHz, Benzene-$d_6$): δ 7.20 – 7.16 (m, 2H), 7.13 – 7.08 (m, 3H), 5.15 (s, 1H), 4.97 (s, 1H), 4.04 – 3.97 (m, 1H), 3.73 – 3.63 (m, 4H), 2.68 – 2.58 (m, 2H), 2.43 (dd, *J* = 13.3, 10.7 Hz, 1H), 2.05 (d, *J* = 16.3 Hz, 1H), 1.94 – 1.86 (m, 1H), 1.82 – 1.75 (m, 1H), 1.64 – 1.56 (m, 1H), 1.51 – 1.43 (m, 1H), 1.09 (s, 9H). $^{13}$C NMR (101 MHz, Benzene-$d_6$): δ 154.0, 140.5, 129.9, 128.9, 126.6, 110.2, 74.5, 63.7, 63.6, 39.1, 37.9, 37.4, 33.2, 32.4, 31.6, 29.9. HRMS-CI (m/z): $[M + H]^+$ calcd for $C_{20}H_{31}O_2$, 303.2324; found, 303.2322.

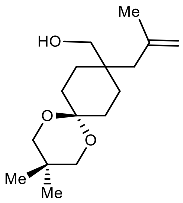


**13s**

(3,3-dimethyl-9-(2-methylallyl)-1,5-dioxaspiro[5.5]undecan-9-yl)methanol (**13s**). $R_f$ = 0.60 (30% ethyl acetate–hexanes). Prepared according to general procedure D: $^1$H NMR (400 MHz, Chloroform-*d*): δ 4.90 – 4.86 (m, 1H), 4.76 – 4.73 (m, 1H), 3.50 (s, 2H), 3.48 (s, 4H), 2.11 (s, 2H), 1.85 – 1.74 (m, 7H), 1.50 – 1.41 (m, 4H), 0.96 (s, 6H). $^{13}$C NMR (101 MHz, Chloroform-*d*): δ 144.2, 114.7, 97.8, 70.1, 70.1, 67.7, 43.4, 38.3, 30.3, 29.2, 28.1, 25.4, 22.9. HRMS-CI (m/z): $[M + H]^+$ calcd for $C_{16}H_{29}O_3$, 269.2117; found, 269.2115.

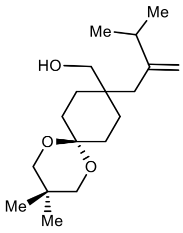


**13t**

(3,3-dimethyl-9-(3-methyl-2-methylenebutyl)-1,5-dioxaspiro[5.5]undecan-9-yl)methanol (**13t**). $R_f$ = 0.60 (30% ethyl acetate–hexanes). Prepared according to general procedure D: $^1$H NMR (400 MHz, Benzene-$d_6$): δ 4.99 – 4.97 (m, 1H), 4.84 – 4.82 (m, 1H), 3.33 (d, $J$ = 4.2 Hz, 4H), 3.28 (s, 2H), 2.30 – 2.22 (m, 1H), 2.17 (s, 2H), 1.92 – 1.85 (m, 2H), 1.80 – 1.73 (m, 2H), 1.52 – 1.48 (m, 4H), 1.04 (d, $J$ = 6.8 Hz, 6H), 0.80 (s, 6H). $^{13}$C NMR (101 MHz, Benzene-$d_6$): δ 154.5, 110.3, 97.9, 69.9, 69.9, 66.5, 38.6, 34.5, 30.2, 29.3, 28.5, 22.8, 22.6. HRMS-CI (m/z): $[M + H]^+$ calcd for $C_{18}H_{33}O_3$, 297.2430; found, 297.2429.

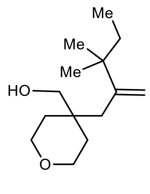


**13u**

(4-(3,3-dimethyl-2-methylenepentyl)tetrahydro-2*H*-pyran-4-yl)methanol (**13u**). $R_f$ = 0.40 (30% ethyl acetate–hexanes). Prepared according to general procedure D: $^1$H NMR (400 MHz, Benzene-$d_6$): δ 5.03 – 5.02 (m, 1H), 4.98 – 4.96 (m, 1H), 3.66 – 3.60 (m, 2H), 3.54 – 3.47 (m, 2H), 3.40 (d, $J$ = 3.3 Hz, 2H), 2.04 (d, $J$ = 1.2 Hz, 2H), 1.52 – 1.39 (m, 4H), 1.32 (q, $J$ = 7.4 Hz, 2H), 0.99 (s, 6H), 0.75 (t, $J$ = 7.4 Hz, 3H). $^{13}$C NMR (101 MHz, Benzene-$d_6$): δ 152.1, 111.7, 65.5, 63.9, 40.4, 36.7, 36.6, 34.0, 33.9, 27.2, 9.3. HRMS-CI (m/z): $[M + H]^+$ calcd for $C_{14}H_{27}O_2$, 227.2011; found, 227.2008.

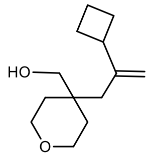


**13v**

(4-(2-cyclobutylallyl)tetrahydro-2*H*-pyran-4-yl)methanol (**13v**). $R_f$ = 0.40 (35% ethyl acetate–hexanes). Prepared according to general procedure D: $^1$H NMR (400 MHz, Benzene-$d_6$): δ 4.93 – 4.90 (m, 1H), 4.83 – 4.80 (m, 1H), 3.61 – 3.50 (m, 4H), 3.20 (d, $J$ = 4.2 Hz, 2H), 2.88 – 2.75 (m, 1H), 2.05 – 1.99 (m, 4H), 1.89 – 1.79 (m, 2H), 1.77 – 1.68 (m, 1H), 1.65 – 1.57 (m, 1H), 1.34 – 1.26 (m, 4H). $^{13}$C NMR (101 MHz, Benzene-$d_6$): δ 151.1, 111.8, 66.6, 63.8, 42.5, 40.1, 36.4, 33.4, 28.8, 17.6. HRMS-CI (m/z): $[M + H]^+$ calcd for $C_{13}H_{23}O_2$, 211.1698; found, 211.1696.

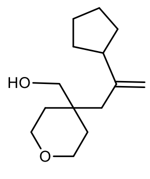


**13w**

(4-(2-cyclopentylallyl)tetrahydro-2*H*-pyran-4-yl)methanol (**13w**). R$_f$ = 0.40 (35% ethyl acetate–hexanes). Prepared according to general procedure D: $^1$H NMR (400 MHz, Benzene-$d_6$): δ 4.98 – 4.96 (m, 1H), 4.78 – 4.76 (m, 1H), 3.64 – 3.51 (m, 4H), 3.26 (d, *J* = 4.7 Hz, 2H), 2.38 – 2.29 (m, 1H), 2.14 (s, 2H), 1.85 – 1.77 (m, 2H), 1.69 – 1.58 (m, 2H), 1.54 – 1.44 (m, 2H), 1.38 – 1.31 (m, 6H). $^{13}$C NMR (101 MHz, Benzene-$d_6$): δ 151.1, 111.0, 66.4, 63.8, 47.4, 42.5, 36.7, 33.4, 32.7, 25.2. HRMS-CI (m/z): [M + H]$^+$ calcd for $C_{14}H_{25}O_2$, 225.1855; found, 225.1852.

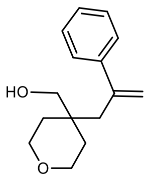


**13x**

(4-(2-phenylallyl)tetrahydro-2*H*-pyran-4-yl)methanol (**13x**). R$_f$ = 0.40 (35% ethyl acetate–hexanes). Prepared according to general procedure D: $^1$H NMR (400 MHz, Benzene-$d_6$): δ 7.27 – 7.22 (m, 2H), 7.15 – 7.01 (m, 3H), 5.20 (d, *J* = 2.0 Hz, 1H), 5.02 – 4.98 (m, 1H), 3.55 – 3.43 (m, 4H), 3.09 (s, 2H), 2.55 (s, 2H), 1.27 (t, *J* = 5.5 Hz, 4H). $^{13}$C NMR (101 MHz, Benzene-$d_6$): δ 146.8, 143.9, 128.6, 127.6, 126.7, 117.5, 66.2, 63.7, 40.6, 36.9, 33.2. HRMS-CI (m/z): [M + H]$^+$ calcd for $C_{15}H_{21}O_2$, 233.1542; found, 233.1535.

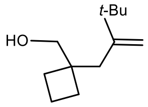


**13y**

(1-(3,3-dimethyl-2-methylenebutyl)cyclobutyl)methanol (**13y**). R$_f$ = 0.60 (20% ethyl acetate–hexanes). Prepared according to general procedure A: $^1$H NMR (400 MHz, Benzene-$d_6$): δ 4.97 (d, *J* = 1.0 Hz, 1H), 4.65 (d, *J* = 1.3 Hz, 1H), 3.56 (s, 2H), 2.23 (s, 2H), 1.93 – 1.75 (m, 6H), 1.04 (s, 9H). $^{13}$C NMR (101 MHz, Benzene-$d_6$): δ 155.1, 107.2, 66.5, 43.3, 38.2, 36.6, 30.6, 29.3, 16.6. HRMS-CI (m/z): [M + H]$^+$ calcd for $C_{12}H_{23}O$, 183.1749; found, 183.1751.

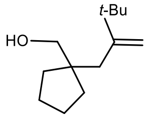


**13z**

(1-(3,3-dimethyl-2-methylenebutyl)cyclopentyl)methanol (**13z**). R$_f$ = 0.60 (20% ethyl acetate–hexanes). Prepared according to general procedure A: $^1$H NMR (400 MHz, Benzene-$d_6$): δ 5.05 (q, *J* = 0.8 Hz, 1H), 4.92 (q, *J* = 1.3 Hz, 1H), 3.36 (s, 2H), 2.21 (dd, *J* = 1.5, 0.8 Hz, 2H), 1.60 –

1.43 (m, 8H), 1.06 (s, 9H). $^{13}$C NMR (101 MHz, Benzene-$d_6$): δ 155.3, 108.1, 67.4, 47.7, 38.1, 37.0, 36.3, 29.5, 25.5. HRMS-CI (m/z): $[M + H]^+$ calcd for $C_{13}H_{25}O$, 197.1905; found, 197.1904.

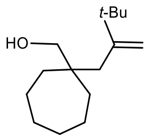

**13aa**

(1-(3,3-dimethyl-2-methylenebutyl)cycloheptyl)methanol (**13aa**). $R_f$ = 0.60 (20% ethyl acetate–hexanes). Prepared according to general procedure A: $^1$H NMR (400 MHz, Benzene-$d_6$): δ 5.15 (d, $J$ = 1.1 Hz, 1H), 5.07 (d, $J$ = 1.1 Hz, 1H), 3.31 (s, 2H), 2.10 (d, $J$ = 1.1 Hz, 2H), 1.45 – 1.35 (m, 12H), 1.07 (s, 9H). $^{13}$C NMR (101 MHz, Benzene-$d_6$): δ 154.8, 109.9, 67.7, 42.1, 37.3, 36.9, 36.1, 31.8, 29.7, 23.5. HRMS-CI (m/z): $[M + H]^+$ calcd for $C_{15}H_{29}O$, 225.2218; found, 225.2218.

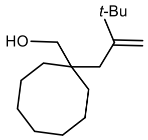

**13ab**

(1-(3,3-dimethyl-2-methylenebutyl)cyclooctyl)methanol (**13ab**). $R_f$ = 0.60 (20% ethyl acetate–hexanes). Prepared according to general procedure A: $^1$H NMR (400 MHz, Benzene-$d_6$): δ 5.15 (d, $J$ = 1.1 Hz, 1H), 5.04 (d, $J$ = 1.2 Hz, 1H), 3.35 (d, $J$ = 3.9 Hz, 2H), 2.08 (d, $J$ = 1.1 Hz, 2H), 1.51 – 1.37 (m, 14H), 1.08 (s, 9H). $^{13}$C NMR (101 MHz, Benzene-$d_6$): δ 155.0, 110.2, 67.3, 41.7, 37.4, 36.0, 31.5, 29.8, 29.3, 26.3, 23.5. HRMS-CI (m/z): $[M + H]^+$ calcd for $C_{16}H_{31}O$, 239.2375; found, 239.2374.

Annulated and Desaturated Products:

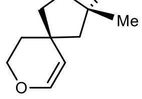

**14a**

(3S,5S)-3-(*tert*-butyl)-3-methyl-2,8-dioxaspiro[4.5]dec-6-ene (**14a**). $R_f$ = 0.50 (5% ethyl acetate–hexanes). Prepared according to general procedure E: $^1$H NMR (500 MHz, $CDCl_3$): δ 6.33 (d, $J$ = 6.2 Hz, 1H), 4.66 (d, $J$ = 6.2 Hz, 1H), 4.03 – 3.98 (m, 1H), 3.91 – 3.86 (m, 1H), 3.65 (d, $J$ = 9.2

Hz, 1H), 3.57 (d, $J$ = 9.1 Hz, 1H), 1.94 – 1.87 (m, 2H), 1.75 – 1.69 (m, 1H), 1.58 (d, $J$ = 13.2 Hz, 1H), 1.23 (s, 3H), 0.94 (s, 9H). $^{13}$C NMR (126 MHz, $CDCl_3$): δ 143.6, 108.2, 88.8, 77.7, 64.3, 48.9, 40.3, 37.3, 35.2, 25.9, 22.0. HRMS-CI (m/z): $[M + H]^+$ calcd for $C_{13}H_{23}O_2$, 211.1698; found, 211.1698.

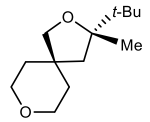


**18**

3-(*tert*-butyl)-3-methyl-2,8-dioxaspiro[4.5]decane (**18**). $R_f$ = 0.5 (20% ethyl acetate–hexanes). Prepared according to general procedure E: $^1$H NMR (400 MHz, $CDCl_3$): δ 3.72 (d, $J$ = 8.9 Hz, 1H), 3.69 – 3.55 (m, 5H), 1.89 (d, $J$ = 13.1 Hz, 1H), 1.71 – 1.52 (m, 4H), 1.46 (d, $J$ = 13.0 Hz, 1H), 1.19 (s, 3H), 0.92 (s, 9H). $^{13}$C NMR (101 MHz, $CDCl_3$): δ 88.2, 77.3, 65.9, 65.8, 45.8, 41.7, 37.7, 37.5, 37.2, 25.8, 22.7. HRMS-CI (m/z): $[M + H]^+$ calcd for $C_{13}H_{25}O_2$, 213.1855; found, 213.1854.

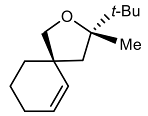


**14b**

(3S,5S)-3-(*tert*-butyl)-3-methyl-2-oxaspiro[4.5]dec-6-ene (**14b**). $R_f$ = 0.6 (5% ethyl acetate–hexanes). Prepared according to general procedure E: $^1$H NMR (400 MHz, $CDCl_3$): δ 5.69 – 5.62 (m, 2H), 3.64 – 3.59 (m, 2H), 1.98 – 1.88 (m, 3H), 1.71 – 1.47 (m, 5H), 1.21 (s, 3H), 0.94 (s, 9H). $^{13}$C NMR (126 MHz, $CDCl_3$): δ 134.7, 126.7, 88.6, 77.6, 48.7, 44.4, 37.5, 35.1, 25.9, 25.0, 22.2, 20.8. HRMS-CI (m/z): $[M + H]^+$ calcd for $C_{14}H_{25}O$, 209.1905; found, 209.1900.

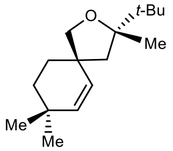


**14c**

(3S,5S)-3-(*tert*-butyl)-3,8,8-trimethyl-2-oxaspiro[4.5]dec-6-ene (**14c**). $R_f$ = 0.60 (5% ethyl acetate–hexanes). Prepared according to general procedure E: $^1$H NMR (400 MHz, $CDCl_3$): δ 5.52 (d, $J$ = 9.9 Hz, 1H), 5.36 (d, $J$ = 9.9 Hz, 1H), 3.59 (s, 2H), 1.92 (d, $J$ = 13.1 Hz, 1H), 1.74 – 1.66 (m, 1H), 1.56 – 1.49 (m, 2H), 1.47 – 1.36 (m, 2H), 1.20 (s, 3H), 0.97 (s, 3H), 0.95 (s, 3H), 0.94 (s, 9H). $^{13}$C NMR (101 MHz, $CDCl_3$): δ 137.3, 132.1, 88.6, 77.3, 48.5, 44.6, 37.5, 35.5, 32.3, 31.6, 29.9, 29.4, 25.9, 22.2. HRMS-CI (m/z): $[M + H]^+$ calcd for $C_{16}H_{29}O$, 237.2218; found, 237.2211.

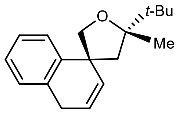

**14d**

(3S,5S)-5-(*tert*-butyl)-5-methyl-4,5-dihydro-2*H*,4'*H*-spiro[furan-3,1'-naphthalene] (**14d**). R*f* = 0.50 (5% ethyl acetate–hexanes). Prepared according to general procedure E: $^1$H NMR (400 MHz, $CDCl_3$): δ 7.42 – 7.37 (m, 1H), 7.22 – 7.12 (m, 2H), 7.05 – 7.02 (m, 1H), 6.51 – 6.43 (m, 1H), 6.01 – 5.94 (m, 1H), 4.21 (d, *J* = 9.4 Hz, 1H), 3.92 (d, *J* = 9.3 Hz, 1H), 2.60 (dt, *J* = 17.0, 3.0 Hz, 1H), 2.40 (dd, *J* = 17.0, 5.4 Hz, 1H), 2.16 (d, *J* = 13.3 Hz, 1H), 1.87 (d, *J* = 13.3 Hz, 1H), 1.31 (s, 3H), 0.90 (s, 9H). $^{13}$C NMR (101 MHz, $CDCl_3$): δ 141.7, 133.3, 128.9, 127.8, 126.9, 126.8, 126.5, 124.5, 89.0, 77.0, 46.6, 45.9, 37.9, 36.3, 26.0, 22.5. HRMS-CI (m/z): $[M + H]^+$ calcd for $C_{18}H_{25}O$, 257.1905; found, 257.1901.

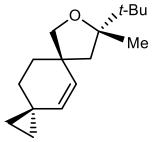

**14e**

(6S,9S)-9-(*tert*-butyl)-9-methyl-8-oxadispiro[2.2.4$^{6.2^3}$]dodec-11-ene (**14e**). R*f* = 0.60 (5% ethyl acetate–hexanes). Prepared according to general procedure E: $^1$H NMR (400 MHz, $CDCl_3$): δ 5.65 (d, *J* = 9.8 Hz, 1H), 4.99 (d, *J* = 9.8 Hz, 1H), 3.65 (s, 2H), 1.95 (d, *J* = 13.1 Hz, 1H), 1.85 – 1.78 (m, 1H), 1.64 – 1.47 (m, 4H), 1.23 (s, 3H), 0.95 (s, 9H), 0.57 – 0.48 (m, 4H). $^{13}$C NMR (101 MHz, $CDCl_3$): δ 134.2, 133.2, 88.7, 77.4, 48.6, 44.4, 37.5, 34.5, 31.4, 25.9, 22.2, 18.3, 14.8, 14.6. HRMS-CI (m/z): $[M + H]^+$ calcd for $C_{16}H_{27}O$, 235.2062; found, 235.2063.

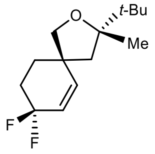

**14f**

(3S,5S)-3-(*tert*-butyl)-8,8-difluoro-3-methyl-2-oxaspiro[4.5]dec-6-ene (**14f**). R*f* = 0.40 (5% ethyl acetate–hexanes). Prepared according to general procedure E: $^1$H NMR (400 MHz, $CDCl_3$): δ 6.11 (d, *J* = 10.1 Hz, 1H), 5.75 – 5.68 (m, 1H), 3.67 (s, 2H), 2.16 – 2.04 (m, 2H), 2.01 (d, *J* = 13.3 Hz, 1H), 1.95 – 1.88 (m, 1H), 1.79 – 1.72 (m, 1H), 1.63 (d, *J* = 13.4 Hz, 1H), 1.22 (s, 3H), 0.95 (s, 9H). $^{13}$C NMR (101 MHz, $CDCl_3$): δ 143.3 (t, *J* = 10.5 Hz), 122.6 (t, *J* = 29.2 Hz), 119.1 (t, *J* = 232.6 Hz), 88.9, 75.6 (t, *J* = 2.7 Hz), 47.0 (t, *J* = 2.2 Hz), 44.9 (t, *J* = 2.1 Hz), 37.6, 31.6 (t, *J* = 4.4 Hz), 31.3 (d, *J* = 24.3 Hz), 25.8, 22.3. $^{19}$F NMR (471 MHz, Chloroform-*d*): δ -90.71, -90.74. HRMS-CI (m/z): $[M + H]^+$ calcd for $C_{14}H_{23}F_2O$, 245.1717; found, 245.1715.

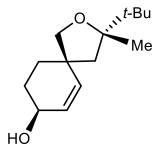

**14g**

(3S,5S,8S)-3-(*tert*-butyl)-3-methyl-2-oxaspiro[4.5]dec-6-en-8-ol (**14g**). $R_f$ = 0.40 (30% ethyl acetate–hexanes). Prepared according to general procedure E: $^1$H NMR (400 MHz, $CDCl_3$): δ 5.76 (d, $J$ = 10.1 Hz, 1H), 5.68 (dd, $J$ = 10.0, 2.7 Hz, 1H), 4.21 – 4.15 (m, 1H), 3.67 – 3.61 (m, 2H), 1.98 – 1.83 (m, 3H), 1.57 – 1.45 (m, 3H), 1.20 (s, 3H), 0.93 (s, 9H). $^{13}$C NMR (101 MHz, $CDCl_3$): δ 137.1, 129.8, 88.8, 76.7, 65.9, 48.0, 44.6, 37.4, 32.0, 30.8, 25.9, 22.1. HRMS-CI (m/z): $[M + H]^+$ calcd for $C_{14}H_{25}O_2$, 225.1855; found, 225.1853.

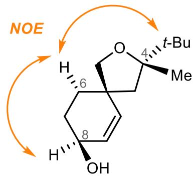


**14g**

Note: The relative stereochemistry of ether **14g** was established via NOE analysis. The correlation between the C4 *t*-butyl and the hydrogen at C6 as well as the correlation between the hydrogen at C6 and the hydrogen at C8 support the relative assignment shown.

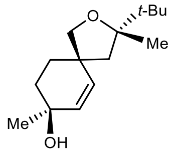

**14h**

(3S,5S,8S)-3-(*tert*-butyl)-3,8-dimethyl-2-oxaspiro[4.5]dec-6-en-8-ol (**14h**). $R_f$ = 0.60 (30% ethyl acetate–hexanes). Prepared according to general procedure E: $^1$H NMR (400 MHz, $CDCl_3$): δ 5.68 (d, $J$ = 9.9 Hz, 1H), 5.57 (d, $J$ = 9.9 Hz, 1H), 3.64 – 3.55 (m, 2H), 1.96 (d, $J$ = 13.2 Hz, 1H), 1.77 – 1.64 (m, 4H), 1.59 (d, $J$ = 13.2 Hz, 1H), 1.27 (s, 3H), 1.20 (s, 3H), 0.94 (s, 9H). $^{13}$C NMR (101 MHz, $CDCl_3$): δ 136.2, 133.0, 88.7, 75.7, 67.7, 48.2, 44.7, 37.4, 36.2, 32.1, 29.4, 25.9, 22.0. HRMS-CI (m/z): $[M + H]^+$ calcd for $C_{15}H_{27}O_2$, 239.2011; found, 239.2020.

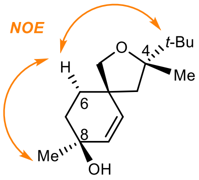


**14h**

Note: The relative stereochemistry of ether **14h** was established via NOE analysis. The correlation between the C4 *t*-butyl and the hydrogen at C6 as well as the correlation between the hydrogen at C6 and the methyl group at C8 support the relative assignment shown.

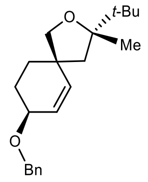


**14i**

(3S,5S,8S)-8-(benzyloxy)-3-(*tert*-butyl)-3-methyl-2-oxaspiro[4.5]dec-6-ene (**14i**). $R_f$ = 0.50 (10% ethyl acetate–hexanes). Prepared according to general procedure E: $^1$H NMR (400 MHz, $CDCl_3$): δ 7.39 – 7.31 (m, 4H), 7.30 – 7.25 (m, 1H), 5.81 – 5.74 (m, 2H), 4.57 (q, *J* = 11.8 Hz, 2H), 3.97 – 3.90 (m, 1H), 3.69 – 3.62 (m, 2H), 1.97 – 1.86 (m, 3H), 1.70 – 1.62 (m, 1H), 1.54 (d, *J* = 13.2 Hz, 1H), 1.50 – 1.41 (m, 1H), 1.21 (s, 3H), 0.94 (s, 9H). $^{13}$C NMR (101 MHz, $CDCl_3$): δ 138.9, 137.3, 128.5, 127.8, 127.7, 127.6, 88.7, 76.6, 72.6, 70.4, 48.0, 44.8, 37.4, 32.3, 27.2, 25.9, 22.0. HRMS-CI (m/z): $[M + H]^+$ calcd for $C_{21}H_{31}O_2$, 315.2324; found, 315.2320.

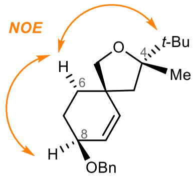


**14i**

Note: The relative stereochemistry of ether **14i** was established via NOE analysis. The correlation between the C4 *t*-butyl and the hydrogen at C6 as well as the correlation between the hydrogen at C6 and the hydrogen at C8 support the relative assignment shown.

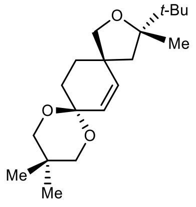


**14j**

(3S,5S)-3-(*tert*-butyl)-3,11,11-trimethyl-2,9,13-trioxadispiro[4.2.5$^{8.25}$]pentadec-6-ene (**14j**). $R_f$ = 0.40 (10% ethyl acetate–hexanes). Prepared according to general procedure E: $^1$H NMR (400 MHz, Benzene-$d_6$): δ 6.14 (d, *J* = 10.3 Hz, 1H), 5.71 (d, *J* = 10.3 Hz, 1H), 3.59 (s, 2H), 3.52 – 3.46 (m, 2H), 3.36 – 3.30 (m, 2H), 2.07 – 1.99 (m, 1H), 1.94 – 1.83 (m, 2H), 1.77 (d, *J* = 13.2 Hz, 1H), 1.73 – 1.65 (m, 1H), 1.38 (d, *J* = 13.2 Hz, 1H), 1.12 (s, 3H), 0.94 (s, 9H), 0.94 (s, 3H), 0.70 (s, 3H). $^{13}$C NMR (101 MHz, Benzene-$d_6$): δ 138.2, 124.6, 95.1, 88.3, 76.3, 70.7, 70.5, 47.7, 45.8,

37.6, 32.1, 31.8, 30.1, 26.0, 23.0, 22.5, 22.2. HRMS-CI (m/z): $[M + H]^+$ calcd for $C_{19}H_{33}O_3$, 309.2430; found, 309.2420.

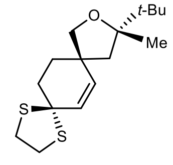


**14k**

(8S,11S)-11-(*tert*-butyl)-11-methyl-10-oxa-1,4-dithiadispiro[4.2.4$^{8.25}$]tetradec-13-ene (**14k**). $R_f$ = 0.40 (5% ethyl acetate–hexanes). Prepared according to general procedure E: $^1$H NMR (400 MHz, $CDCl_3$): δ 5.78 (d, $J$ = 9.7 Hz, 1H), 5.65 (d, $J$ = 9.7 Hz, 1H), 3.62 (s, 2H), 3.39 – 3.28 (m, 4H), 2.23 – 2.13 (m, 2H), 1.95 (d, $J$ = 13.3 Hz, 1H), 1.93 – 1.86 (m, 1H), 1.75 – 1.68 (m, 1H), 1.58 (d, $J$ = 13.2 Hz, 1H), 1.20 (s, 3H), 0.94 (s, 9H). $^{13}$C NMR (101 MHz, $CDCl_3$): δ 134.8, 131.4, 88.8, 76.6, 64.5, 48.0, 44.0, 40.1, 40.1, 39.8, 37.5, 35.0, 25.9, 22.2. HRMS-CI (m/z): $[M + H]^+$ calcd for $C_{16}H_{27}OS_2$, 299.1503; found, 299.1506.

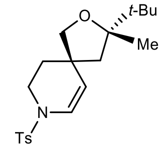


**14l**

(3S,5S)-3-(*tert*-butyl)-3-methyl-8-tosyl-2-oxa-8-azaspiro[4.5]dec-6-ene (**14l**). $R_f$ = 0.50 (20% ethyl acetate–hexanes). Prepared according to general procedure E: $^1$H NMR (400 MHz, $CDCl_3$): δ 7.65 (d, $J$ = 8.3 Hz, 2H), 7.31 (d, $J$ = 8.0 Hz, 2H), 6.61 (d, $J$ = 8.3 Hz, 1H), 4.94 (d, $J$ = 8.3 Hz, 1H), 3.54 (d, $J$ = 9.2 Hz, 1H), 3.47 – 3.40 (m, 1H), 3.38 (d, $J$ = 9.2 Hz, 1H), 3.25 – 3.18 (m, 1H), 2.43 (s, 3H), 1.76 (d, $J$ = 13.3 Hz, 1H), 1.73 – 1.67 (m, 1H), 1.56 – 1.49 (m, 1H), 1.46 (d, $J$ = 13.2 Hz, 1H), 1.18 (s, 3H), 0.88 (s, 9H). $^{13}$C NMR (101 MHz, $CDCl_3$): δ 143.9, 135.0, 129.9, 127.2, 124.4, 114.8, 88.8, 77.2, 48.4, 42.1, 41.5, 37.3, 33.0, 25.8, 22.0, 21.7. HRMS-CI (m/z): $[M + H]^+$ calcd for $C_{20}H_{30}NO_3S$, 364.1946; found, 364.1950.

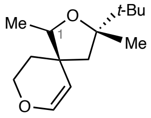


**14m**

3-(*tert*-butyl)-1,3-dimethyl-2,8-dioxaspiro[4.5]dec-6-ene (**14m**). Prepared according to general procedure E and isolated as a separable mixture of the diastereomers at C1 (1:1). Diastereomer A: $R_f$ = 0.60 (5% ethyl acetate–hexanes). Diastereomer B $R_f$ = 0.50 (5% ethyl acetate–hexanes). *Diastereomer A:* $^1$H NMR (500 MHz, $CDCl_3$): δ 6.35 (d, $J$ = 6.1 Hz, 1H), 4.49 (d, $J$ = 6.2 Hz, 1H), 4.09 – 4.04 (m, 1H), 3.88 – 3.81 (m, 2H), 1.92 – 1.85 (m, 2H), 1.65 (d, $J$ = 13.5 Hz, 1H), 1.61 –

1.57 (m, 1H), 1.23 (s, 3H), 1.14 (d, *J* = 6.4 Hz, 3H), 0.93 (s, 9H). $^{13}$C NMR (126 MHz, $CDCl_3$): δ 144.0, 108.7, 85.4, 80.0, 63.8, 51.5, 41.9, 36.6, 30.0, 26.0, 22.2, 14.8. HRMS-CI (m/z): $[M + H]^+$ calcd for $C_{14}H_{25}O_2$, 225.1855; found, 225.1855. *Diastereomer B:* $^1$H NMR (500 MHz, $CDCl_3$): δ 6.33 (d, *J* = 6.4 Hz, 1H), 4.90 (dd, *J* = 6.4, 1.7 Hz, 1H), 4.11 (dt, *J* = 10.8, 3.8 Hz, 1H), 3.94 (td, *J* = 11.4, 2.3 Hz, 1H), 3.71 (q, *J* = 6.2 Hz, 1H), 1.99 (d, *J* = 12.5 Hz, 1H), 1.89 (d, *J* = 12.6 Hz, 1H), 1.76 (m, 1H), 1.55 – 1.50 (m, 1H), 1.28 (s, 3H), 1.07 (d, *J* = 6.2 Hz, 3H), 0.92 (s, 9H). $^{13}$C NMR (126 MHz, $CDCl_3$): δ 142.5, 105.8, 85.6, 82.2, 64.3, 50.4, 42.8, 39.0, 33.2, 25.8, 25.0, 15.5. HRMS-CI (m/z): $[M + H]^+$ calcd for $C_{14}H_{25}O_2$, 225.1855; found, 225.1853.

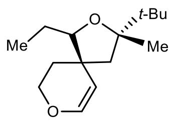


**14n**

(1S,3S,5S)-3-(*tert*-butyl)-1-ethyl-3-methyl-2,8-dioxaspiro[4.5]dec-6-ene (**14n**). $R_f$ = 0.60 (5% ethyl acetate–hexanes). Prepared according to general procedure E as an inseparable mixture of diastereomers 1.3:1. $^1$H NMR (400 MHz, $CDCl_3$): δ 6.31 (dd, *J* = 6.3, 3.2 Hz, 1H), 4.90 (dd, *J* = 6.4, 1.9 Hz, 0.44H), 4.53 (dd, *J* = 6.2, 1.8 Hz, 0.6H), 4.15 – 4.03 (m, 1H), 3.98 – 3.75 (m, 1H), 3.52 (t, *J* = 6.5 Hz, 0.6H), 3.43 (dd, *J* = 9.1, 3.1 Hz, 0.44H), 2.01 – 1.73 (m, 2H), 1.68 – 1.29 (m, 4H), 1.26 (s, 1.28H), 1.21 (s, 1.80H), 1.06 – 0.93 (m, 3H), 0.92 (d, 9H). $^{13}$C NMR (101 MHz, $CDCl_3$): δ 143.4, 142.3, 109.3, 106.2, 88.3, 85.7, 85.1, 84.9, 64.4, 63.8, 51.7, 50.8, 42.6, 41.6, 39.1, 36.6, 33.4, 29.9, 25.9, 25.8, 25.0, 24.1, 23.1, 22.1, 12.1, 11.8. HRMS-CI (m/z): $[M + H]^+$ calcd for $C_{15}H_{27}O_2$, 239.2011; found, 239.2014.

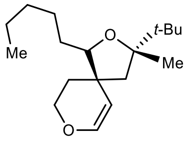


**14o**

(1S,3S,5S)-3-(*tert*-butyl)-3-methyl-1-pentyl-2,8-dioxaspiro[4.5]dec-6-ene (**14o**). $R_f$ = 0.60 (5% ethyl acetate–hexanes). Prepared according to general procedure E as an inseparable mixture of diastereomers 1.2:1. $^1$H NMR (500 MHz, $CDCl_3$): δ 6.31 (m, 1H), 4.90 (dd, *J* = 6.4, 1.9 Hz, 0.44H), 4.52 (dd, *J* = 6.3, 1.8 Hz, 0.60H), 4.14 – 4.06 (m, 1H), 3.95 – 3.80 (m, 1H), 3.59 – 3.48 (m, 1H), 1.97 – 1.77 (m, 2H), 1.62 – 1.42 (m, 4H), 1.36 – 1.27 (m, 6H), 1.23 (d, 3H), 0.91 (d, 9H), 0.90 – 0.87 (m, 3H). $^{13}$C NMR (126 MHz, $CDCl_3$): δ 143.5, 142.3, 109.2, 106.3, 86.7, 85.2, 84.9, 84.2, 64.4, 63.8, 51.6, 50.7, 42.7, 41.7, 39.1, 36.6, 33.3, 32.3, 31.0, 29.9, 29.9, 27.5, 27.0, 26.0, 25.8, 25.8, 25.0, 22.8, 22.7, 22.1, 14.3, 14.3. HRMS-CI (m/z): $[M + H]^+$ calcd for $C_{18}H_{33}O_2$, 281.2481; found, 281.2477.

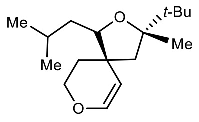

**14p**

(1S,3S,5S)-3-(*tert*-butyl)-1-isobutyl-3-methyl-2,8-dioxaspiro[4.5]dec-6-ene (**14p**). $R_f$ = 0.6 (5% ethyl acetate–hexanes). Prepared according to general procedure E as an inseparable mixture of diastereomers 1.2:1. $^1H$ NMR (400 MHz, $CDCl_3$): δ 6.32 (m, 1H), 4.89 (dd, *J* = 6.4, 2.0 Hz, 0.38H), 4.50 (dd, *J* = 6.2, 1.8 Hz, 0.63H), 4.14 – 4.04 (m, 1H), 3.96 – 3.79 (m, 1H), 3.70 – 3.55 (m, 1H), 1.97 – 1.69 (m, 3H), 1.62 – 1.55 (m, 2H), 1.39 – 1.32 (m, 1H), 1.27 – 1.18 (m, 4H), 0.94 – 0.87 (m, 15H). $^{13}C$ NMR (101 MHz, $CDCl_3$): δ 143.7, 142.3, 108.9, 106.4, 85.3, 84.9, 84.4, 82.0, 64.3, 63.8, 51.5, 50.5, 42.8, 41.9, 39.8, 39.1, 38.6, 36.6, 33.1, 29.9, 26.3, 26.0, 26.0, 25.8, 25.0, 23.9, 23.6, 22.6, 22.2, 22.0. HRMS-CI (m/z): $[M + H]^+$ calcd for $C_{17}H_{31}O_2$, 267.2324; found, 267.2326.

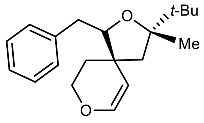

**14q**

(1S,3S,5S)-1-benzyl-3-(*tert*-butyl)-3-methyl-2,8-dioxaspiro[4.5]dec-6-ene (**14q**). $R_f$ = 0.40 (10% ethyl acetate–hexanes). Prepared according to general procedure E as an inseparable mixture of diastereomers 1.3:1. $^1H$ NMR (400 MHz, $CDCl_3$): δ 7.23 – 7.17 (m, 4H), 7.14 – 7.08 (m, 1H), 6.28 (d, *J* = 6.1 Hz, 1H), 4.44 (dd, *J* = 6.2, 1.9 Hz, 1H), 4.10 – 4.04 (m, 1H), 3.82 – 3.73 (m, 2H), 2.73 – 2.58 (m, 2H), 1.93 – 1.81 (m, 2H), 1.67 – 1.55 (m, 2H), 1.08 (s, 3H), 0.87 (s, 9H). $^{13}C$ NMR (101 MHz, $CDCl_3$): δ 144.1, 140.7, 129.3, 128.1, 125.9, 108.4, 85.4, 84.7, 63.7, 51.3, 42.4, 36.6, 36.2, 29.9, 26.0, 22.1. HRMS-CI (m/z): $[M + H]^+$ calcd for $C_{20}H_{29}O_2$, 301.2168; found, 301.2164.

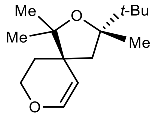

**14r**

(3S,5S)-3-(*tert*-butyl)-1,1,3-trimethyl-2,8-dioxaspiro[4.5]dec-6-ene (**14r**). $R_f$ = 0.50 (5% ethyl acetate–hexanes). Prepared according to general procedure E: $^1H$ NMR (400 MHz, $CDCl_3$): δ 6.26 (d, *J* = 6.6 Hz, 1H), 5.07 (dd, *J* = 6.6, 2.0 Hz, 1H), 4.10 – 4.04 (m, 1H), 3.98 – 3.92 (m, 1H), 2.10 (d, *J* = 12.6 Hz, 1H), 1.91 (d, *J* = 12.7 Hz, 1H), 1.89 – 1.81 (m, 1H), 1.68 – 1.62 (m, 1H), 1.34 (s, 3H), 1.21 (s, 3H), 1.14 (s, 3H), 0.92 (s, 9H). $^{13}C$ NMR (101 MHz, $CDCl_3$): δ 141.4, 108.4, 85.7, 84.4, 64.5, 49.5, 44.5, 37.7, 33.0, 27.6, 26.6, 25.5. HRMS-CI (m/z): $[M + H]^+$ calcd for $C_{15}H_{27}O_2$, 239.2011; found, 239.2012.

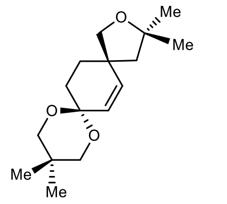

**14s**

(S)-3,3,11,11-tetramethyl-2,9,13-trioxadispiro[4.2.5$^{8.25}$]pentadec-6-ene (**14s**). R$_f$ = 0.30 (10% ethyl acetate–hexanes). Prepared according to general procedure E: $^{1}$H NMR (400 MHz, Benzene-$d_6$): δ 6.15 (d, $J$ = 10.3 Hz, 1H), 5.64 (d, $J$ = 10.3 Hz, 1H), 3.60 – 3.53 (m, 2H), 3.51 – 3.45 (m, 2H), 3.34 – 3.28 (m, 2H), 2.02 – 1.89 (m, 3H), 1.75 – 1.68 (m, 1H), 1.58 (d, $J$ = 12.9 Hz, 1H), 1.48 (d, $J$ = 12.8 Hz, 1H), 1.18 (s, 6H), 0.95 (s, 3H), 0.66 (s, 3H). $^{13}$C NMR (101 MHz, Benzene-$d_6$): δ 137.5, 124.6, 95.2, 80.7, 75.9, 70.6, 70.5, 52.4, 46.9, 32.0, 31.3, 30.1, 29.3, 29.1, 23.0, 22.4. HRMS-CI (m/z): [M + H]$^+$ calcd for $C_{16}H_{27}O_3$, 267.1960; found, 267.1950.

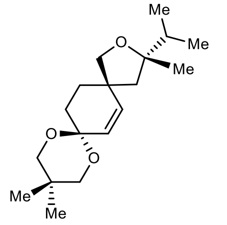

**14t**

(3S,5S)-3-isopropyl-3,11,11-trimethyl-2,9,13-trioxadispiro[4.2.5$^{8.25}$]pentadec-6-ene (**14t**). R$_f$ = 0.40 (10% ethyl acetate–hexanes). Prepared according to general procedure E: $^{1}$H NMR (400 MHz, Benzene-$d_6$): δ 6.15 (d, $J$ = 10.3 Hz, 1H), 5.68 (d, $J$ = 10.3 Hz, 1H), 3.55 (s, 2H), 3.51 – 3.46 (m, 2H), 3.34 – 3.29 (m, 2H), 2.05 – 1.84 (m, 3H), 1.76 – 1.66 (m, 2H), 1.59 (d, $J$ = 13.0 Hz, 1H), 1.47 (d, $J$ = 13.0 Hz, 1H), 1.05 (s, 3H), 0.98 (d, $J$ = 6.7 Hz, 3H), 0.95 (s, 3H), 0.77 (d, $J$ = 6.9 Hz, 3H), 0.68 (s, 3H). $^{13}$C NMR (101 MHz, Benzene-$d_6$): δ 137.8, 124.6, 95.2, 86.0, 75.6, 70.7, 70.5, 50.1, 46.2, 38.1, 31.9, 31.8, 30.1, 23.0, 22.5, 21.3, 18.5, 17.7. HRMS-CI (m/z): [M + H]$^+$ calcd for $C_{18}H_{31}O_3$, 295.2273; found, 295.2271.

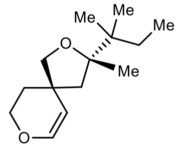

**14u**

(3S,5S)-3-methyl-3-(*tert*-pentyl)-2,8-dioxaspiro[4.5]dec-6-ene (**14u**). R$_f$ = 0.50 (5% ethyl acetate–hexanes). Prepared according to general procedure E: $^{1}$H NMR (400 MHz, $CDCl_3$): δ 6.33 (d, $J$ = 6.2 Hz, 1H), 4.66 (d, $J$ = 6.2 Hz, 1H), 4.04 – 3.98 (m, 1H), 3.91 – 3.85 (m, 1H), 3.65 (dd, $J$ = 9.1, 0.9 Hz, 1H), 3.56 (d, $J$ = 9.1 Hz, 1H), 1.95 – 1.86 (m, 2H), 1.76 – 1.69 (m, 1H), 1.57 (d, $J$ = 13.2 Hz, 1H), 1.41 – 1.30 (m, 2H), 1.22 (s, 3H), 0.88 – 0.83 (m, 9H). $^{13}$C NMR (101 MHz, $CDCl_3$): δ

143.6, 108.3, 89.6, 77.6, 64.4, 49.2, 40.0, 39.9, 35.3, 29.6, 21.8, 21.4, 21.2, 9.0. HRMS-CI (m/z): $[M + H]^+$ calcd for $C_{14}H_{25}O_2$, 225.1855; found, 225.1854.

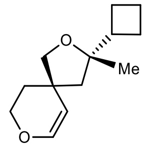

**14v**

(3S,5S)-3-cyclobutyl-3-methyl-2,8-dioxaspiro[4.5]dec-6-ene (**14v**). $R_f$ = 0.40 (5% ethyl acetate–hexanes). Prepared according to general procedure E: $^1H$ NMR (400 MHz, $CDCl_3$): δ 6.34 (d, $J$ = 6.2 Hz, 1H), 4.63 (d, $J$ = 6.2 Hz, 1H), 4.02 – 3.96 (m, 1H), 3.93 – 3.87 (m, 1H), 3.63 – 3.56 (m, 2H), 2.49 – 2.40 (m, 1H), 1.93 – 1.67 (m, 8H), 1.63 (s, 2H), 1.15 (s, 3H). $^{13}C$ NMR (101 MHz, $CDCl_3$): δ 143.9, 107.6, 84.3, 77.4, 64.3, 49.8, 45.0, 40.6, 34.8, 23.8, 23.6, 23.1, 17.0. HRMS-CI (m/z): $[M + H]^+$ calcd for $C_{13}H_{21}O_2$, 209.1542; found, 209.1540.

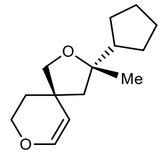

**14w**

(3S,5S)-3-cyclopentyl-3-methyl-2,8-dioxaspiro[4.5]dec-6-ene (**14w**). $R_f$ = 0.40 (5% ethyl acetate–hexanes). Prepared according to general procedure E: $^1H$ NMR (400 MHz, $CDCl_3$): δ 6.34 (d, $J$ = 6.2 Hz, 1H), 4.62 (d, $J$ = 6.2 Hz, 1H), 4.04 – 3.97 (m, 1H), 3.92 – 3.86 (m, 1H), 3.63 – 3.55 (m, 2H), 2.12 – 2.01 (m, 1H), 1.97 – 1.89 (m, 1H), 1.77 – 1.66 (m, 5H), 1.62 – 1.51 (m, 4H), 1.35 – 1.25 (m, 2H), 1.21 (s, 3H). $^{13}C$ NMR (101 MHz, $CDCl_3$): δ 143.9, 107.7, 86.0, 77.1, 64.3, 51.2, 50.2, 40.7, 35.1, 28.3, 28.1, 26.0, 26.0, 24.1. HRMS-CI (m/z): $[M + H]^+$ calcd for $C_{14}H_{23}O_2$, 223.1698; found, 223.1700.

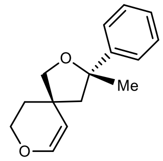

**14x**

(3S,5S)-3-methyl-3-phenyl-2,8-dioxaspiro[4.5]dec-6-ene (**14x**). $R_f$ = 0.40 (5% ethyl acetate–hexanes). Prepared according to general procedure E: $^1H$ NMR (400 MHz, $CDCl_3$): δ 7.43 – 7.38 (m, 2H), 7.35 – 7.29 (m, 2H), 7.24 – 7.18 (m, 1H), 6.37 (d, $J$ = 6.2 Hz, 1H), 4.64 (d, $J$ = 6.2 Hz, 1H), 3.90 – 3.84 (m, 1H), 3.82 – 3.76 (m, 2H), 3.71 (d, $J$ = 8.8 Hz, 1H), 2.25 (d, $J$ = 12.8 Hz, 1H), 2.15 (d, $J$ = 12.8 Hz, 1H), 1.67 – 1.60 (m, 1H), 1.54 (s, 3H), 1.47 – 1.40 (m, 1H). $^{13}C$ NMR (101 MHz, $CDCl_3$): δ 148.9, 144.4, 128.3, 126.4, 124.6, 106.9, 84.8, 78.1, 64.1, 54.9, 41.3, 33.9, 31.7. HRMS-CI (m/z): $[M + H]^+$ calcd for $C_{15}H_{19}O_2$, 231.1385; found, 231.1385.

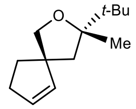

**14z**

(3S,5S)-3-(*tert*-butyl)-3-methyl-2-oxaspiro[4.4]non-6-ene (**14z**). $R_f$ = 0.60 (5% ethyl acetate–hexanes). Prepared according to general procedure E: $^1$H NMR (400 MHz, $CDCl_3$): δ 5.73 – 5.68 (m, 2H), 3.62 (s, 2H), 2.34 – 2.28 (m, 2H), 2.05 – 1.99 (m, 2H), 1.80 – 1.74 (m, 1H), 1.68 (d, *J* = 13.2 Hz, 1H), 1.20 (s, 3H), 0.95 (s, 9H). $^{13}$C NMR (101 MHz, $CDCl_3$): δ 137.5, 130.5, 88.7, 76.8, 57.0, 47.7, 38.3, 37.7, 31.5, 25.9, 22.4. HRMS-CI (m/z): $[M + H]^+$ calcd for $C_{13}H_{23}O$, 195.1749; found, 195.1765.

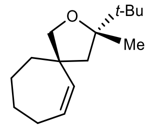

**14aa**

(3S,5S)-3-(*tert*-butyl)-3-methyl-2-oxaspiro[4.6]undec-6-ene (**14aa**). $R_f$ = 0.60 (5% ethyl acetate–hexanes). Prepared according to general procedure E: $^1$H NMR (400 MHz, $CDCl_3$): δ 5.80 – 5.75 (m, 1H), 5.71 – 5.64 (m, 1H), 3.77 (d, *J* = 8.4 Hz, 1H), 3.52 (d, *J* = 8.4 Hz, 1H), 2.20 – 2.08 (m, 2H), 2.04 (d, *J* = 12.7 Hz, 1H), 1.87 – 1.79 (m, 1H), 1.73 – 1.64 (m, 3H), 1.59 – 1.54 (m, 2H), 1.48 – 1.39 (m, 1H), 1.21 (s, 3H), 0.93 (s, 9H). $^{13}$C NMR (101 MHz, $CDCl_3$): δ 141.1, 130.1, 88.7, 81.5, 49.5, 45.8, 38.2, 36.6, 28.6, 28.0, 27.4, 25.8, 23.0. HRMS-CI (m/z): $[M + H]^+$ calcd for $C_{15}H_{27}O$, 223.2062; found, 223.2059.

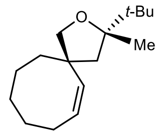

**14ab**

(3S,5S,Z)-3-(*tert*-butyl)-3-methyl-2-oxaspiro[4.7]dodec-6-ene (**14ab**). $R_f$ = 0.60 (5% ethyl acetate–hexanes). Prepared according to general procedure E: $^1$H NMR (400 MHz, $CDCl_3$): δ 5.81 – 5.73 (m, 1H), 5.62 – 5.53 (m, 1H), 3.61 (d, *J* = 8.7 Hz, 1H), 3.52 (d, *J* = 8.8 Hz, 1H), 2.26 – 2.19 (m, 1H), 2.17 – 2.09 (m, 3H), 1.90 (d, *J* = 13.0 Hz, 1H), 1.60 – 1.49 (m, 6H), 1.36 (d, *J* = 13.1 Hz, 1H), 1.18 (s, 3H), 0.95 (s, 9H). $^{13}$C NMR (101 MHz, $CDCl_3$): δ 132.5, 128.4, 88.4, 77.9, 49.2, 45.7, 37.8, 37.2, 35.4, 30.1, 26.7, 25.9, 24.6, 22.6. HRMS-CI (m/z): $[M + H]^+$ calcd for $C_{16}H_{29}O$, 237.2218; found, 237.2214.

**Crystallographic Analysis of 14j and 32.**

**Crystallographic Analysis for the Spirocyclic Ether 14j.**

Single crystals of the ether **14j** suitable for the X-ray analysis were obtained by the slow evaporation of a solution of compound **14j** in hexane–ethyl acetate (4:1 v/v) at 23 °C. Crystals were mounted on a MiTeGen MicroMount with Type B immersion oil (Cargille Labs). Single crystal X-ray intensity data were measured on a Bruker D8 SMART APEXII "three-circle diffractometer" system equipped with a Incoatec "microfocus sealed X-ray tube" (MoK α radiation, λ = 0.71073 Å), a multilayer optics monochromator and a PHOTON-II-C14 detector. Crystal temperature was controlled by an Oxford Cryosystems 700 + Cooler. Full datasets were collected with ω scans at T = 100(2) K. The frames were integrated with the Bruker SAINT software package using a narrow-frame algorithm and the data were corrected for absorption effects using the Multi-Scan method with the SADABS software. The structures were solved by intrinsic phasing methods (SHELXT) and the structure models were completed and refined using the full-matrix least-square methods on F2 (SHELXL). All non-hydrogen atoms were refined with anisotropic displacement parameters, and hydrogen atoms on carbons were placed in idealized positions (C-H = 0.95-1.00 Å) and included using a riding model with Uiso(H) = 1.2 or 1.5 Ueq(non-H). Selected crystallographic parameters are listed in **Table S3**. Crystallographic data of these structures, including cif, res, fcf, and hkl files, have been deposited with the Cambridge Crystallographic Data Centre with deposition numbers 2376833. Copies of these data can be requested, free of charge, from the CCDC website at https://www.ccdc.cam.ac.uk/structures/.

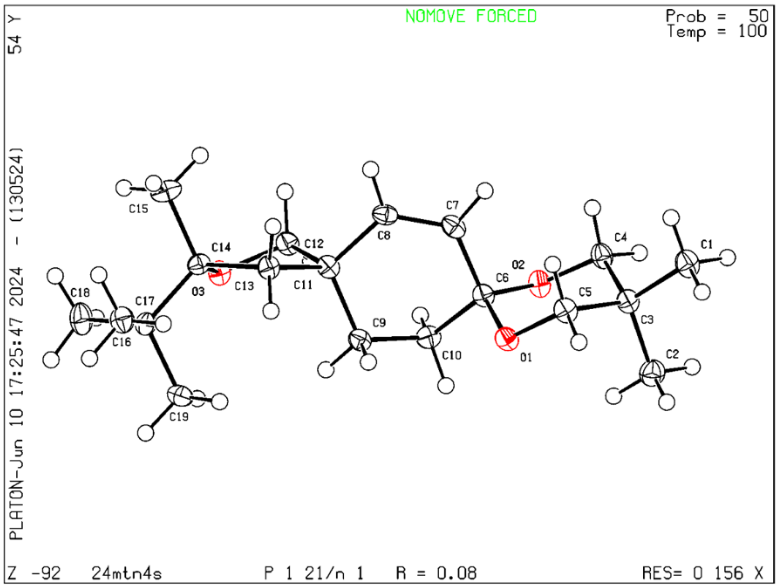


**Figure S1.** The crystal structure of **14j**.

**Table S3**. Crystallographic data and structure refinement of the ether **14j:**

Identification code: 2376833

| | | | |
|---|---|---|---|
| Bond precision: | C–C = 0.0031 Å | | Wavelength=0.71073 |
| Cell: | a = 5.8889(6) Å | b = 51.122(5) Å | c = 6.0851(6) Å |
| | α =90° | β = 108.790(5) ° | γ = 90° |
| Temperature: | 100 K | | |

| | Calculated | Reported |
|---|---|---|
| Volume | 1734.3(3) Å$^3$ | 1734.3(3) |
| Space group | P 21/n | P 1 21/n 1 |
| Hall group | –P 2yn | –P 2yn |
| Moiety formula | C19 H32 O3 | |
| Sum formula | C19 H32 O3 | C19 H32 O3 |
| Mr | 308.45 | 308.44 |
| Dx,g cm-3 | 1.181 | 1.181 |
| Z | 4 | 4 |
| Mu (mm-1) | 0.077 | 0.077 |
| F000 | 680.0 | 680.0 |
| F000' | 680.30 | |
| h,k,lmax | 7,68,8 | 7,68,8 |
| Nref | 4464 | 4380 |
| Tmin,Tmax | 0.989,0.995 | 0.800,0.990 |
| Tmin' | 0.961 | |

Correction method = # Reported T Limits: Tmin=0.800 Tmax=0.990
AbsCorr = MULTI-SCAN

Data completeness = 0.981 Theta(max) = 28.660

R(reflections) = 0.0781( 3704) wR2(reflections) = 0.1680( 4380)

S = 1.165 Npar= 205

## Crystallographic Analysis for the Carboxylic Acid 32.

Single crystals of the ether **32** suitable for the X-ray analysis were obtained by the slow evaporation of a solution of compound **32** in hexane–ethyl acetate (1:1 v/v) at 23 °C. Crystals were mounted on a MiTeGen MicroMount with Type B immersion oil (Cargille Labs). Single crystal X-ray intensity data were measured on a Bruker D8 SMART APEXII "three-circle diffractometer" system equipped with a Incoatec "microfocus sealed X-ray tube" (MoK α radiation, λ = 0.71073 Å), a multilayer optics monochromator and a PHOTON-II-C14 detector. Crystal temperature was controlled by an Oxford Cryosystems 700 + Cooler. Full datasets were collected with ω scans at T = 100(2) K. The frames were integrated with the Bruker SAINT software package using a narrow-frame algorithm and the data were corrected for absorption effects using the Multi-Scan method with the SADABS software. The structures were solved by intrinsic phasing methods (SHELXT) and the structure models were completed and refined using the full-matrix least-square methods on F2 (SHELXL). All non-hydrogen atoms were refined with anisotropic displacement parameters, and hydrogen atoms on carbons were placed in idealized positions (C-H = 0.95-1.00 Å) and included using a riding model with Uiso(H) = 1.2 or 1.5 Ueq(non-H). Selected crystallographic parameters are listed in **Table S4**. Crystallographic data of these structures, including cif, res, fcf, and hkl files, have been deposited with the Cambridge Crystallographic Data Centre with deposition numbers 2376834. Copies of these data can be requested, free of charge, from the CCDC website at https://www.ccdc.cam.ac.uk/structures/.

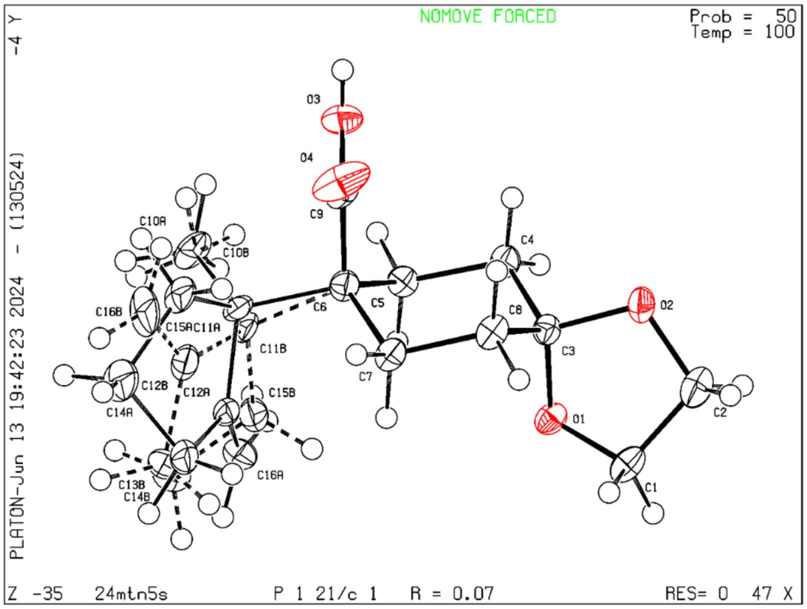


**Figure S2.** The crystal structure of **32**.

**Table S4**. Crystallographic data and structure refinement of the carboxylic acid **32**:

Identification code: 2376834

| | | | |
|---|---|---|---|
| Bond precision: | C–C = 0.0033 Å | | Wavelength=0.71073 |
| Cell: | a = 14.8594(16) Å | b = 7.1857(7) Å | c = 14.9356(15) Å |
| | α = 90° | β = 114.073(6) ° | γ= 90° |
| Temperature: | 100 K | | |

| | Calculated | Reported |
|---|---|---|
| Volume | 1456.1(3) Å$^3$ | 1456.1(3) |
| Space group | P 21/c | P 1 21/c 1 |
| Hall group | –P 2ybc | –P 2ybc |
| Moiety formula | C16 H24 O4 | |
| Sum formula | C16 H24 O4 | C16 H24 O4 |
| Mr | 280.35 | 280.35 |
| Dx,g cm-3 | 1.279 | 1.279 |
| Z | 4 | 4 |
| Mu (mm-1) | 0.090 | 0.090 |
| F000 | 608.0 | 608.0 |
| F000' | 608.32 | |
| h,k,lmax | 19,9,20 | 19,9,20 |
| Nref | 3709 | 3692 |
| Tmin,Tmax | 0.969,0.999 | 0.920,1.000 |
| Tmin' | 0.953 | |

Correction method= # Reported T Limits: Tmin=0.920 Tmax=1.000
AbsCorr = MULTI-SCAN

Data completeness= 0.995 Theta(max)= 28.560

R(reflections)= 0.0728( 2787) wR2(reflections)= 0.1772( 3692)

S = 1.110 Npar= 248

## References.


[1] He, J.; Xue, Y.; Han, B.; Zhang, C.; Wang, Y.; Zhu, S. Nickel-Catalyzed Asymmetric Reductive 1,2-Carboamination of Unactivated Alkenes. *Angew. Chem., Int. Ed.* **2020**, 59 (6), 2328−2332.

[2] Chiba, J.; Muro, F.; Setoguchi, M.; Machinaga, N. A Concise Synthesis of a Very Late Antigen-4 Antagonist trans-4-[1-[[2,5-Dichloro-4-(1-methyl-3-indolylcarboxyamide)phenyl]acetyl]-(4S)-methoxy-(2S)-pyrrolidinylmethoxy]cyclohexanecarboxylic Acid via Reductive Etherification. *Chem. Pharm. Bull.* **2012**, 60, 882–886.

[3] Kobayashi, K.; Takahashi, H.; Kawamoto, H.; Kato, T.; Itoh, S.; Yoshizumi, T.; Okamato, O. Benzimidazole Derivatives. US7125877 B2, October 24, 2006.

[4] Faulkner, A.; Scott, J. S.; Bower, J. F. An Umpolung Approach to Alkene Carboamination: Palladium Catalyzed 1,2-Amino-Acylation, -Carboxylation, -Arylation, -Vinylation, and –Alkynylation *J. Am. Chem. Soc.* **2015**, 137, 7224−7230.

[5] Wei, W.-X.; Li, Y.; Wen, Y.-T.; Li, M.; Li, X.-S.; Wang, C.-T.; Liu, H.-C.; Xia,Y.; Zhang, B.-S.; Jiao, R.-Q.; Liang, Y.-M. Experimental and Computational Studies of Palladium-Catalyzed Spirocyclization via a Narasaka−Heck/C($sp^3$ or $sp^2$)−H Activation Cascade Reaction. *J. Am. Chem. Soc.* **2021**, 143, 7868−78875.

[6] Wu, L.; Wang, M.; Liang, Y.; Shi, Z. Ligand-Controlled Palladium-Catalyzed Regiodivergent Defluorinative Allylation of gem-Difluorocyclopropanes via *σ*-Bond Activation. *Chin. J. Chem.* **2022**, 40, 2345−2355.

[7] Budai, B.; Leclair, A.; Wang, Q.; Zhu, J. Copper-Catalyzed 1,2-Methoxy Methoxycarbonylation of Alkenes with Methyl Formate. *Angew. Chem., Int. Ed.* **2019**, 58, 10305.

[8] Meng, G.; Wang, Z.; Chan, H.S.S.; Chekshin, N.; Li, Z.; Wang, P.; Yu, J.-Q. Dual-Ligand Catalyst for the Nondirected C–H Olefination of Heteroarens. *J. Am. Chem. Soc.* **2023**, 145, 14, 8198-8208.

[9] Mitchell, J. K.; Hussain, W. A.; Bansode, A. H.; O'Connor, R. M.; Parasram, M. Aziridination via Nitrogen-Atom Transfer to Olefins from Photoexcited Azoxy-Triazenes. *J. Am. Chem. Soc.* **2024**, 146, 9499−9505.

[10] Padwa, A.; Dimitroff, M.; Liu, B. Formal Synthesis of (±)-Dendrobine: Use of the Amidofuran Cycloaddition/ Rearrangement Sequence. *Org. Lett.* **2000**, 2, 3233−3235.

[11] Short, R.P; Revol, J.-P.; Ranu, B.C.; Hudlicky, T. General method of synthesis of cyclopentanoid terpenic acids. Stereocontrolled total synthesis (±)-isocomenic acid and (±)-epiiosocomenic acid. *J. Org. Chem.* **1983**, 48, 24, 4453-4461.

[12] Zhang, Y.; Qian, J.; Wang, M.; Huang, Y.; Hu, P. Visible-Light-Induced Decarboxylative Fluorination of Aliphatic Carboxylic Acids Catalyzed by Iron. *Org. Lett.* **2022**, 24, 5972−5976.

[13] Wang, Z.; Hu, L.; Chekshin, N.; Zhuang, Z.; Qian, S.; Qiao, J. X.; Yu, J.-Q. Ligand-controlled divergent dehydrogenative reactions of carboxylic acids via C–H activation. *Science* **2021**, 374, 1281-1285.

[14] Wang, Z.; Hu, L.; Yu, J.-Q. Ligand-controlled Divergent Dehydrogenative Reactions of Aliphatic Acids, U.S. Patent WO-2023287964-A1, July 14, 2022.

**Catalogue of $^{1}$H NMR and $^{13}$C NMR Spectra.**

$^{1}$H NMR (400 MHz, $C_6D_6$)

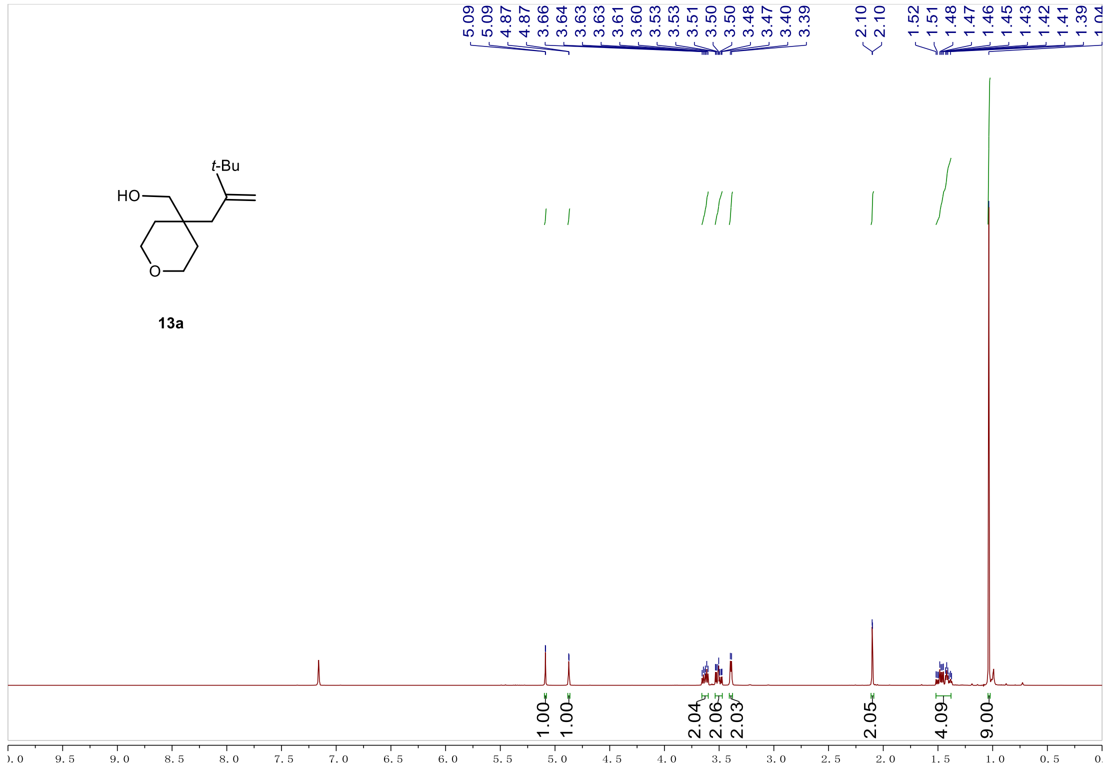


$^{13}$C NMR (101 MHz, $C_6D_6$)

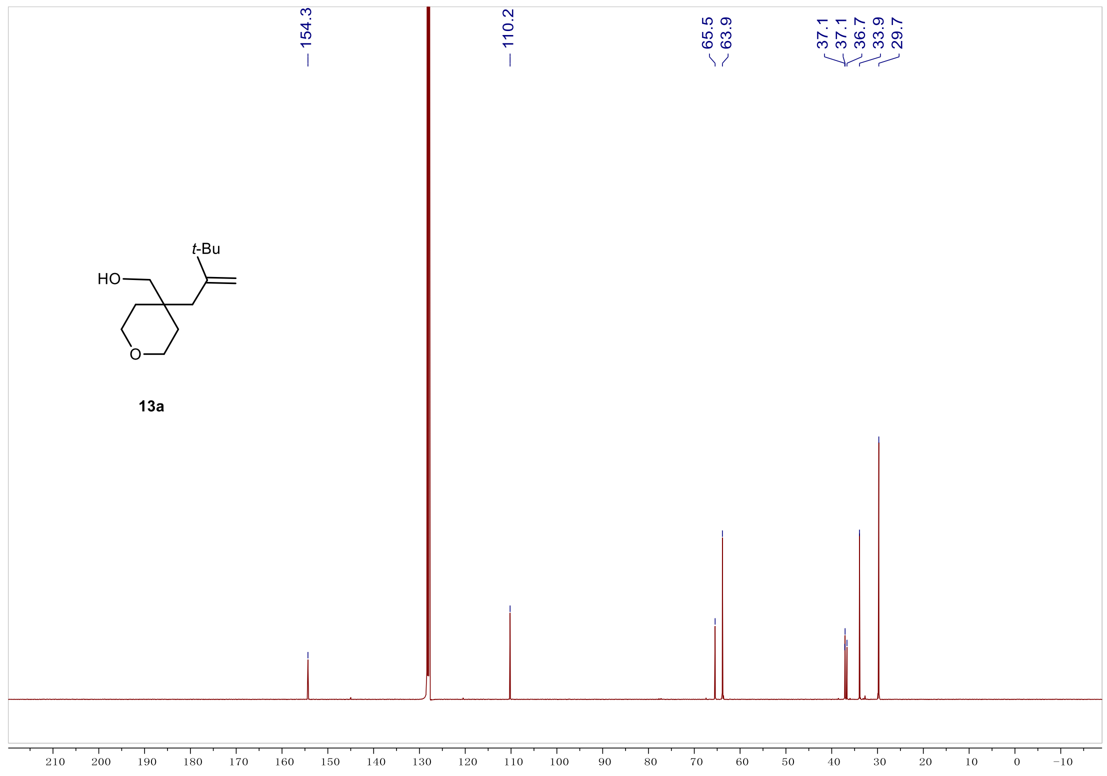

$^{1}H$ NMR (400 MHz, $C_6D_6$)

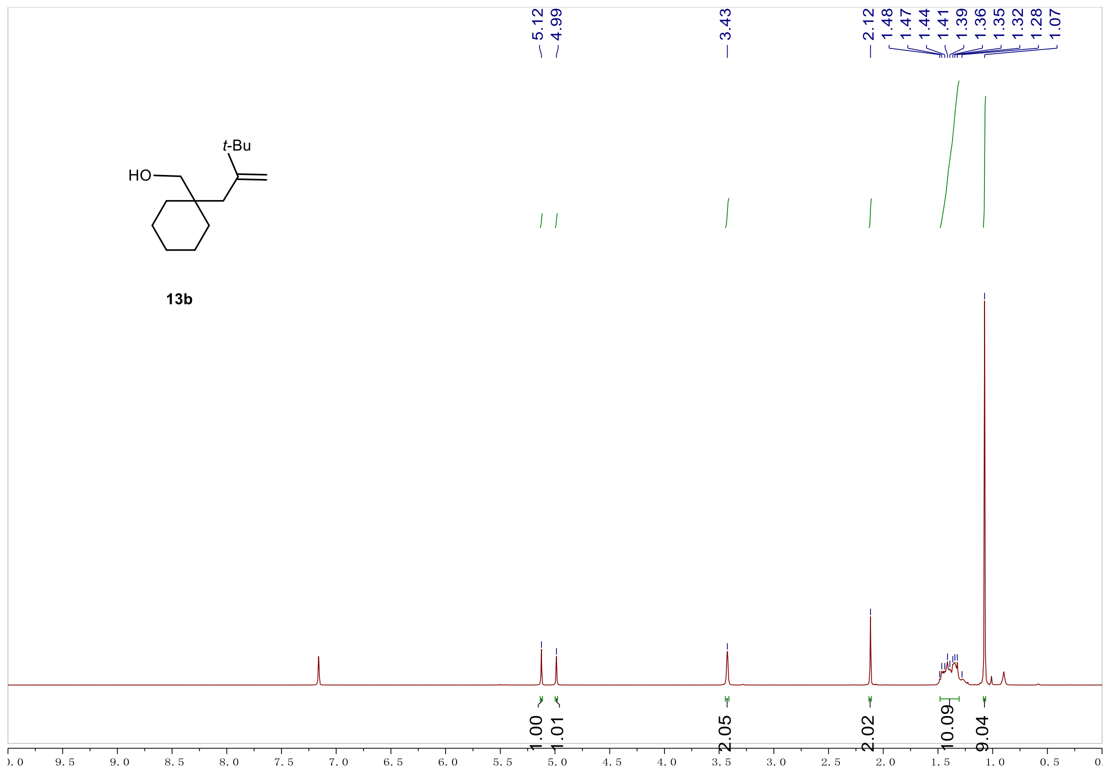


$^{13}C$ NMR (101 MHz, $C_6D_6$)

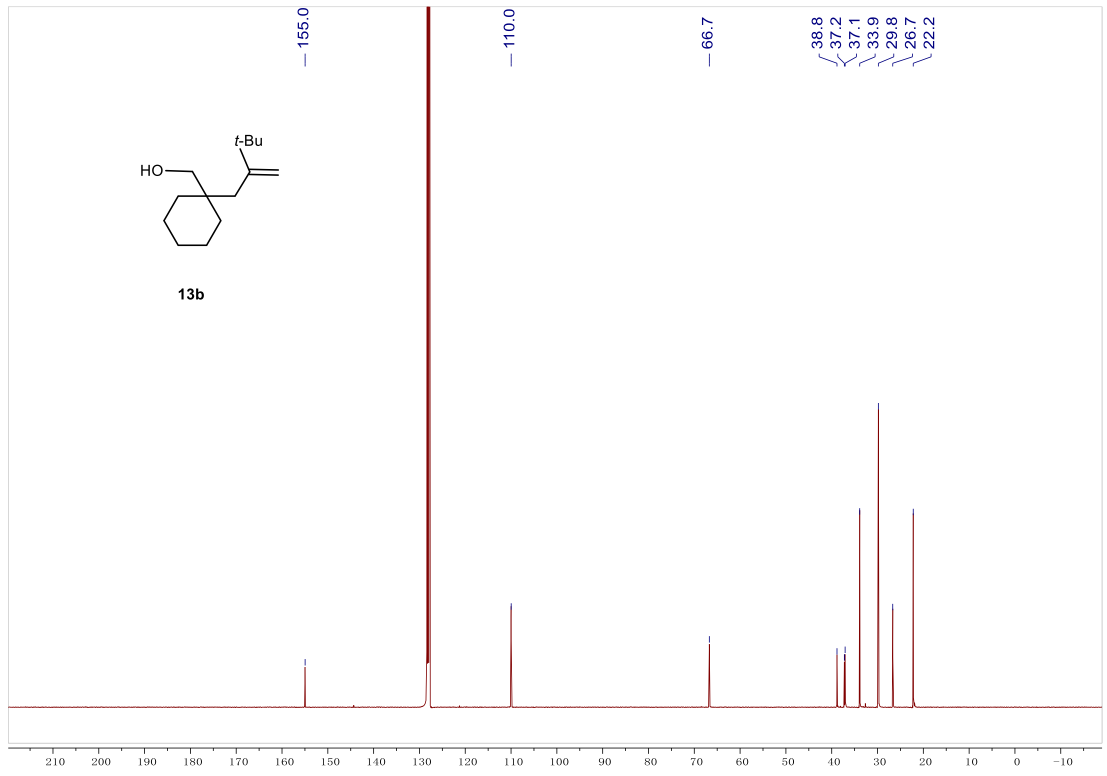

<sup></sup>

$^{1}$H NMR (400 MHz, $C_6D_6$)

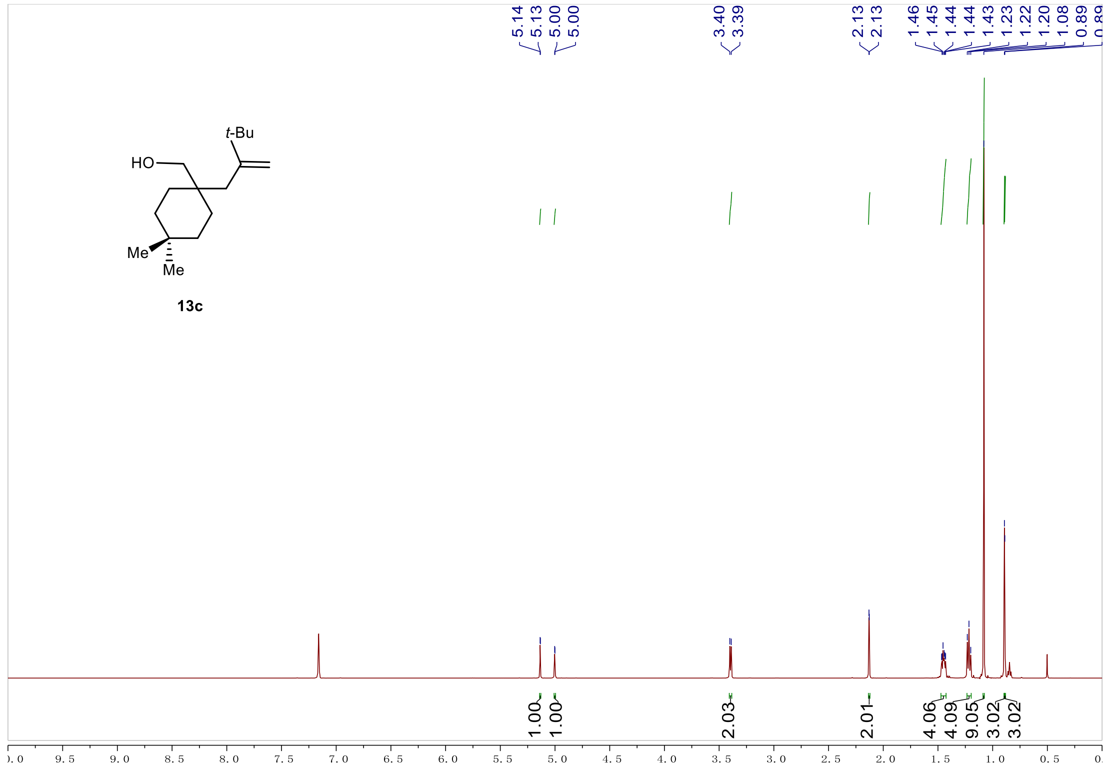


$^{13}$C NMR (101 MHz, $C_6D_6$)

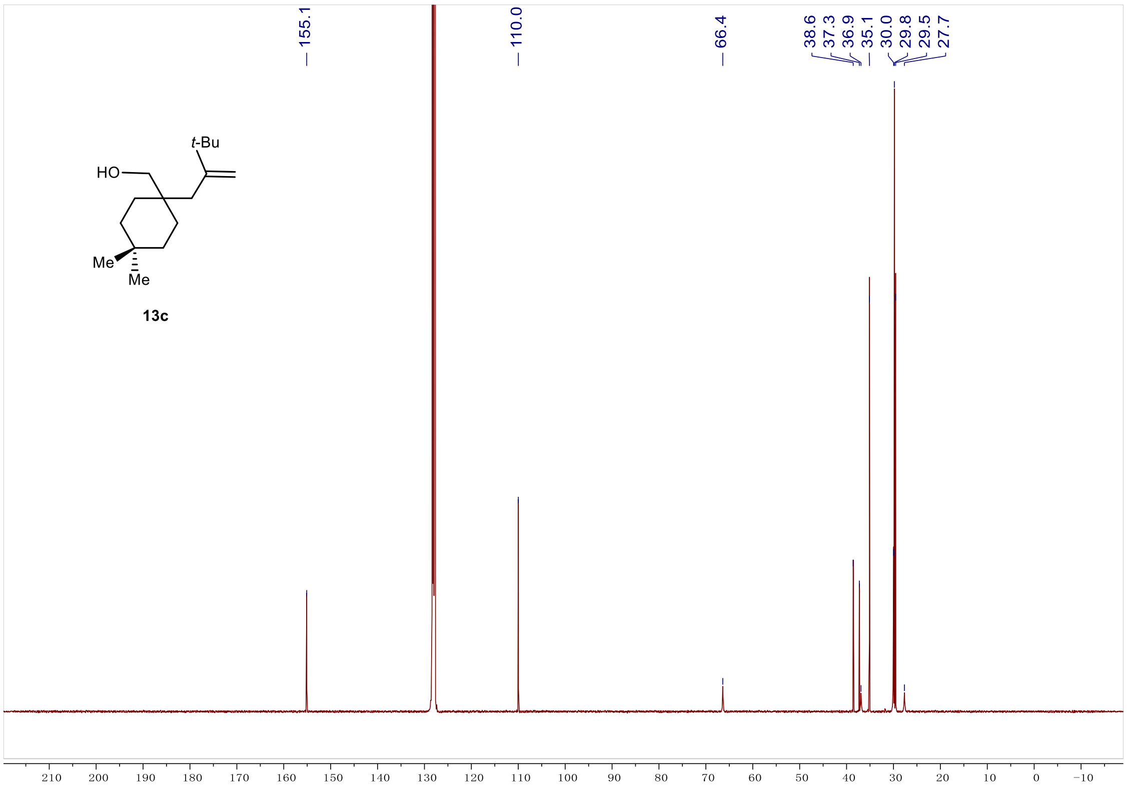

$^{1}$H NMR (400 MHz, $C_6D_6$)

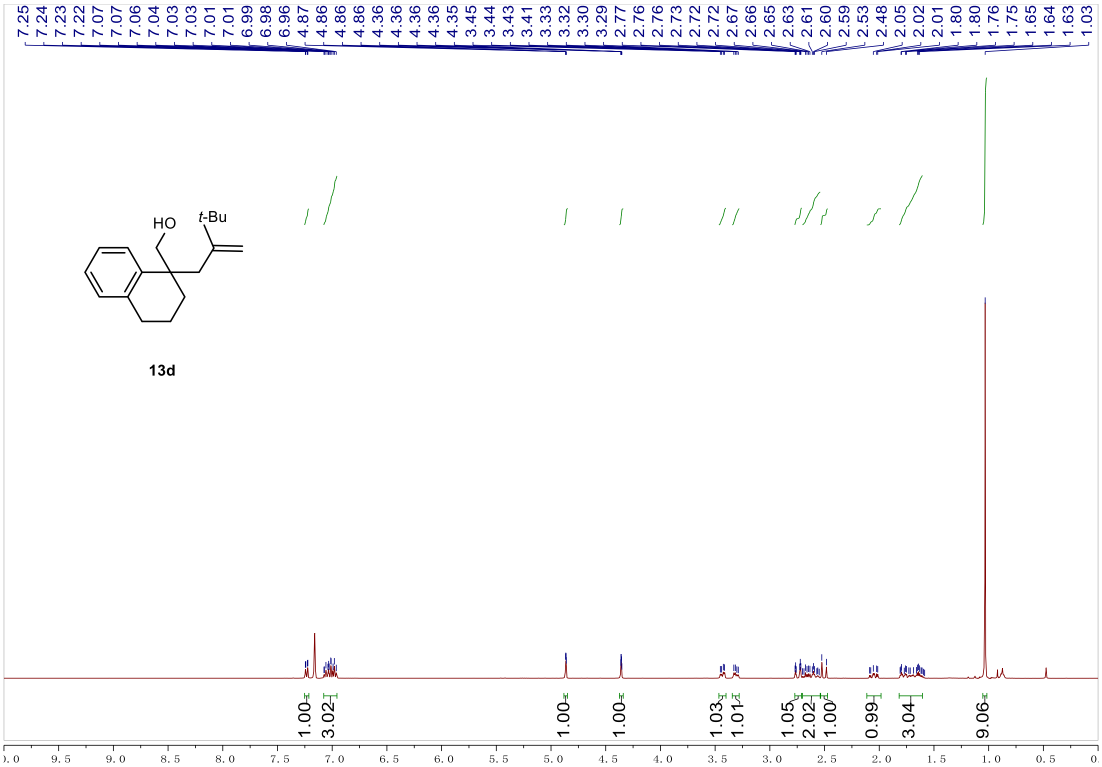

$^{13}$C NMR (101 MHz, $C_6D_6$)

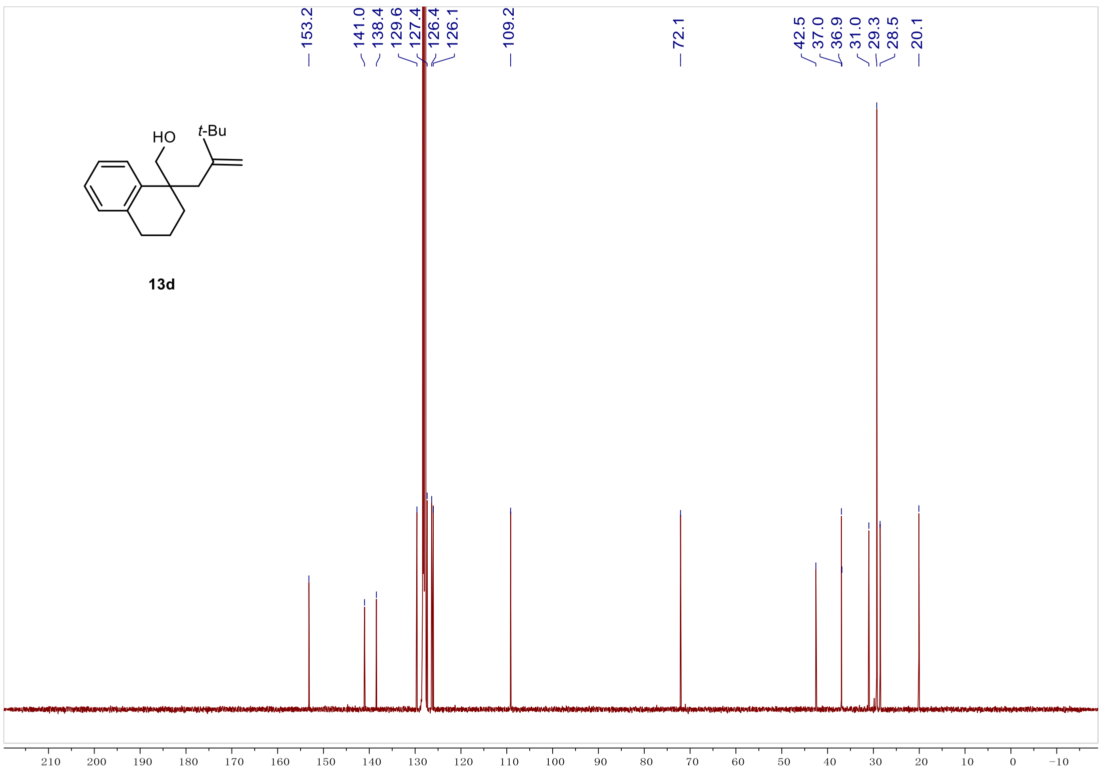

$^{1}H$ NMR (400 MHz, $C_6D_6$)

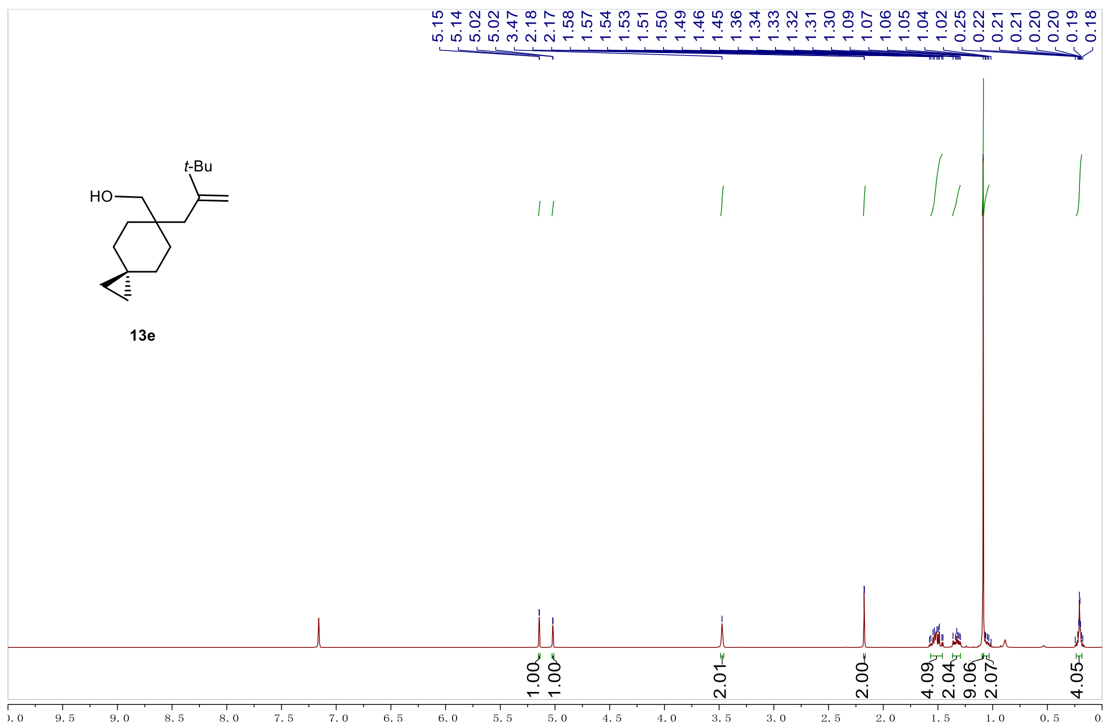


$^{13}C$ NMR (101 MHz, $C_6D_6$)

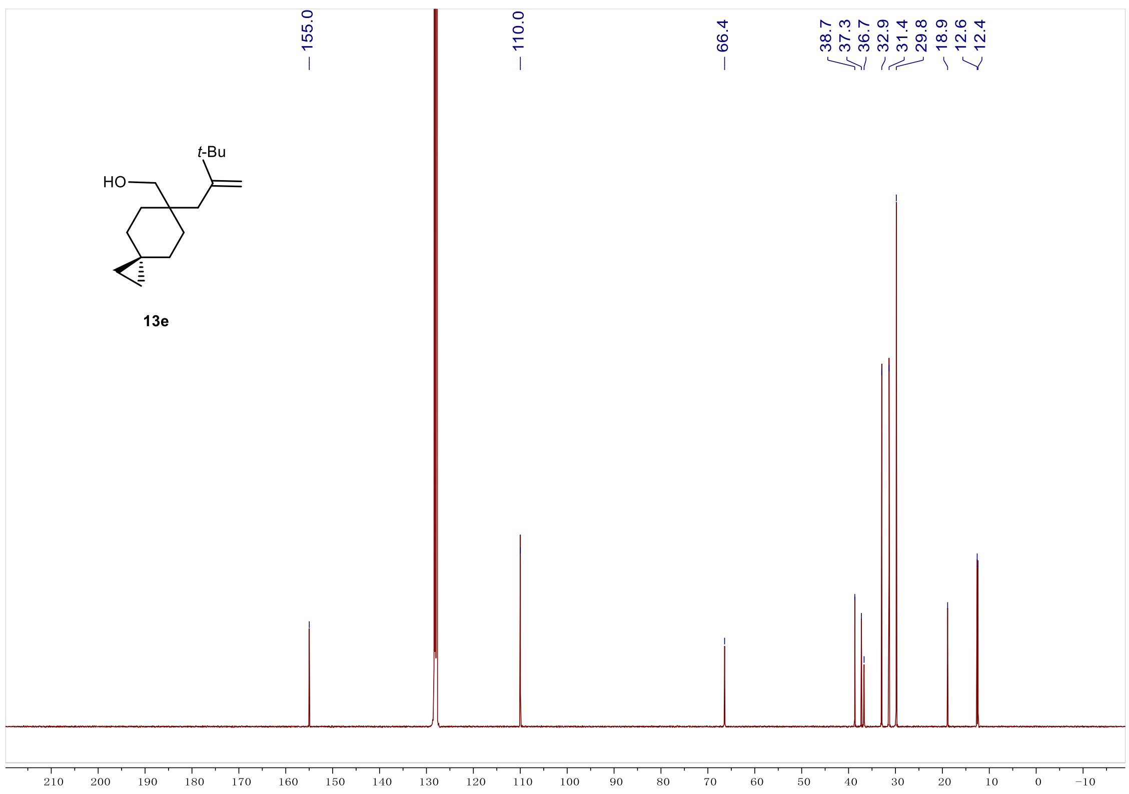

$^{1}H$ NMR (400 MHz, $CDCl_3$)

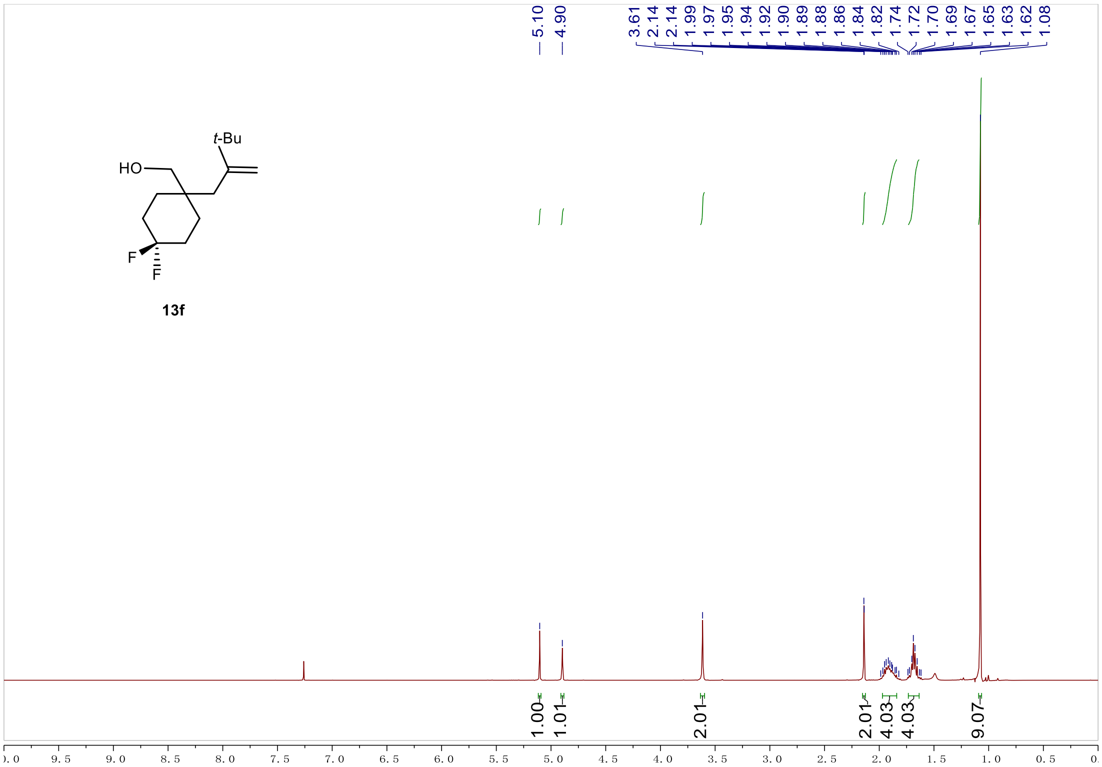


$^{13}C$ NMR (101 MHz, $CDCl_3$)

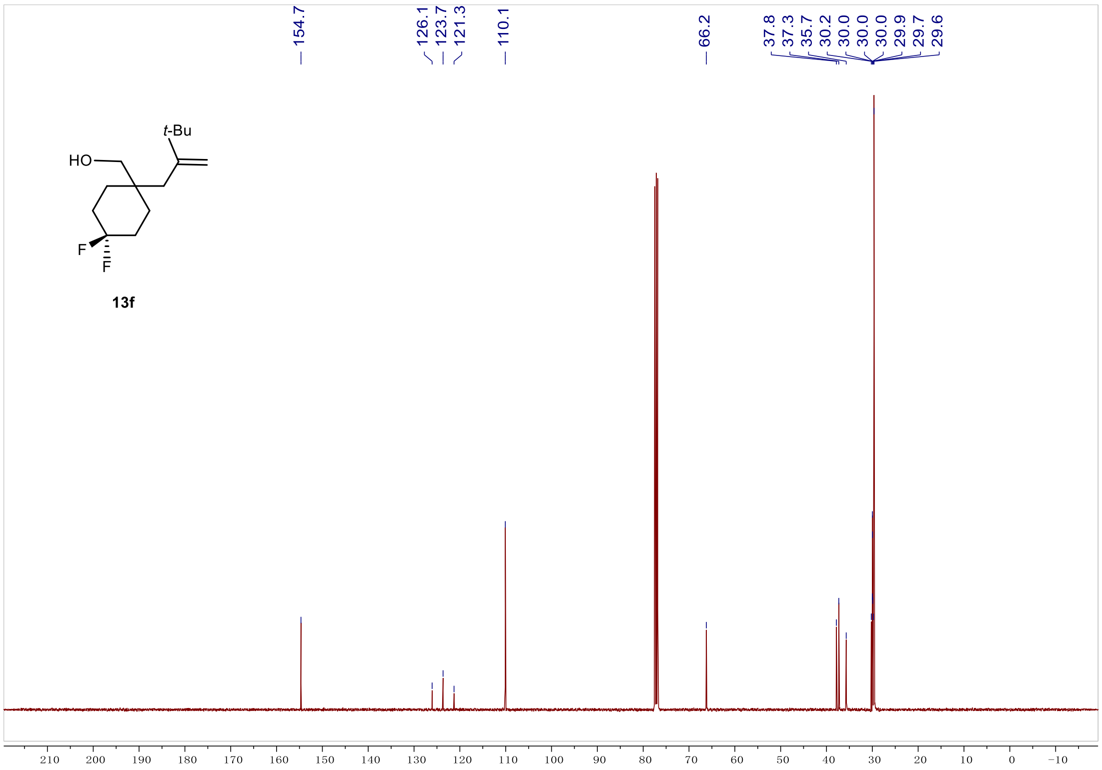

$^{19}F$ NMR (471 MHz, $C_6D_6$)

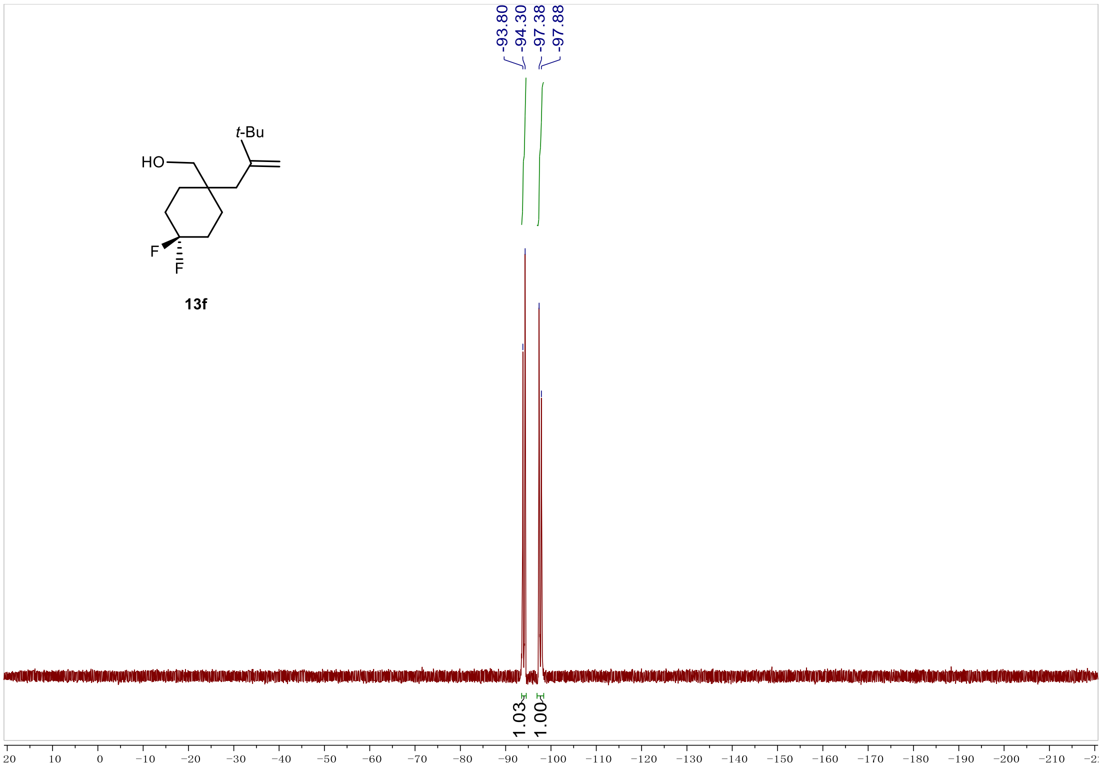

$^1H$ NMR (400 MHz, $C_6D_6$)

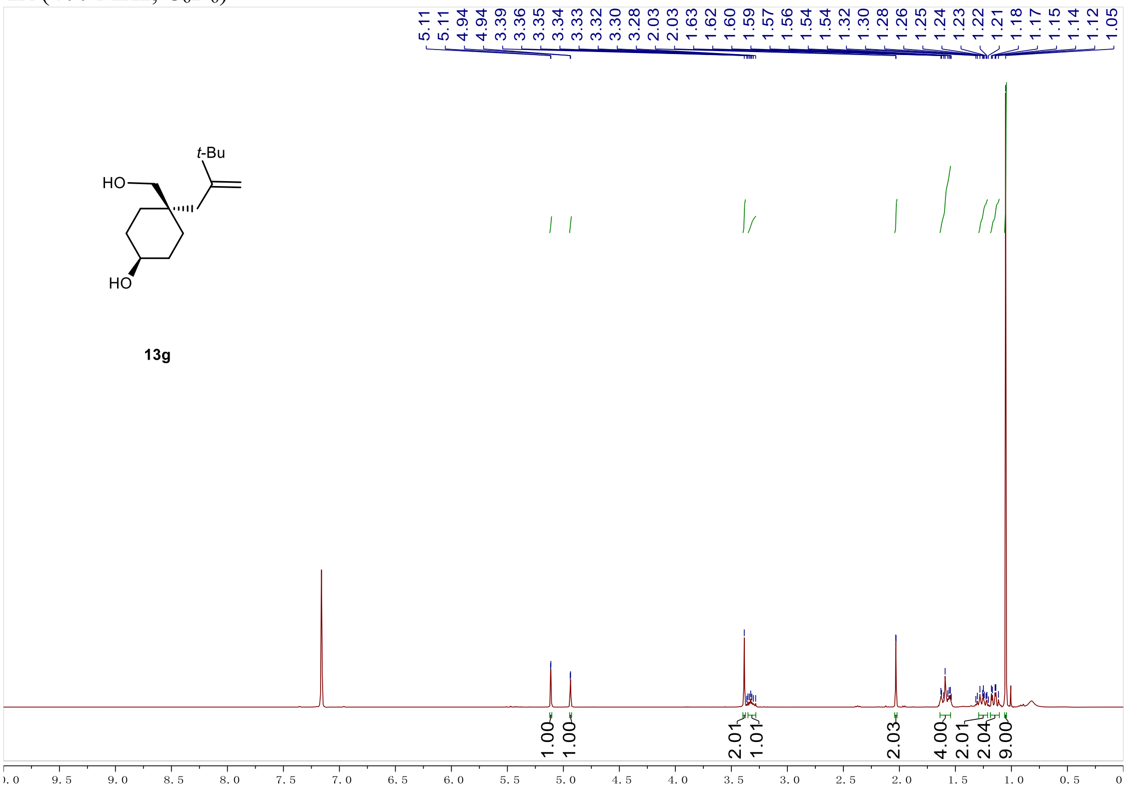


$^{13}C$ NMR (101 MHz, $C_6D_6$)

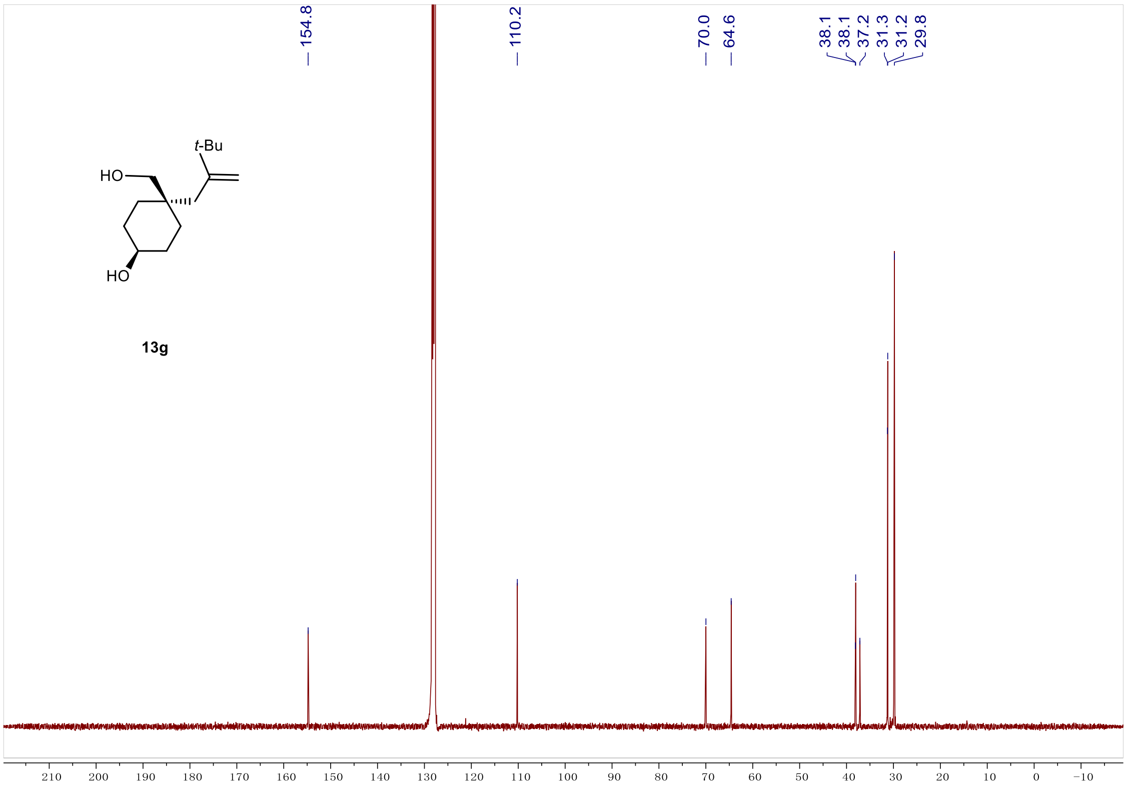

NOESY NMR (600 MHz, $C_6D_6$)

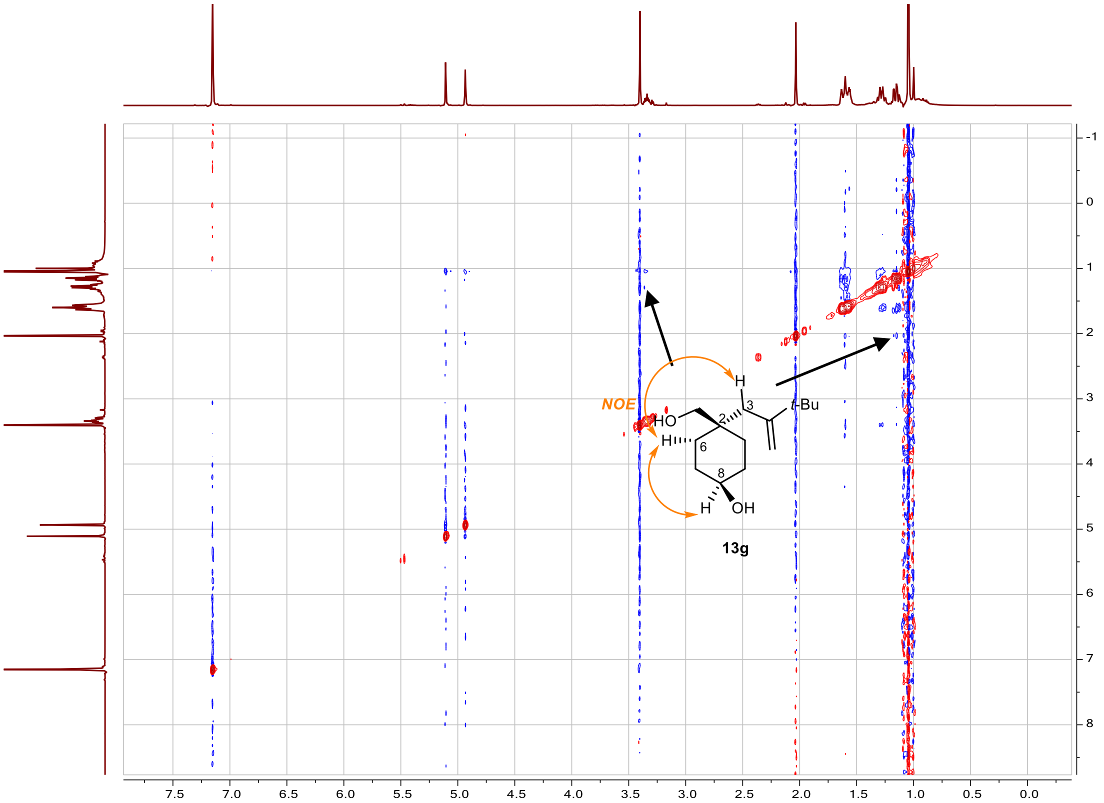

$^{1}H$ NMR (400 MHz, $C_6D_6$)

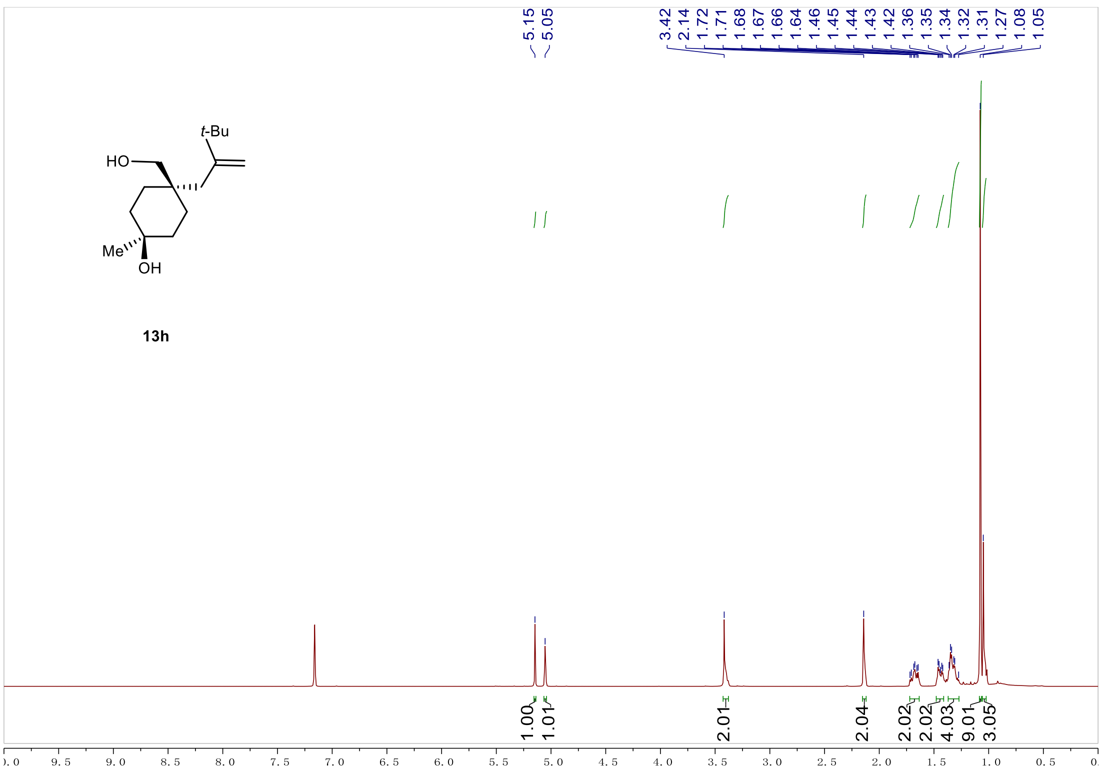


$^{13}C$ NMR (101 MHz, $C_6D_6$)

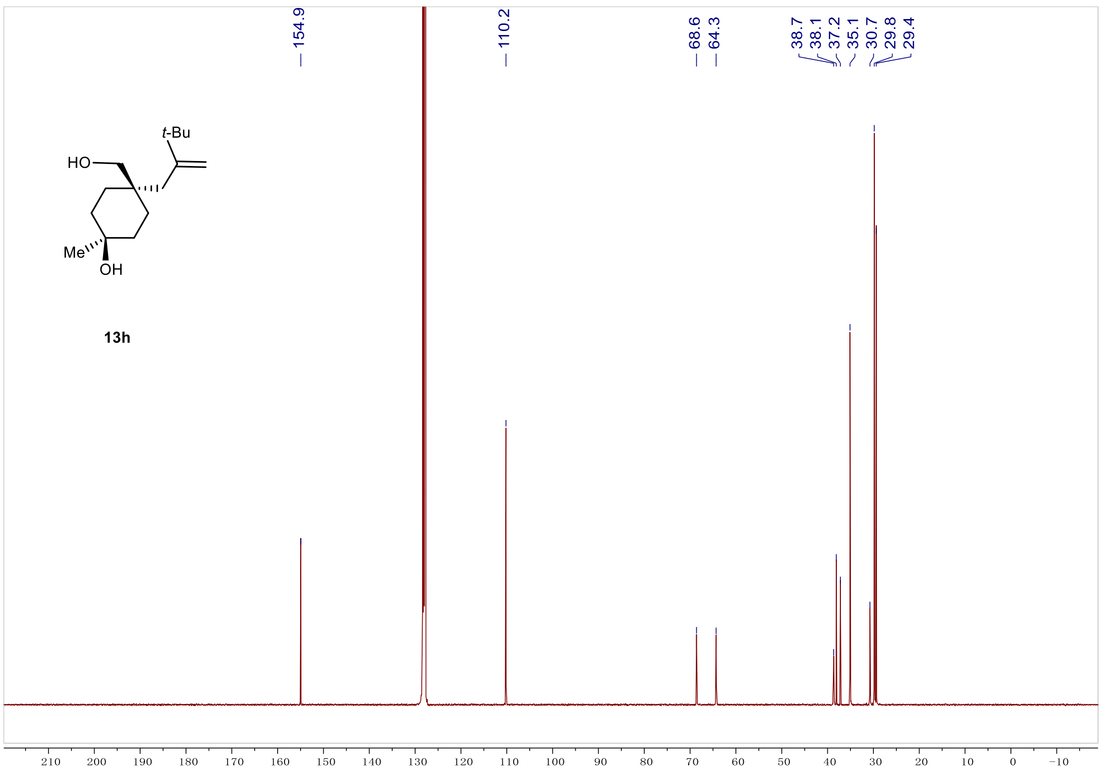

NOESY NMR (500 MHz, $C_6D_6$)

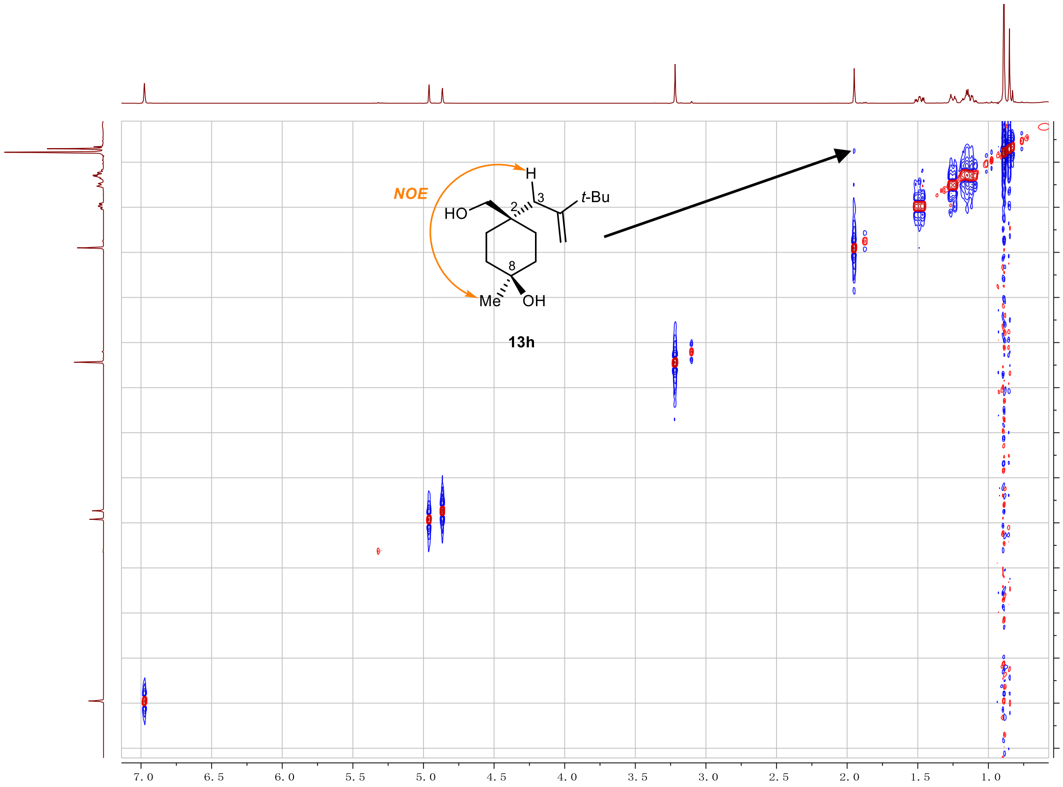

$^{1}$H NMR (400 MHz, $C_6D_6$)

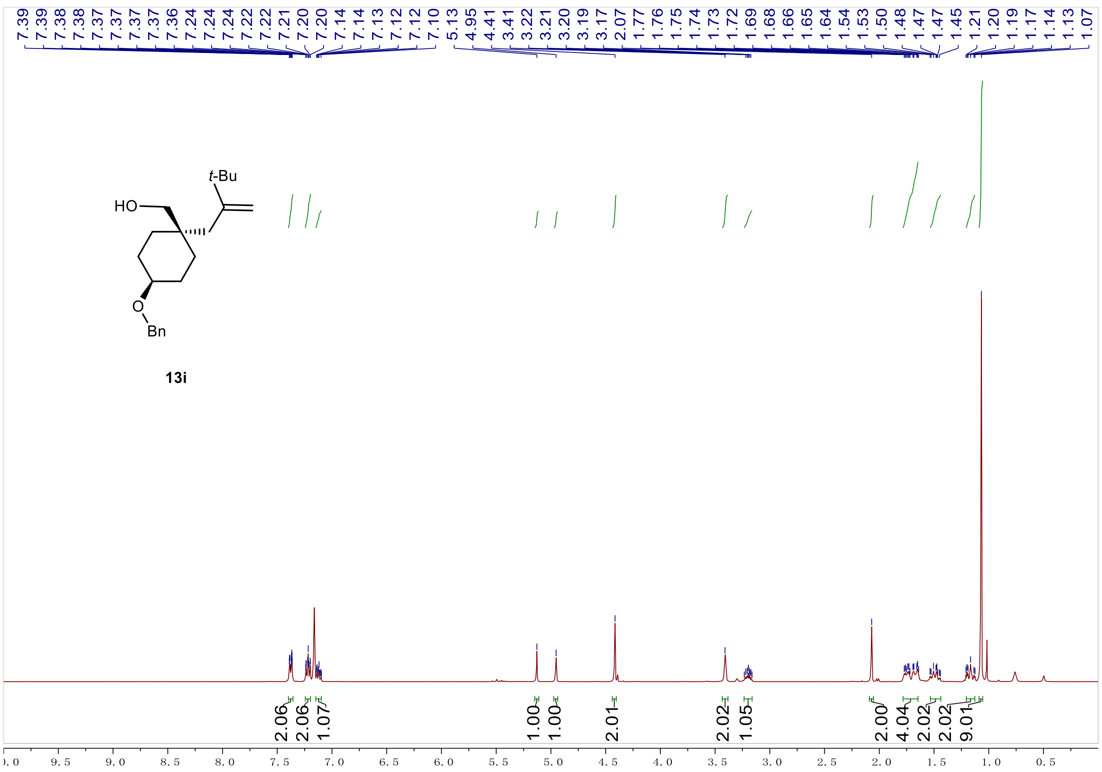

$^{13}$C NMR (101 MHz, $C_6D_6$)

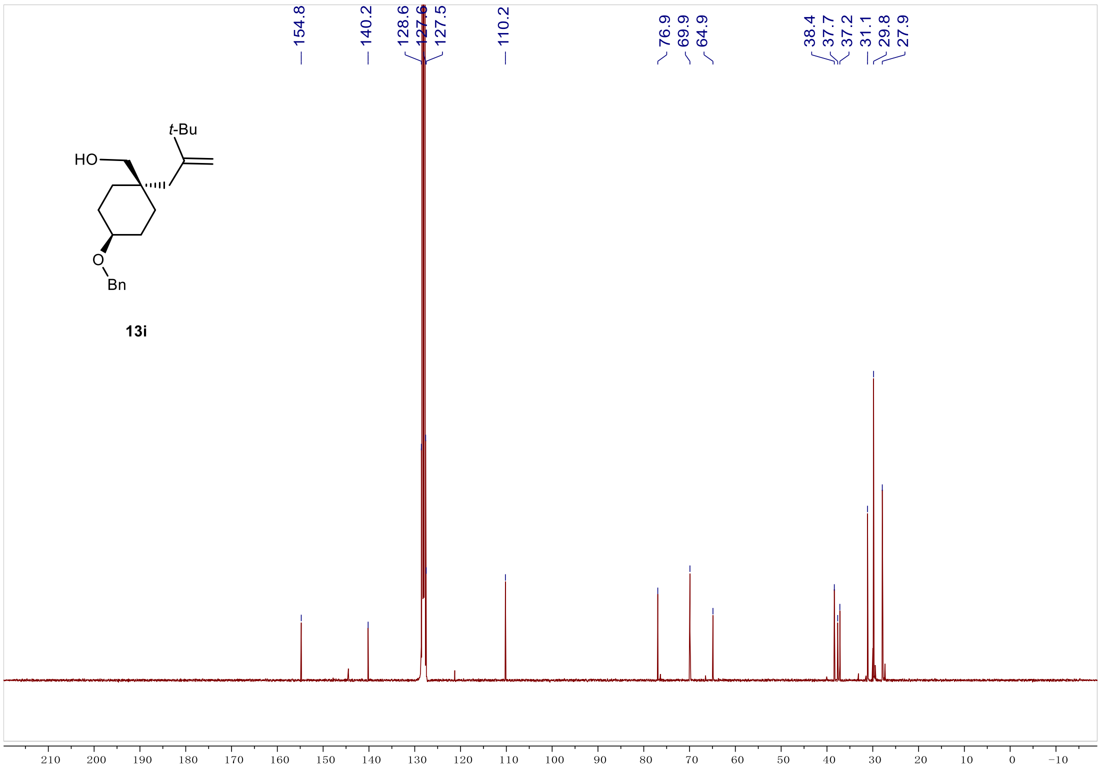

NOESY NMR (600 MHz, $C_6D_6$)

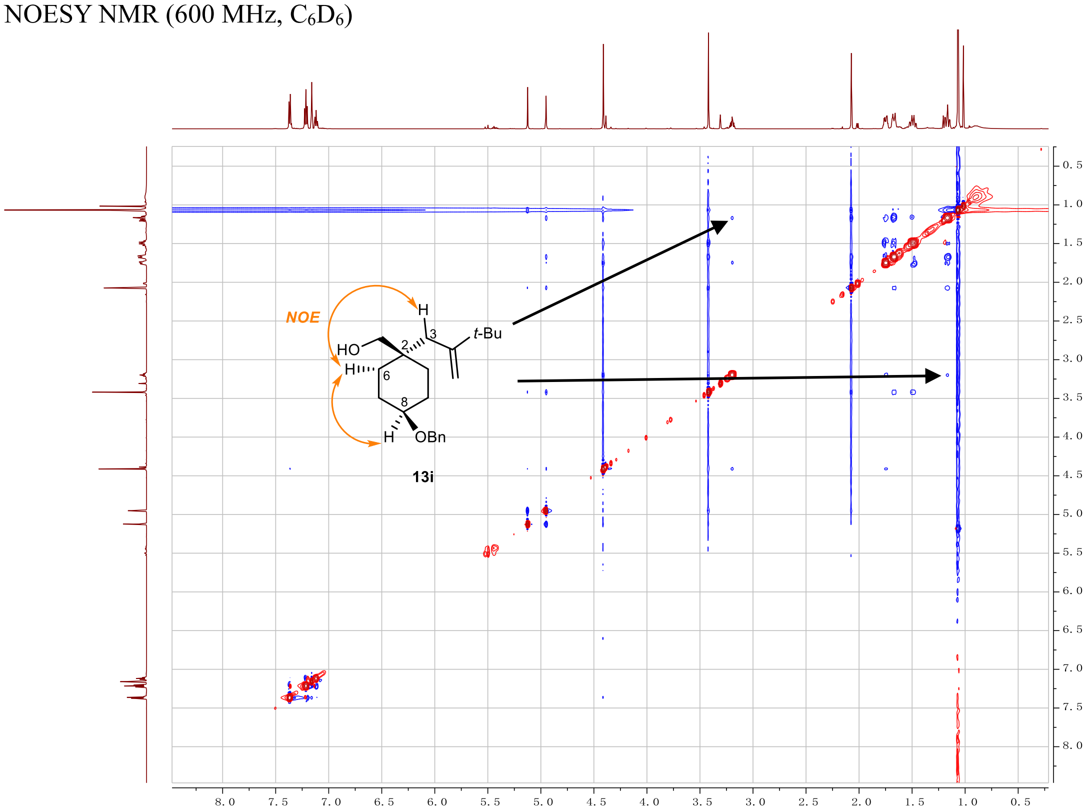

$^{1}$H NMR (400 MHz, $CDCl_3$)

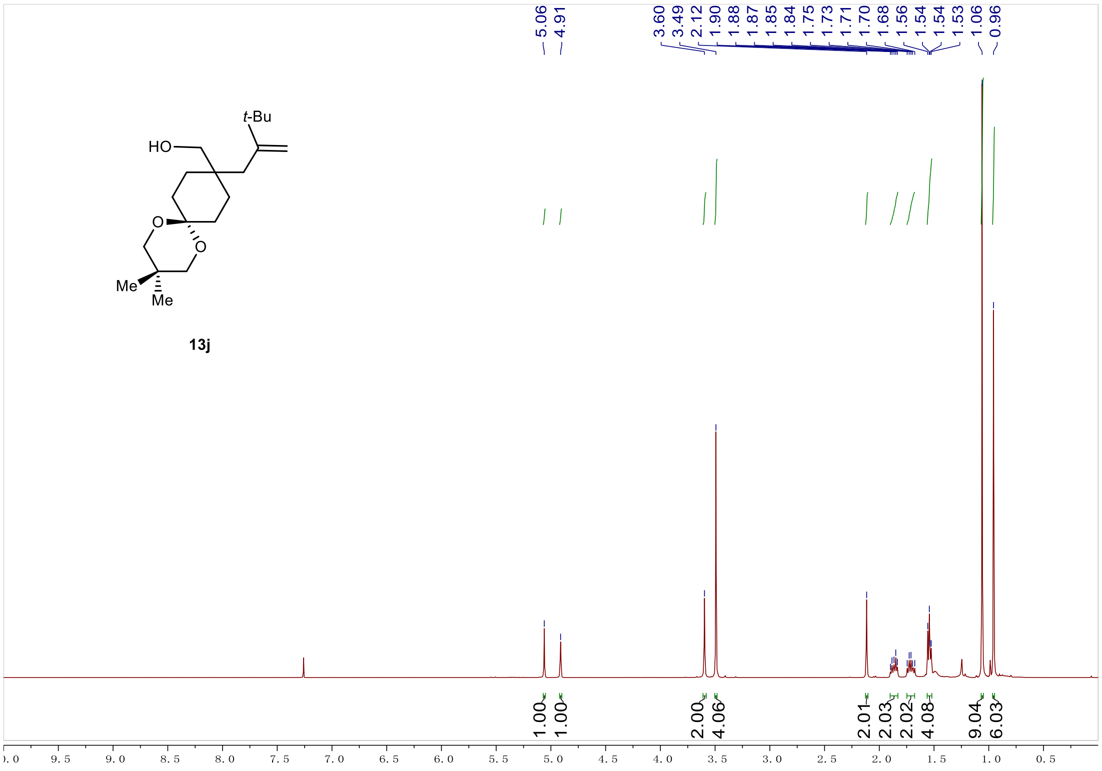


$^{13}$C NMR (101 MHz, $CDCl_3$)

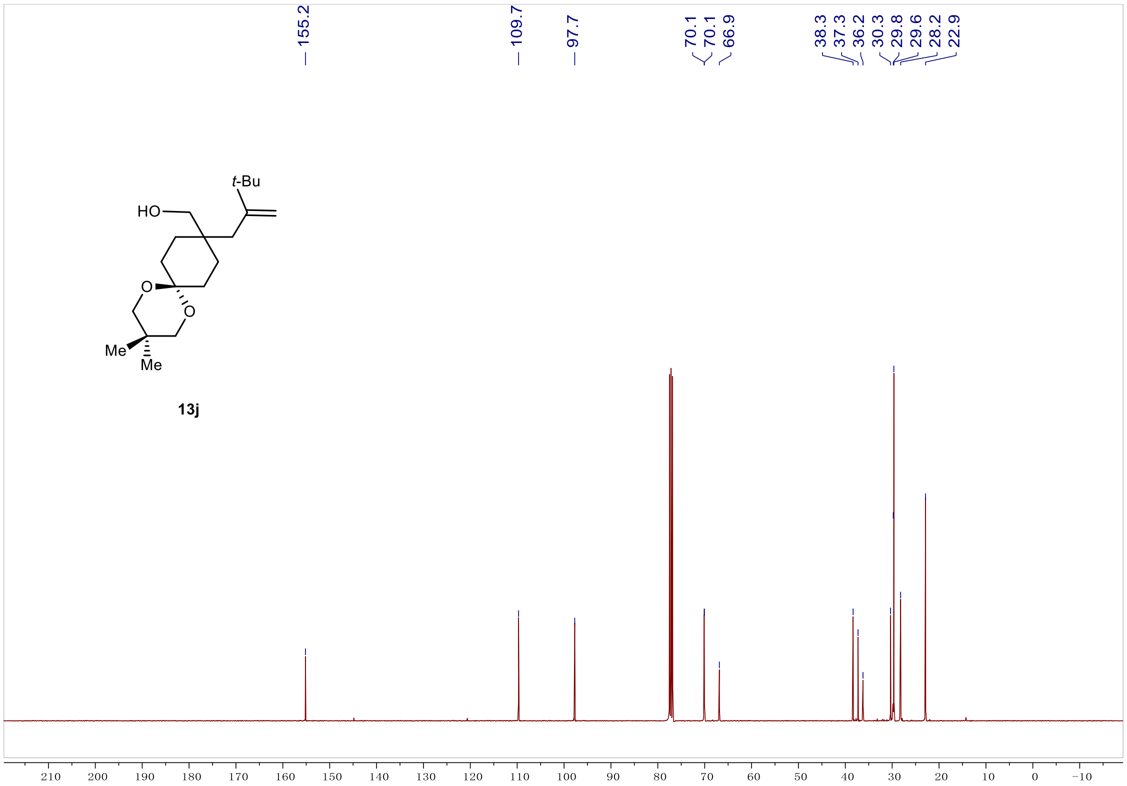

$^{1}H$ NMR (400 MHz, $C_6D_6$)

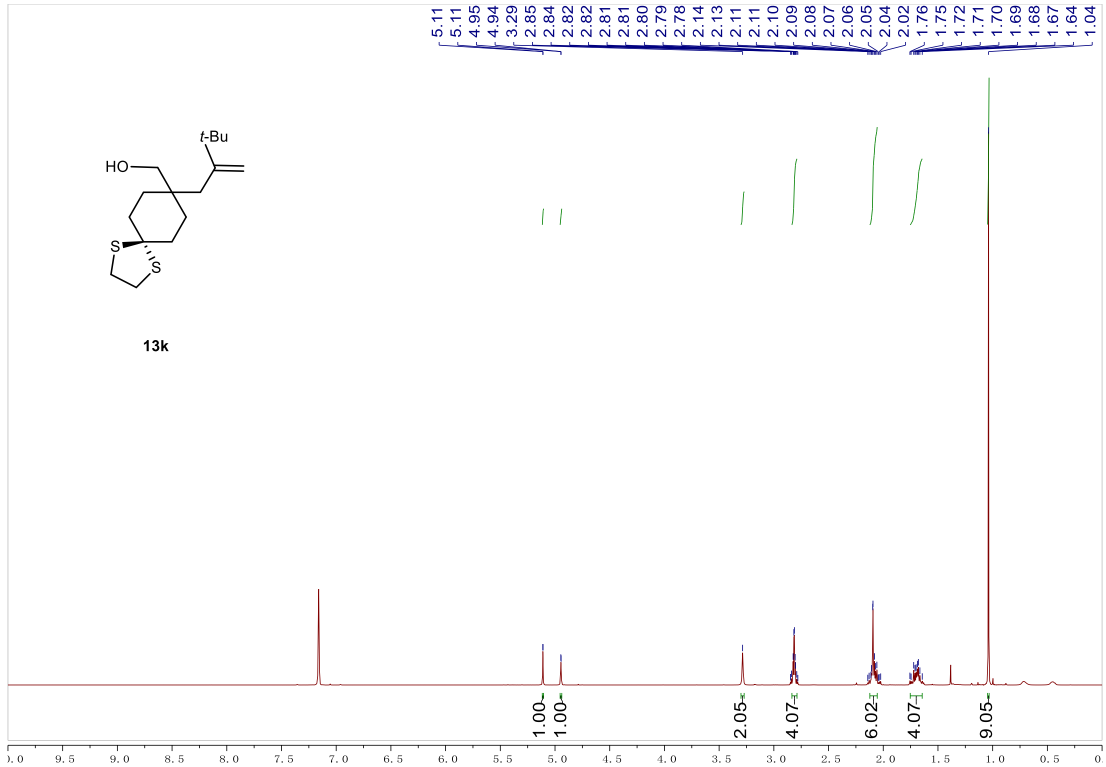


$^{13}C$ NMR (101 MHz, $C_6D_6$)

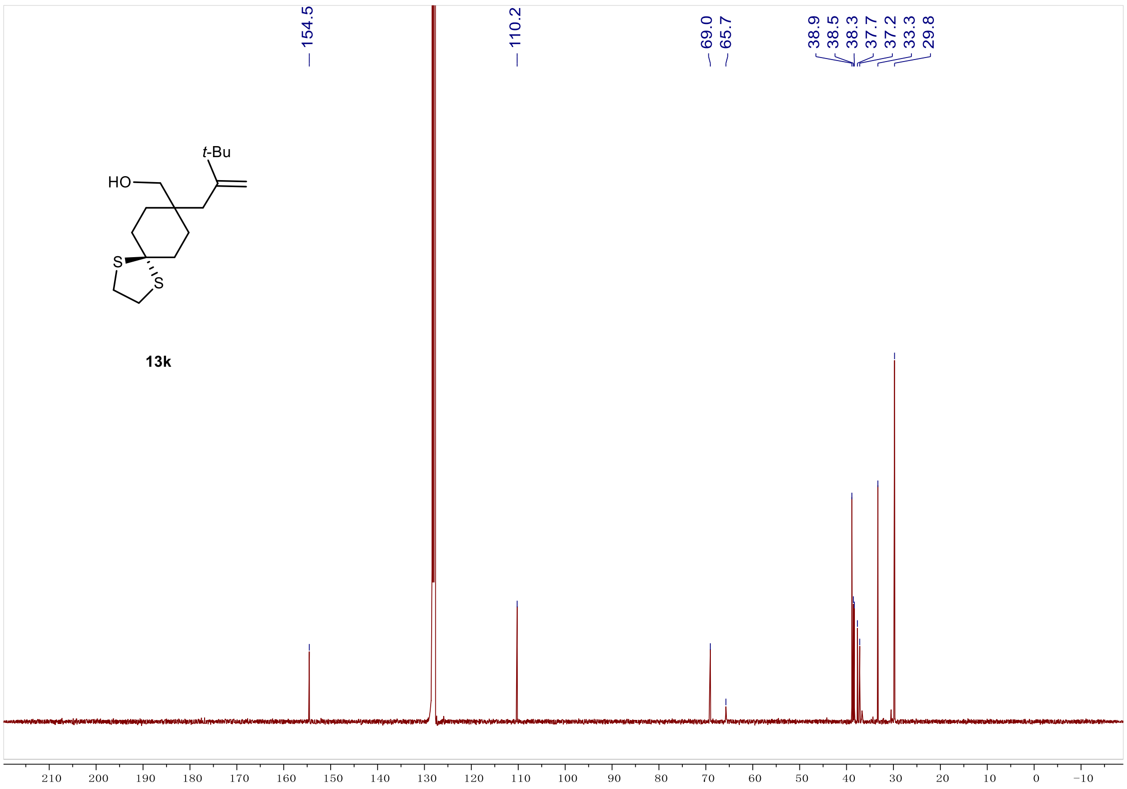

$^1$H NMR (400 MHz, $C_6D_6$)

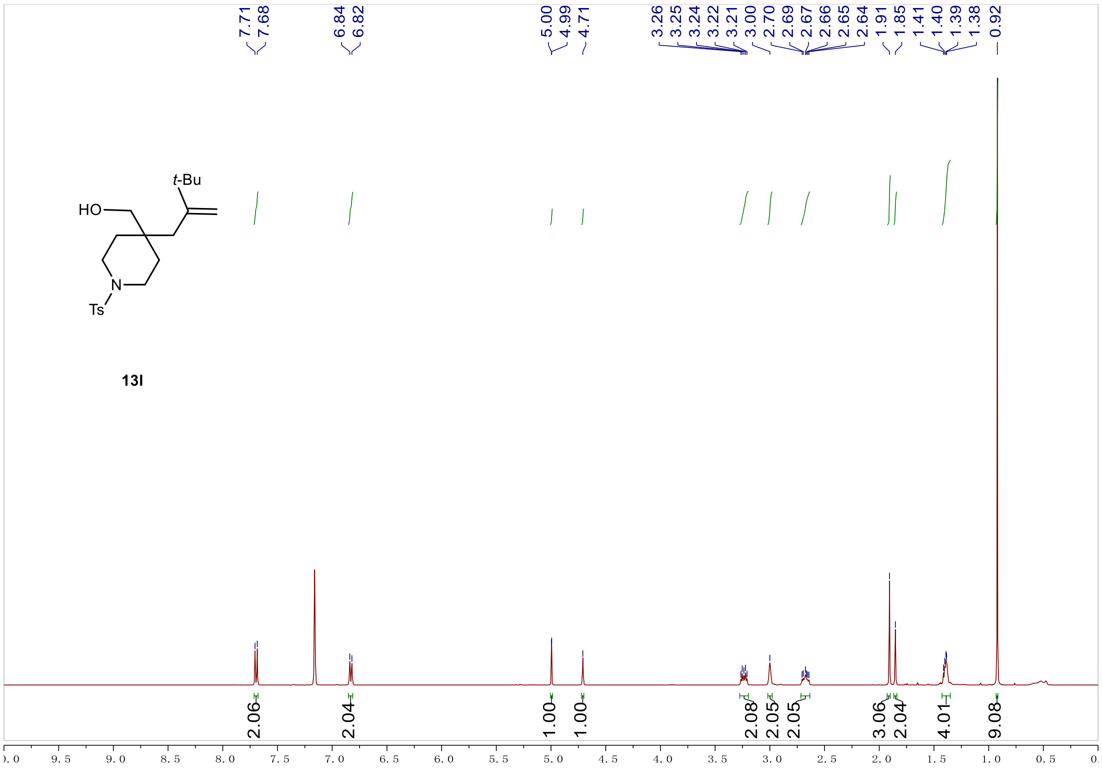


$^{13}$C NMR (101 MHz, $C_6D_6$)

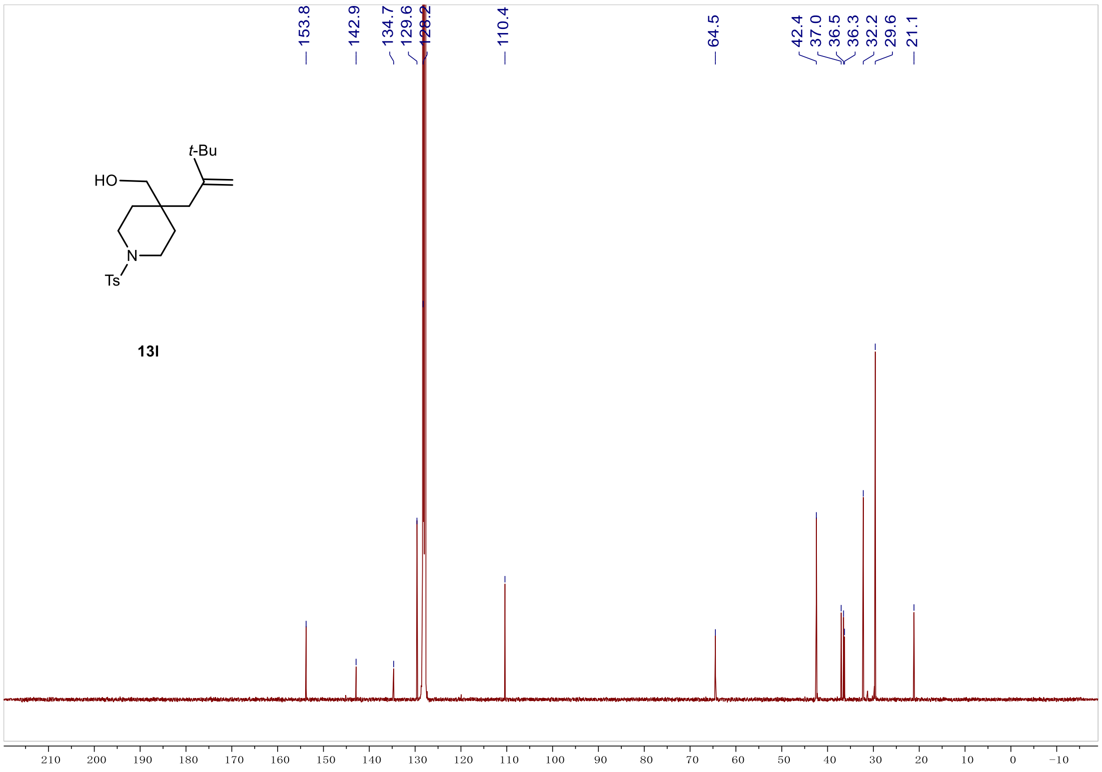

$^{1}H$ NMR (400 MHz, $C_6D_6$)

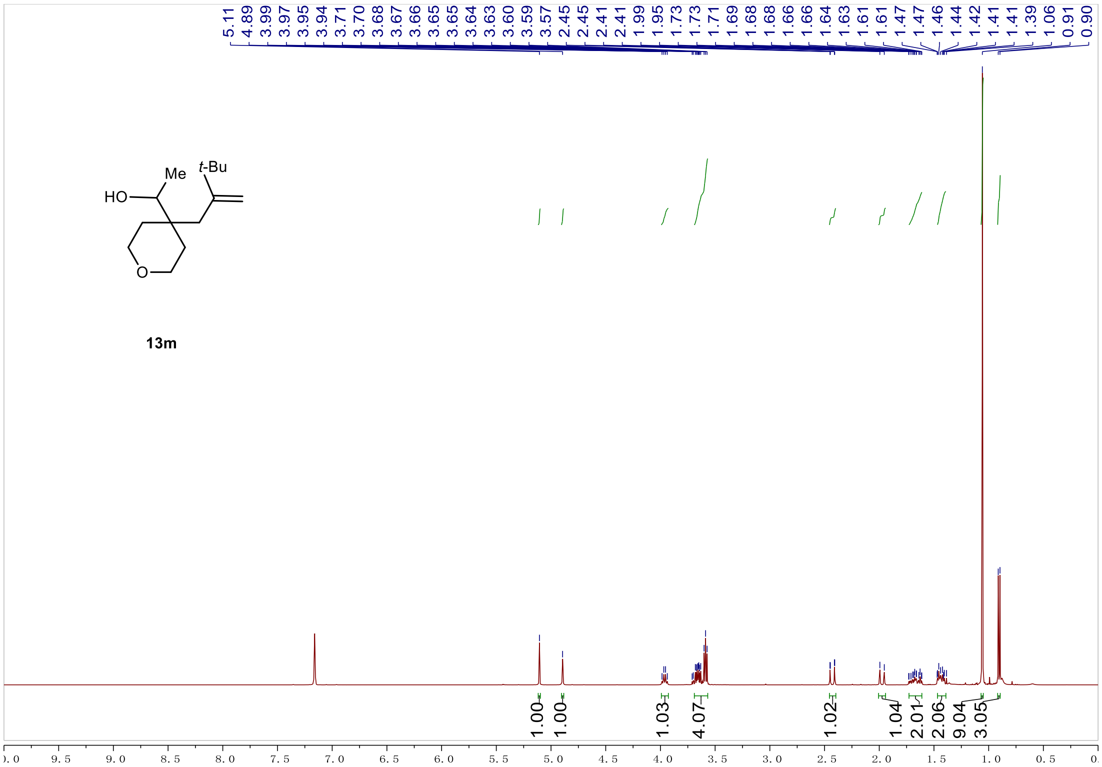


$^{13}C$ NMR (101 MHz, $C_6D_6$)

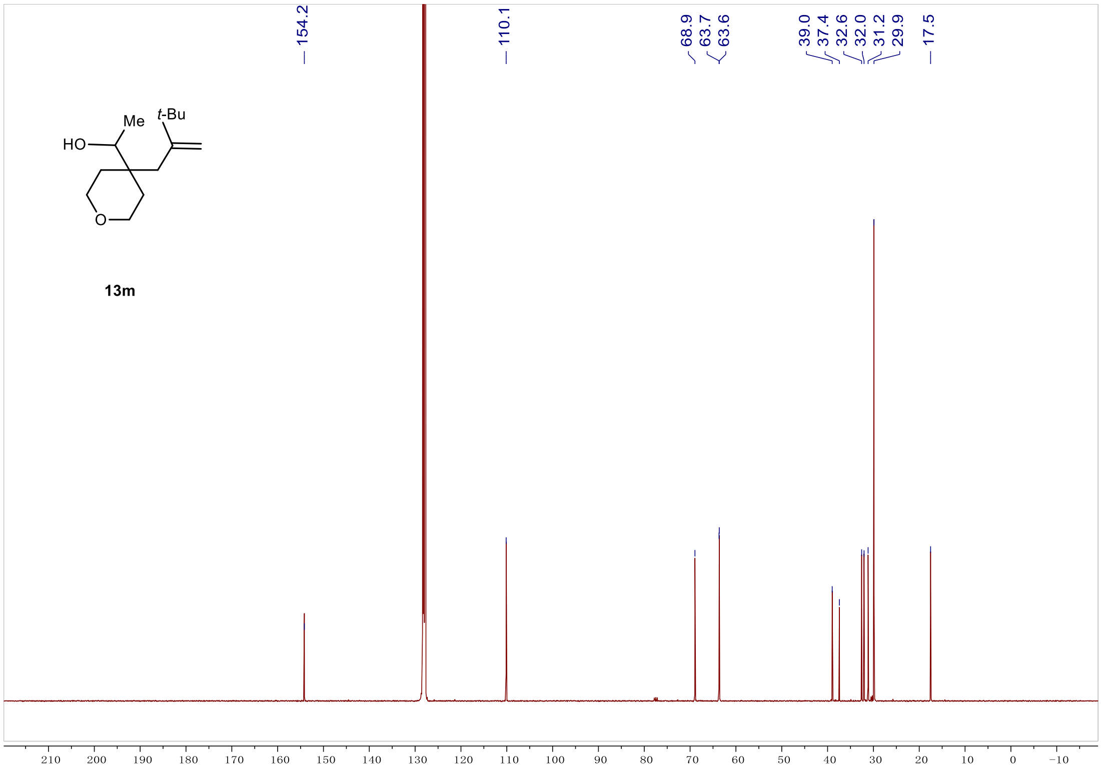

$^{1}H$ NMR (400 MHz, $C_6D_6$)

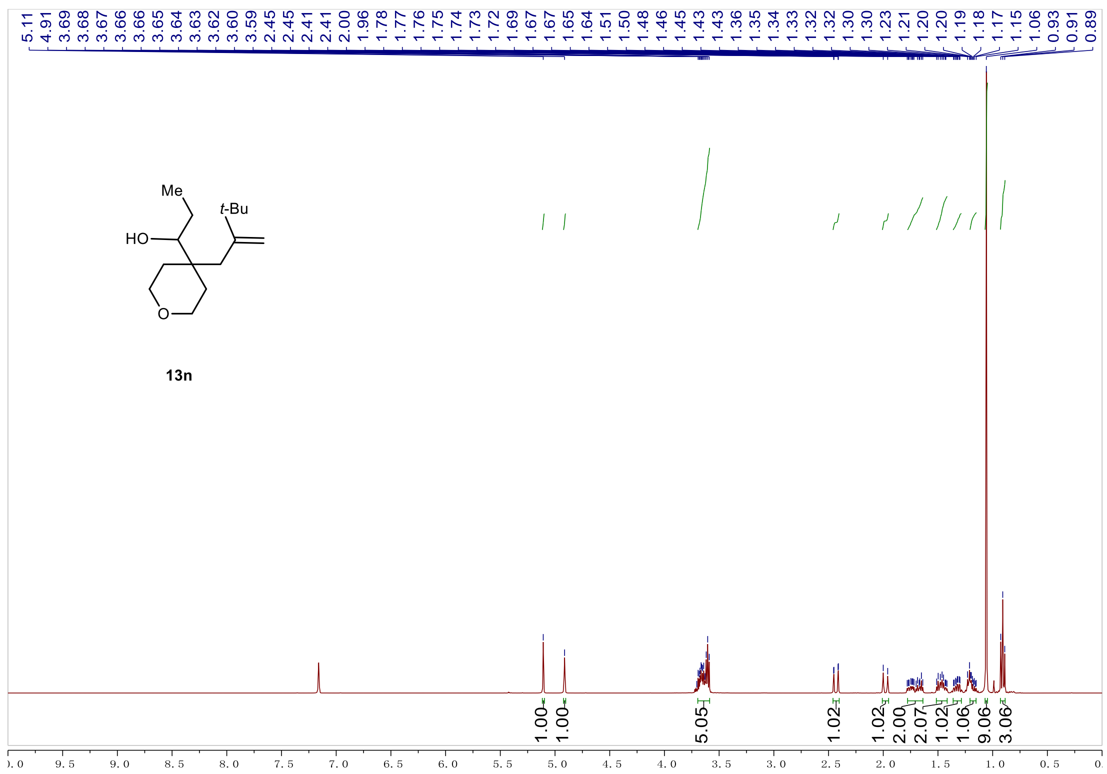

$^{13}C$ NMR (101 MHz, $C_6D_6$)

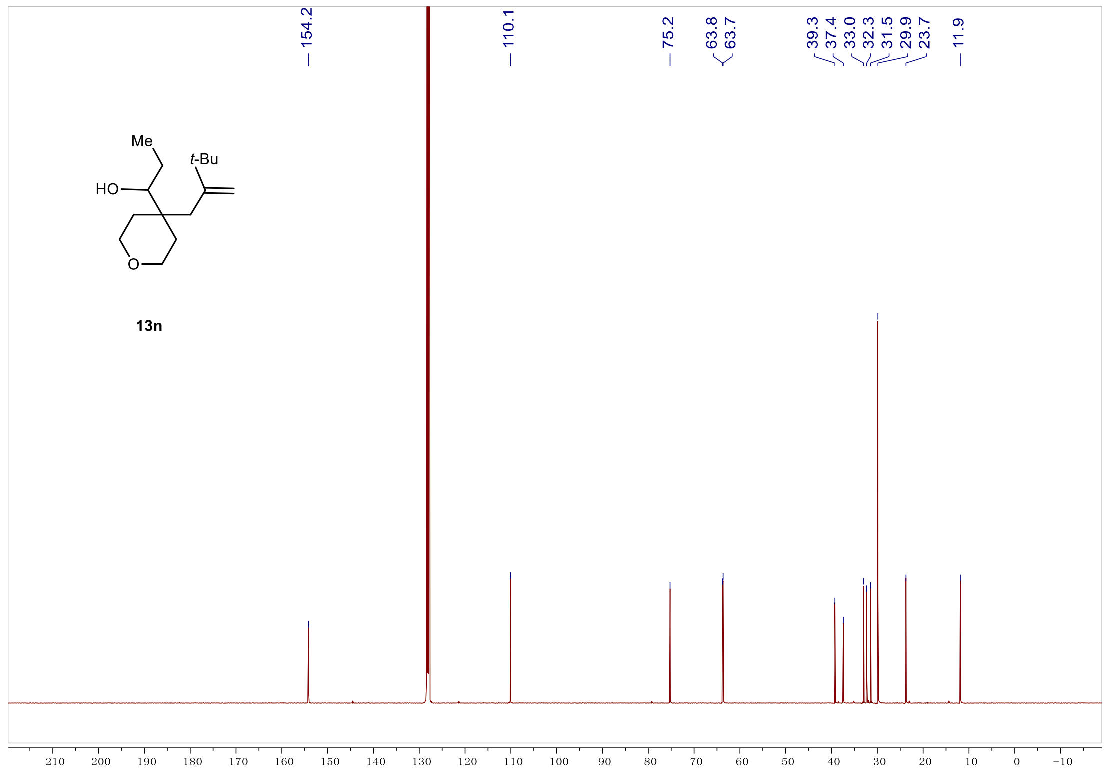

$^{1}H$ NMR (400 MHz, $CDCl_3$)

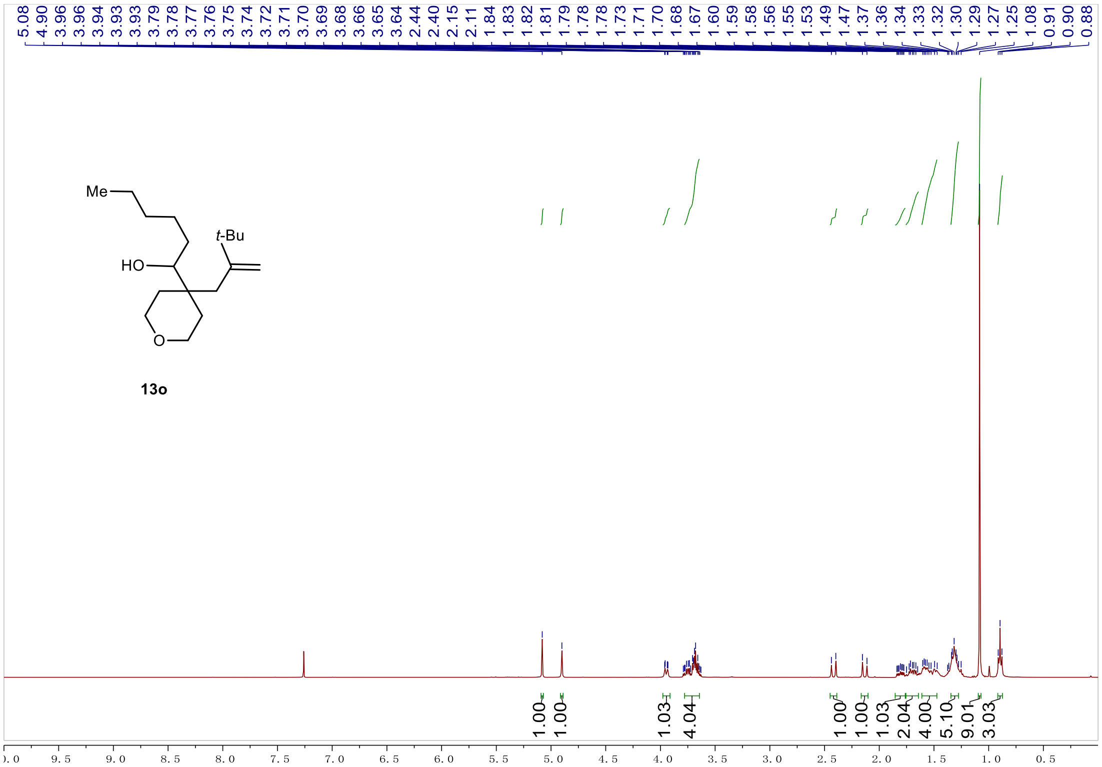


$^{13}C$ NMR (101 MHz, $CDCl_3$)

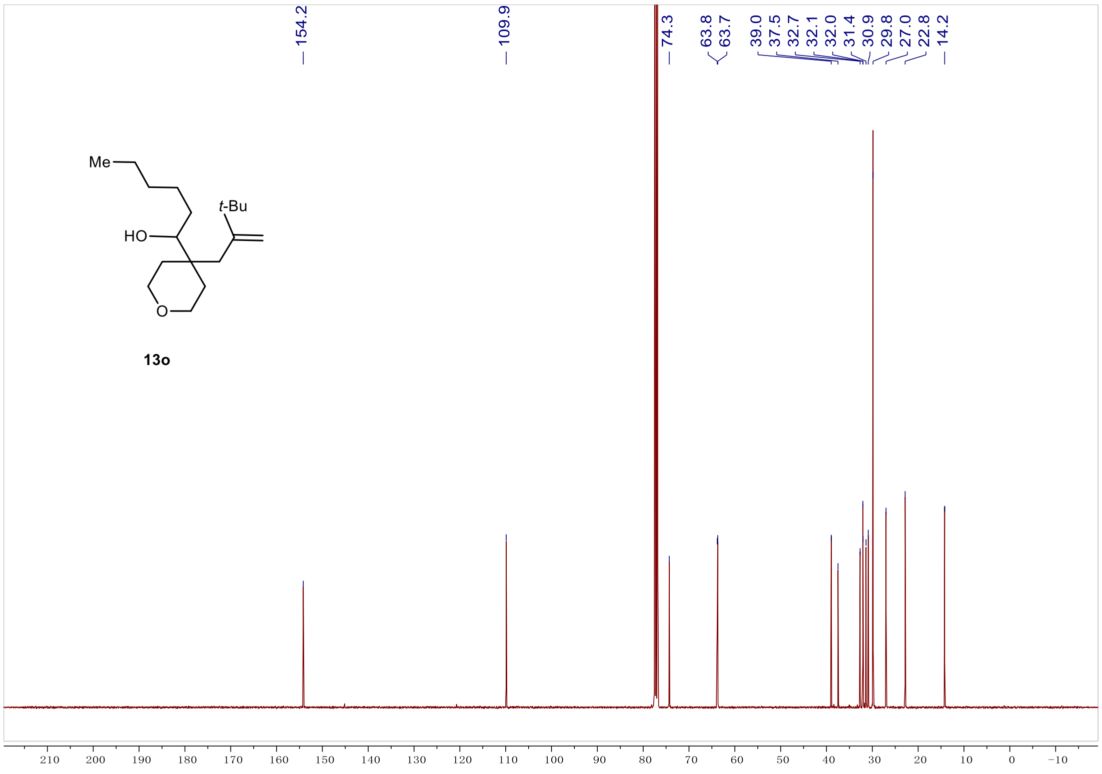

$^{1}H$ NMR (400 MHz, $C_6D_6$)

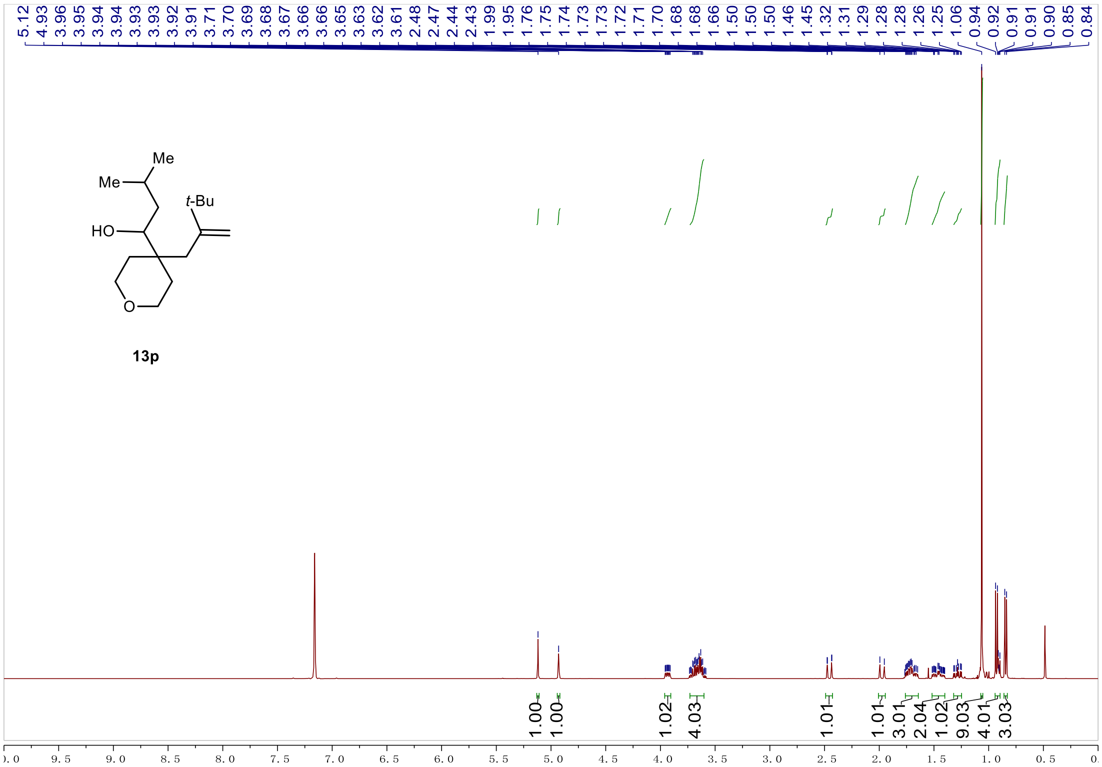

$^{13}C$ NMR (101 MHz, $C_6D_6$)

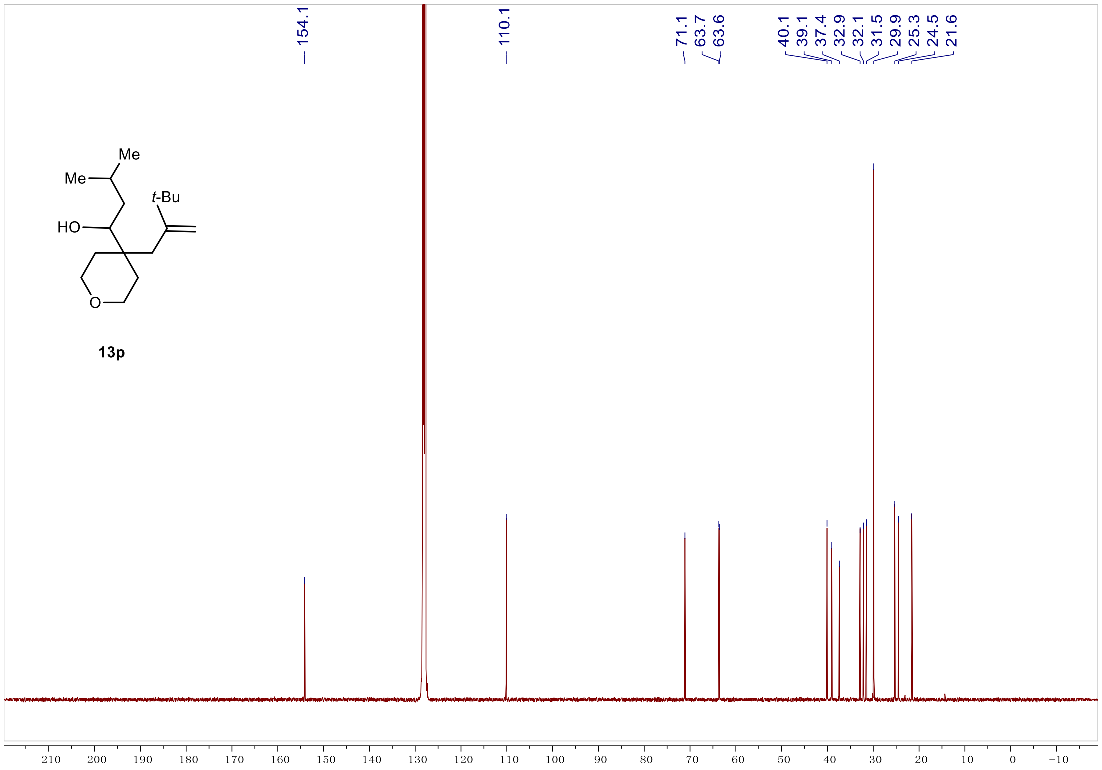

$^{1}H$ NMR (400 MHz, $C_6D_6$)

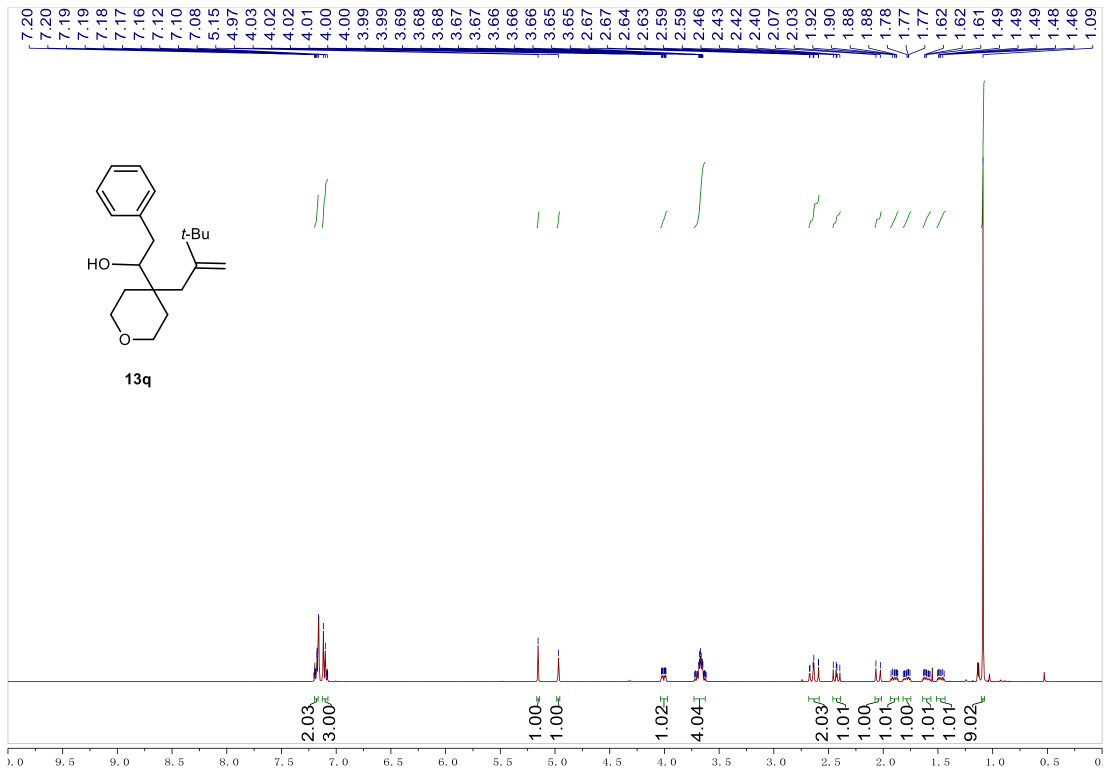

$^{13}C$ NMR (101 MHz, $C_6D_6$)

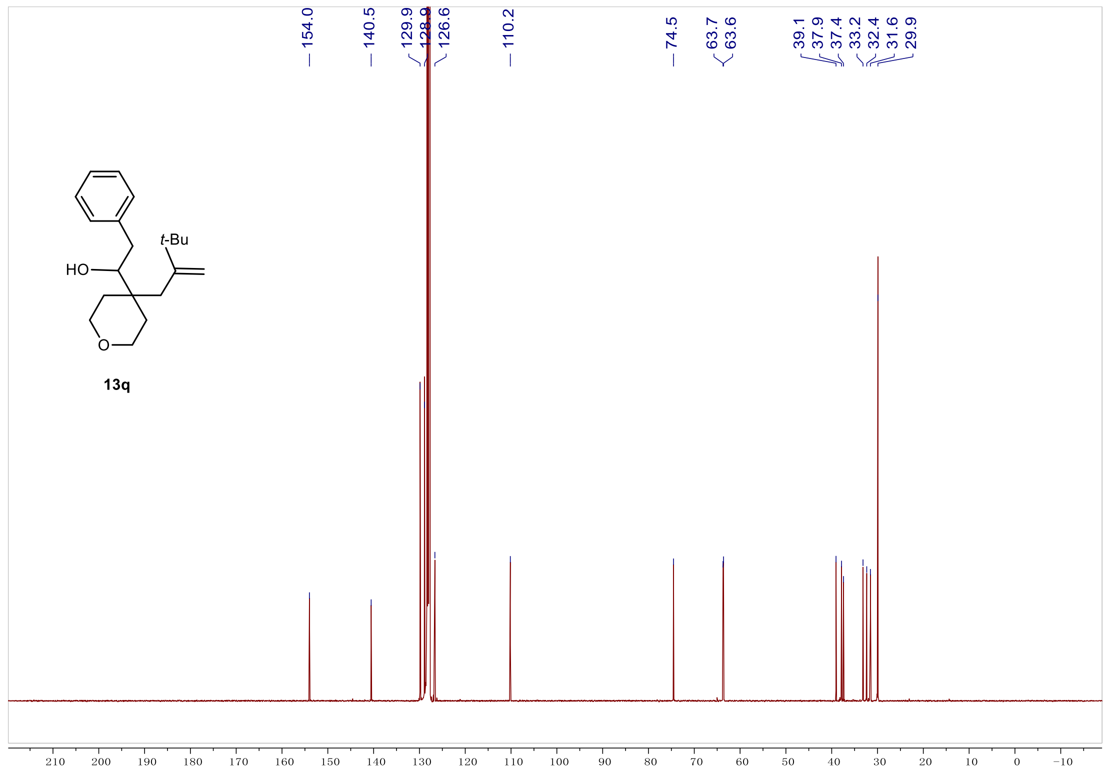

$^1H$ NMR (400 MHz, $C_6D_6$)

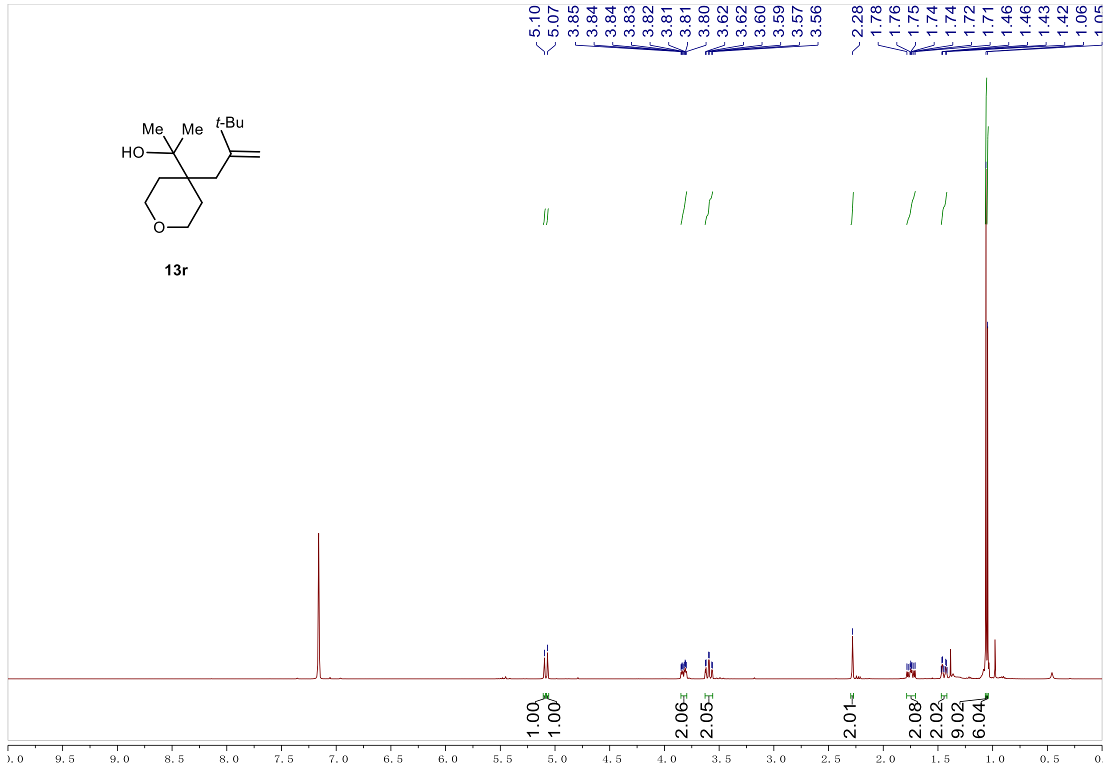


$^{13}C$ NMR (101 MHz, $C_6D_6$)

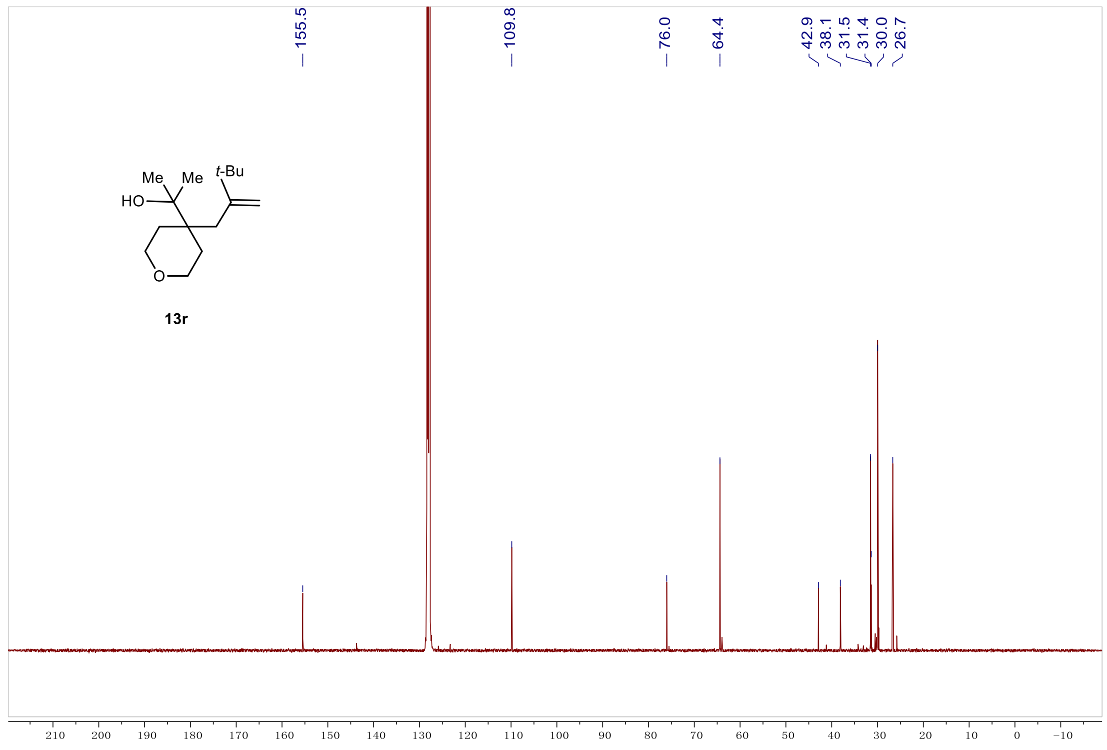

$^{1}H$ NMR (400 MHz, $CDCl_3$)

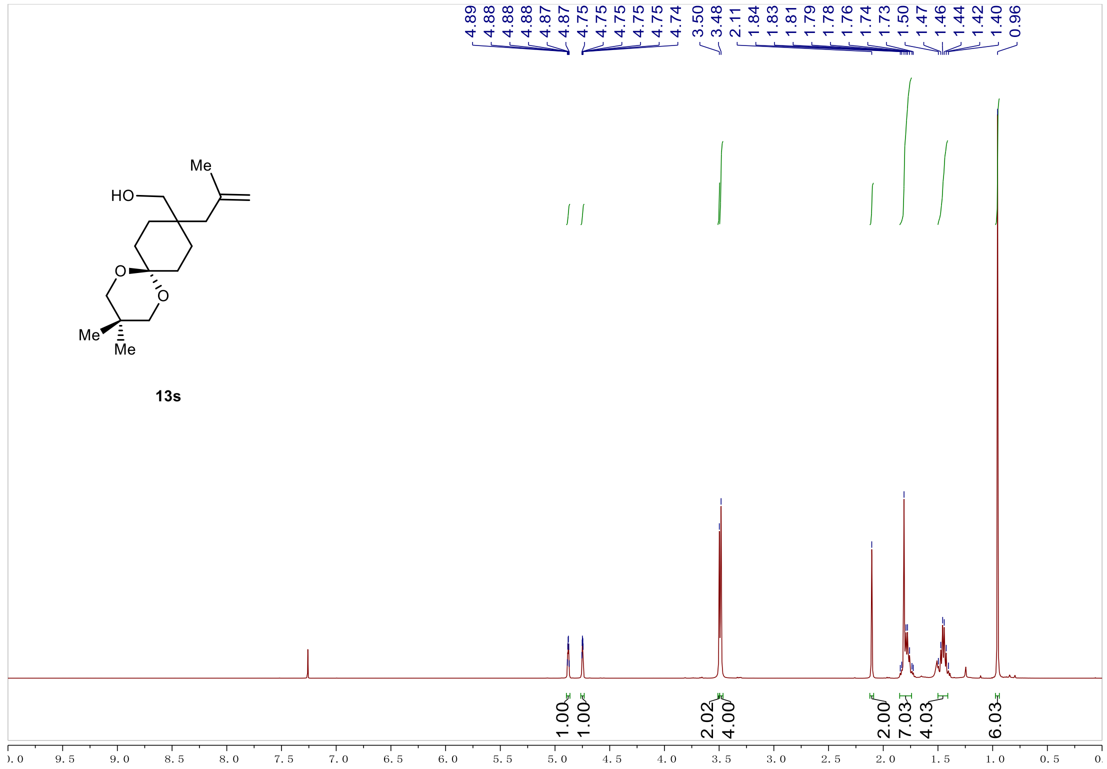


$^{13}C$ NMR (101 MHz, $CDCl_3$)

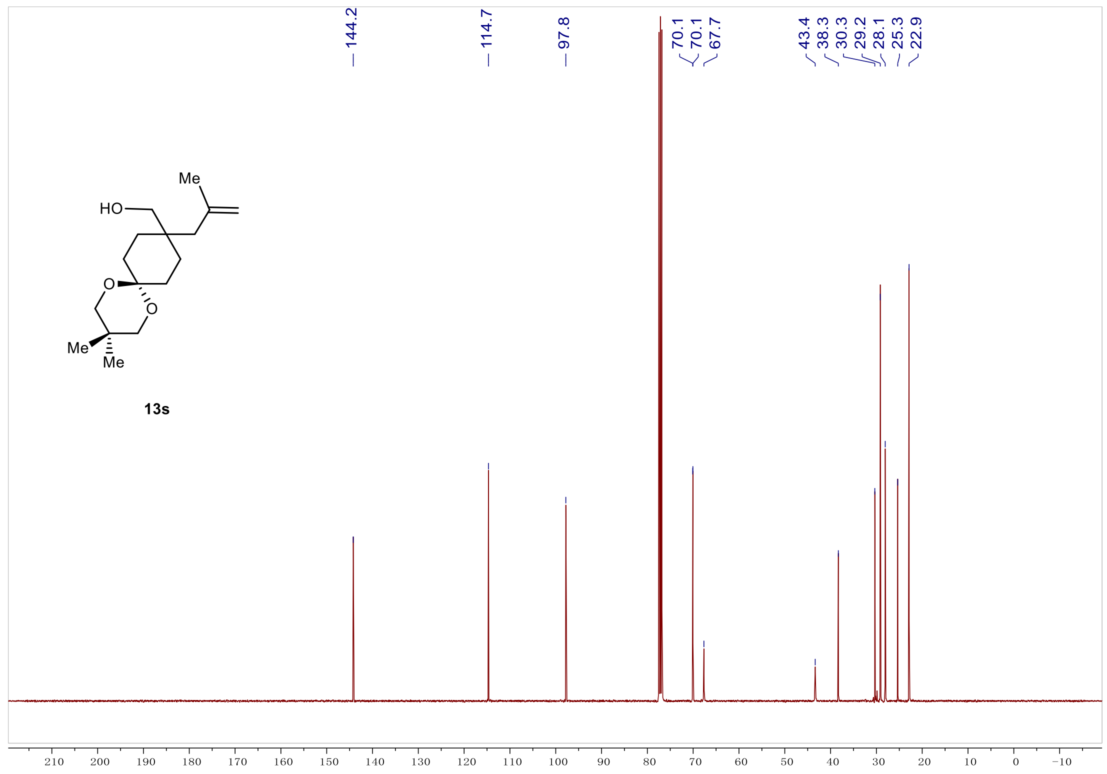

$^{1}H$ NMR (400 MHz, $C_6D_6$)

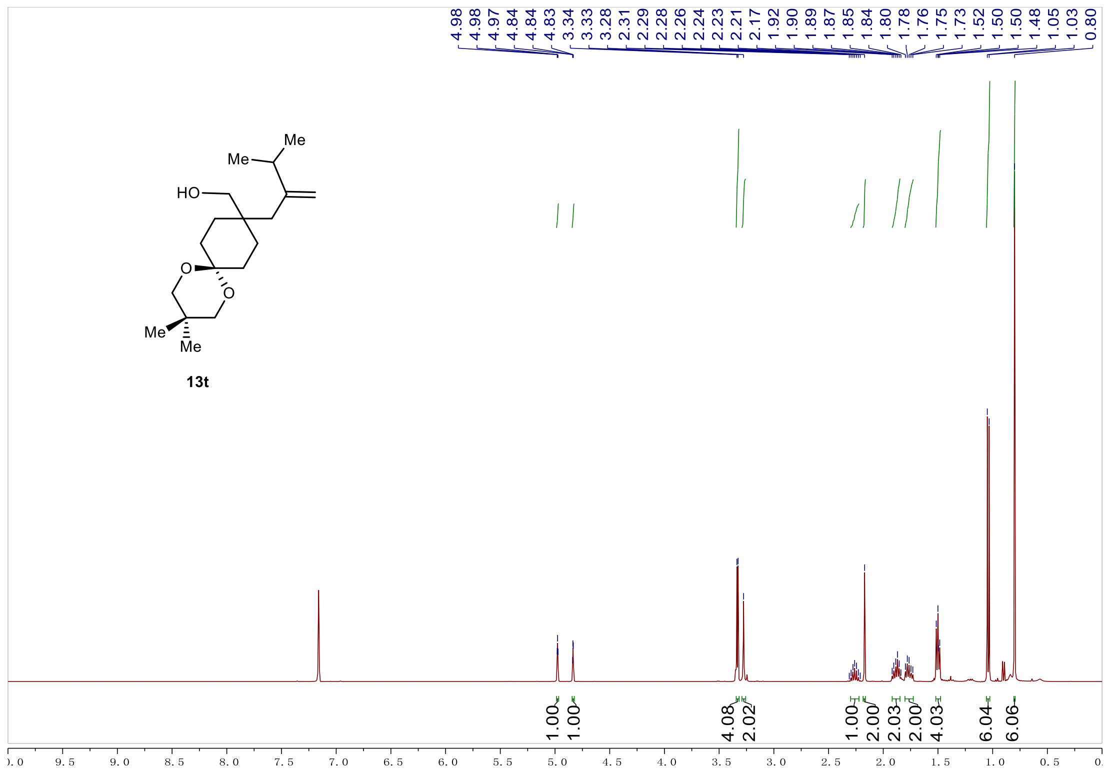

$^{13}C$ NMR (101 MHz, $C_6D_6$)

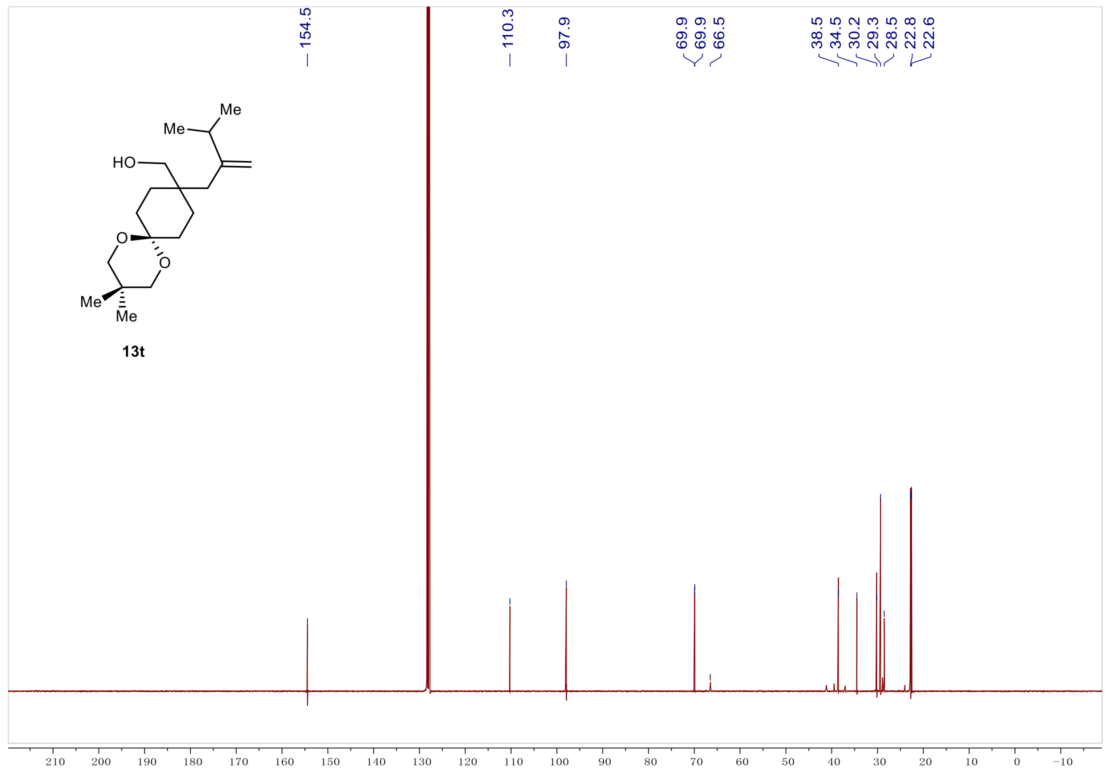

$^{1}$H NMR (400 MHz, $C_6D_6$)

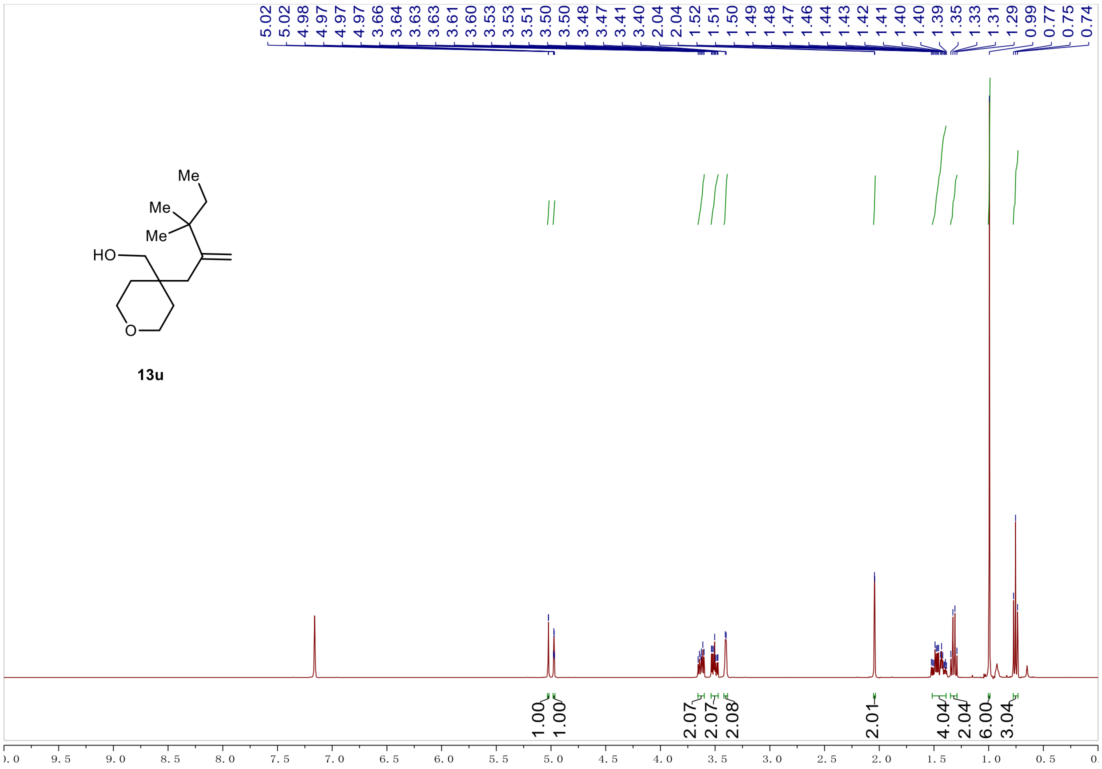


$^{13}$C NMR (101 MHz, $C_6D_6$)

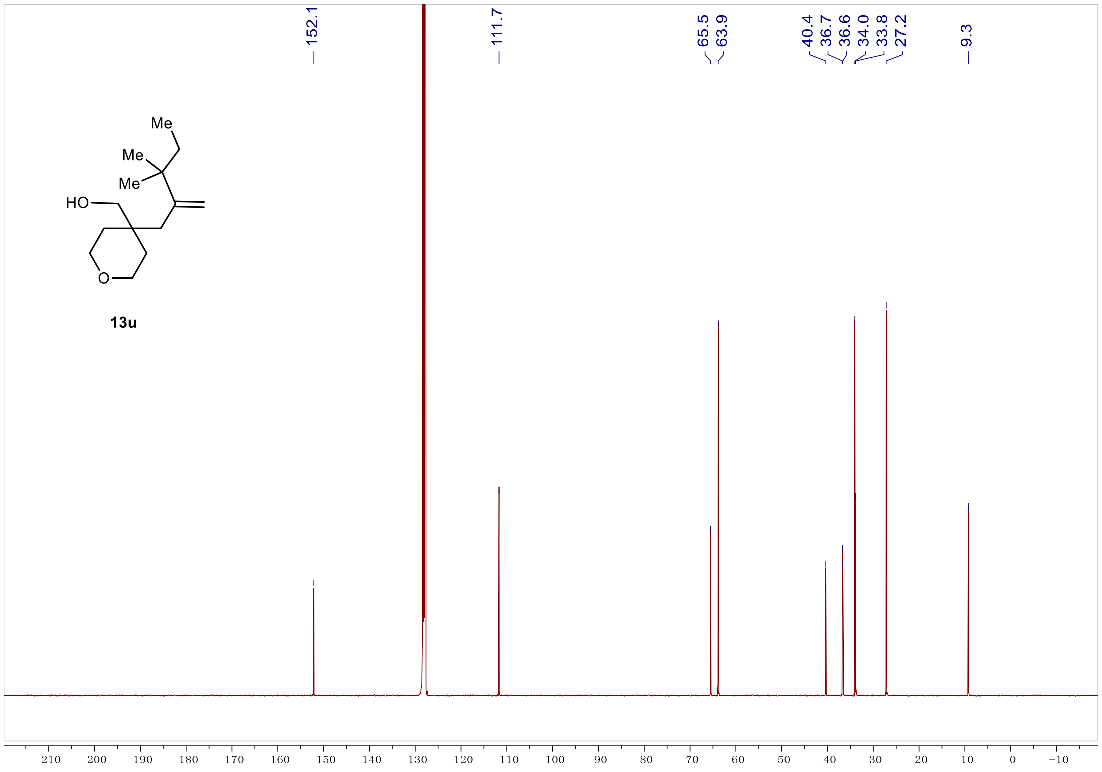

$^{1}H$ NMR (400 MHz, $C_6D_6$)

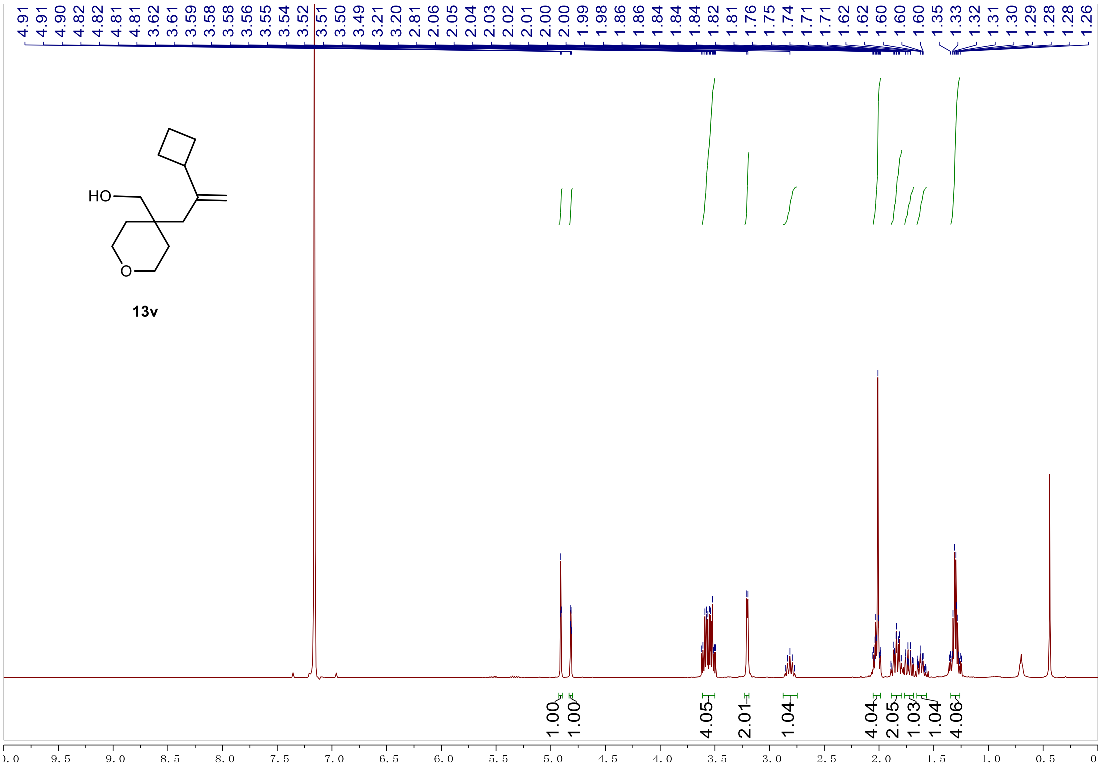


$^{13}C$ NMR (101 MHz, $C_6D_6$)

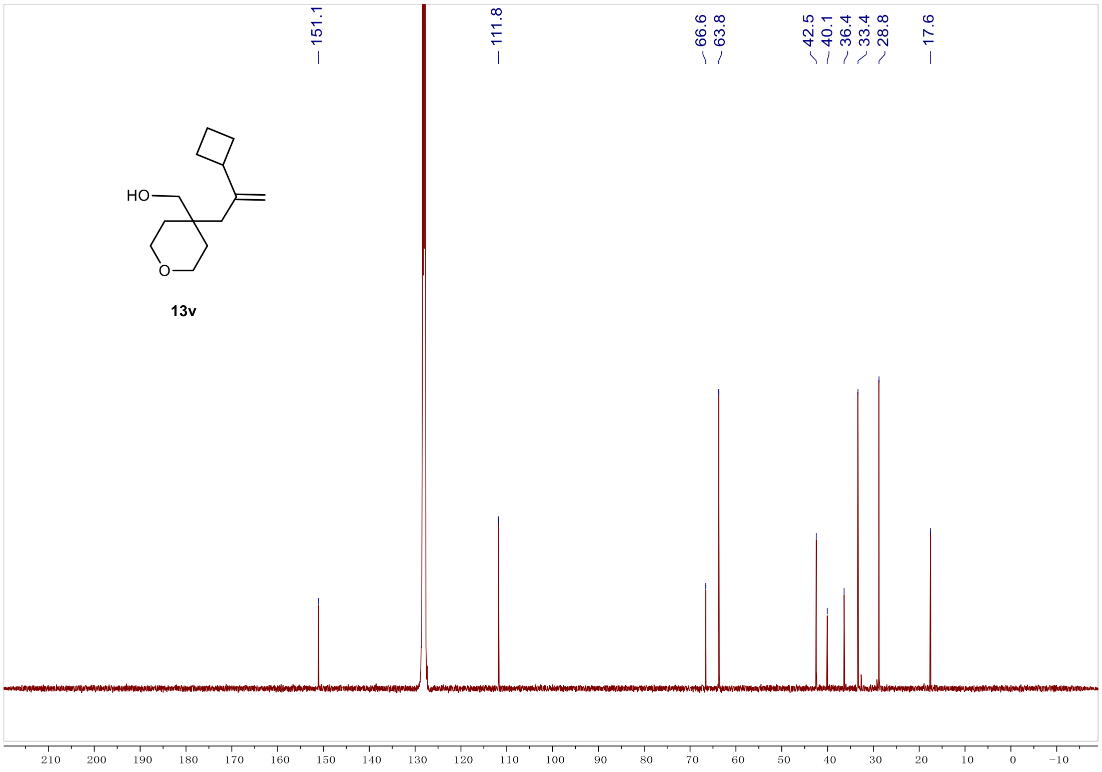

$^{1}$H NMR (400 MHz, $C_6D_6$)

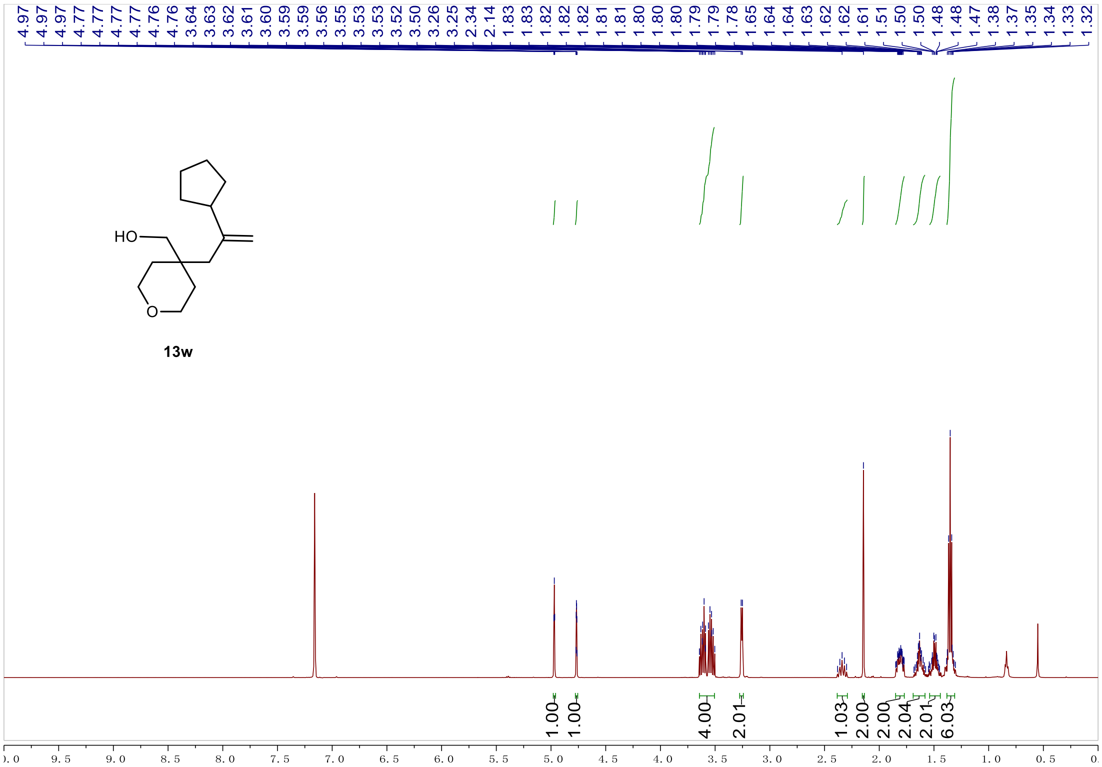

$^{13}$C NMR (101 MHz, $C_6D_6$)

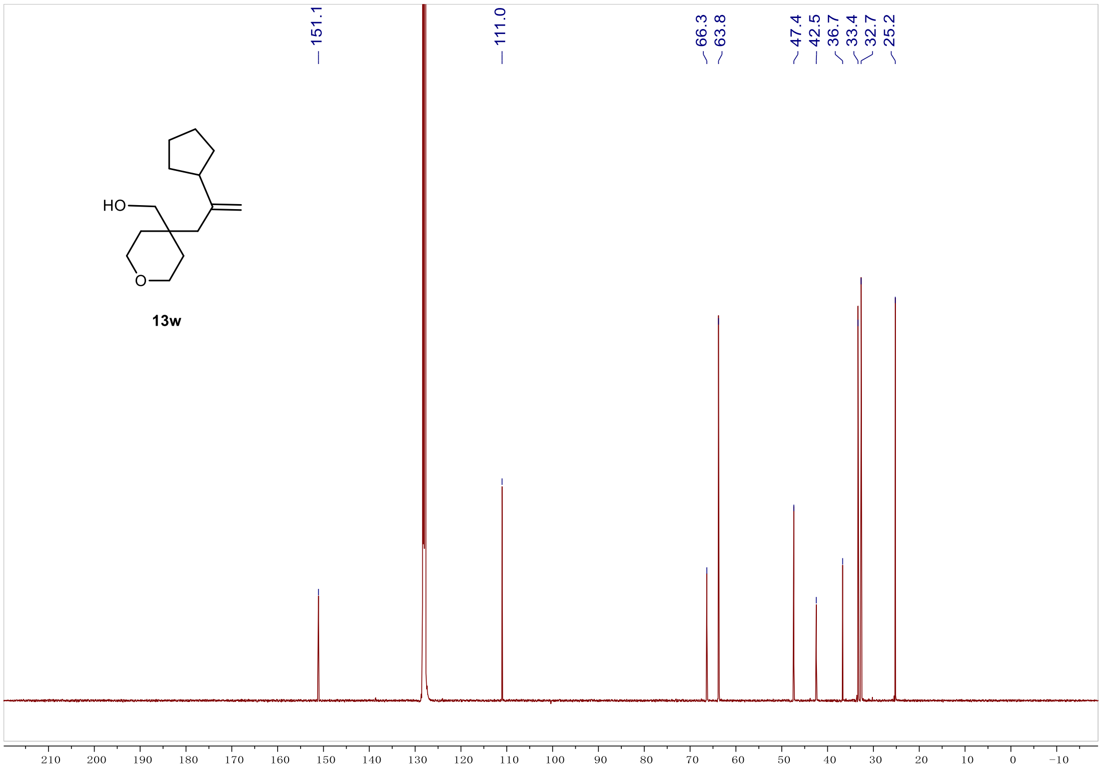

$^{1}$H NMR (400 MHz, $C_6D_6$)

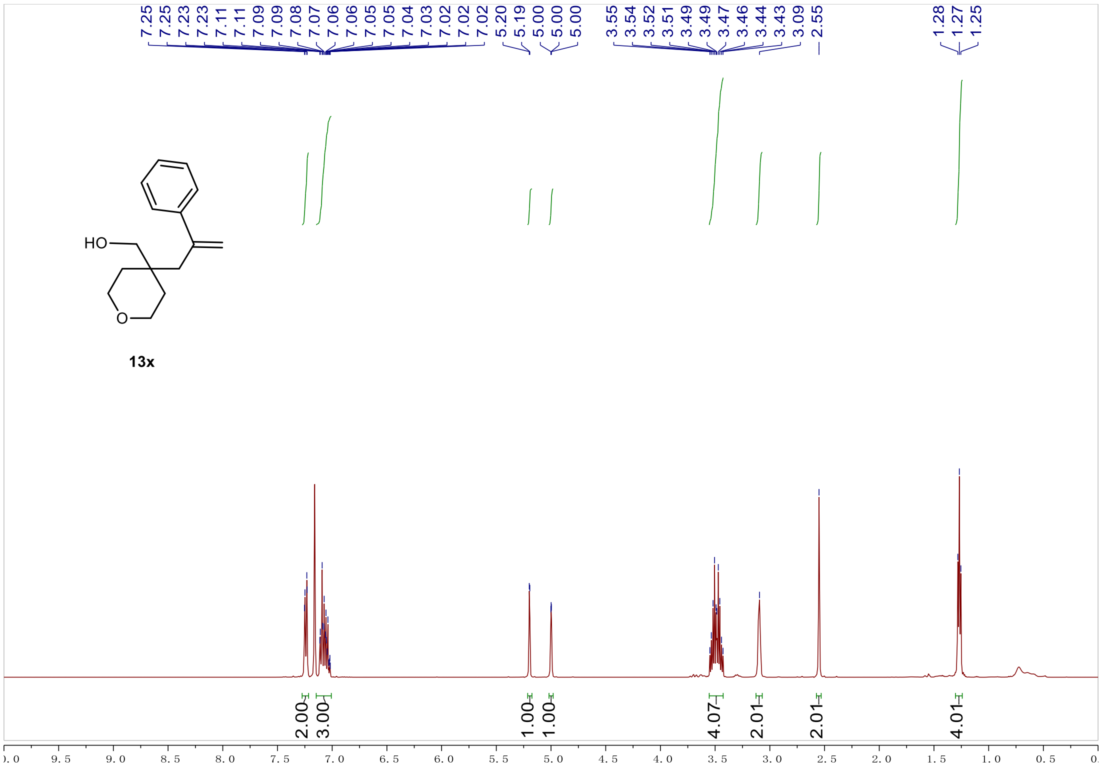


$^{13}$C NMR (101 MHz, $C_6D_6$)

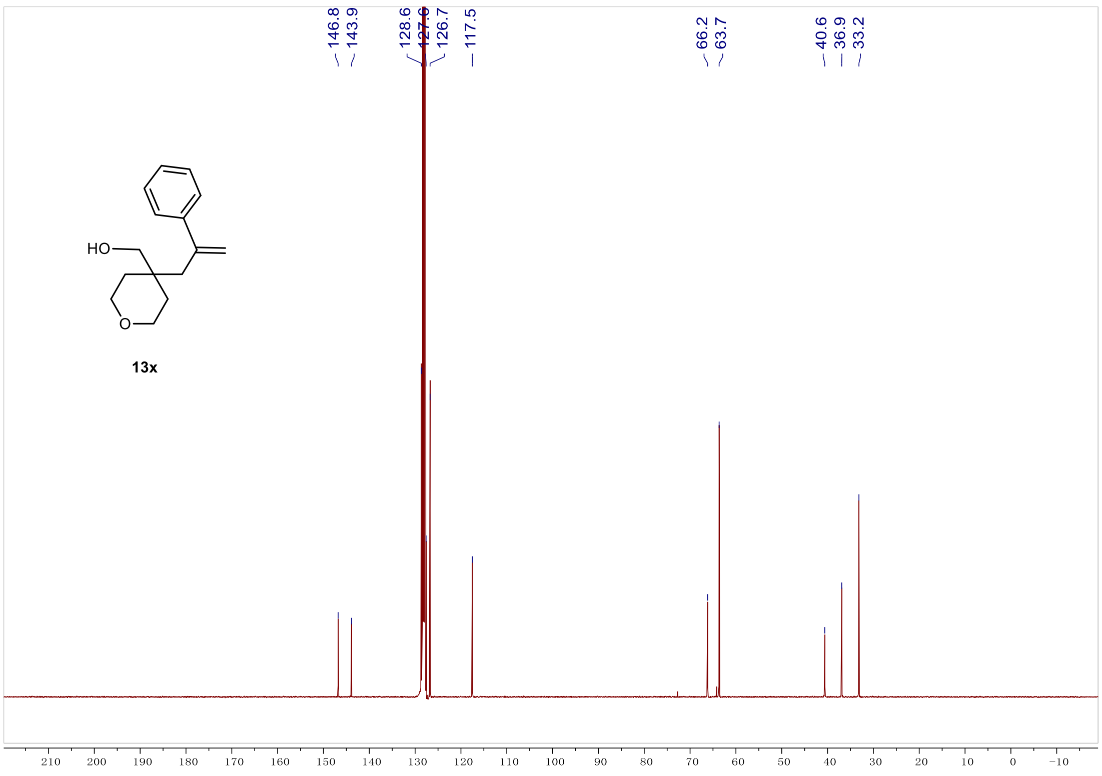

$^1$H NMR (400 MHz, $C_6D_6$)

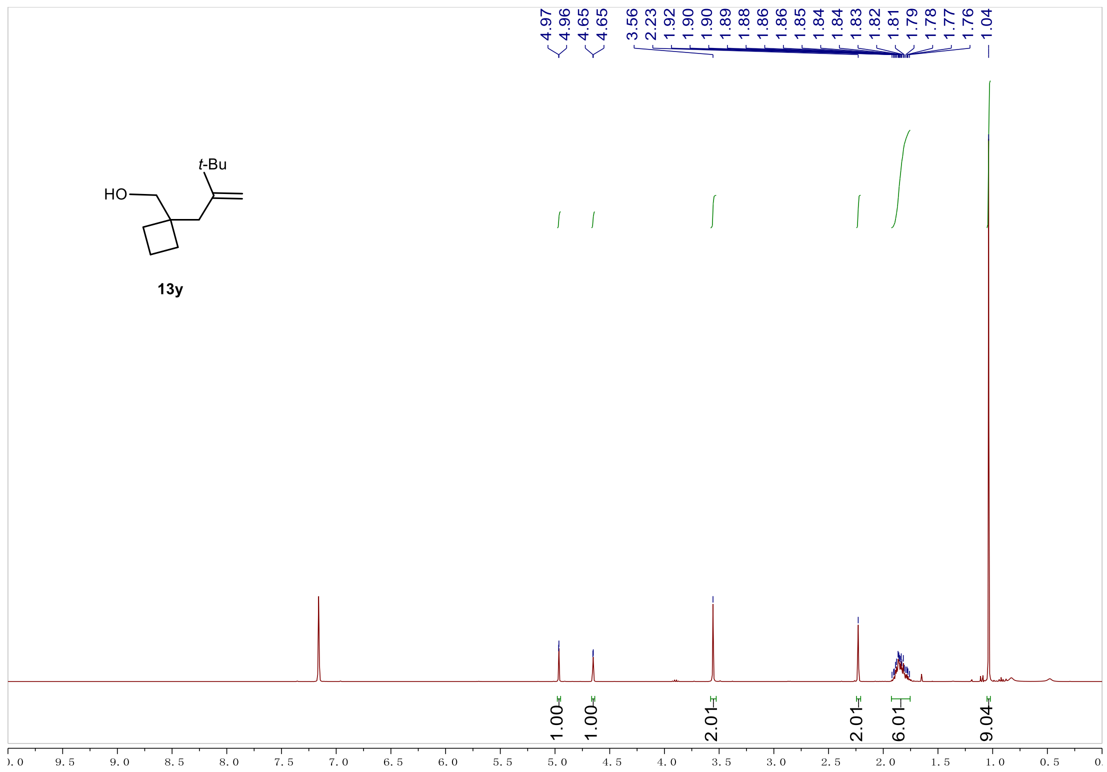


$^{13}$C NMR (101 MHz, $C_6D_6$)

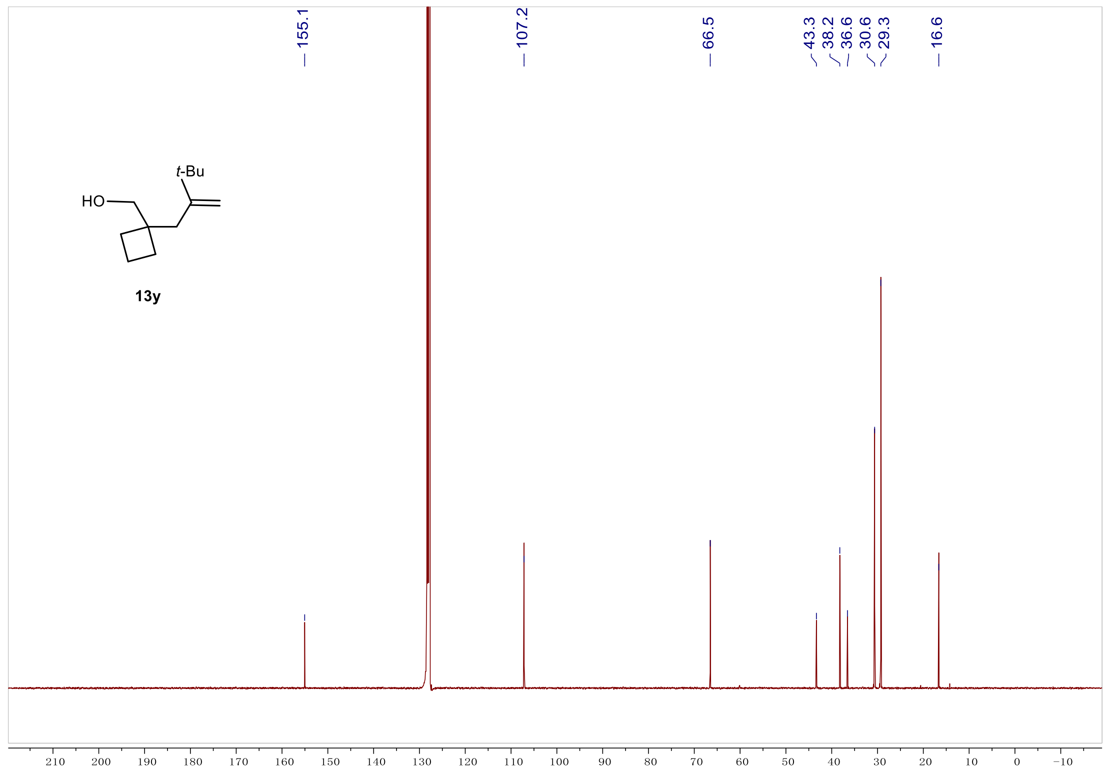

$^{1}H$ NMR (400 MHz, $C_6D_6$)

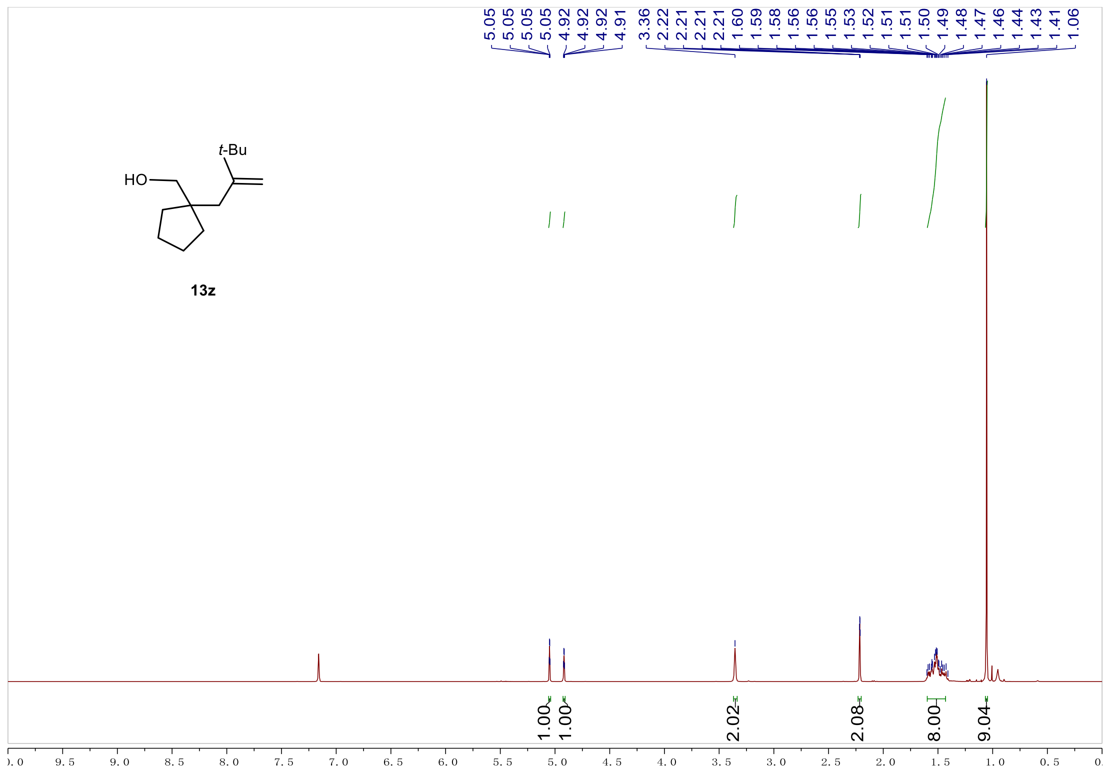


$^{13}C$ NMR (101 MHz, $C_6D_6$)

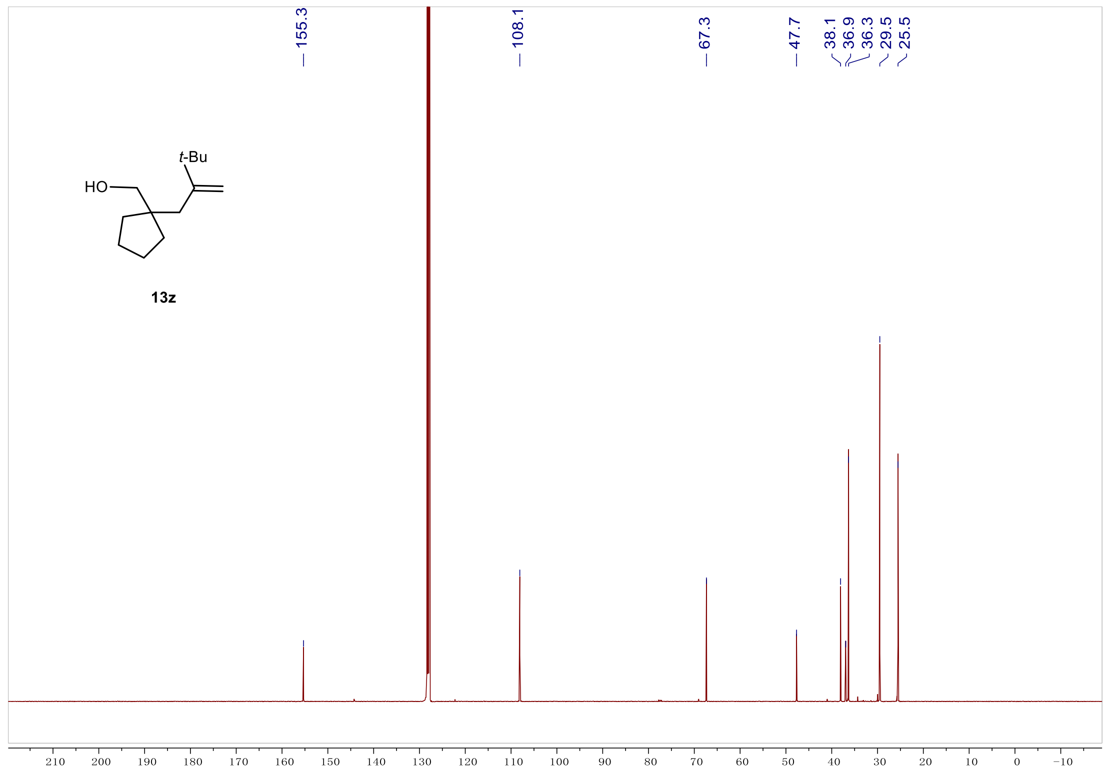

$^1H$ NMR (400 MHz, $C_6D_6$)

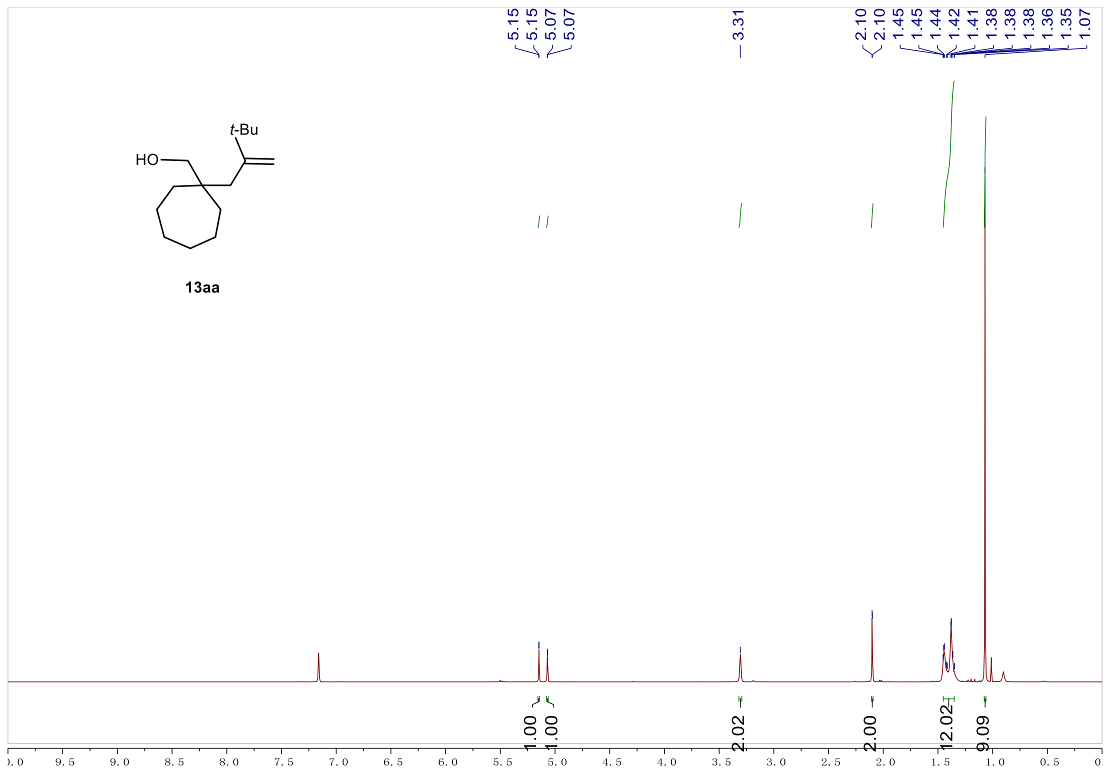


$^{13}C$ NMR (101 MHz, $C_6D_6$)

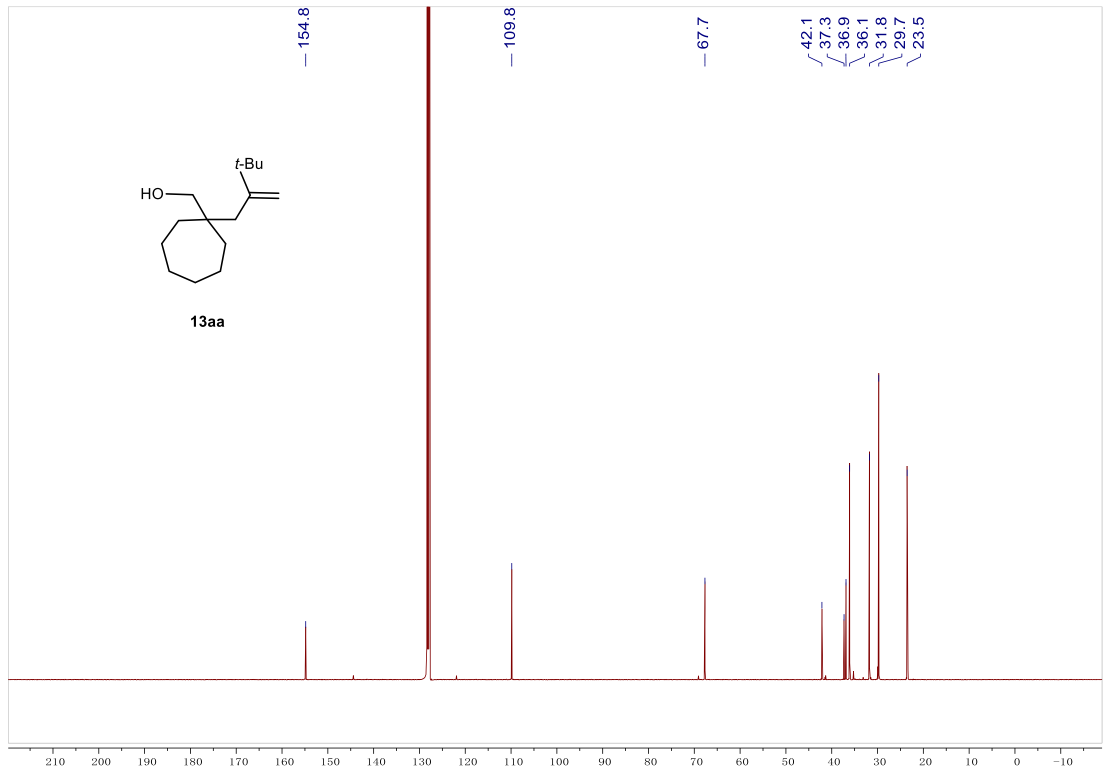

$^{1}H$ NMR (400 MHz, $C_6D_6$)

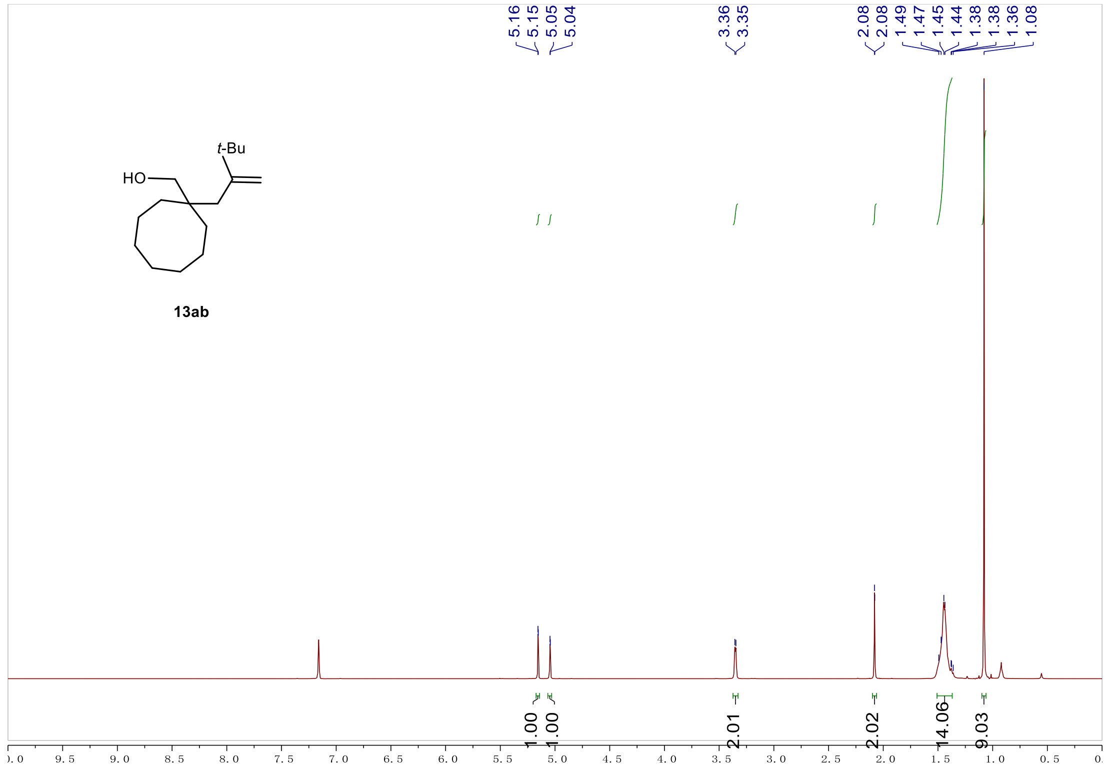


$^{13}C$ NMR (101 MHz, $C_6D_6$)

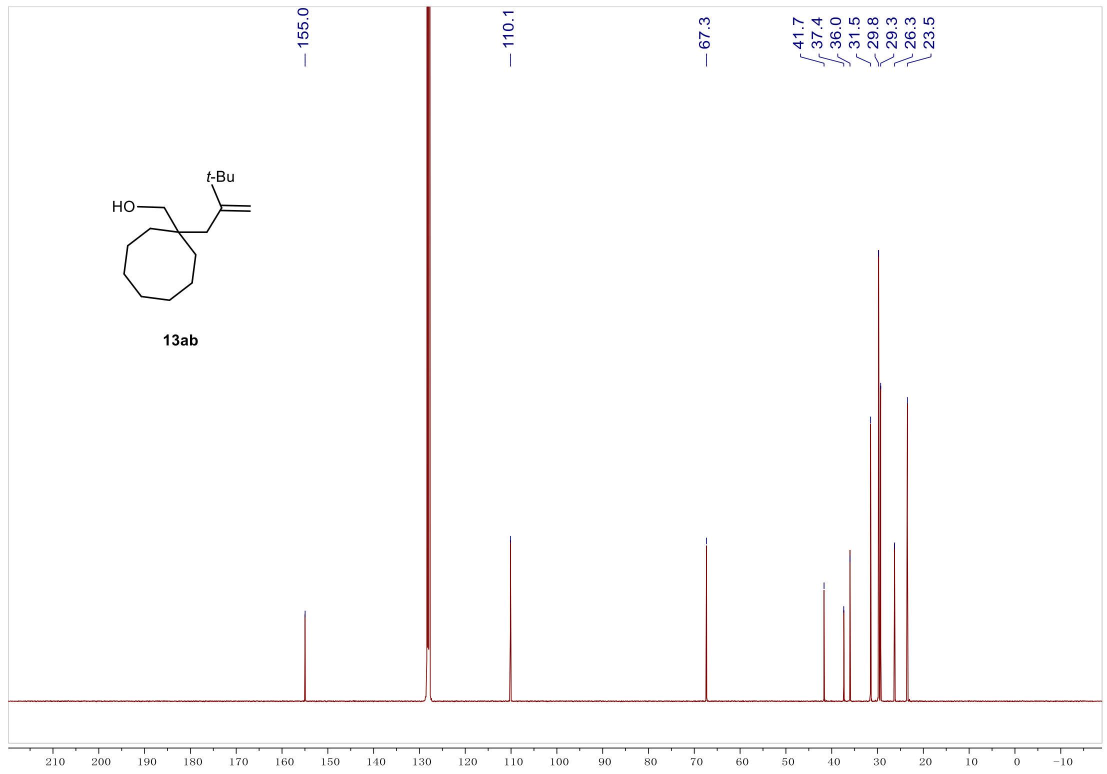

$^{1}$H NMR (500 MHz, $CDCl_3$)

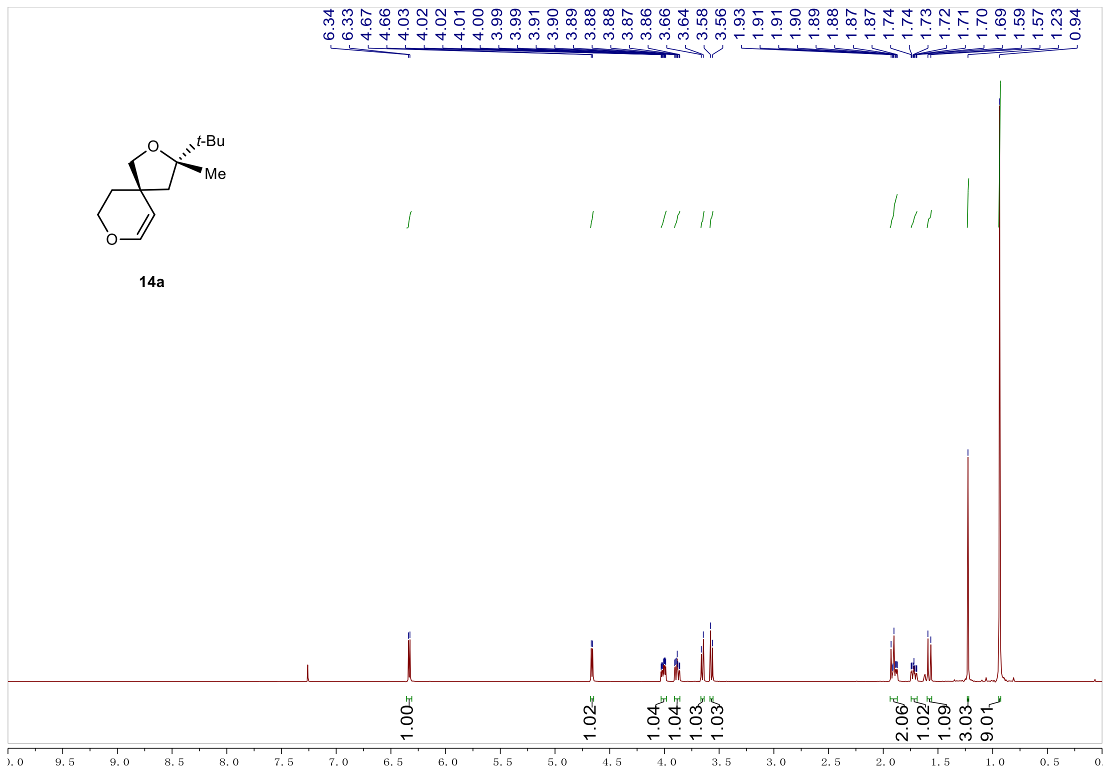


$^{13}$C NMR (126 MHz, $CDCl_3$)

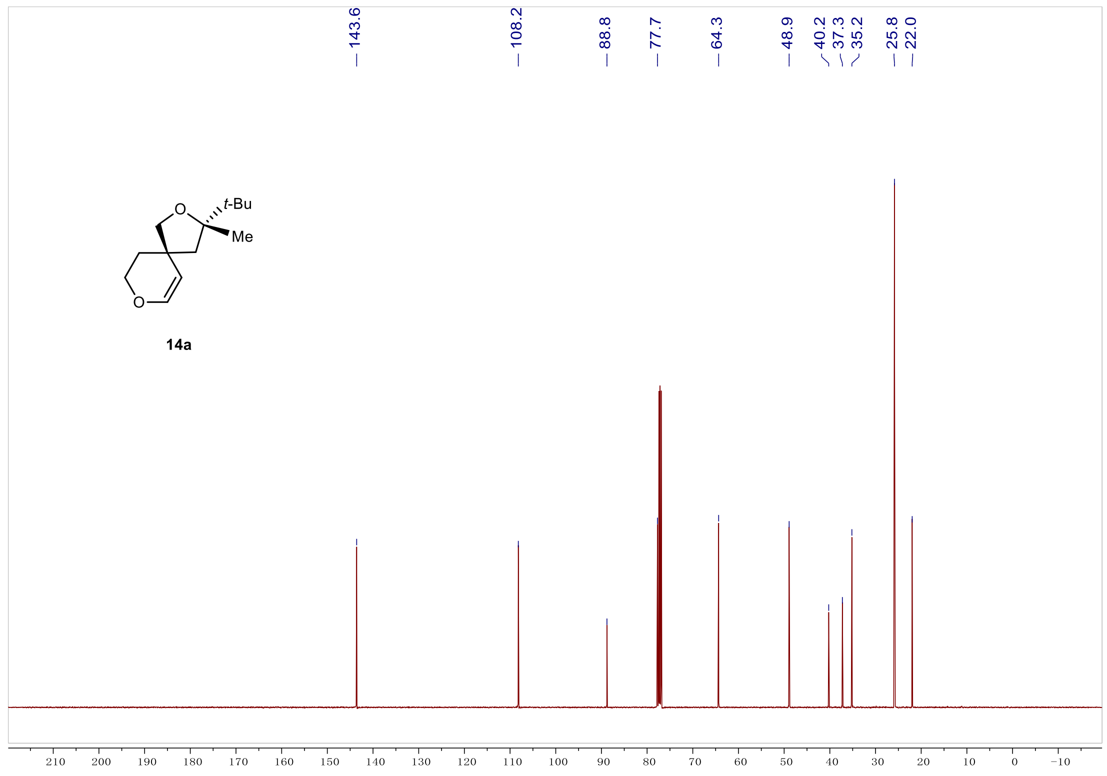

$^1H$ NMR (400 MHz, $CDCl_3$)

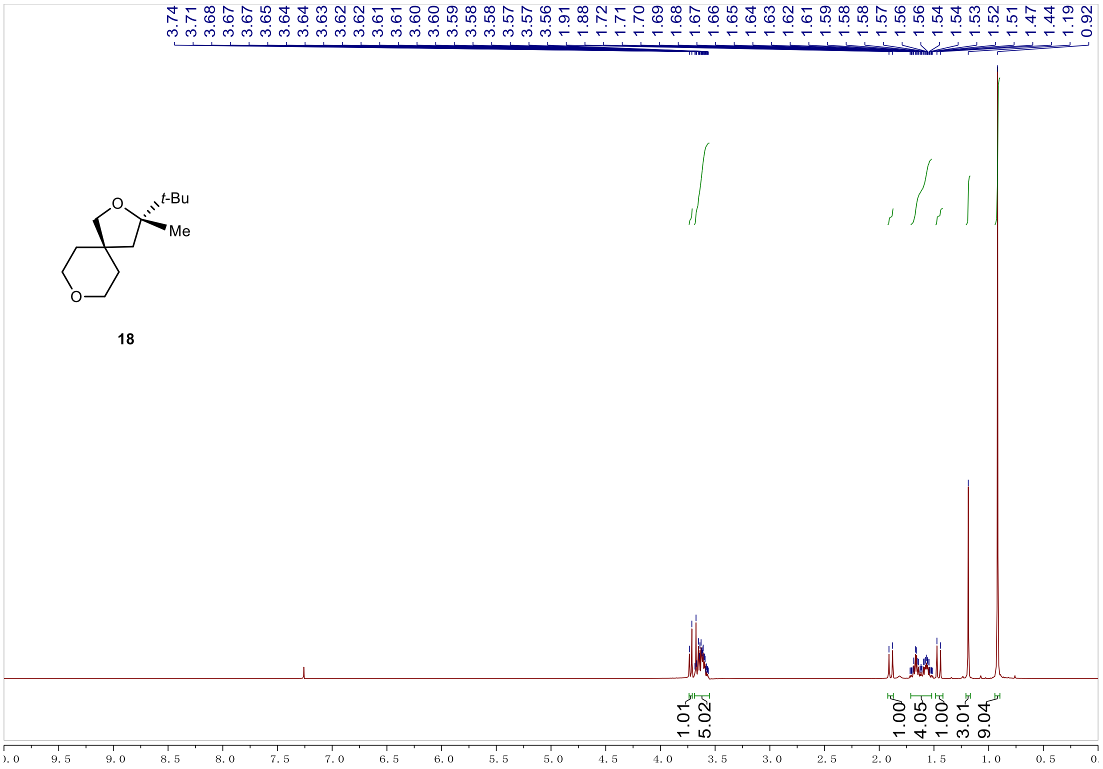


$^{13}C$ NMR (101 MHz, $CDCl_3$)

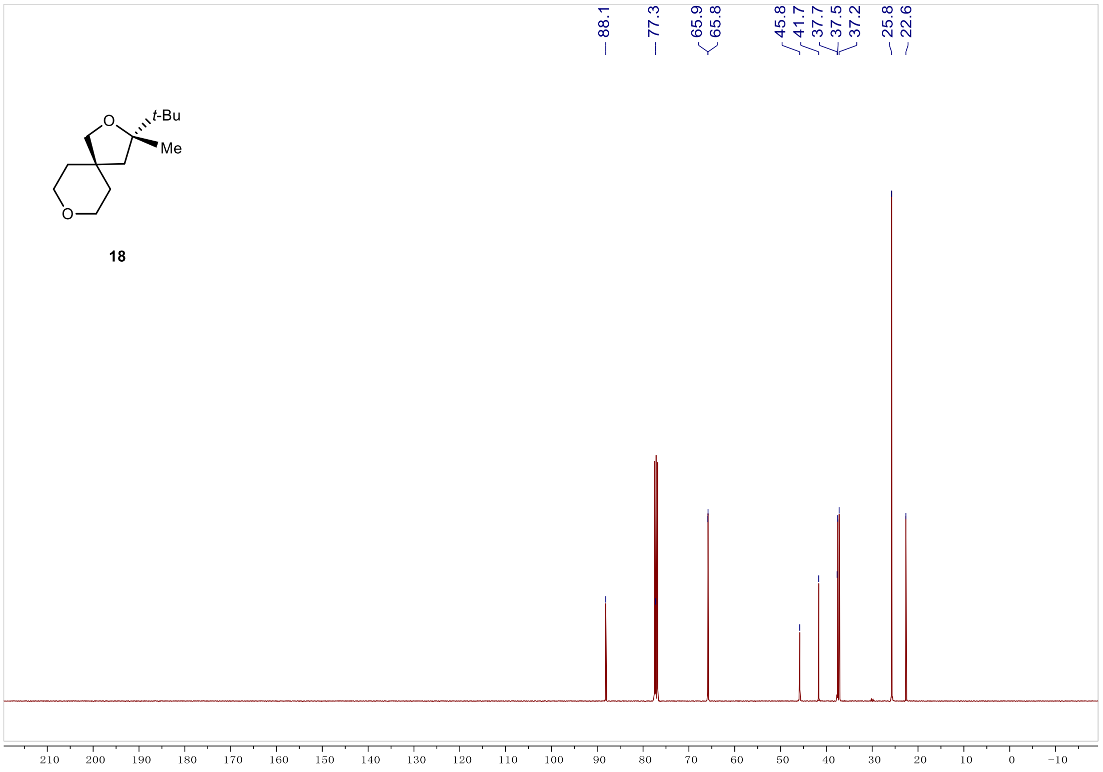

$^{1}H$ NMR (400 MHz, $CDCl_3$)

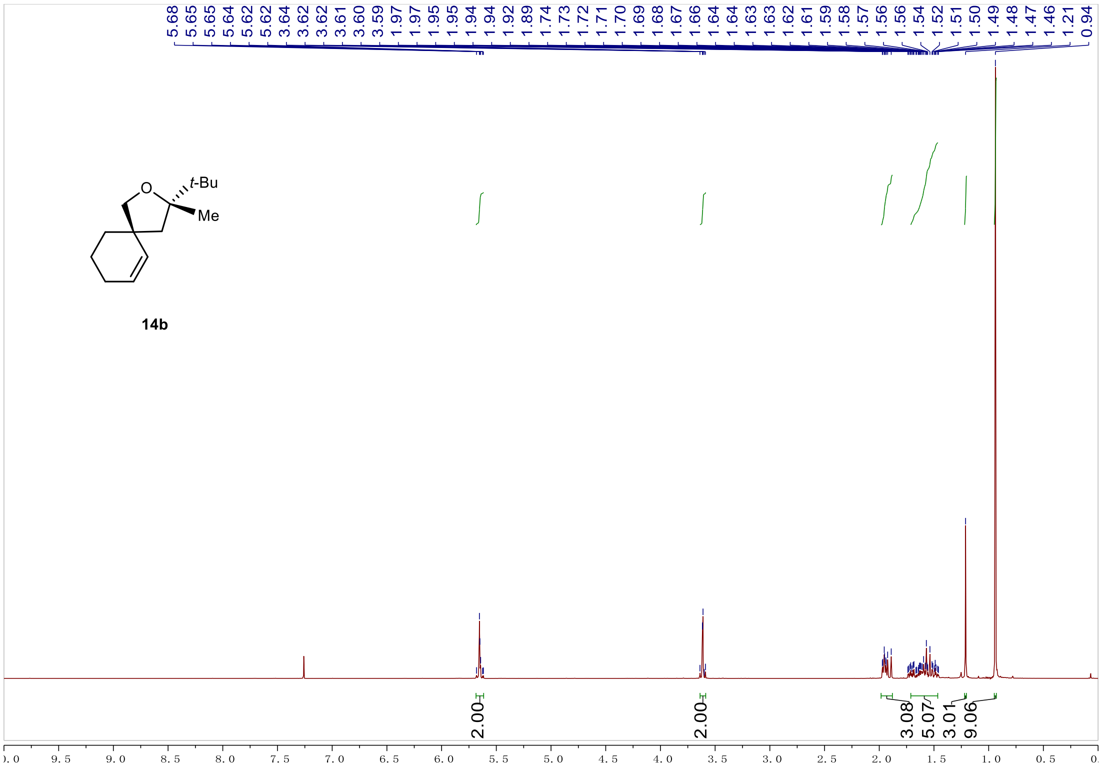


$^{13}C$ NMR (101 MHz, $CDCl_3$)

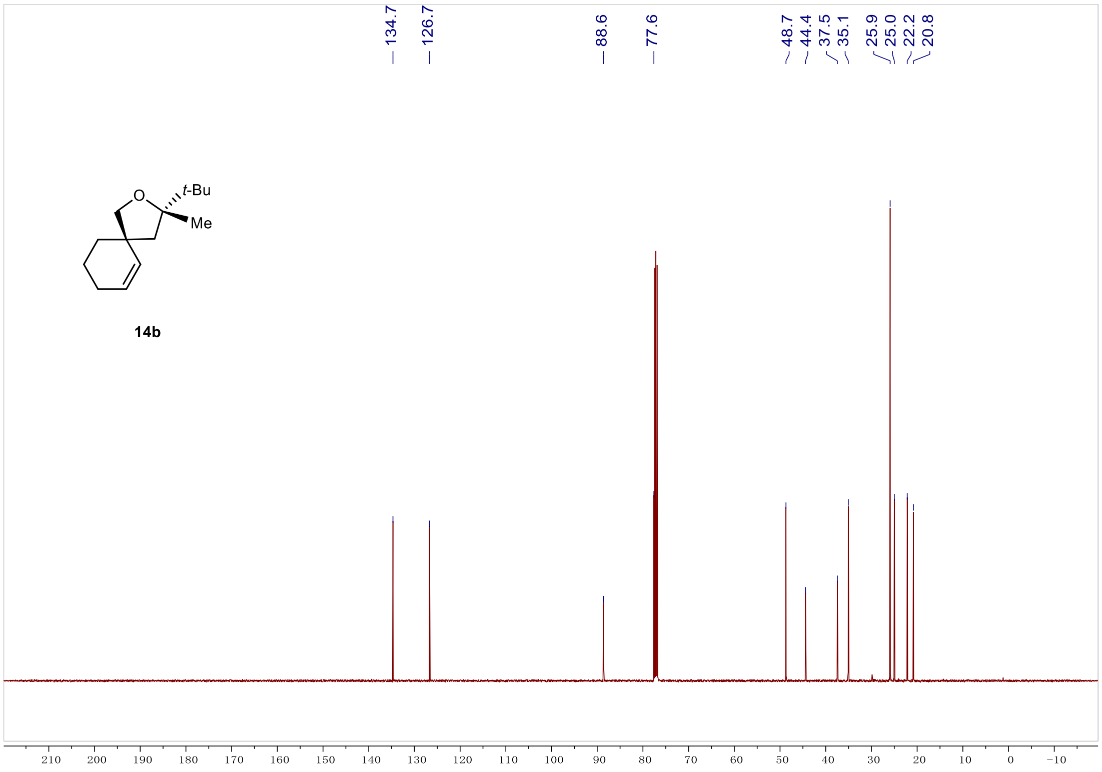

$^{1}H$ NMR (400 MHz, $CDCl_3$)

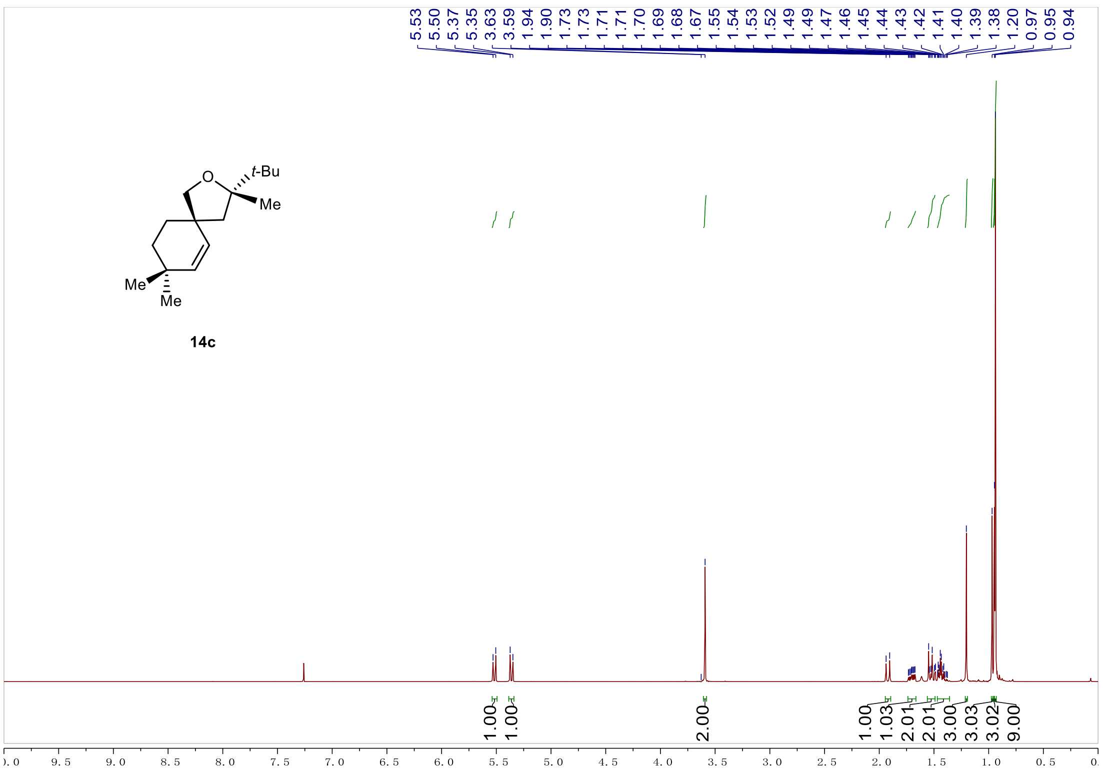


$^{13}C$ NMR (101 MHz, $CDCl_3$)

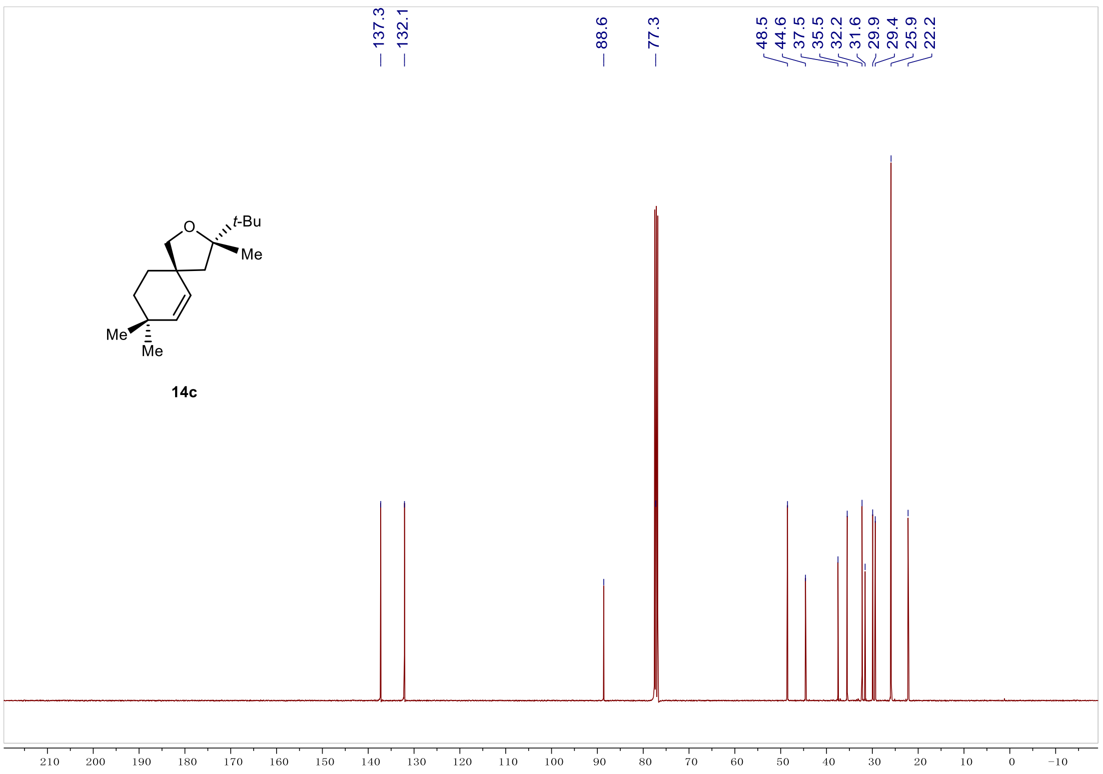

$^{1}H$ NMR (400 MHz, $CDCl_3$)

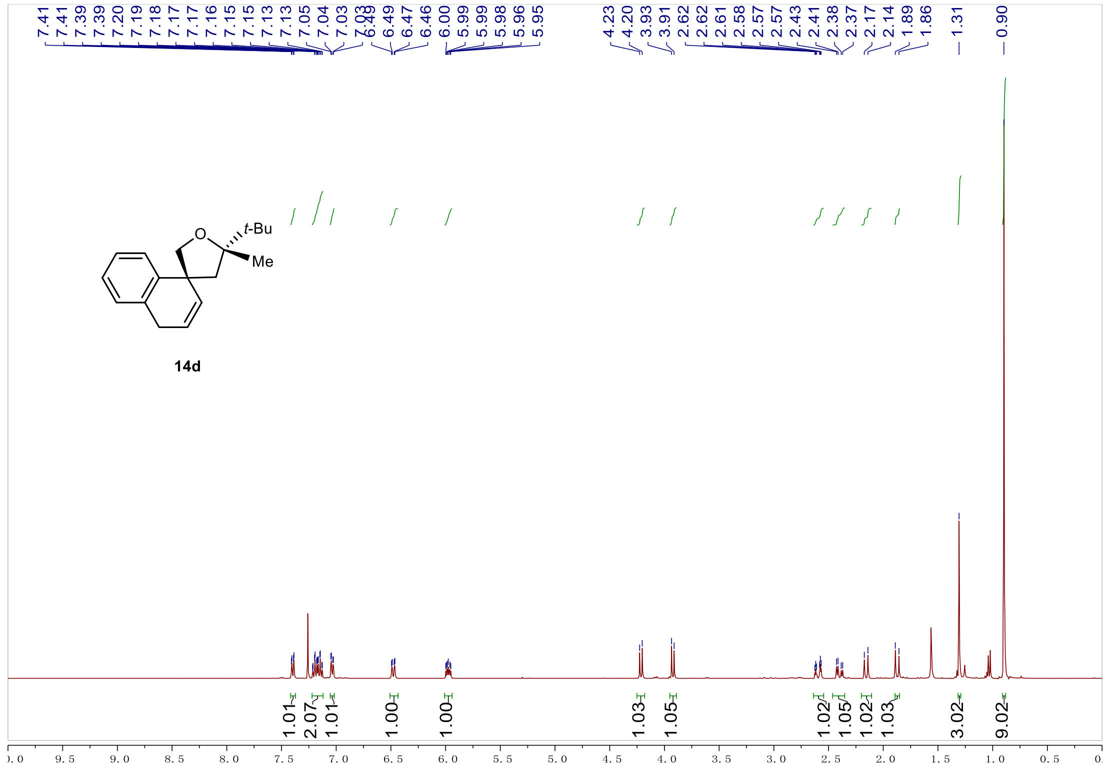


$^{13}C$ NMR (101 MHz, $CDCl_3$)

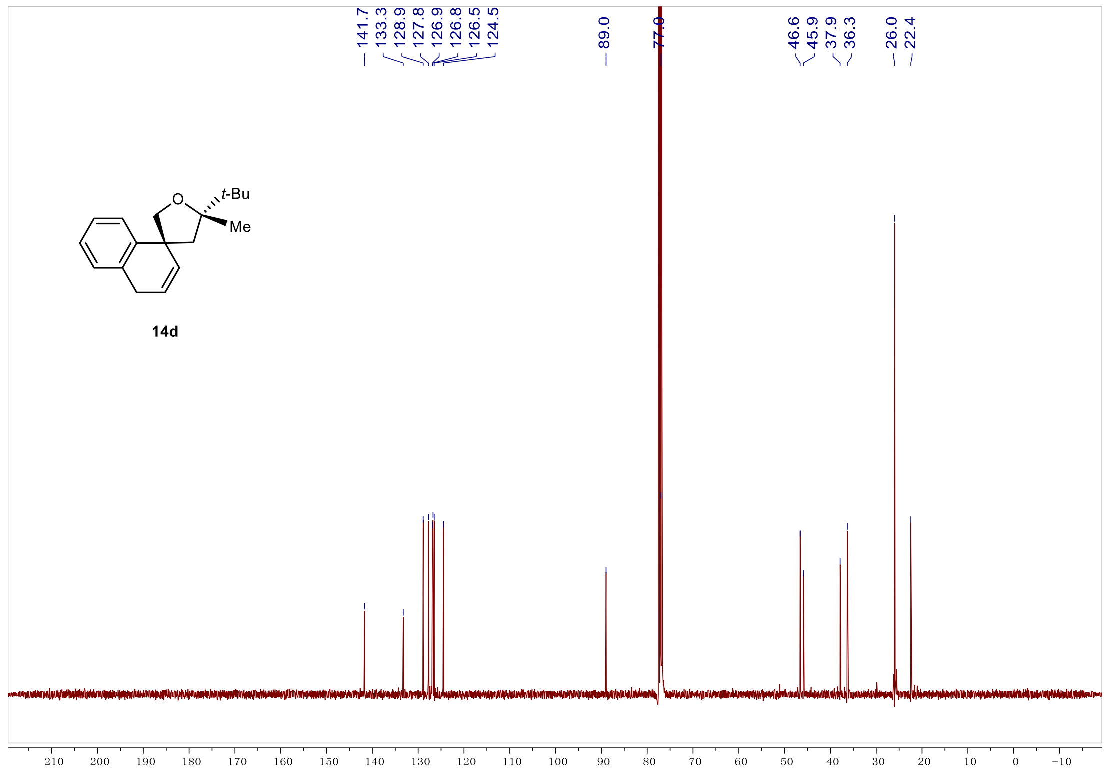

$^{1}$H NMR (400 MHz, $CDCl_3$)

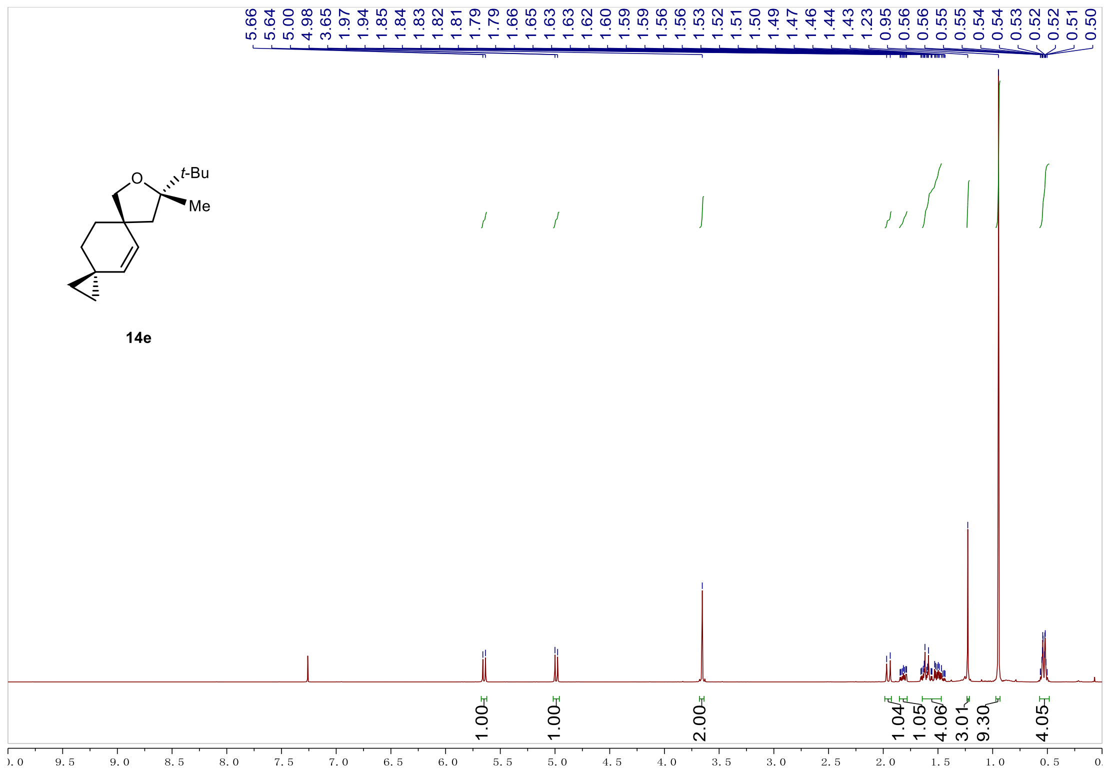


$^{13}$C NMR (101 MHz, $CDCl_3$)

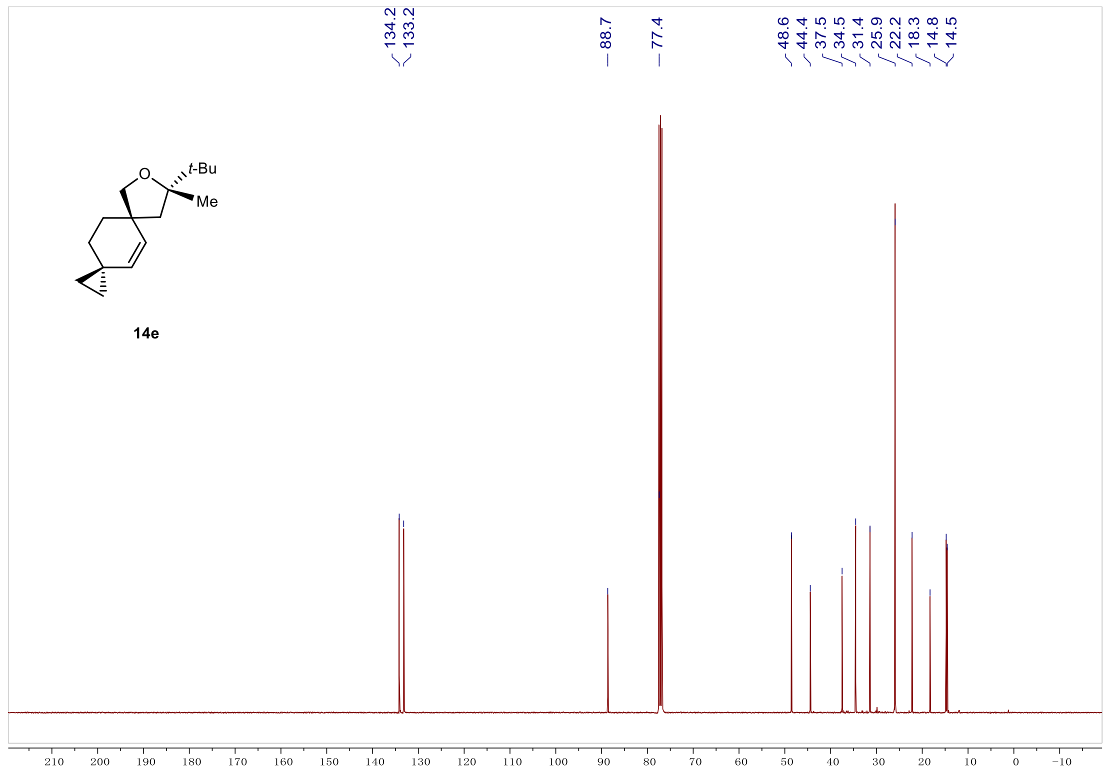

$^{1}H$ NMR (400 MHz, $CDCl_3$)

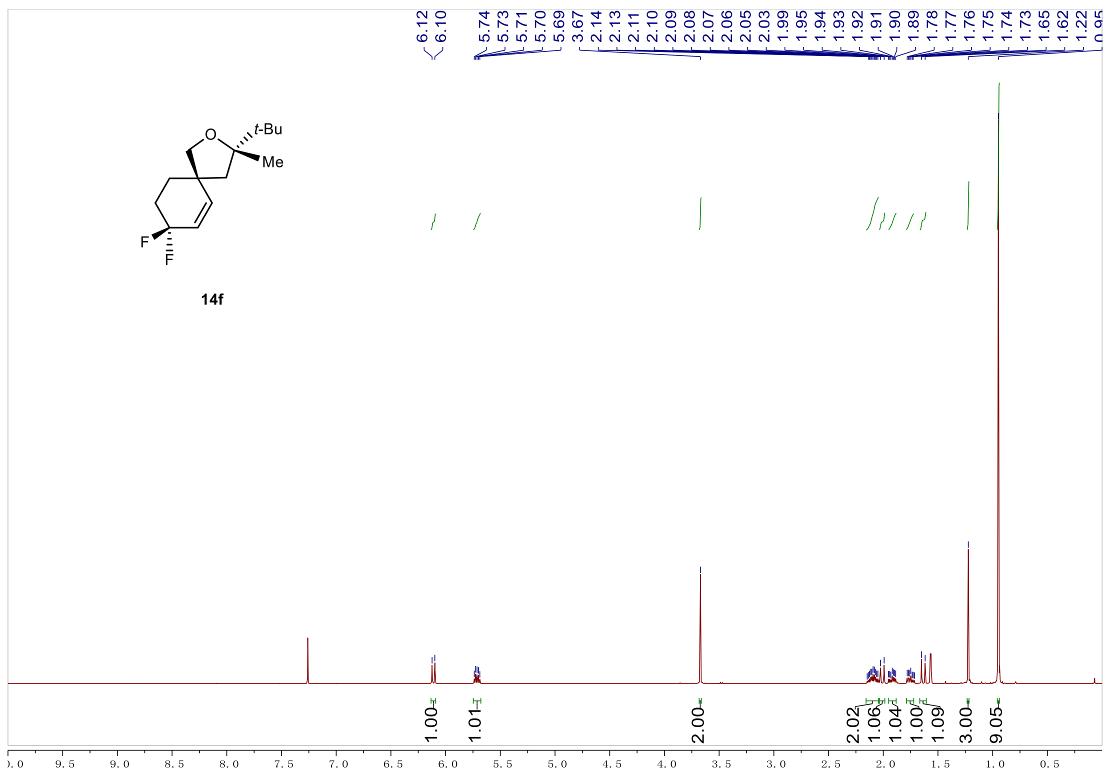

$^{13}C$ NMR (101 MHz, $CDCl_3$)

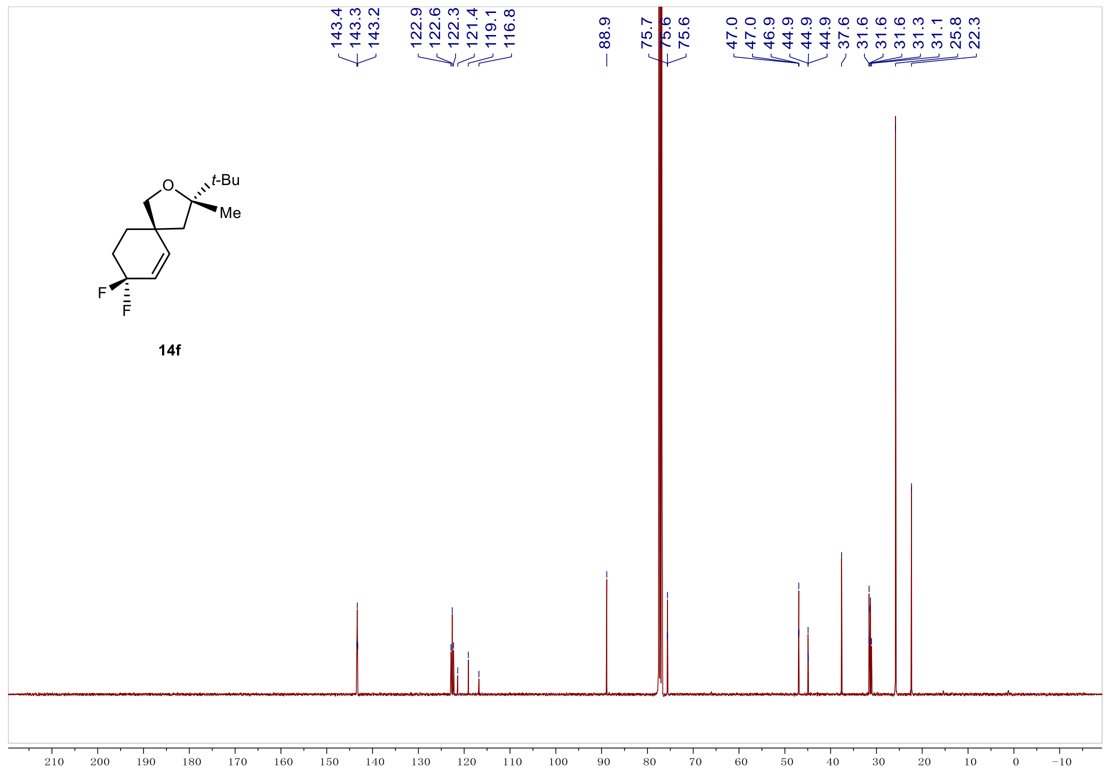

$^{19}F$ NMR (471 MHz, $C_6D_6$)

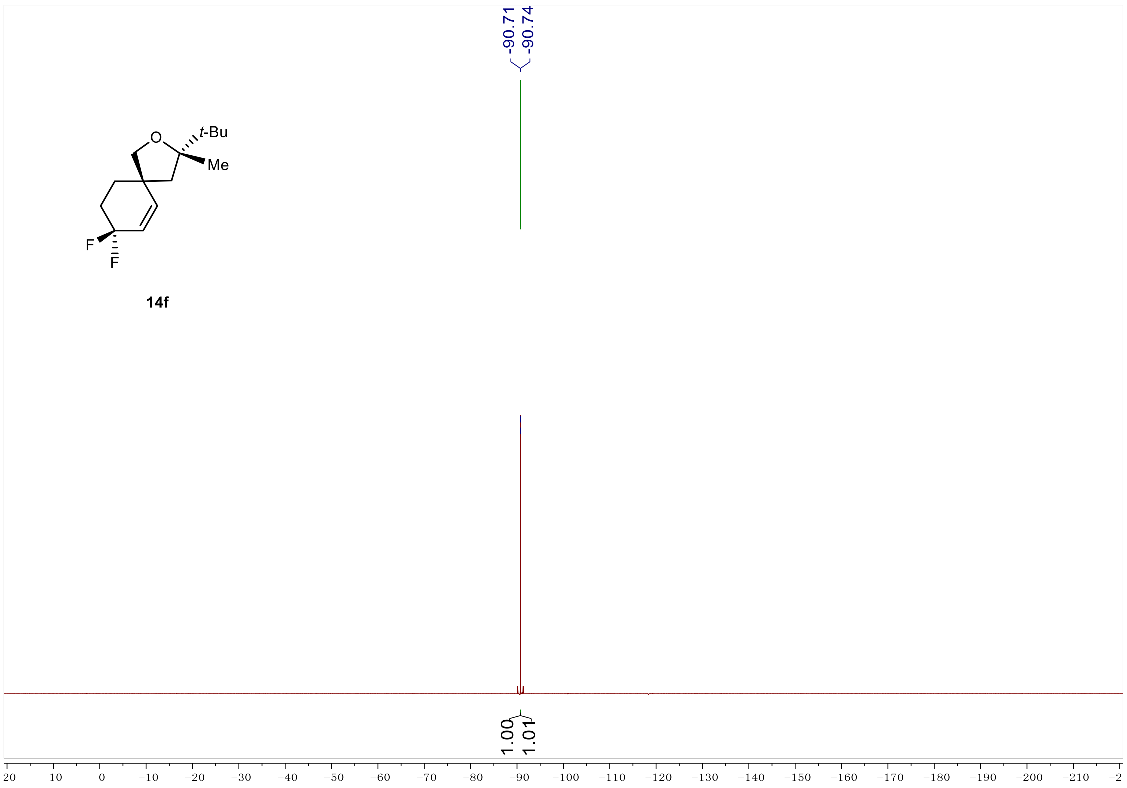

$^{1}H$ NMR (400 MHz, $CDCl_3$)

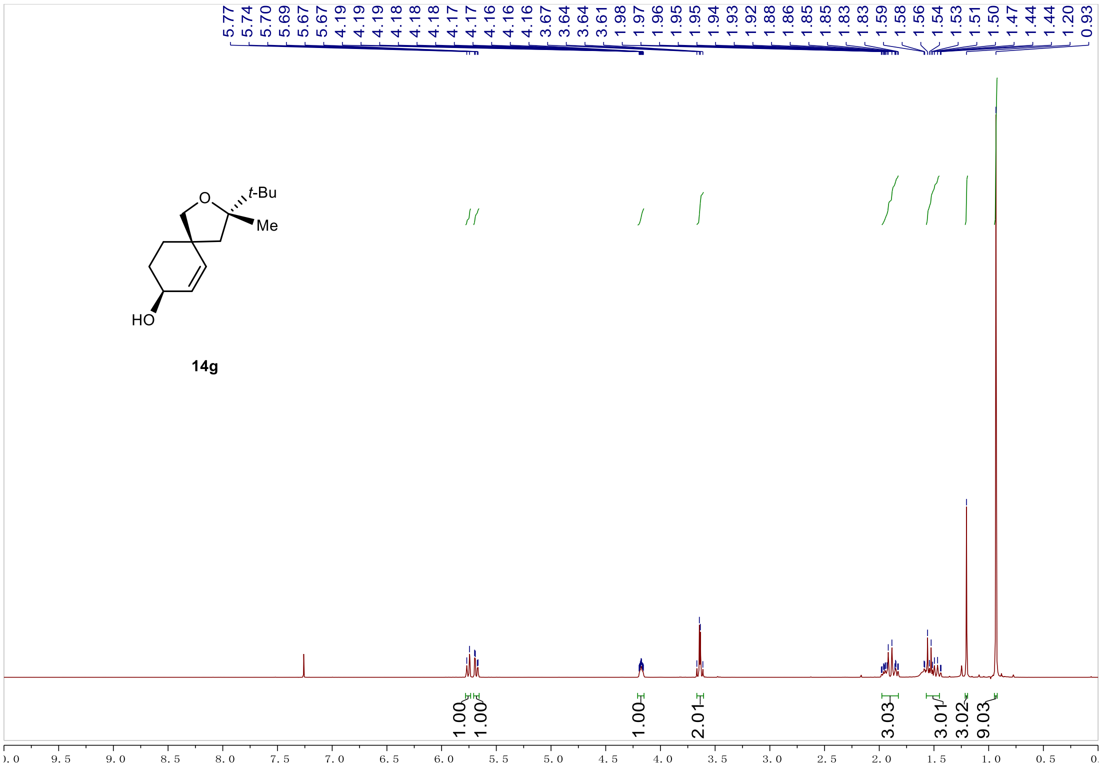


$^{13}C$ NMR (101 MHz, $CDCl_3$)

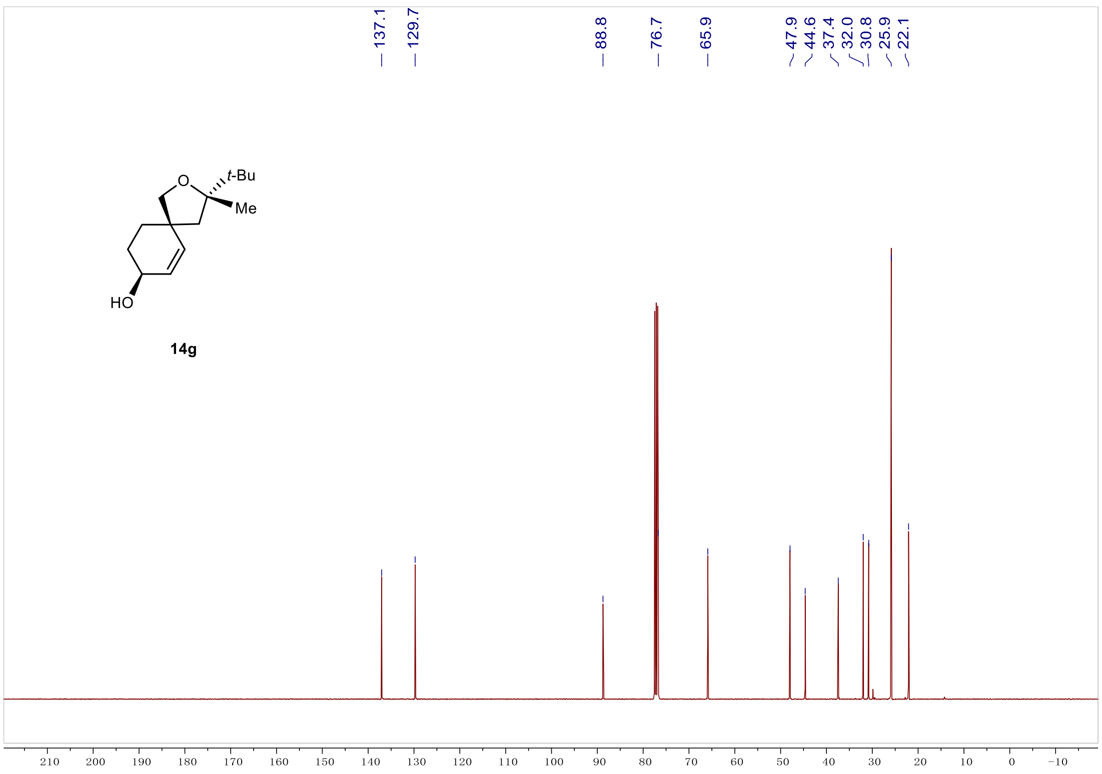

NOESY NMR (400 MHz, $CDCl_3$)

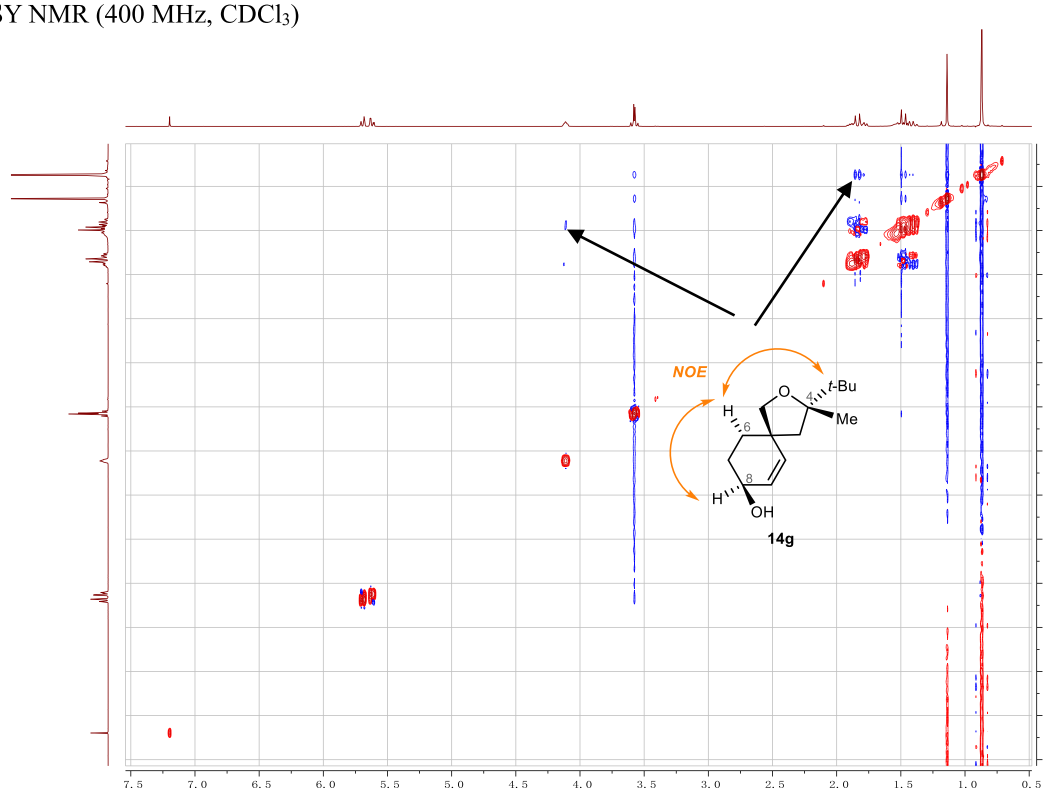

$^{1}H$ NMR (400 MHz, $CDCl_3$)

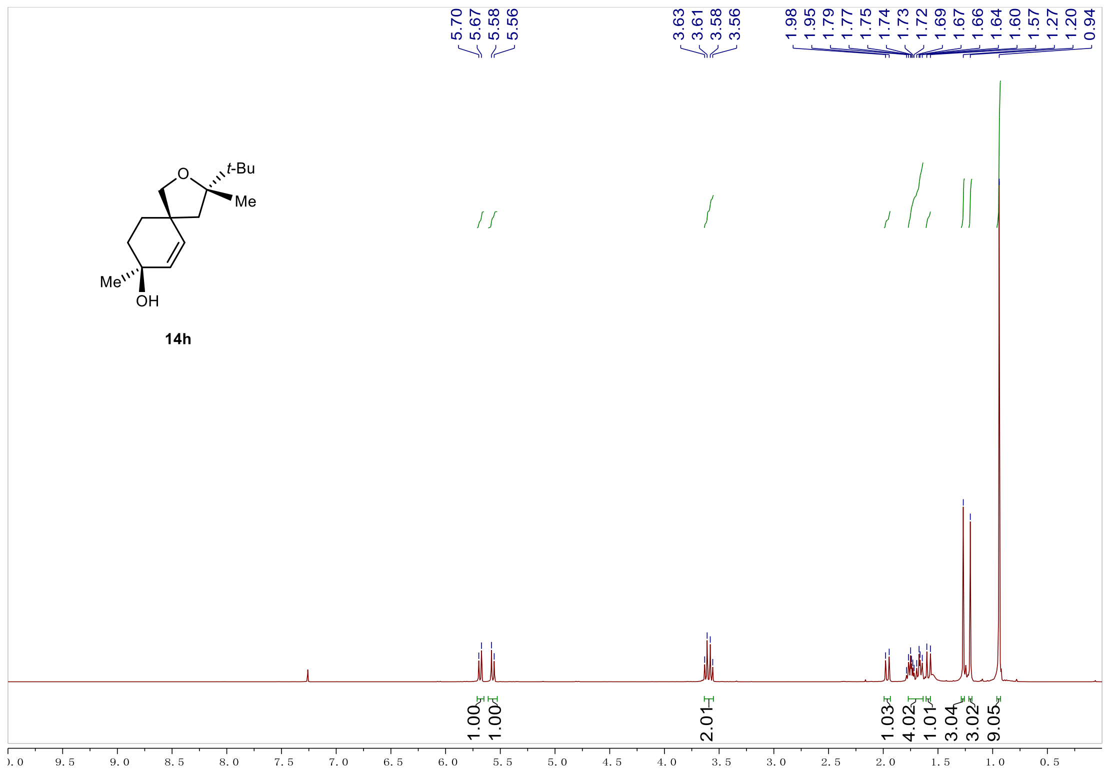


$^{13}C$ NMR (101 MHz, $CDCl_3$)

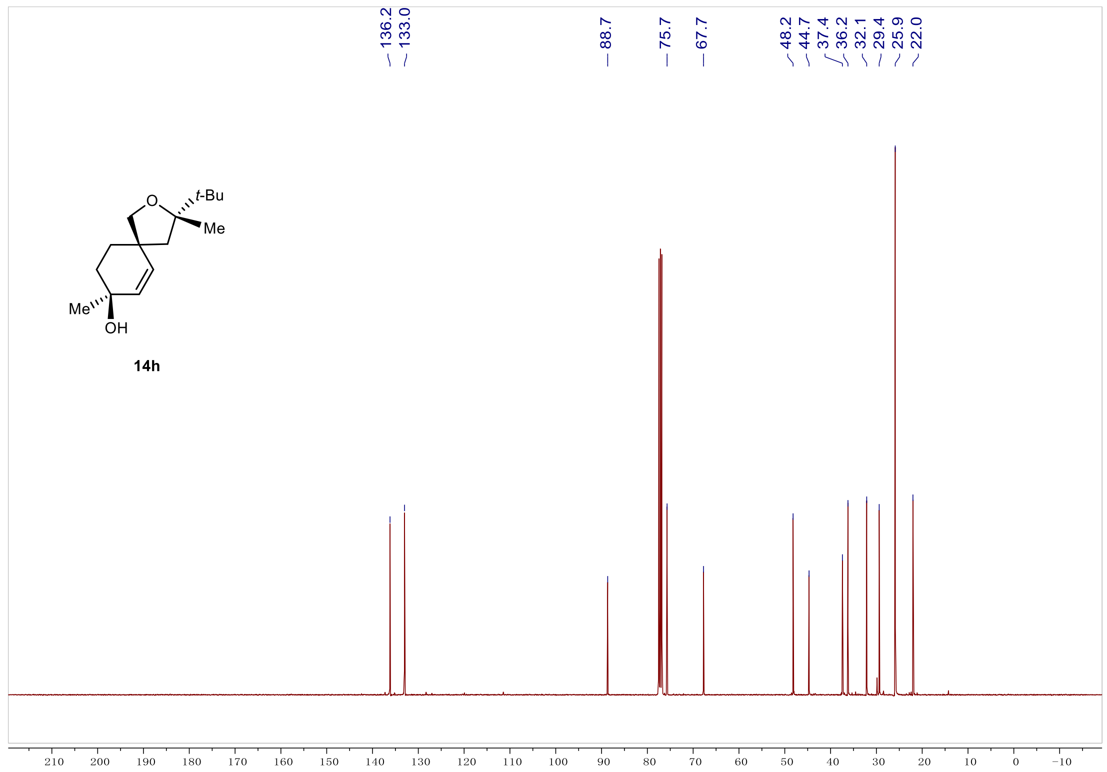

NOESY NMR (400 MHz, $CDCl_3$)

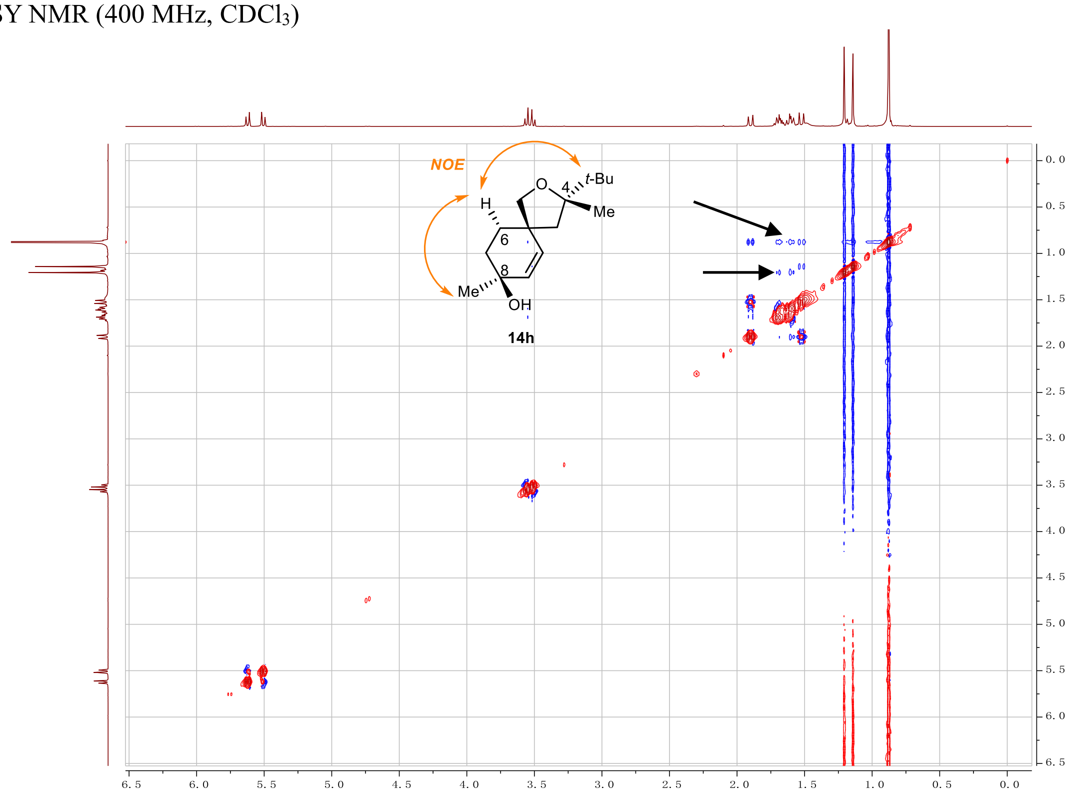

$^{1}H$ NMR (400 MHz, $CDCl_3$)

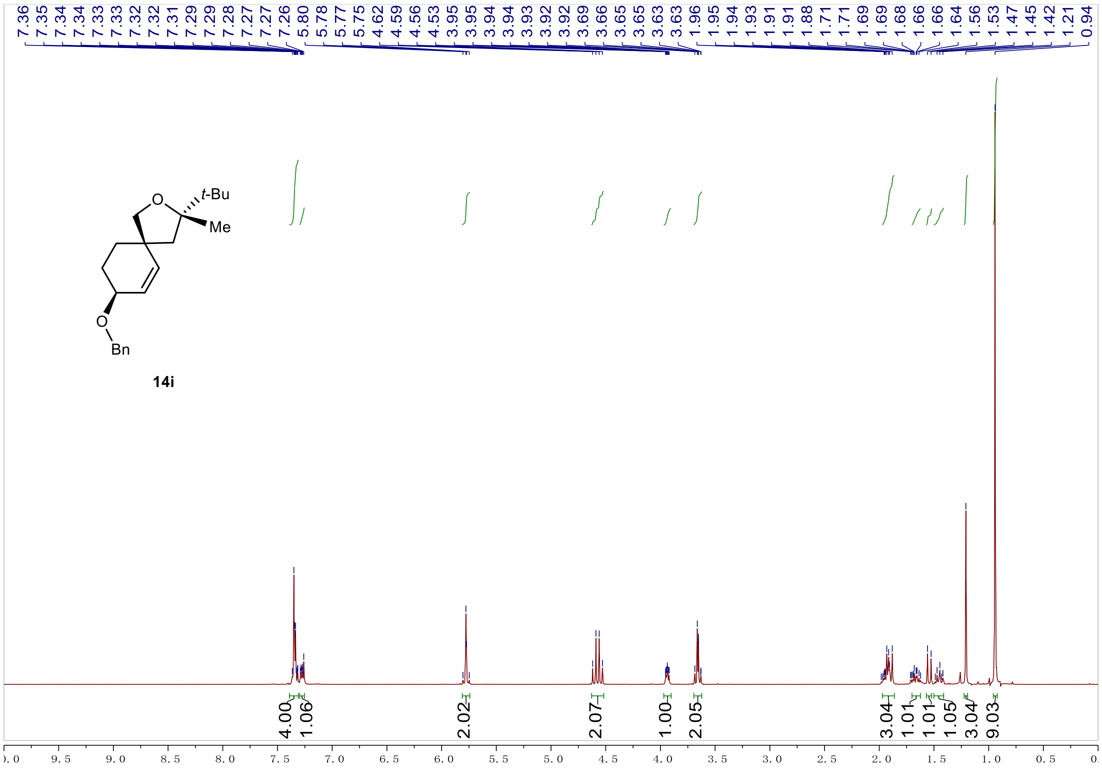


$^{13}C$ NMR (101 MHz, $CDCl_3$)

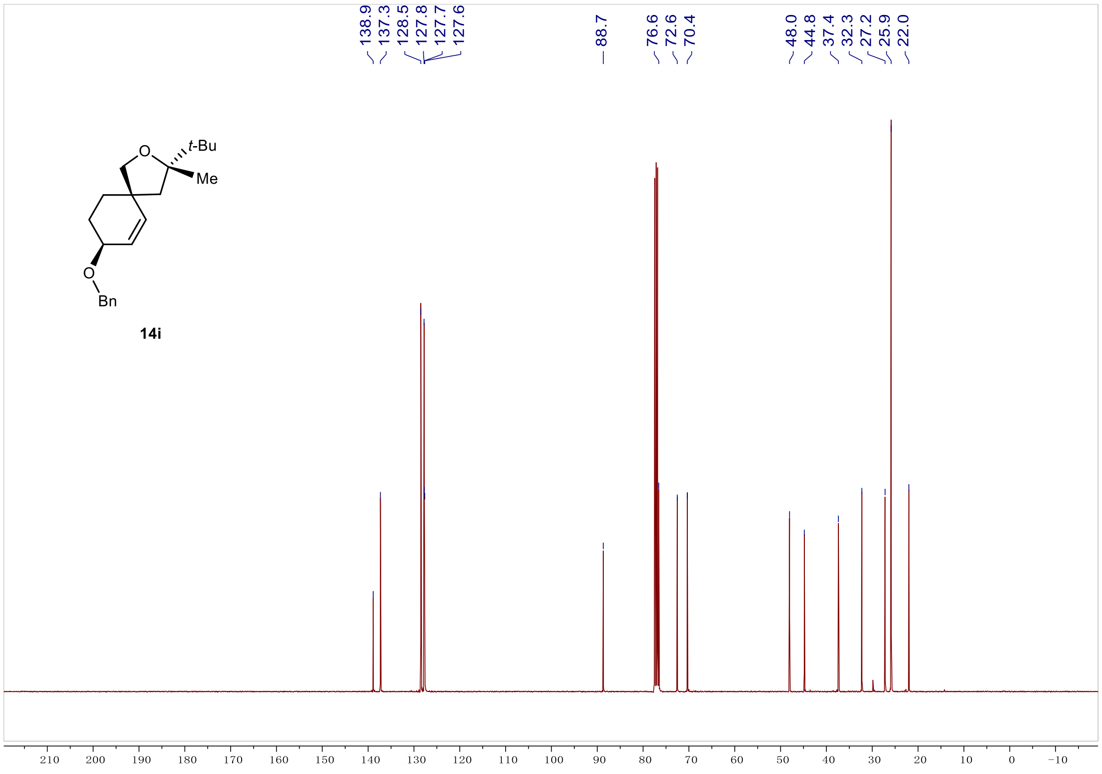

NOESY NMR (400 MHz, $CDCl_3$)

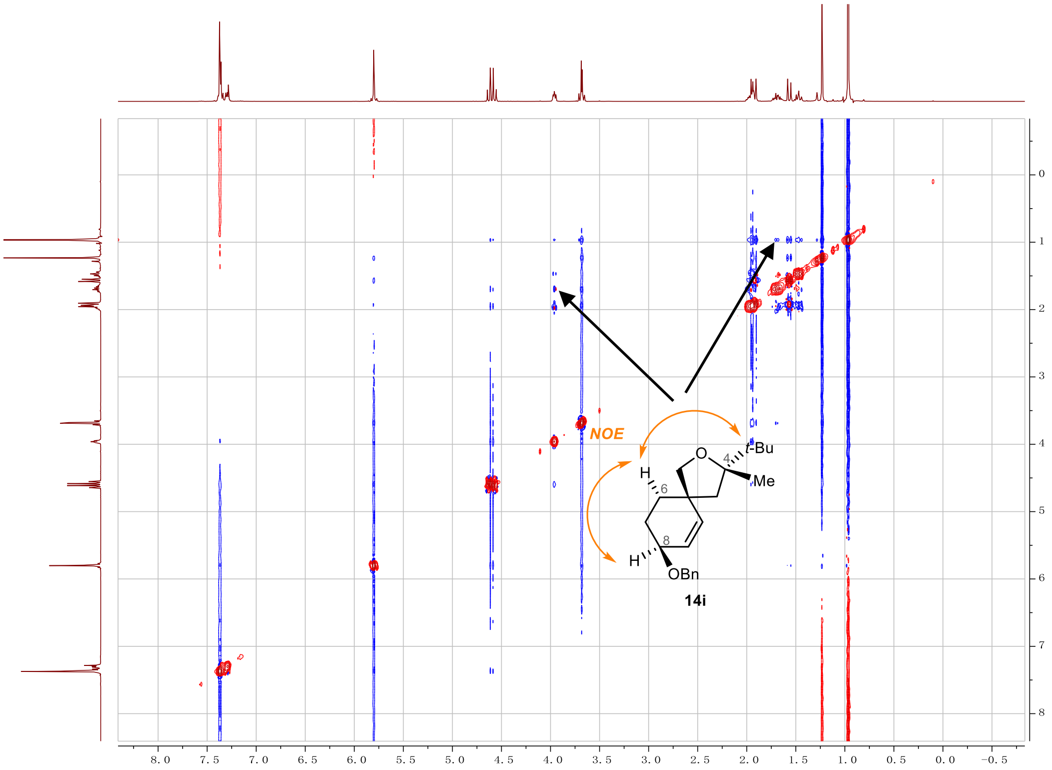

$^{1}H$ NMR (400 MHz, $C_6D_6$)

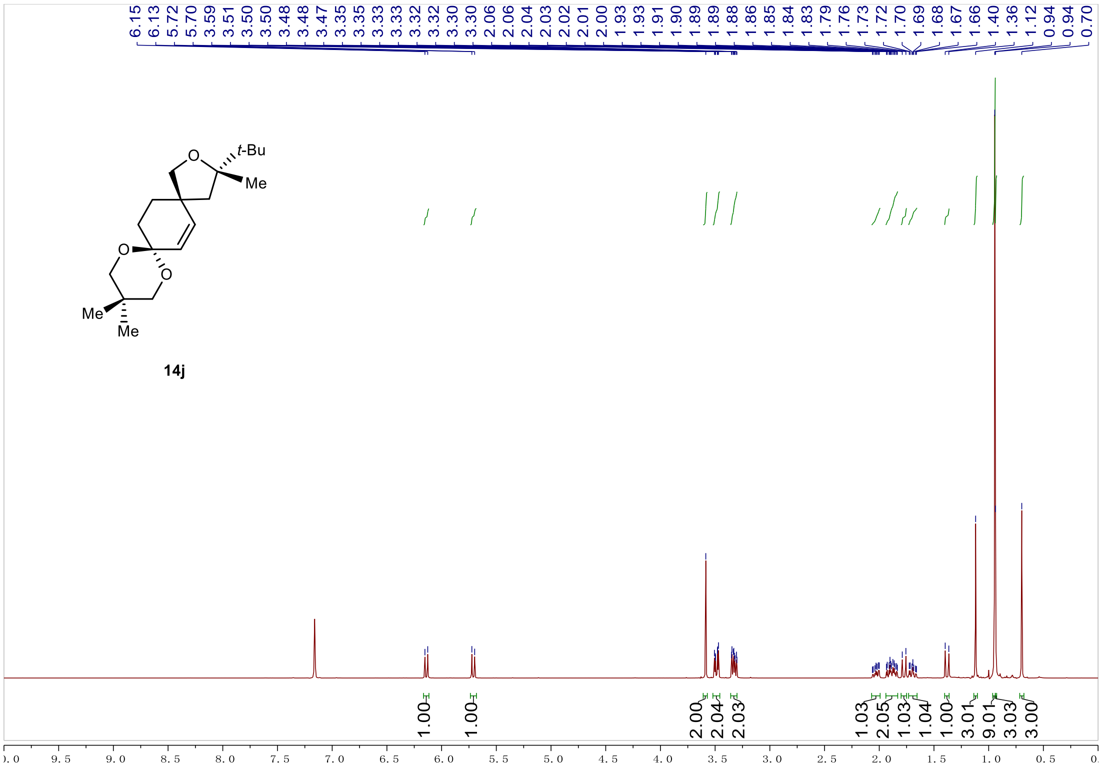

$^{13}C$ NMR (101 MHz, $C_6D_6$)

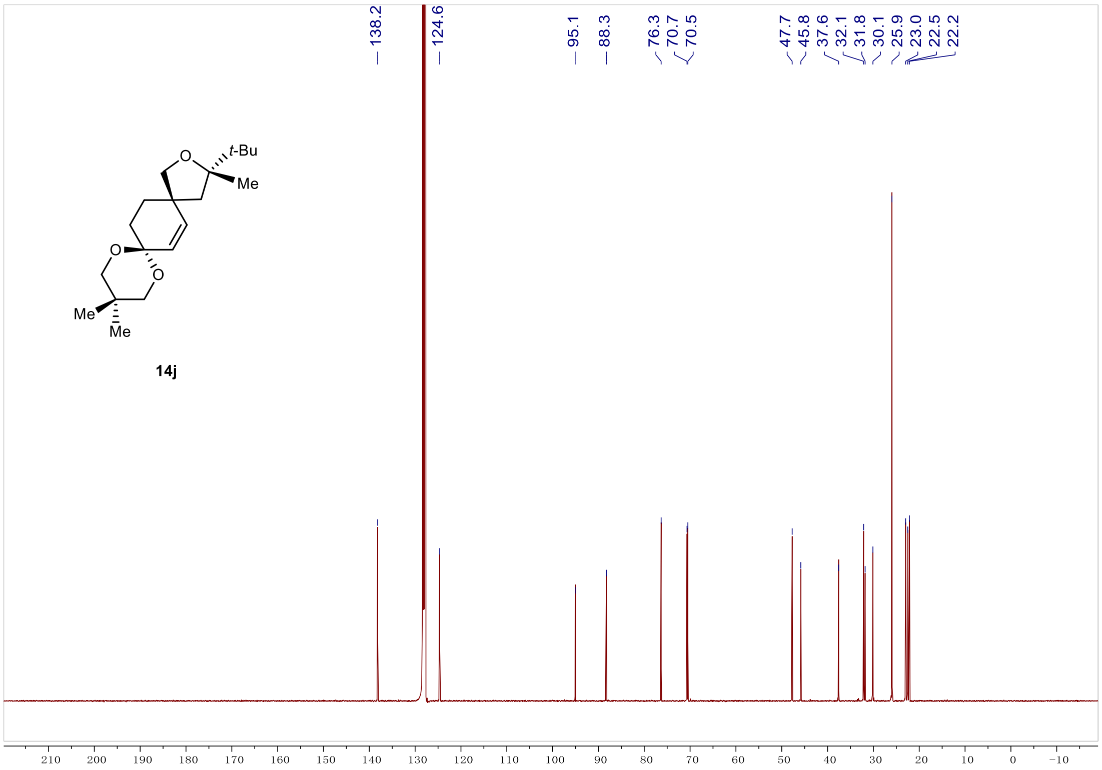

$^{1}H$ NMR (400 MHz, $CDCl_3$)

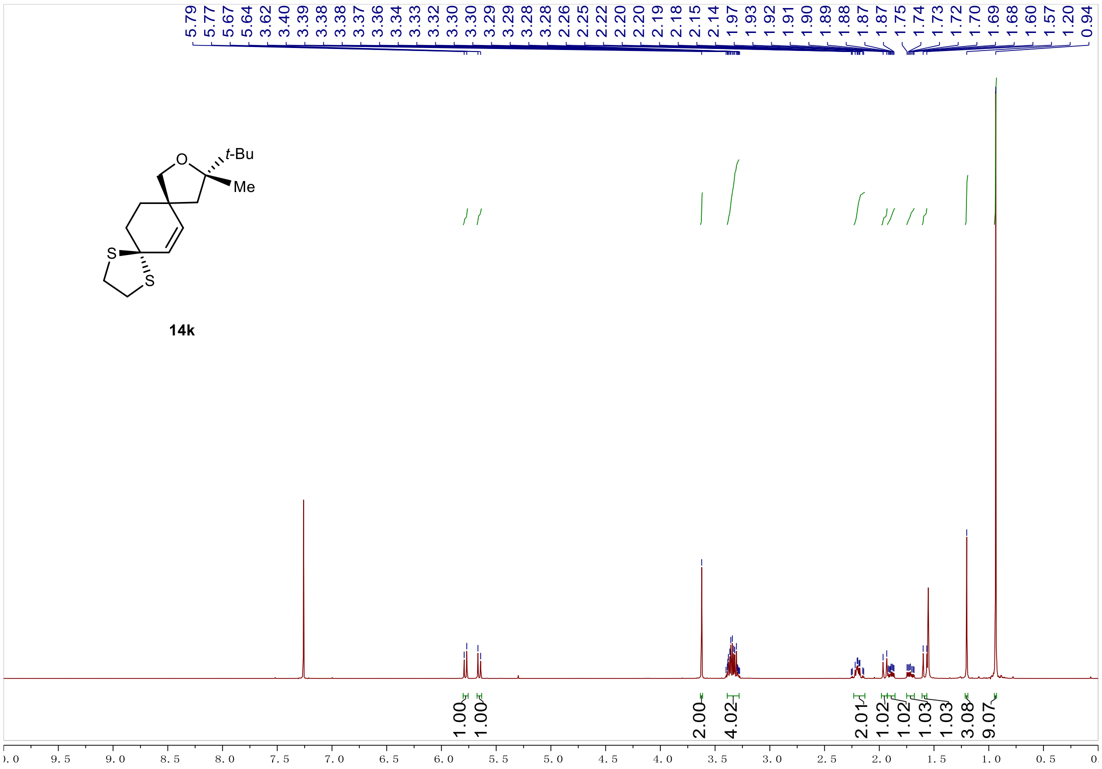

$^{13}C$ NMR (101 MHz, $CDCl_3$)

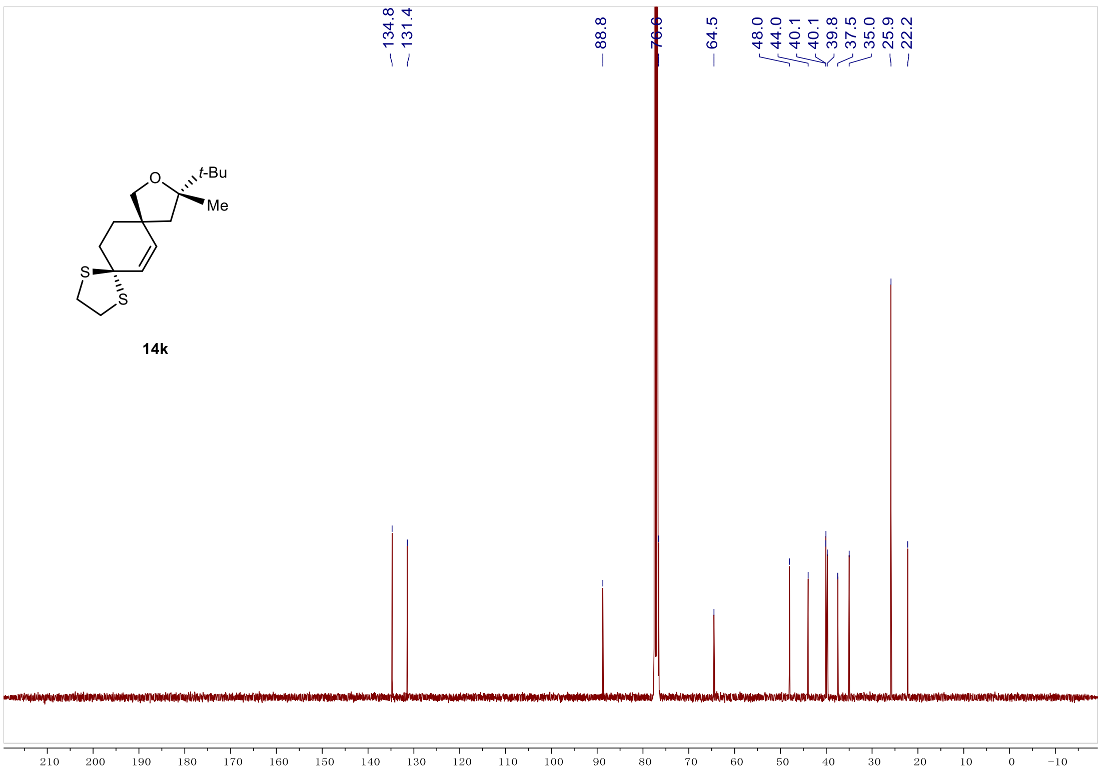

$^{1}H$ NMR (400 MHz, $CDCl_3$)

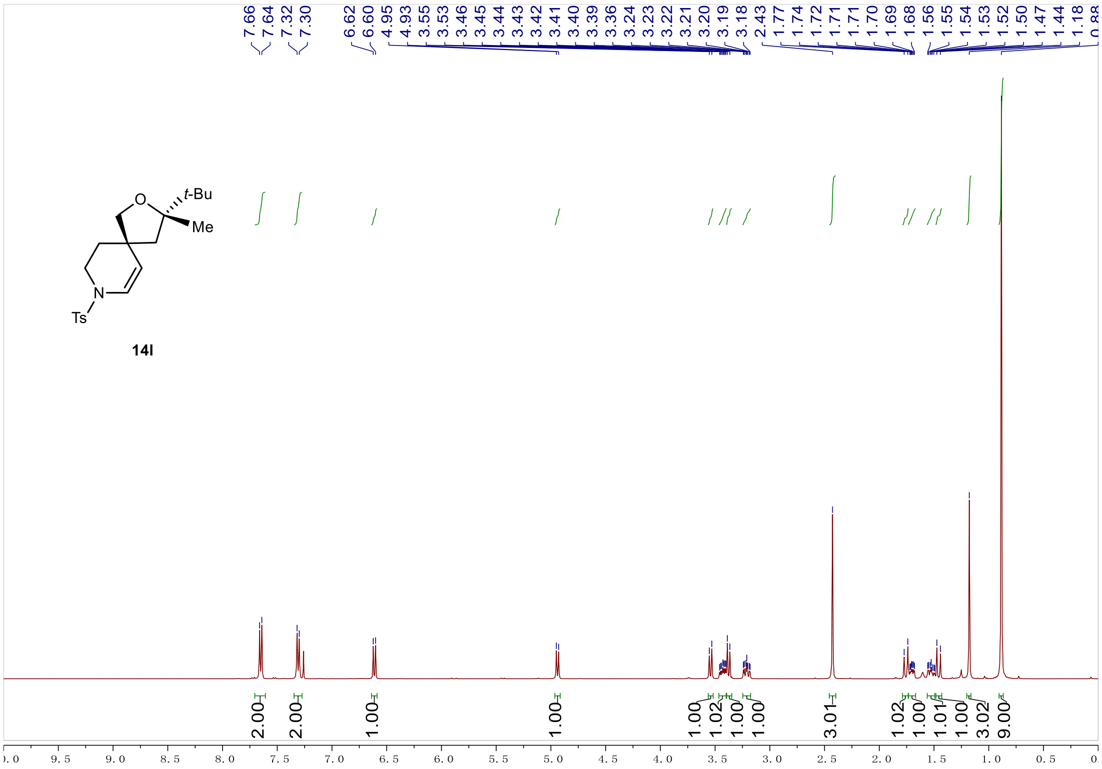

$^{13}C$ NMR (101 MHz, $CDCl_3$)

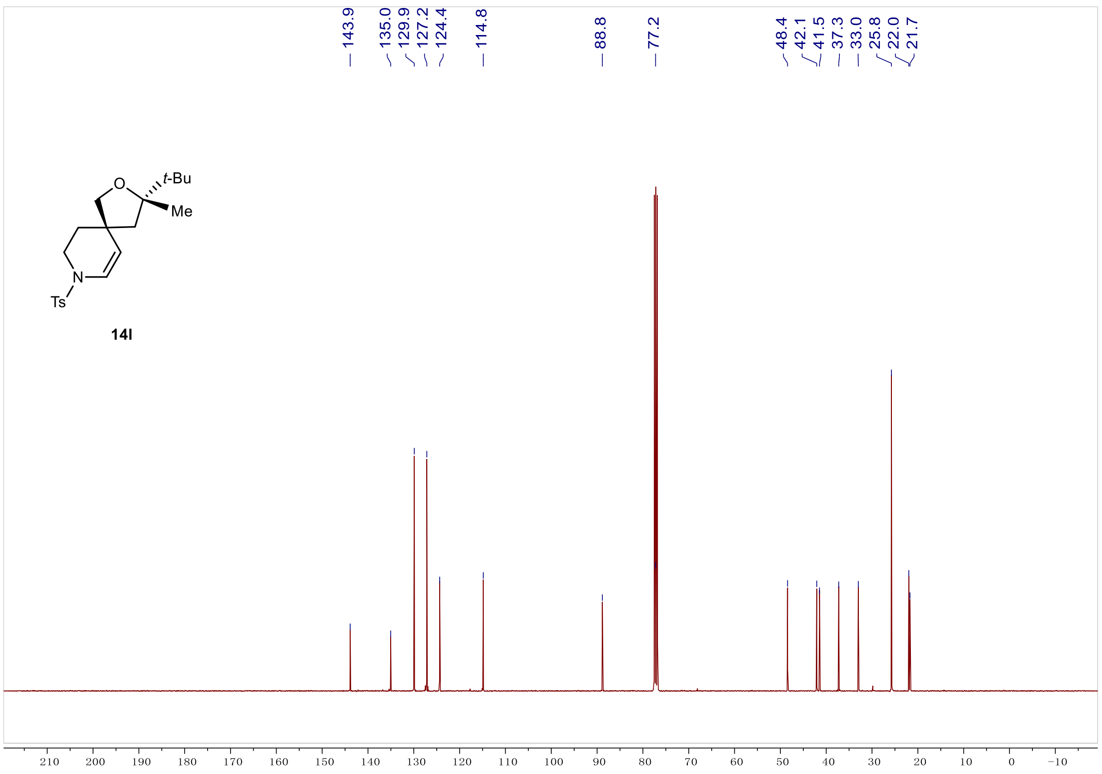

$^{1}H$ NMR (500 MHz, $CDCl_3$)

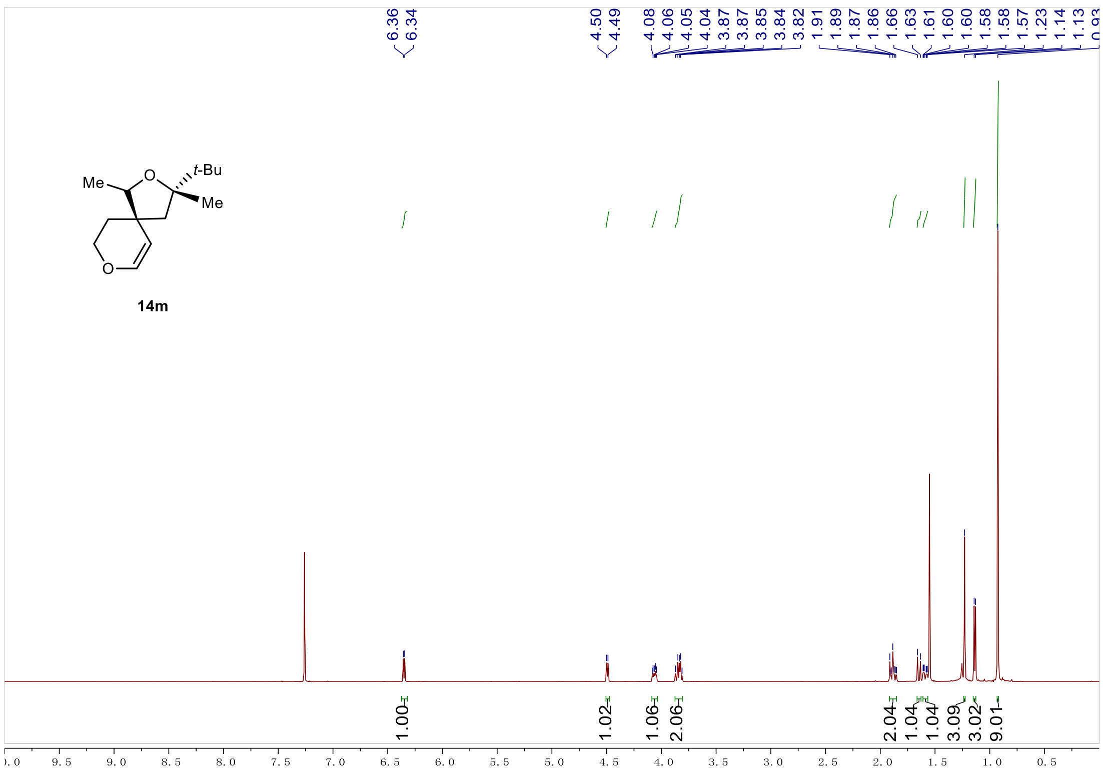


$^{13}C$ NMR (126 MHz, $CDCl_3$)

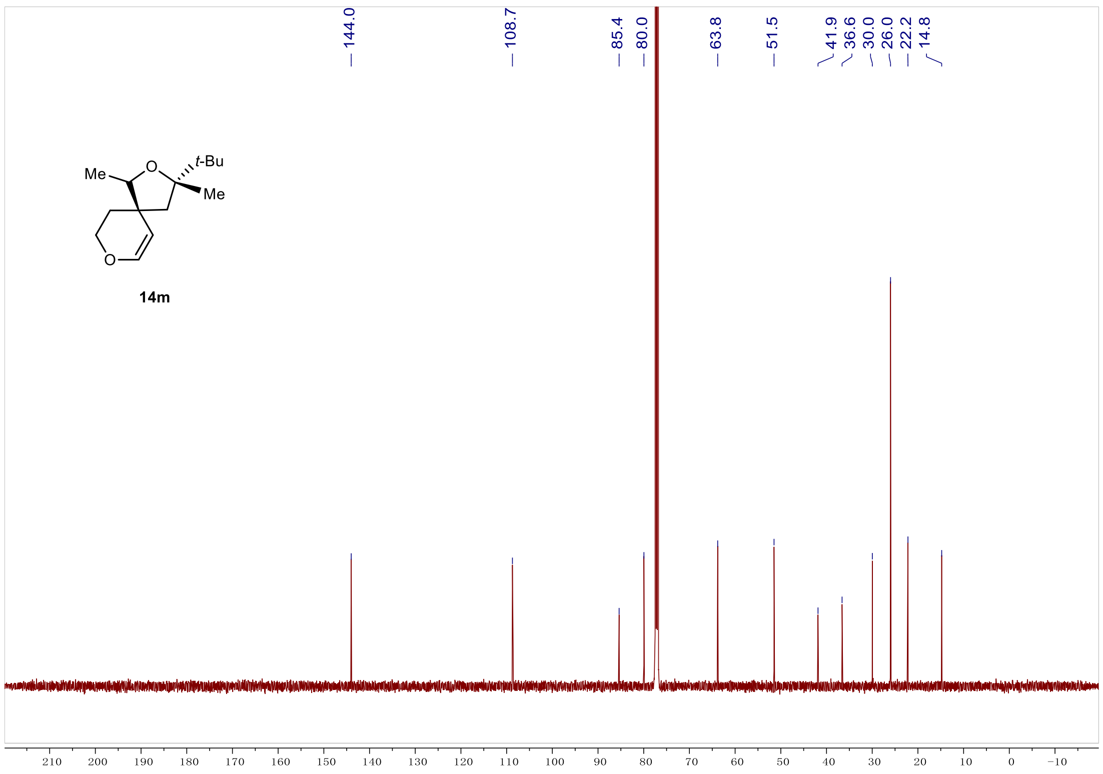

$^{1}H$ NMR (500 MHz, $CDCl_3$)

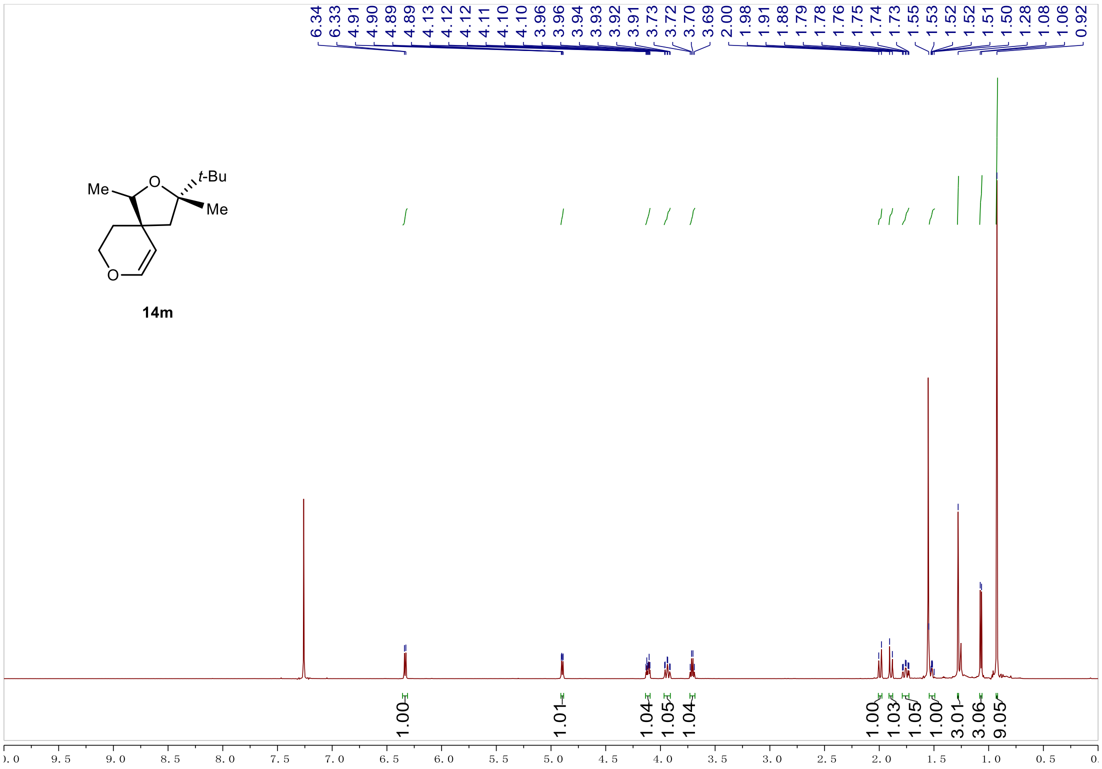


$^{13}C$ NMR (126 MHz, $CDCl_3$)

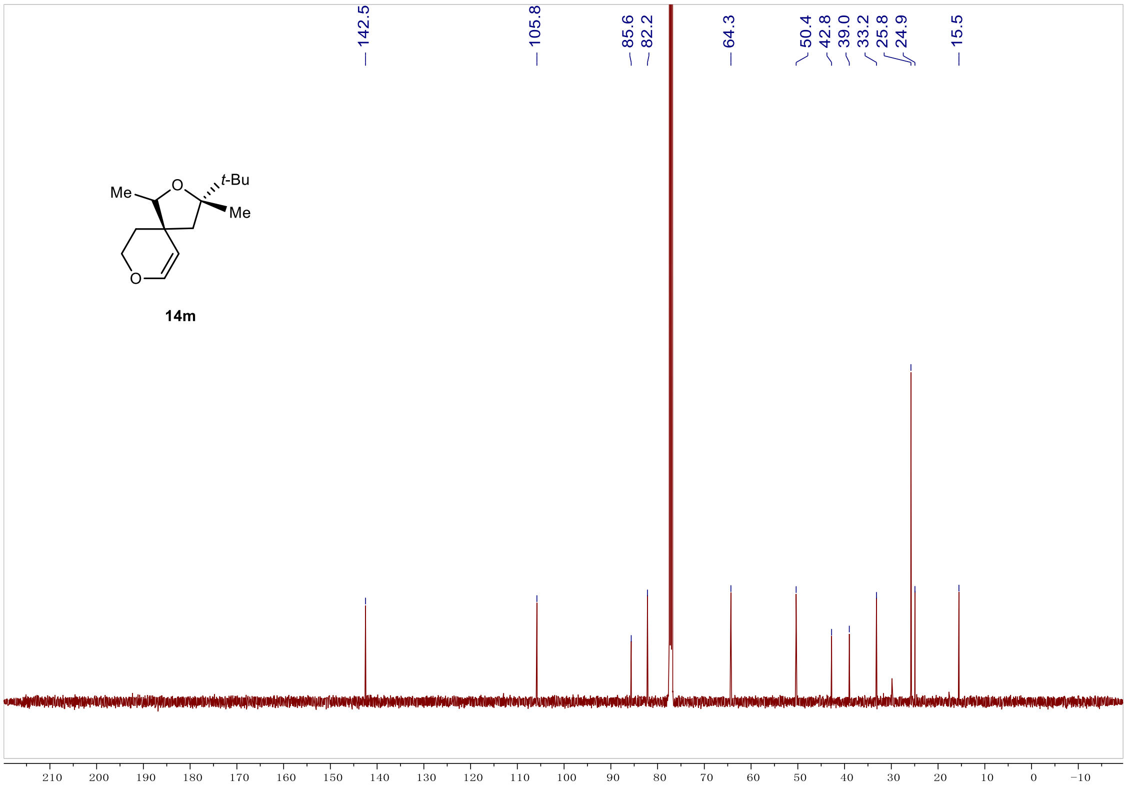

$^{1}H$ NMR (400 MHz, $CDCl_3$)

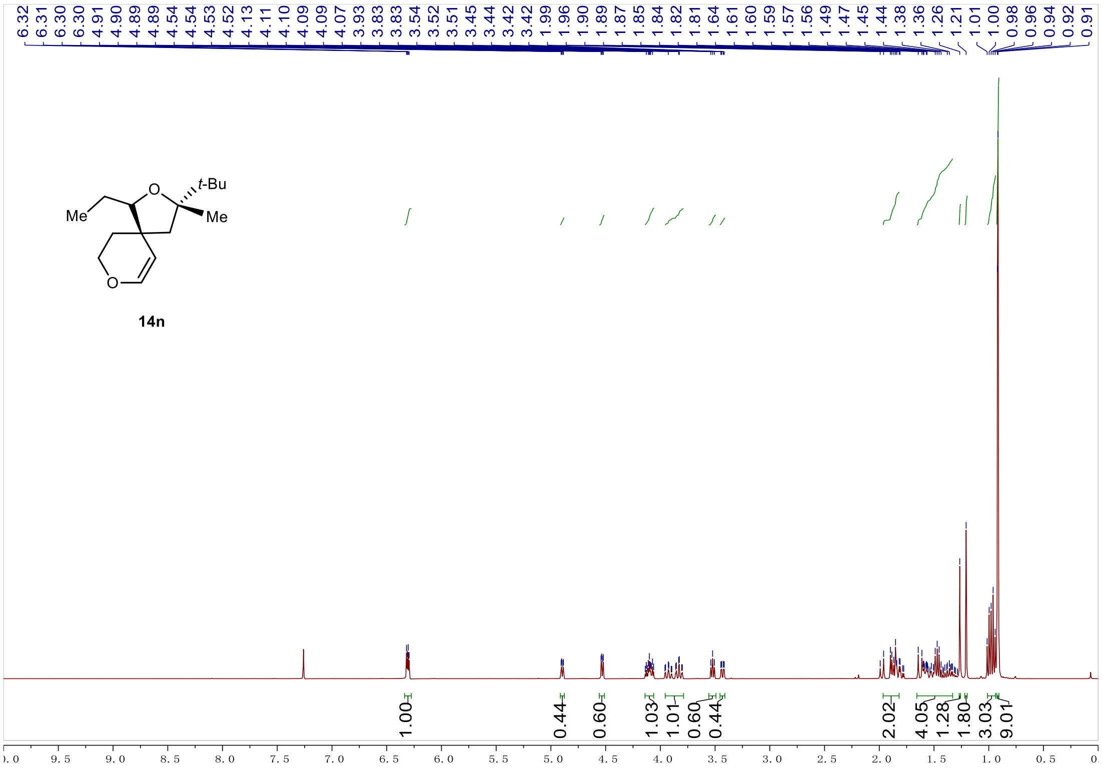


$^{13}C$ NMR (101 MHz, $CDCl_3$)

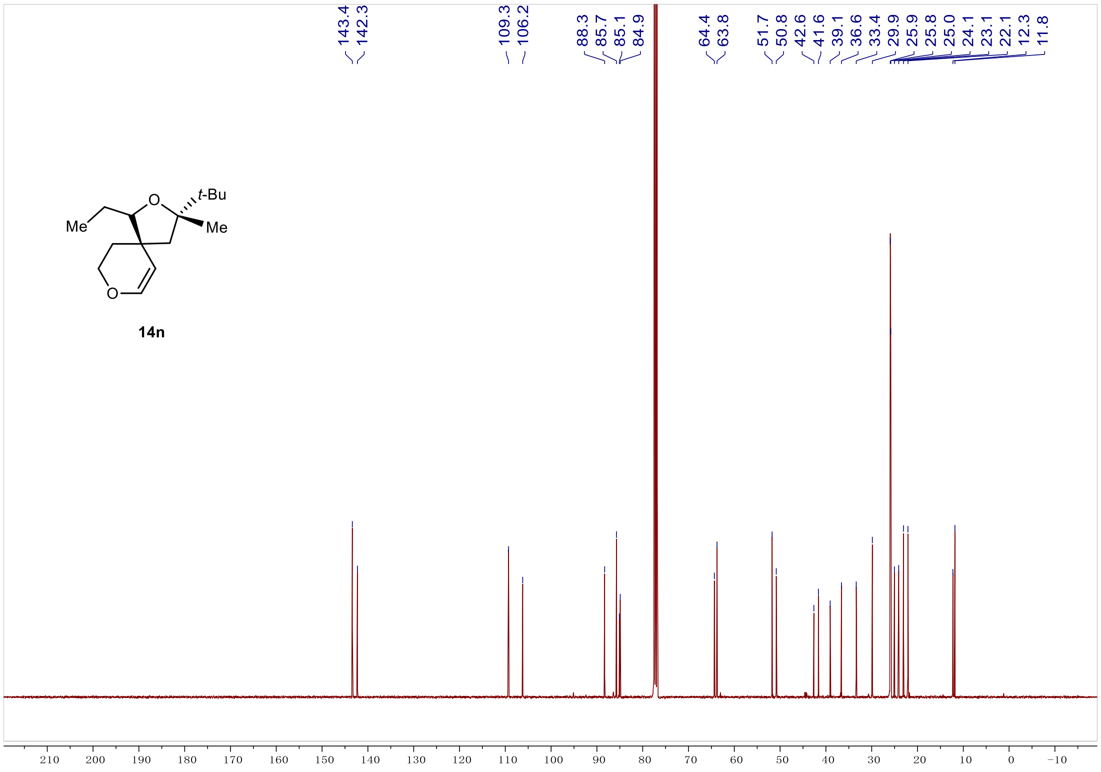

$^{1}$H NMR (500 MHz, $CDCl_3$)

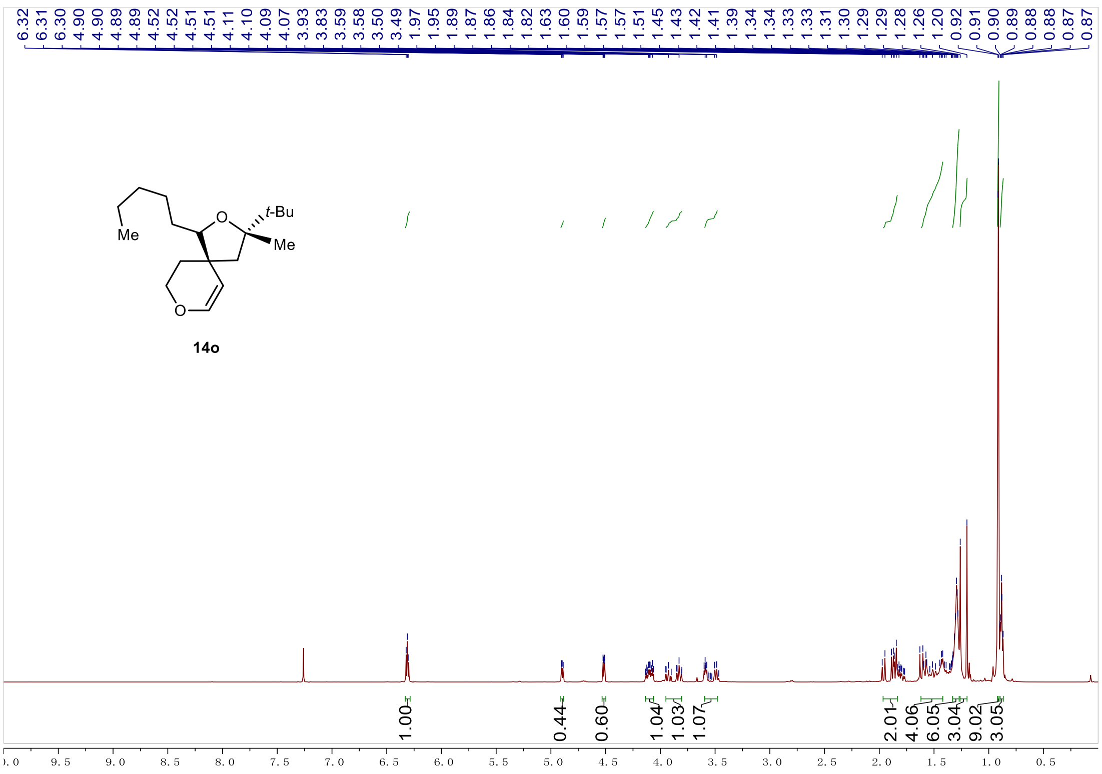


$^{13}$C NMR (126 MHz, $CDCl_3$)

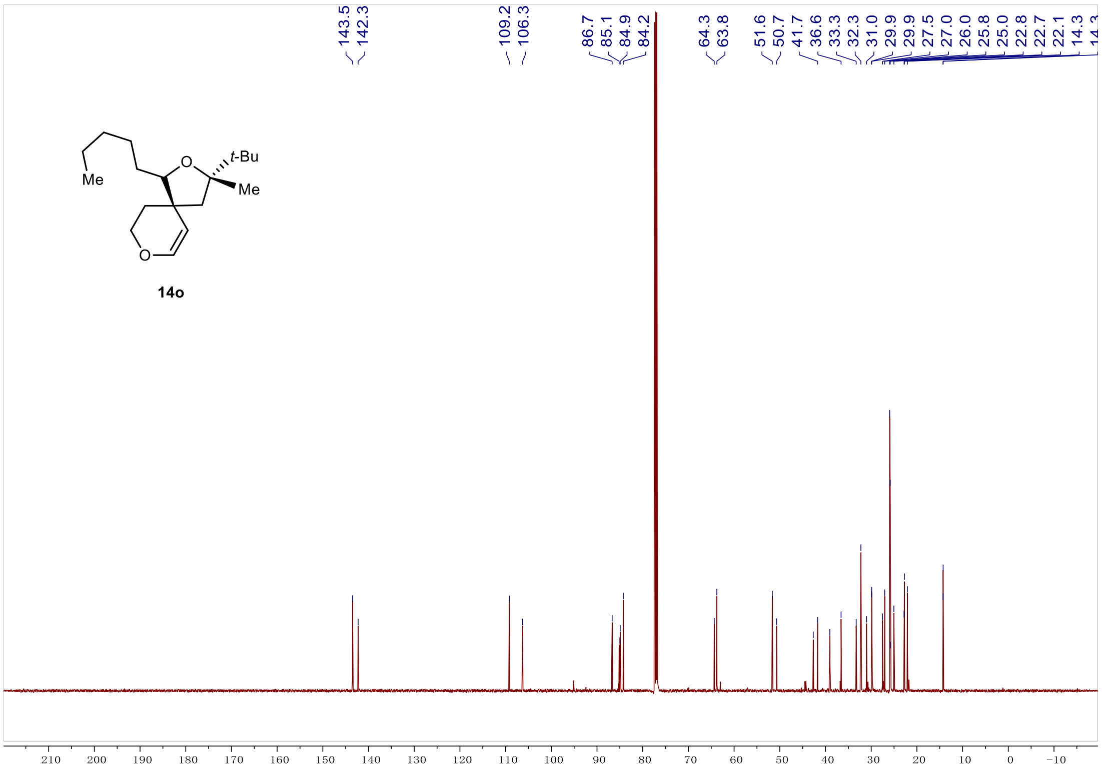

$^{1}H$ NMR (400 MHz, $CDCl_3$)

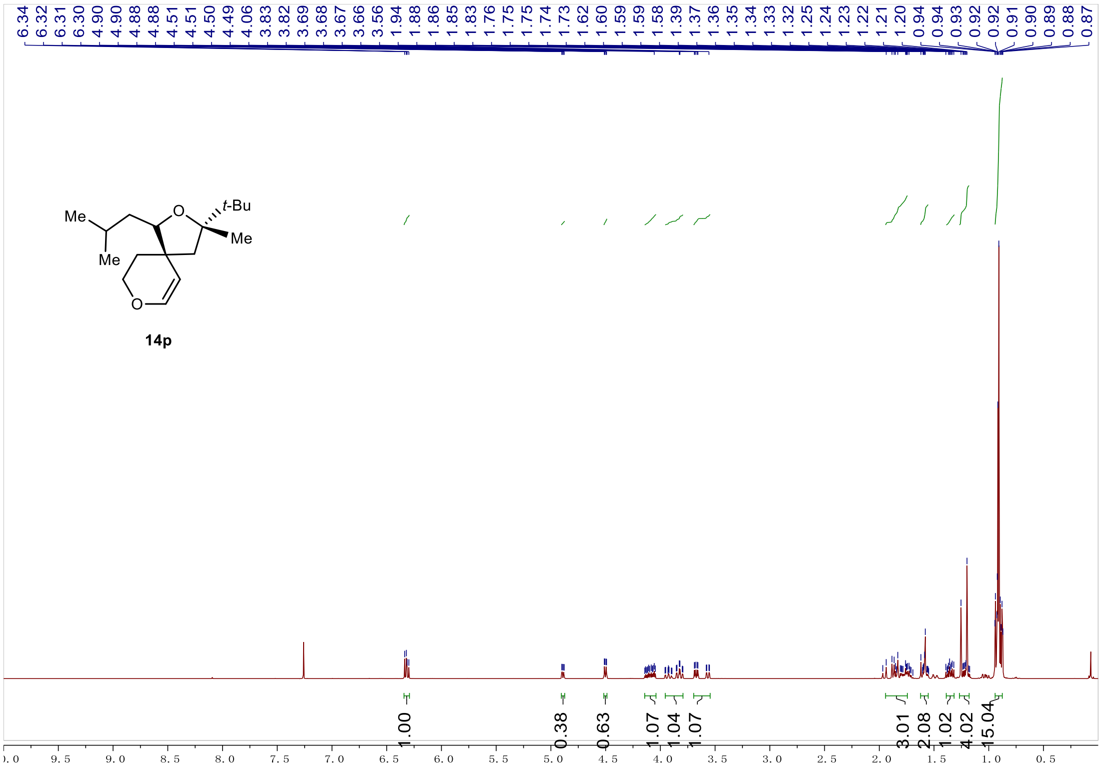


$^{13}C$ NMR (101 MHz, $CDCl_3$)

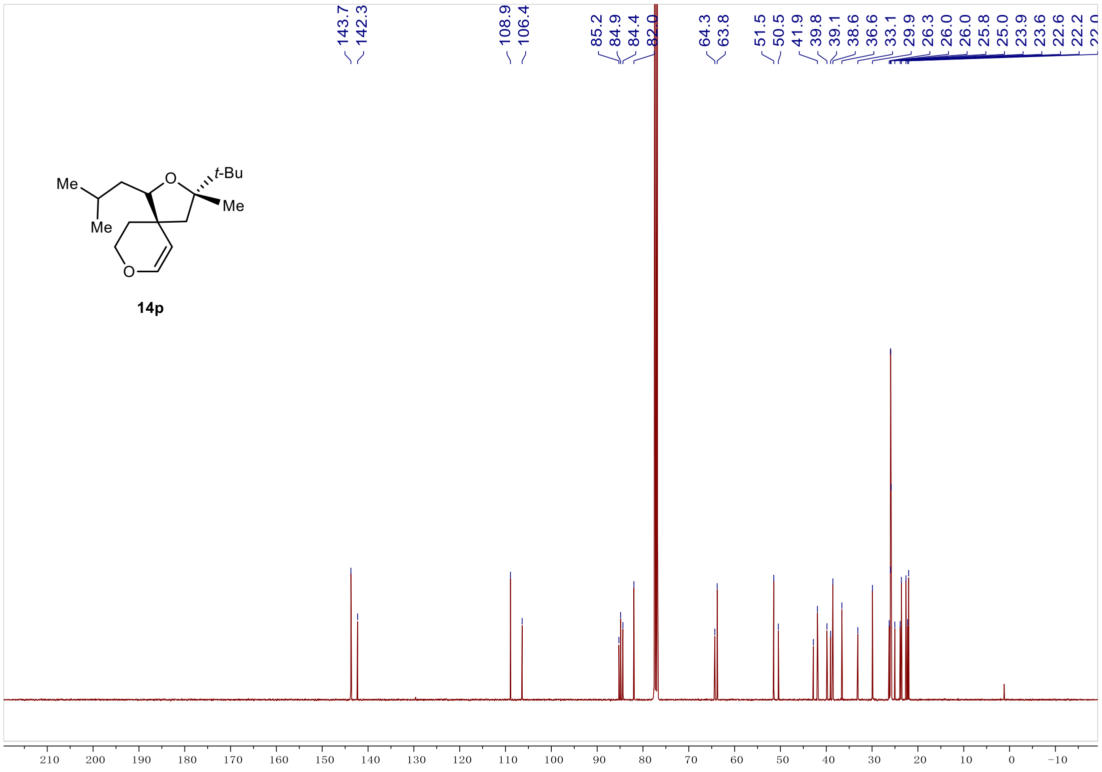

$^{1}$H NMR (400 MHz, $CDCl_3$)

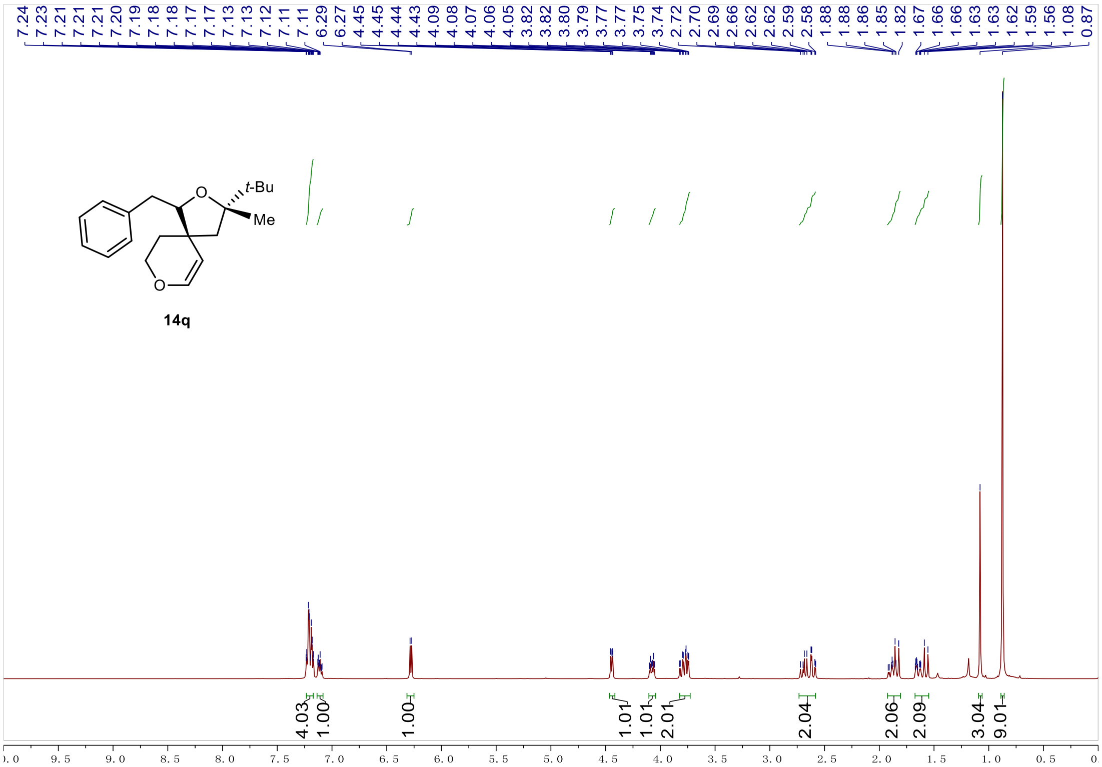


$^{13}$C NMR (101 MHz, $CDCl_3$)

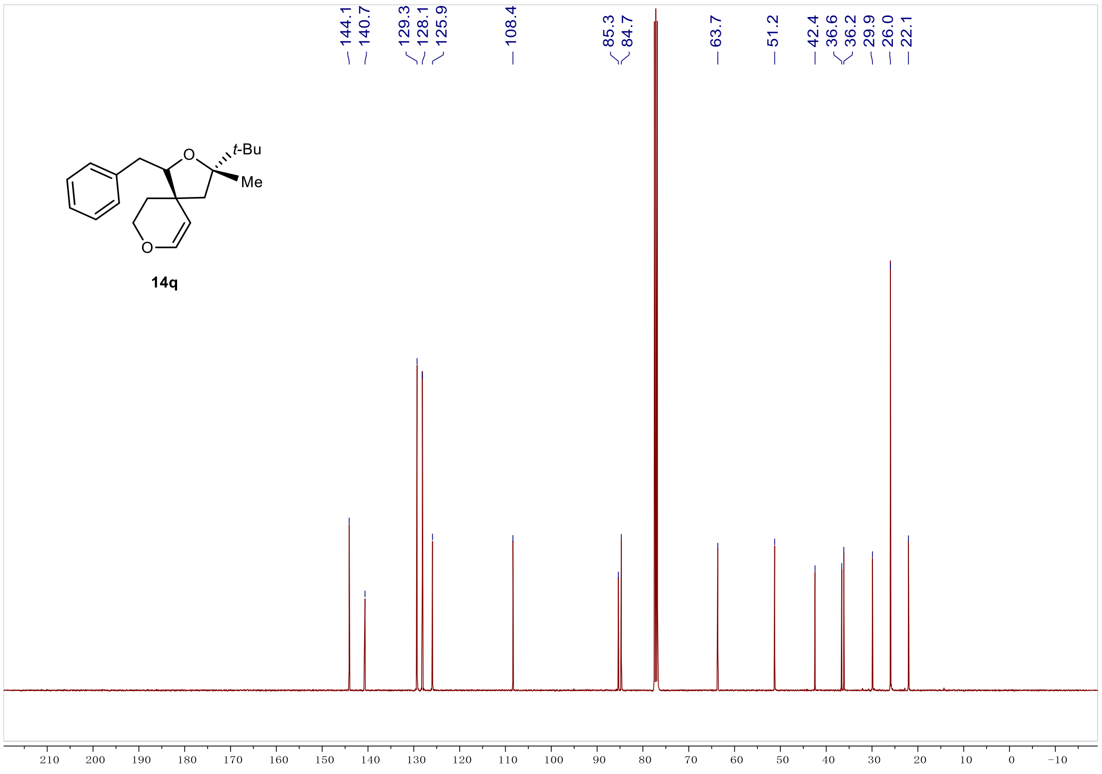

$^{1}H$ NMR (400 MHz, $CDCl_3$)

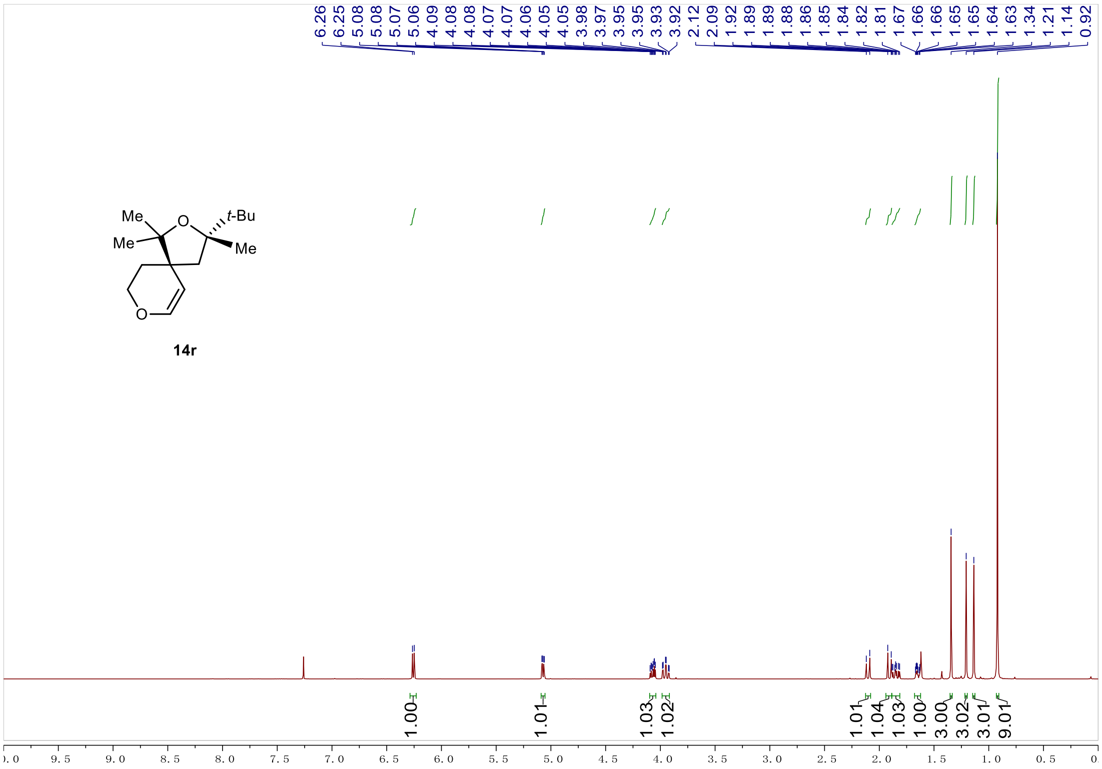


$^{13}C$ NMR (101 MHz, $CDCl_3$)

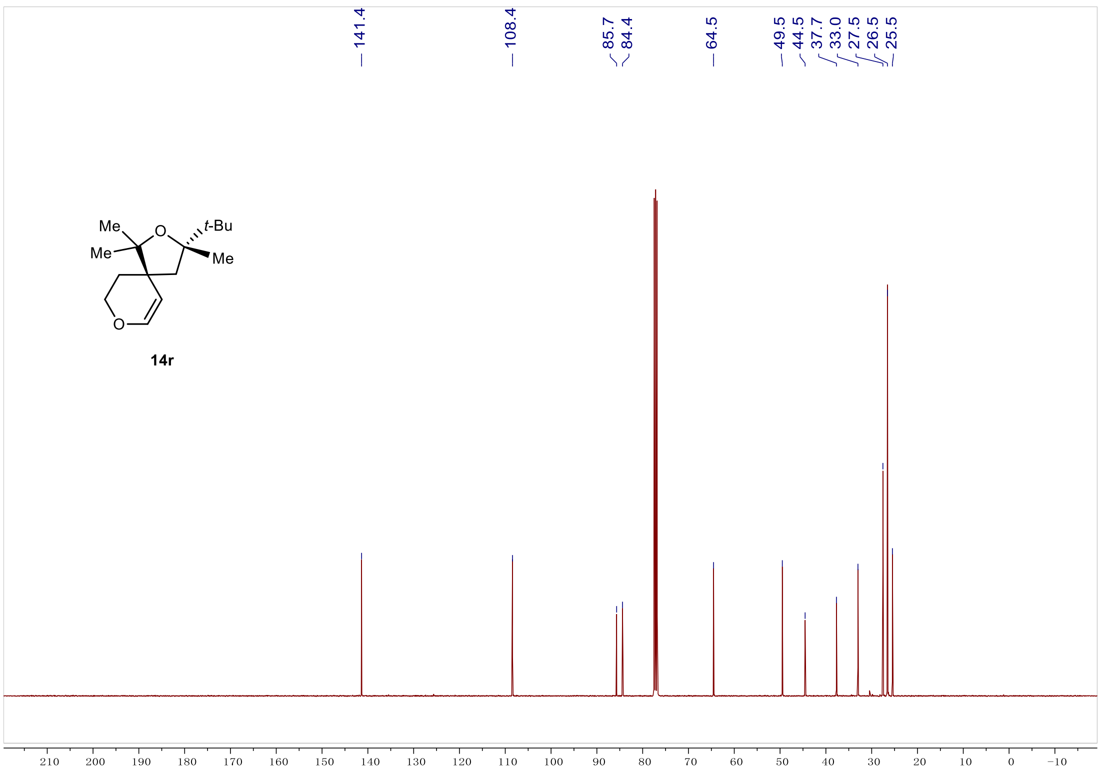

$^{1}H$ NMR (400 MHz, $C_6D_6$)

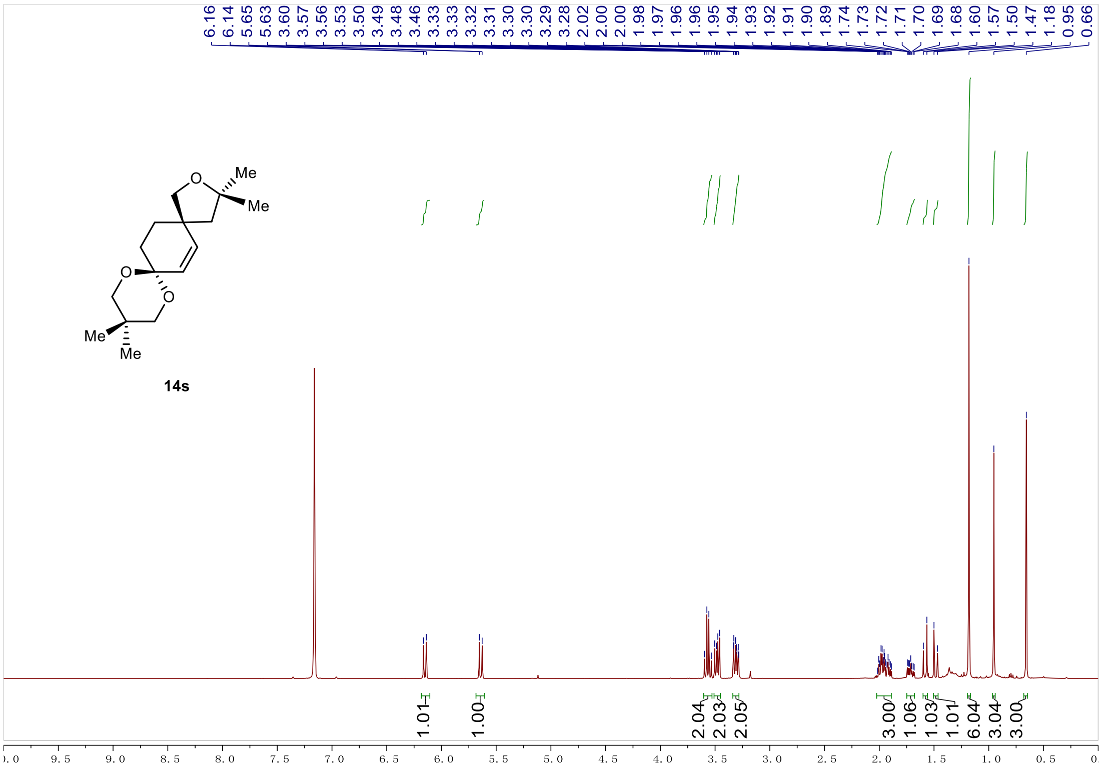


$^{13}C$ NMR (101 MHz, $C_6D_6$)

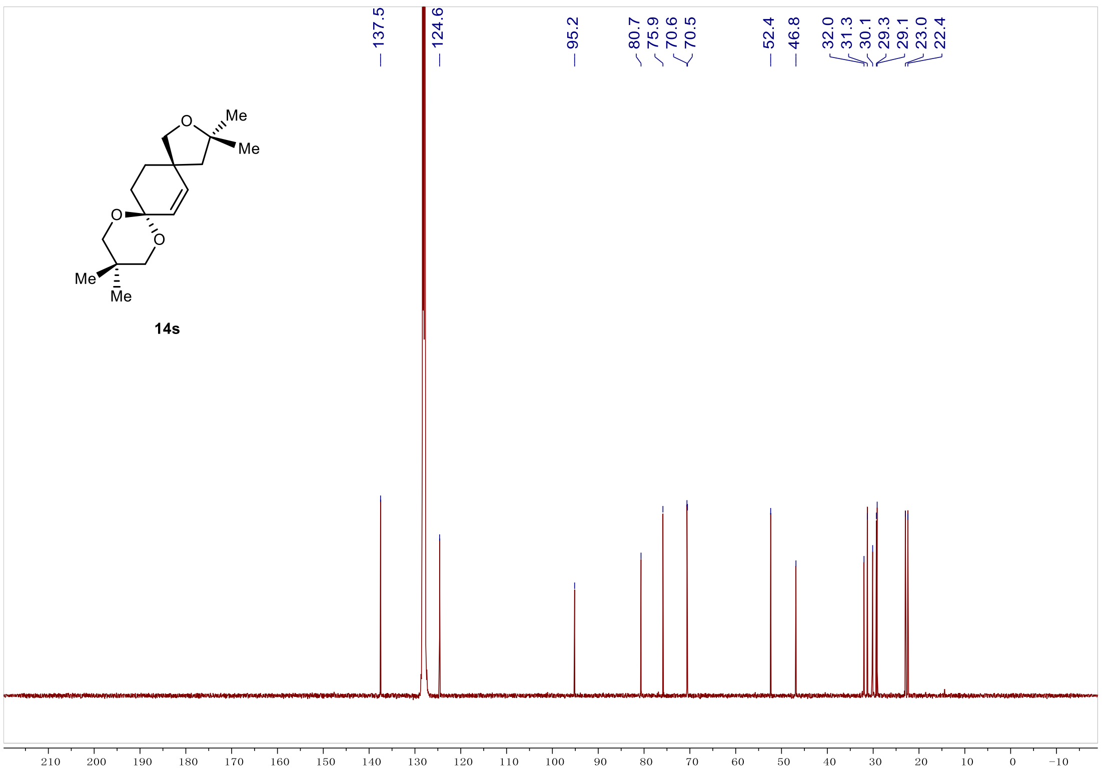

$^{1}$H NMR (400 MHz, $C_6D_6$)

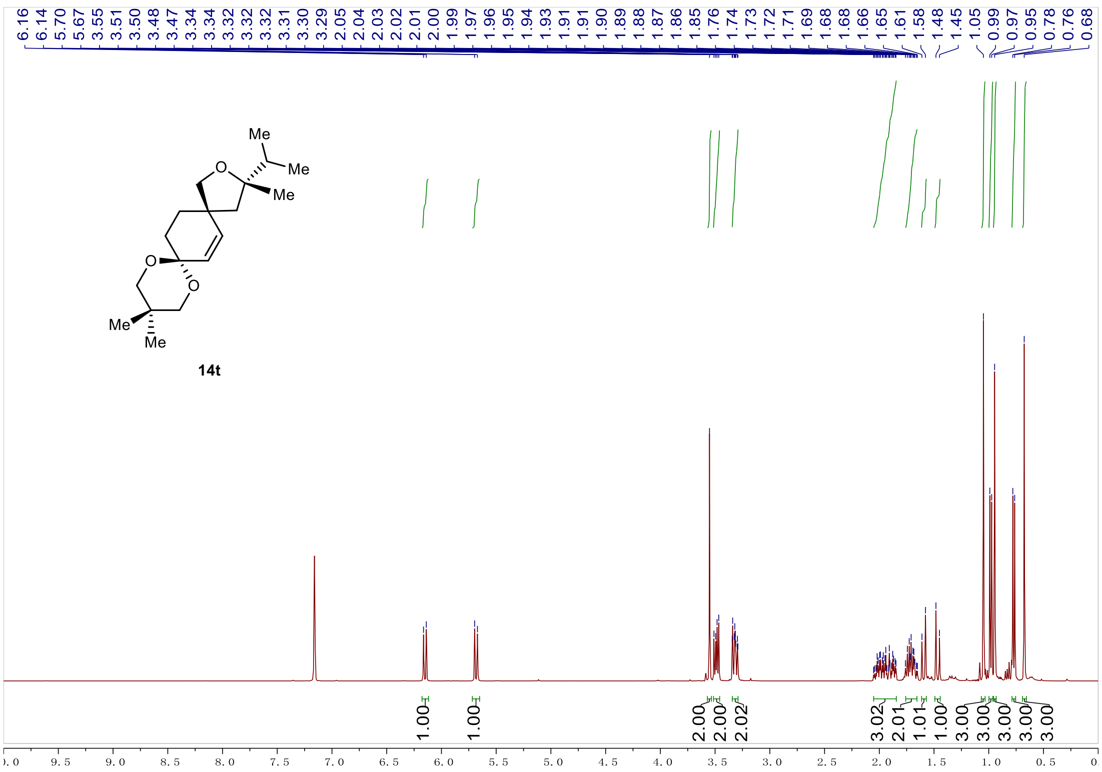


$^{13}$C NMR (101 MHz, $C_6D_6$)

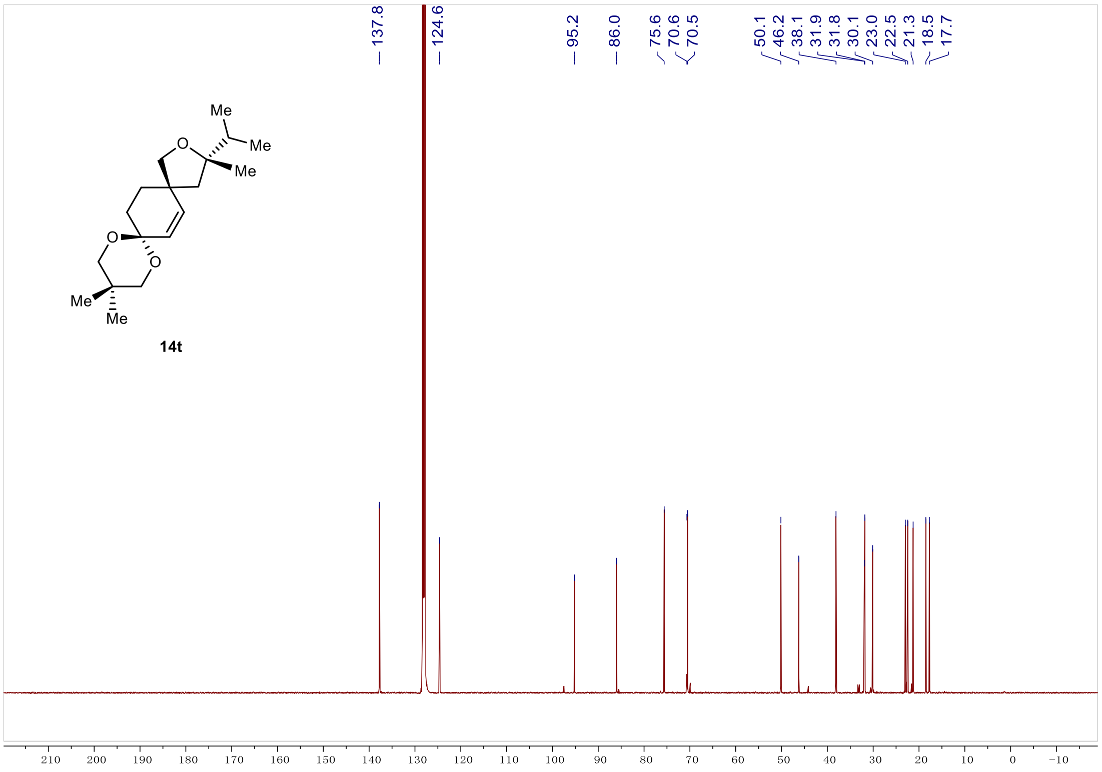

$^{1}H$ NMR (400 MHz, $CDCl_3$)

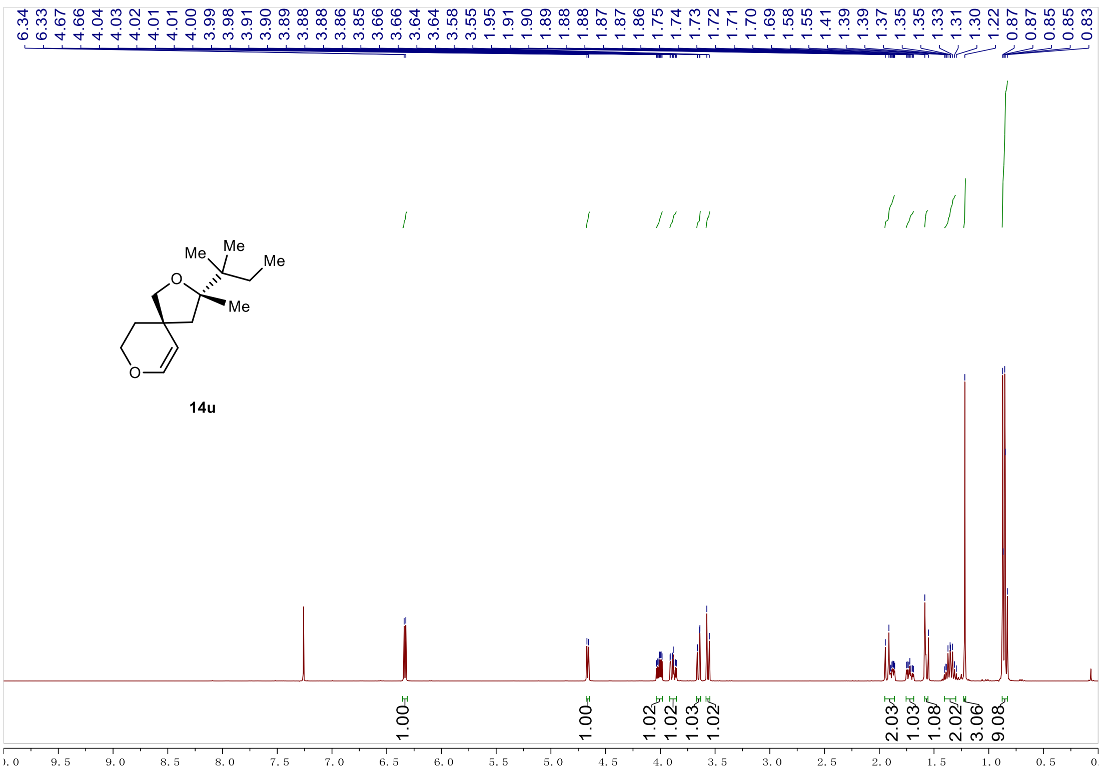

$^{13}C$ NMR (101 MHz, $CDCl_3$)

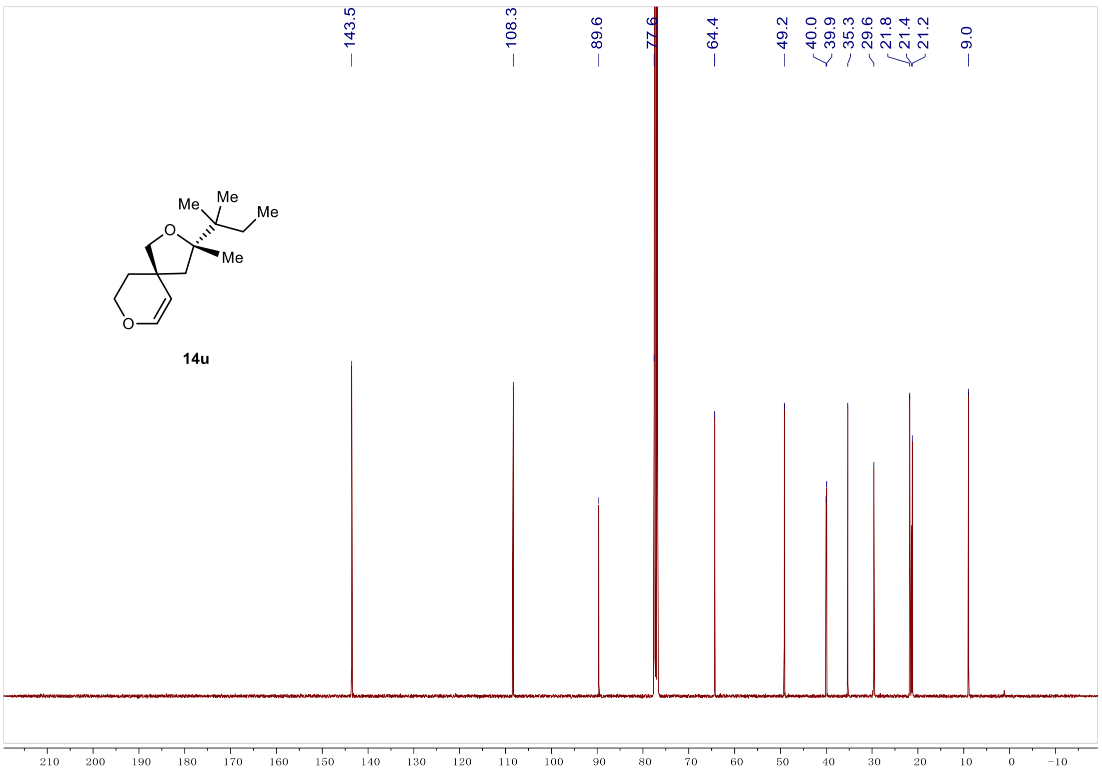

$^1$H NMR (400 MHz, $CDCl_3$)

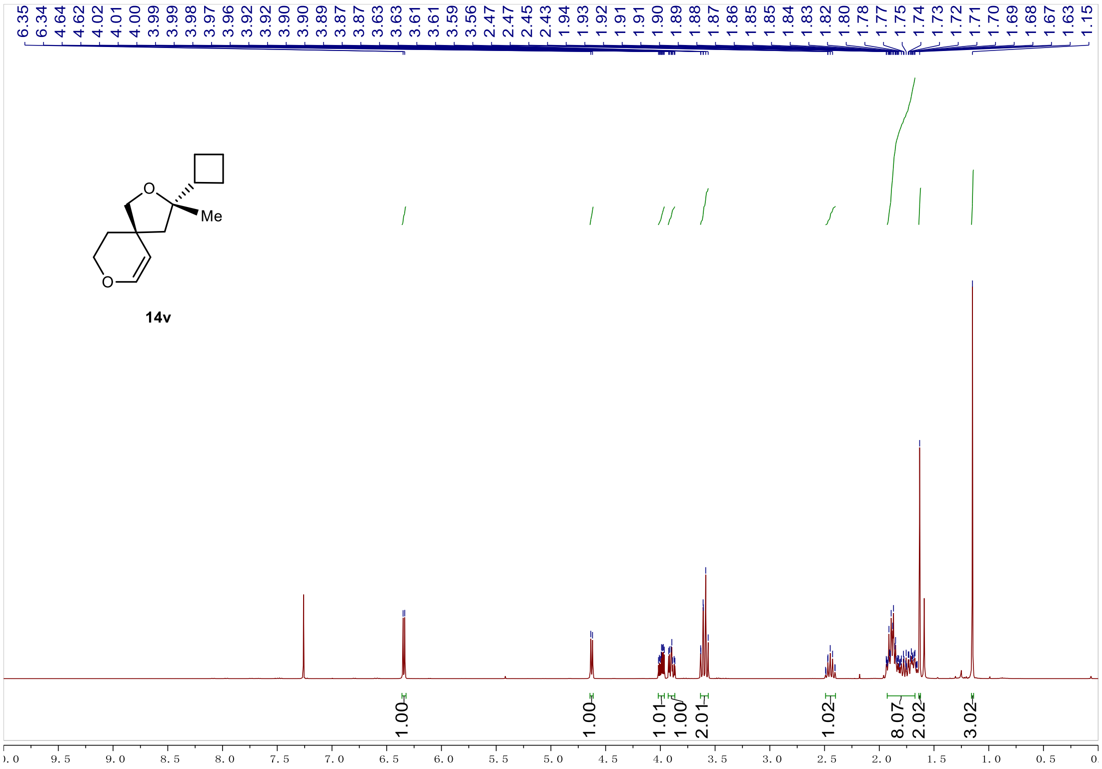

$^{13}$C NMR (101 MHz, $CDCl_3$)

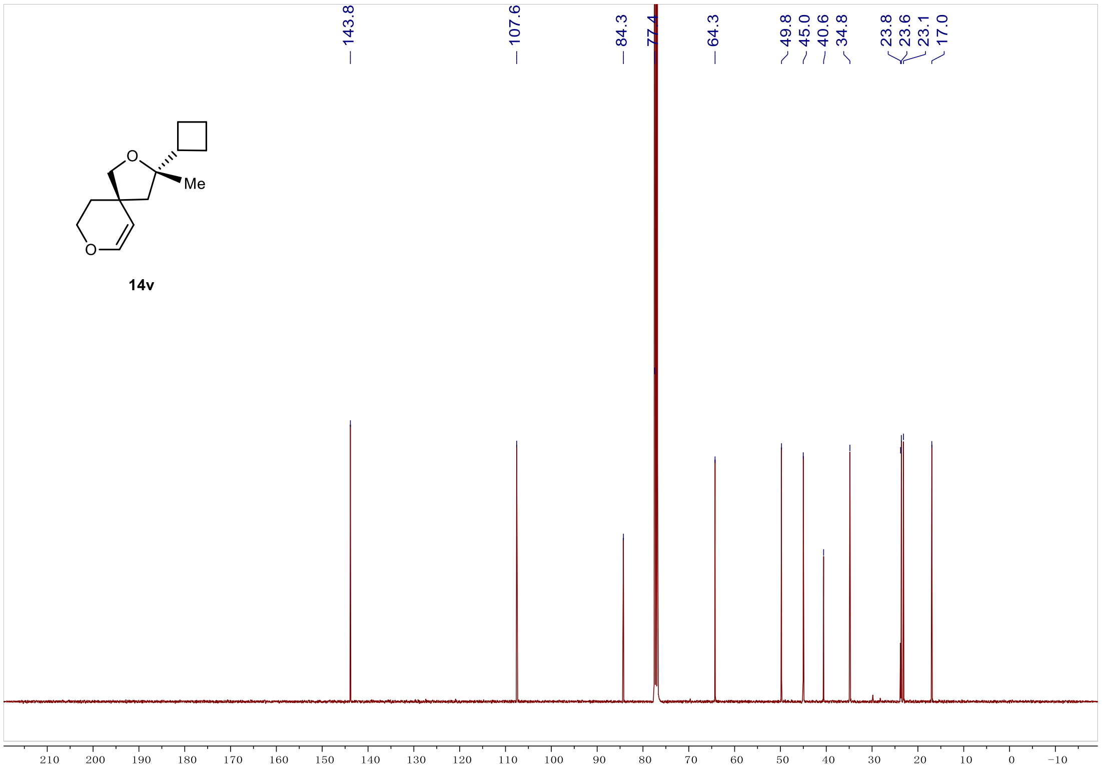

$^{1}$H NMR (400 MHz, $CDCl_3$)

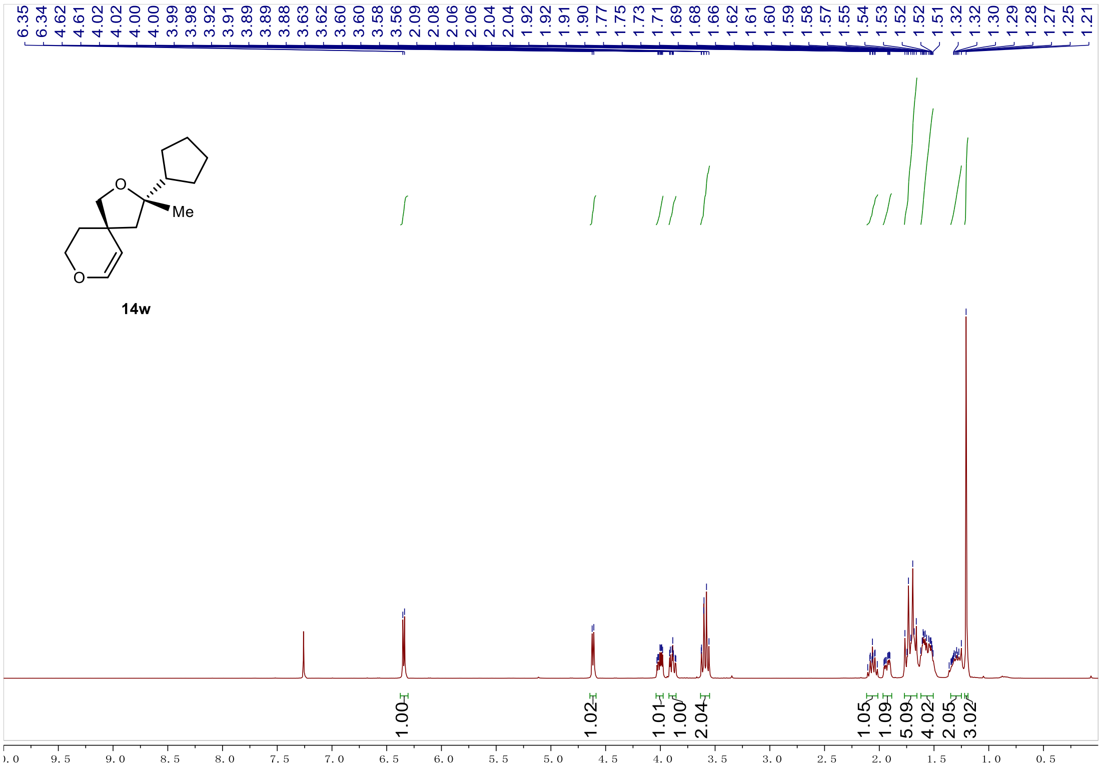


$^{13}$C NMR (101 MHz, $CDCl_3$)

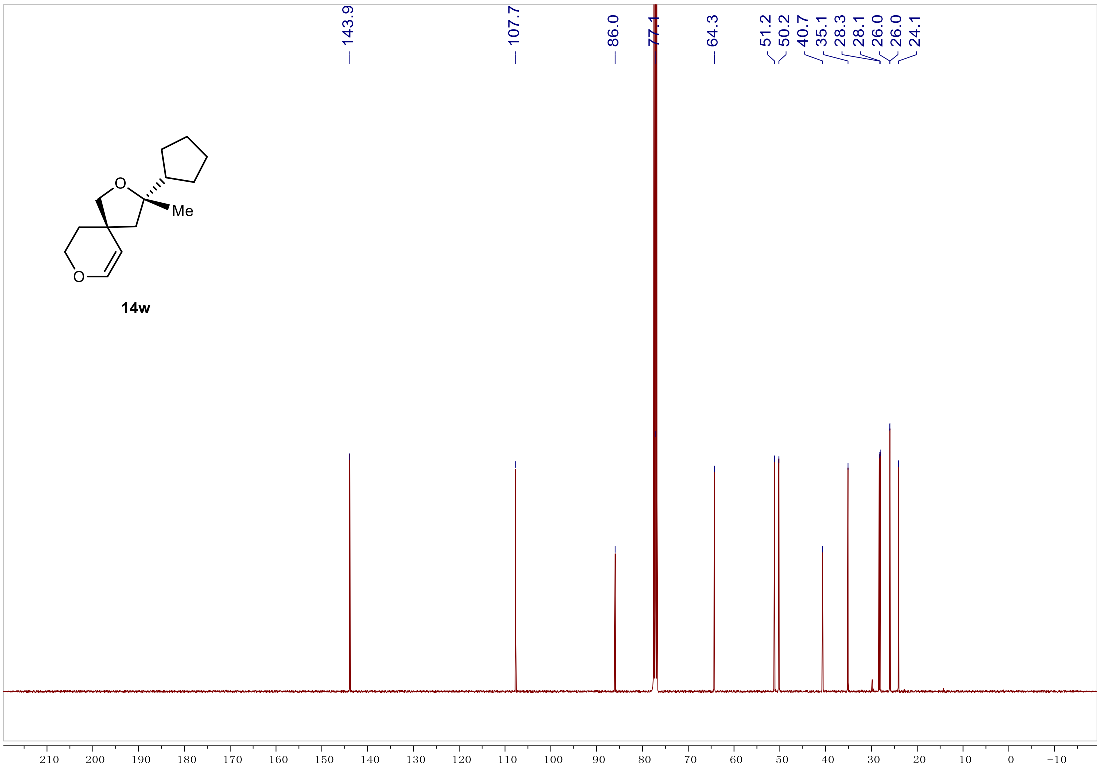

$^{1}H$ NMR (400 MHz, $CDCl_3$)

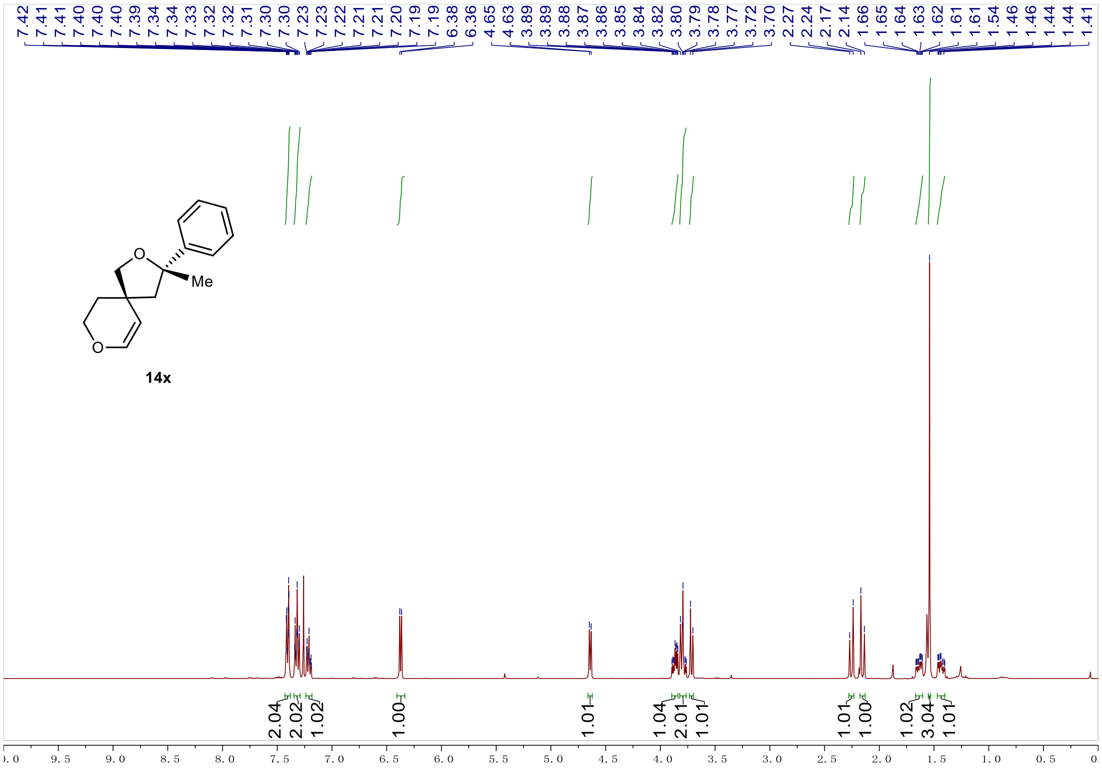

$^{13}C$ NMR (101 MHz, $CDCl_3$)

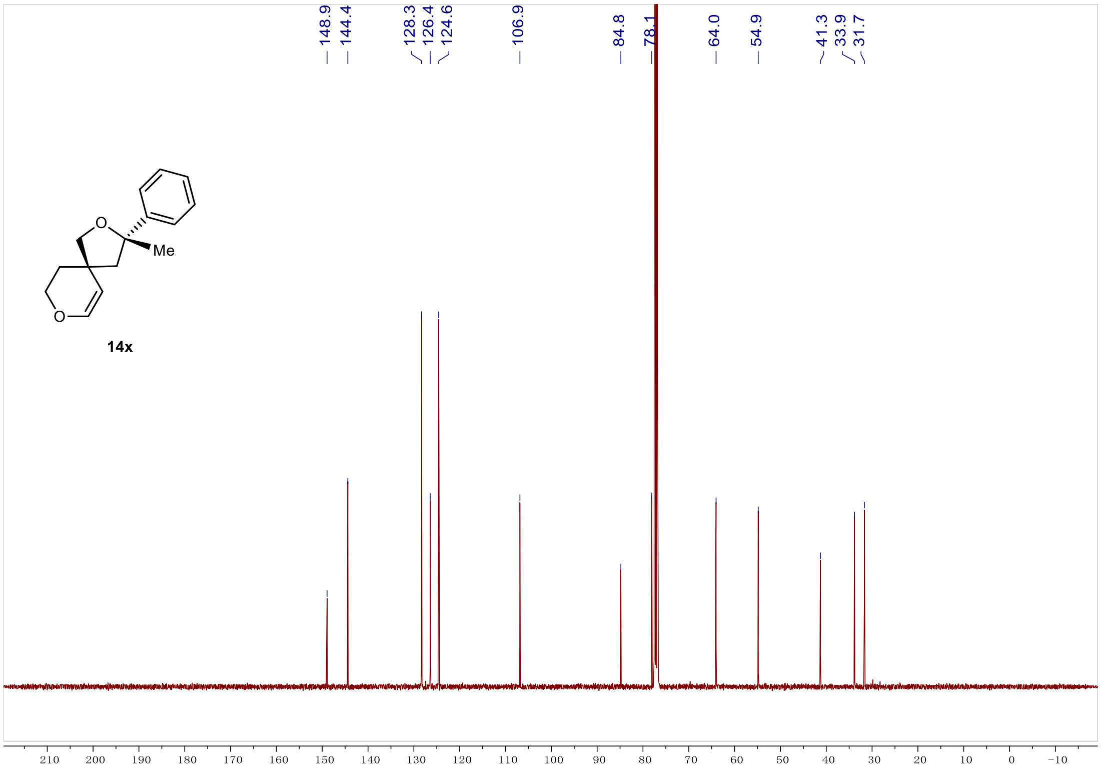

$^{1}$H NMR (400 MHz, $CDCl_3$)

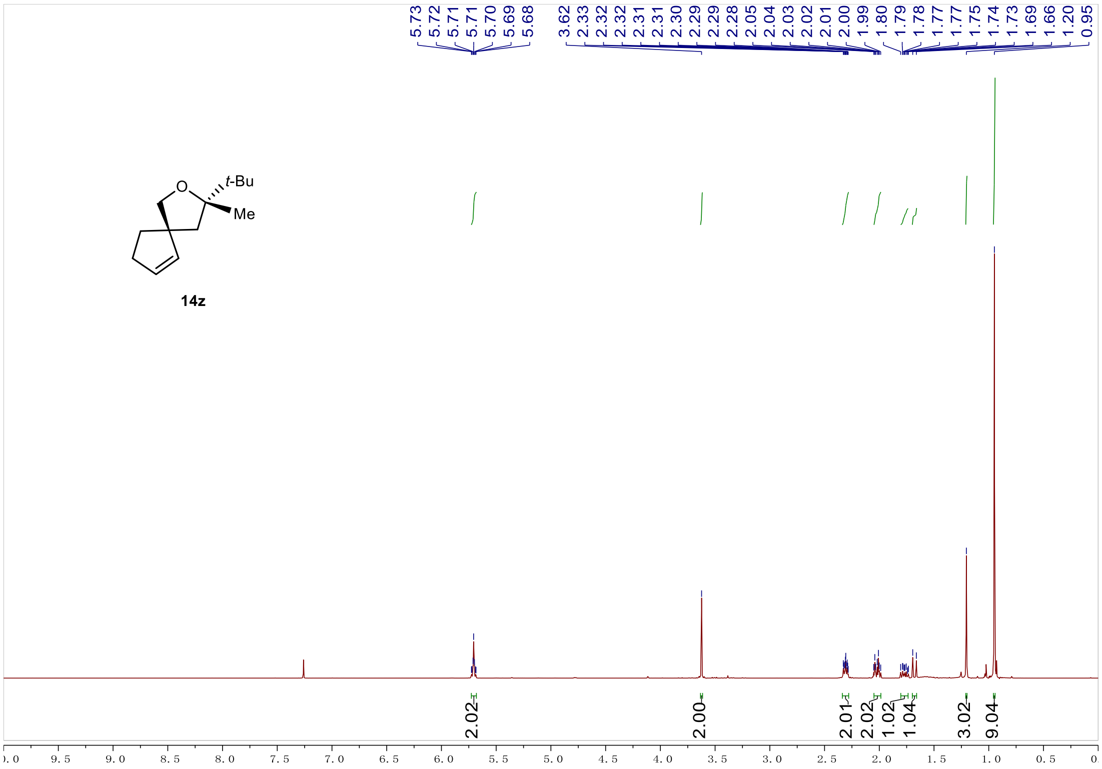


$^{13}$C NMR (101 MHz, $CDCl_3$)

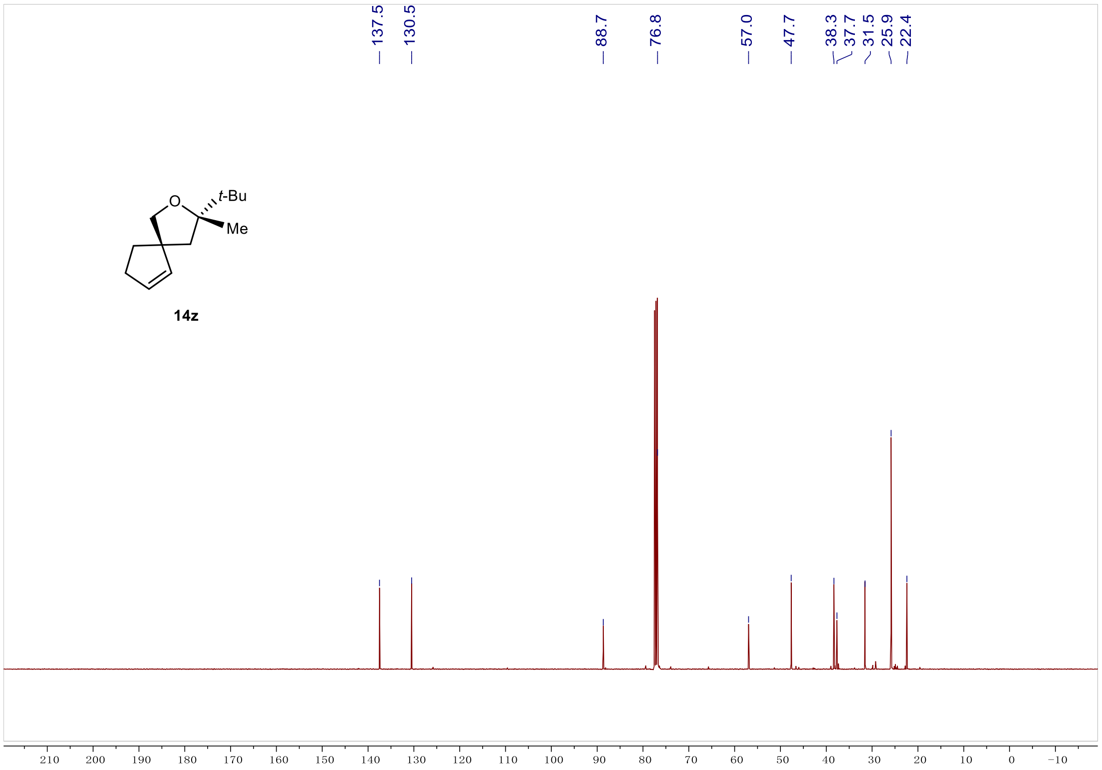

$^{1}H$ NMR (400 MHz, $CDCl_3$)

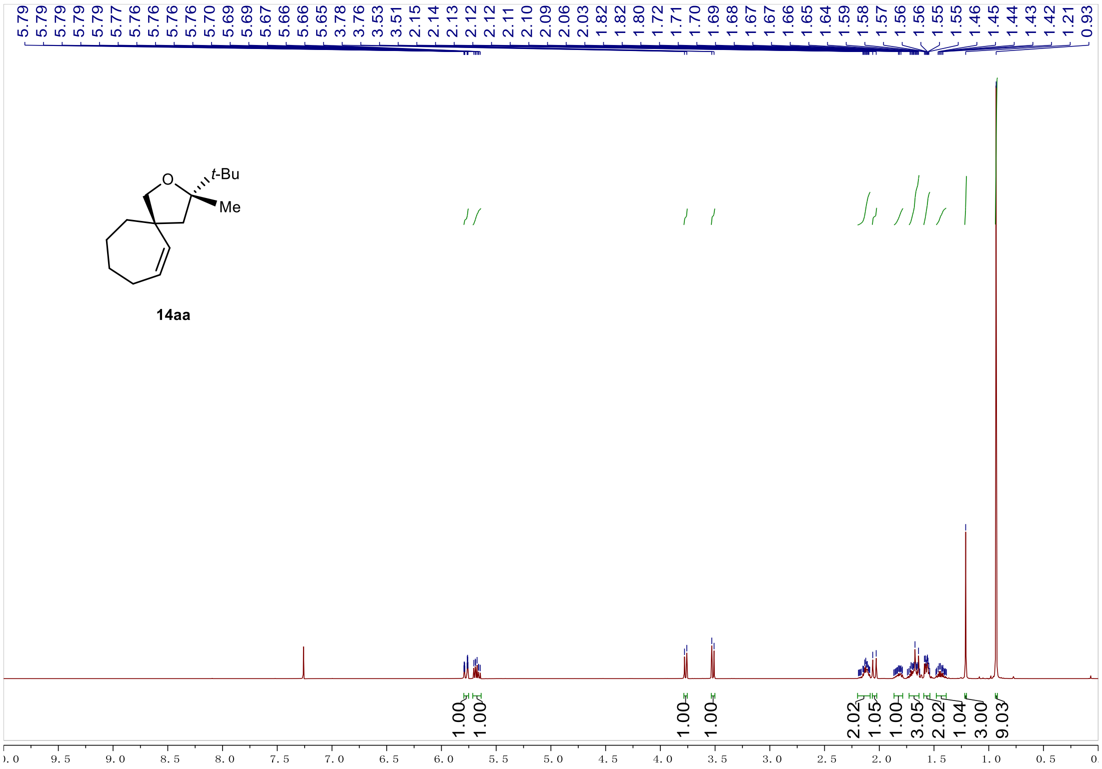


$^{13}C$ NMR (101 MHz, $CDCl_3$)

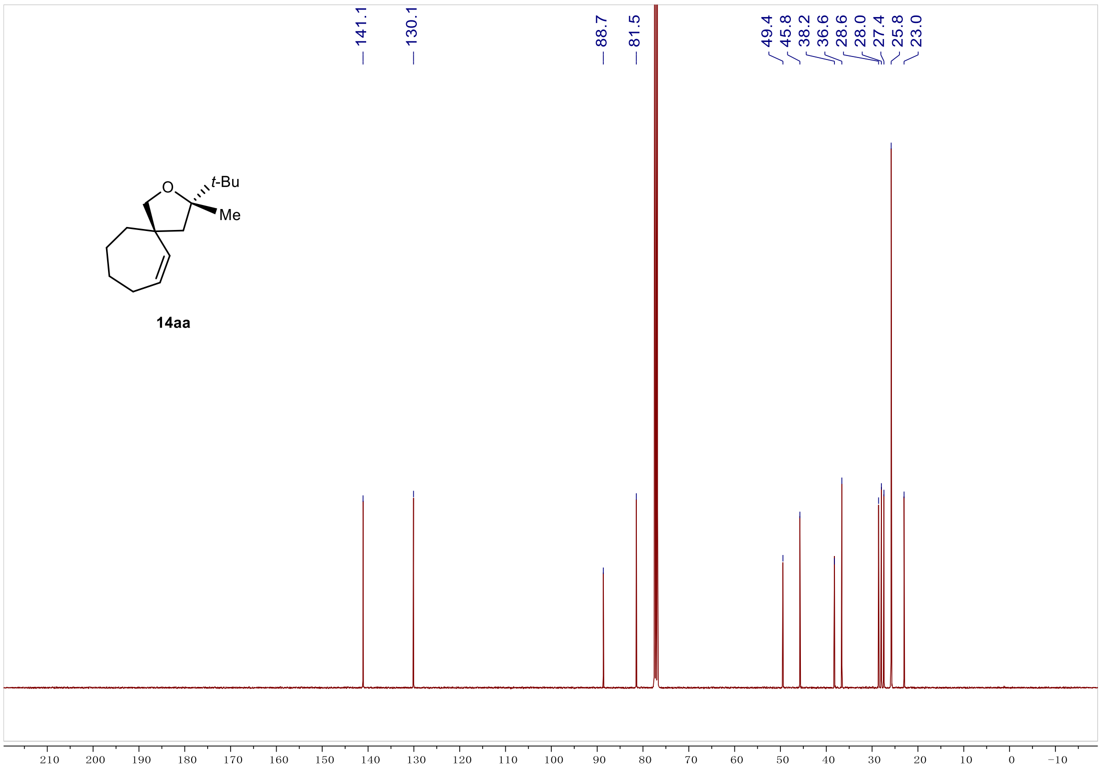

$^{1}H$ NMR (400 MHz, $CDCl_3$)

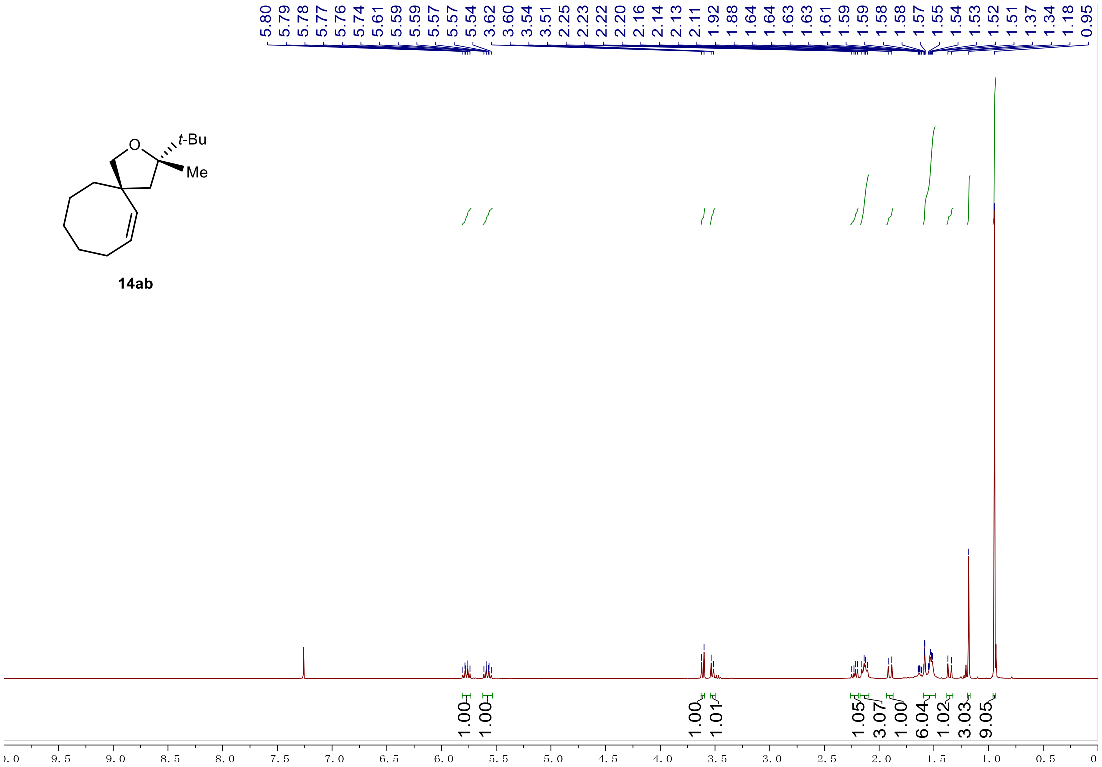


$^{13}C$ NMR (101 MHz, $CDCl_3$)

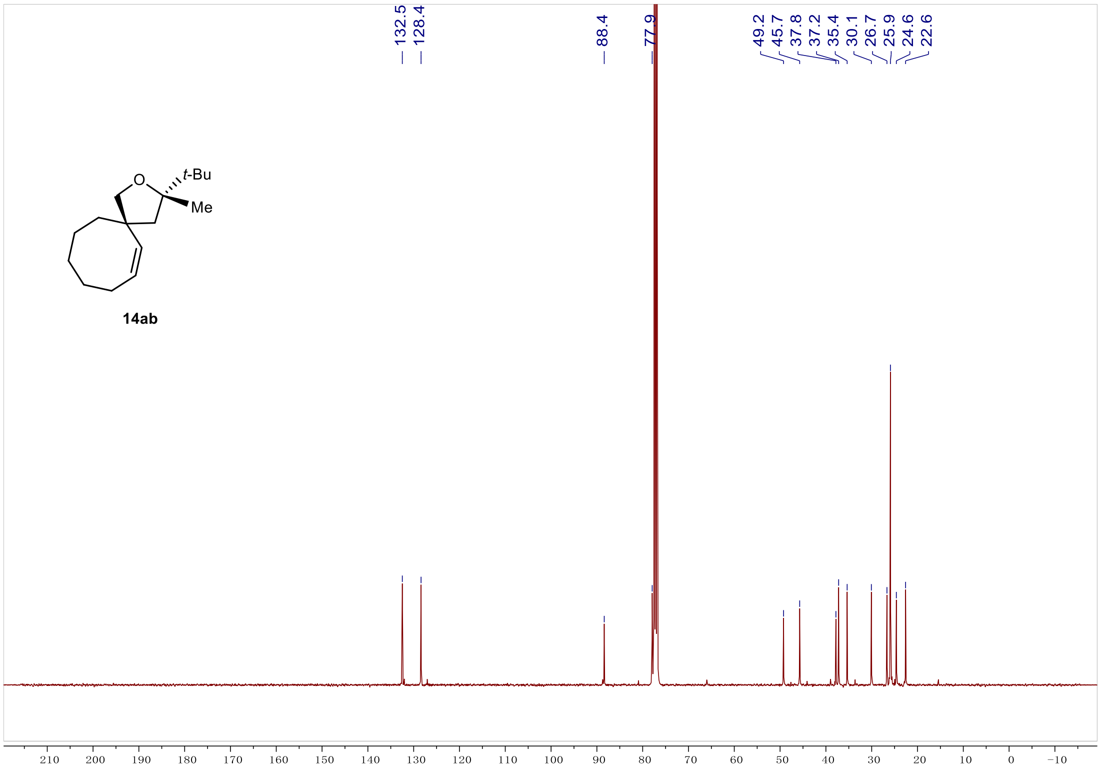

$^{1}H$ NMR (500 MHz, $C_6D_6$)

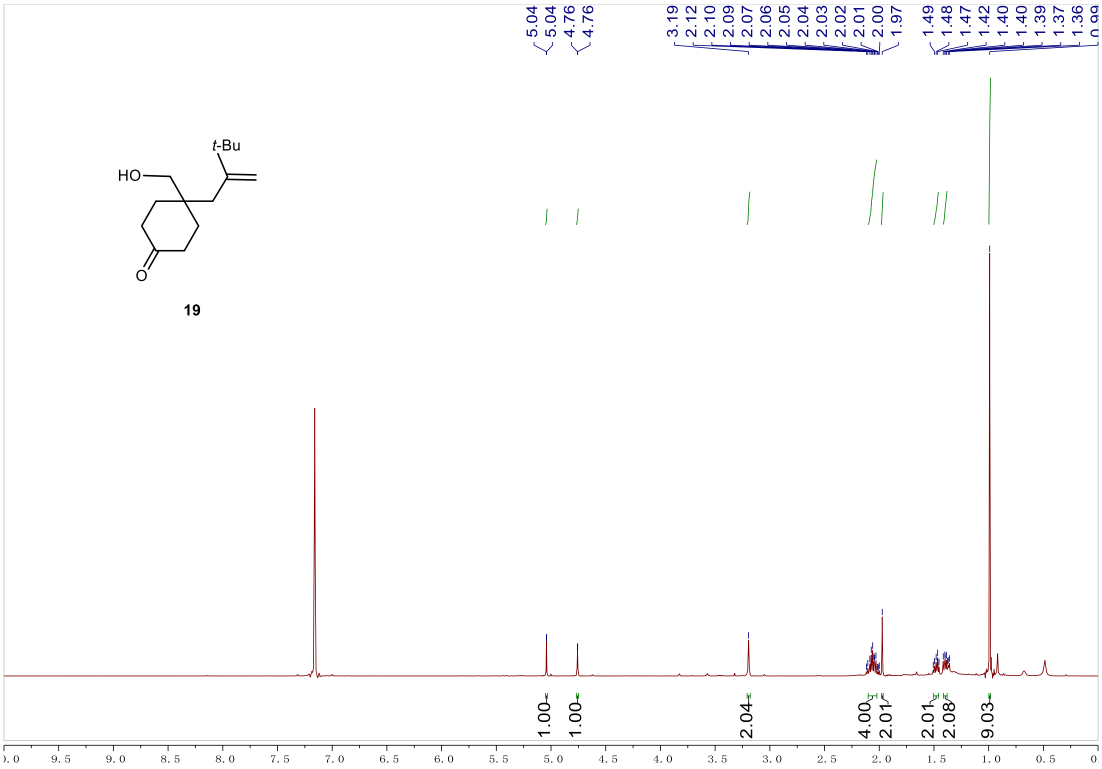


$^{13}C$ NMR (126 MHz, $C_6D_6$)

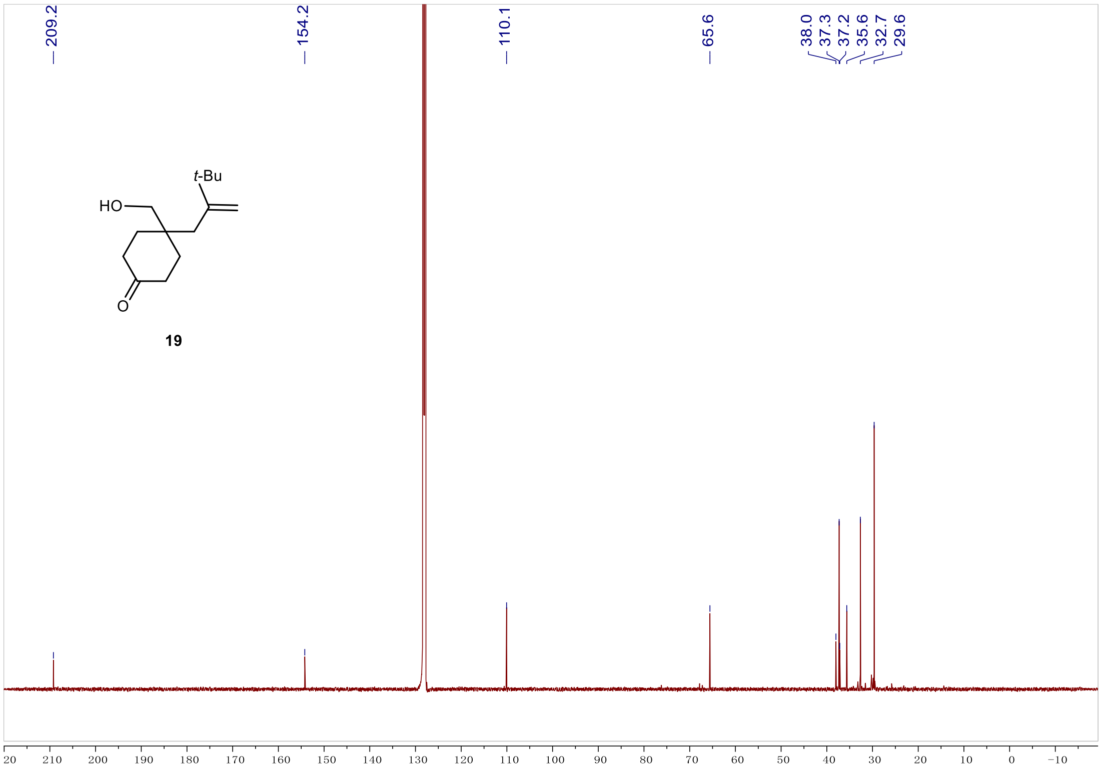

$^{1}$H NMR (500 MHz, $CDCl_3$)

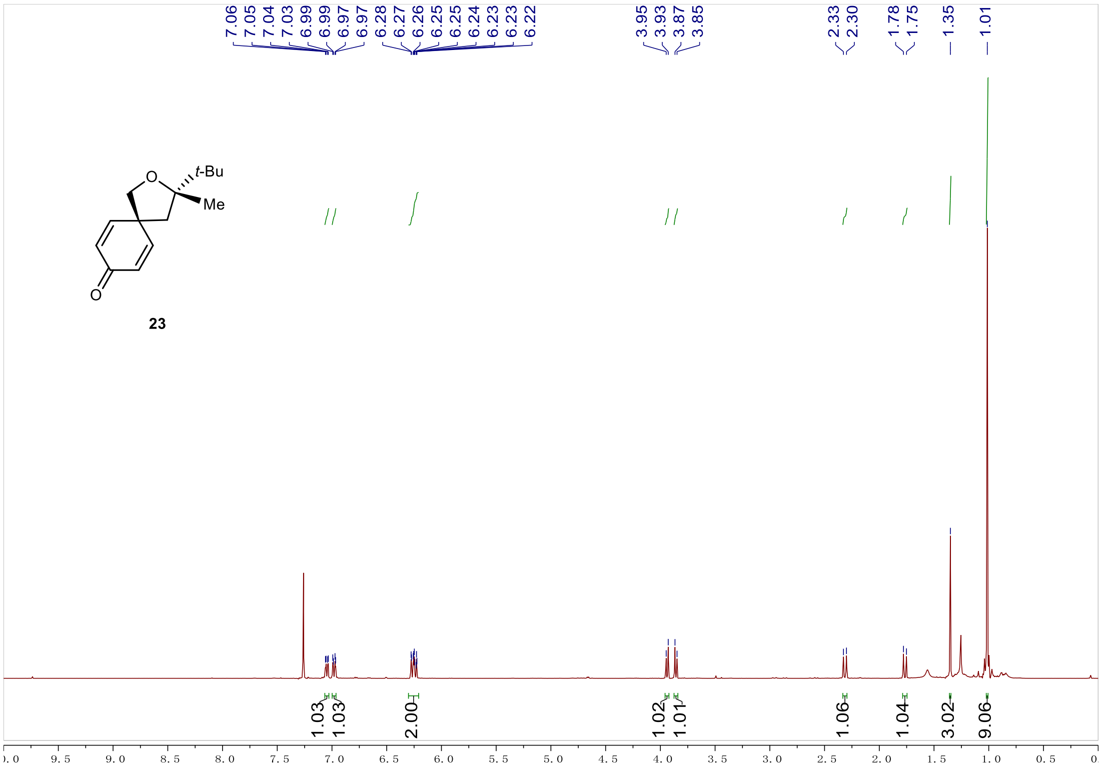

$^{13}$C NMR (126 MHz, $CDCl_3$)

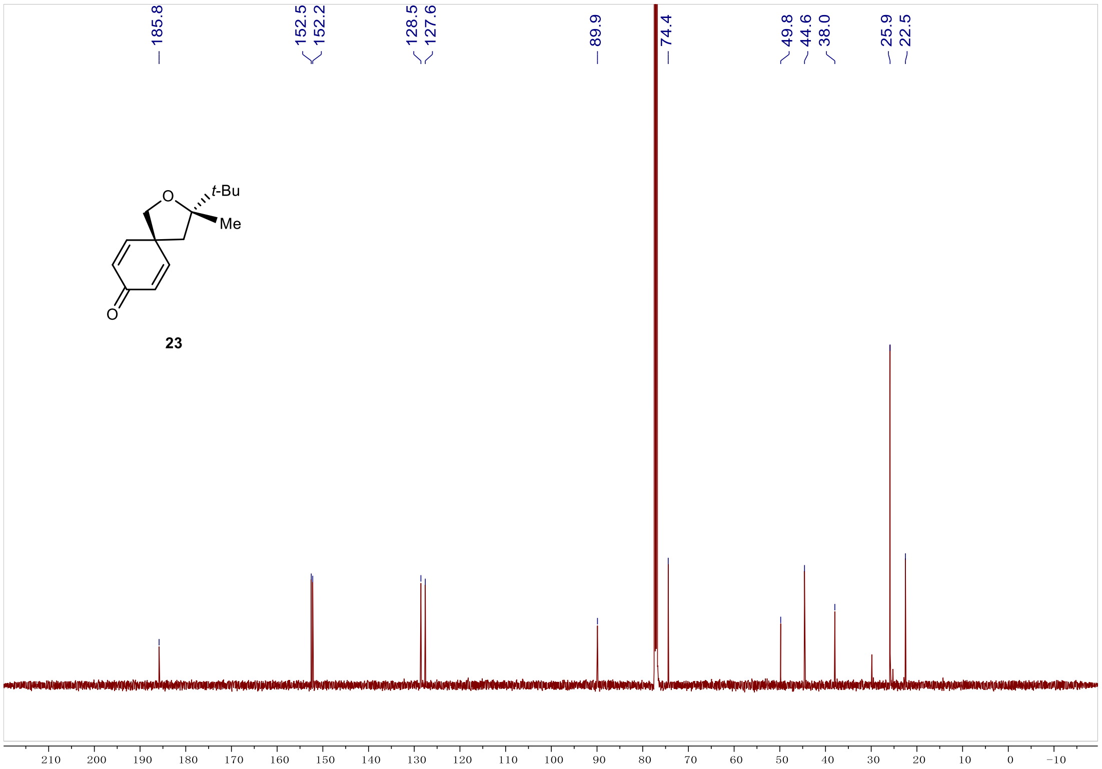

$^1H$ NMR (400 MHz, $CDCl_3$)

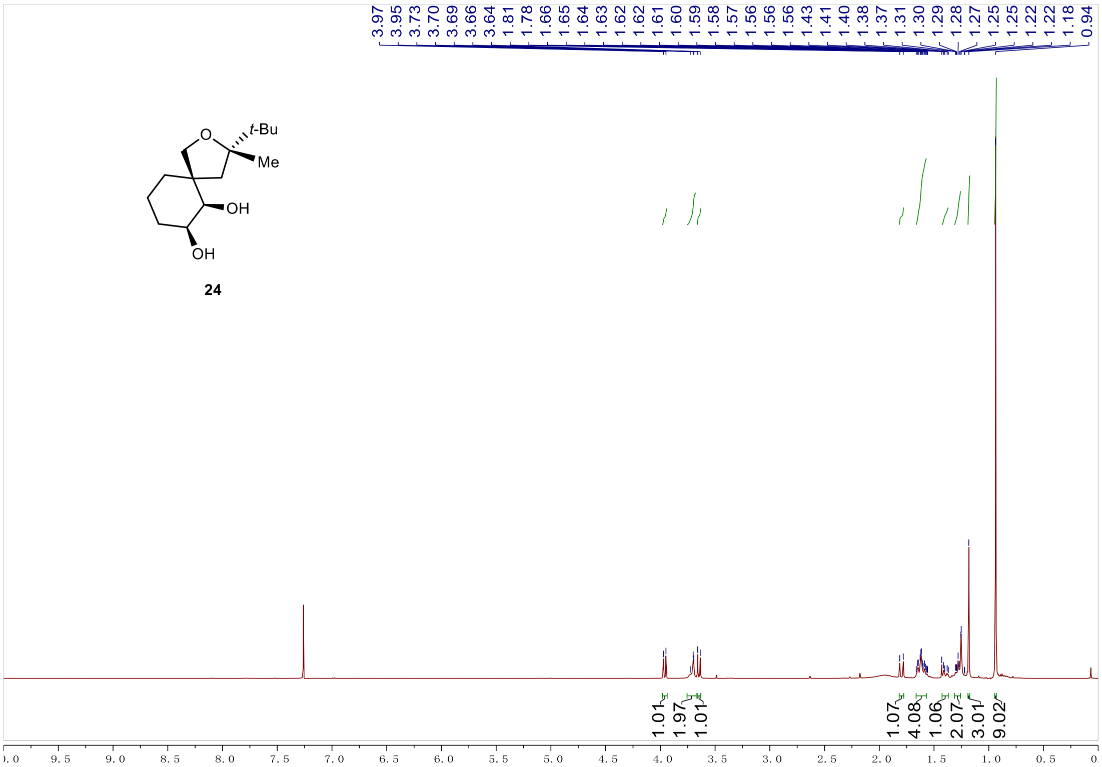

$^{13}C$ NMR (101 MHz, $CDCl_3$)

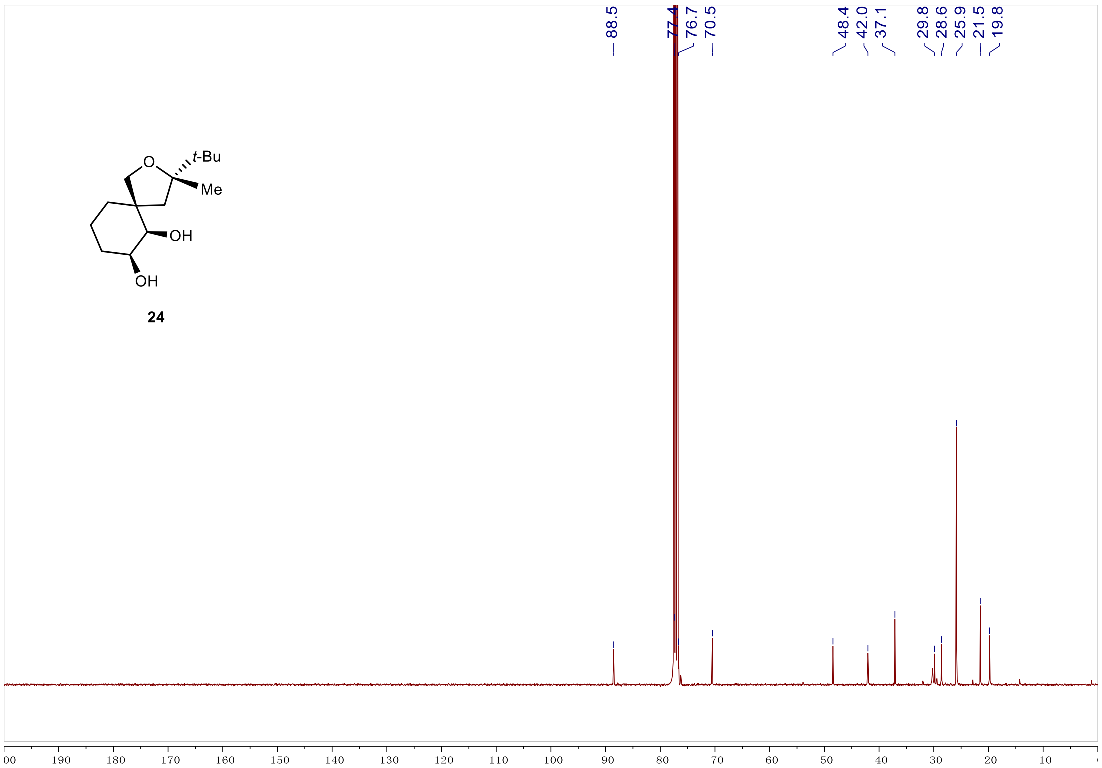

NOESY NMR (400 MHz, $CDCl_3$)

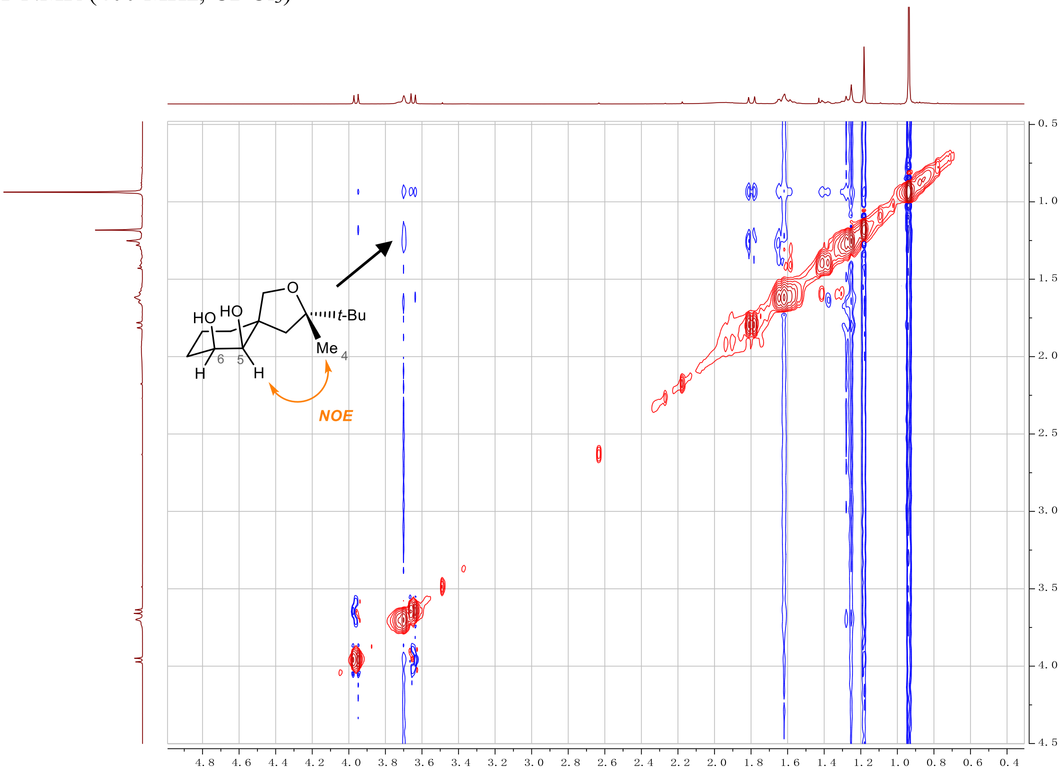

$^{1}H$ NMR (400 MHz, $CDCl_3$)

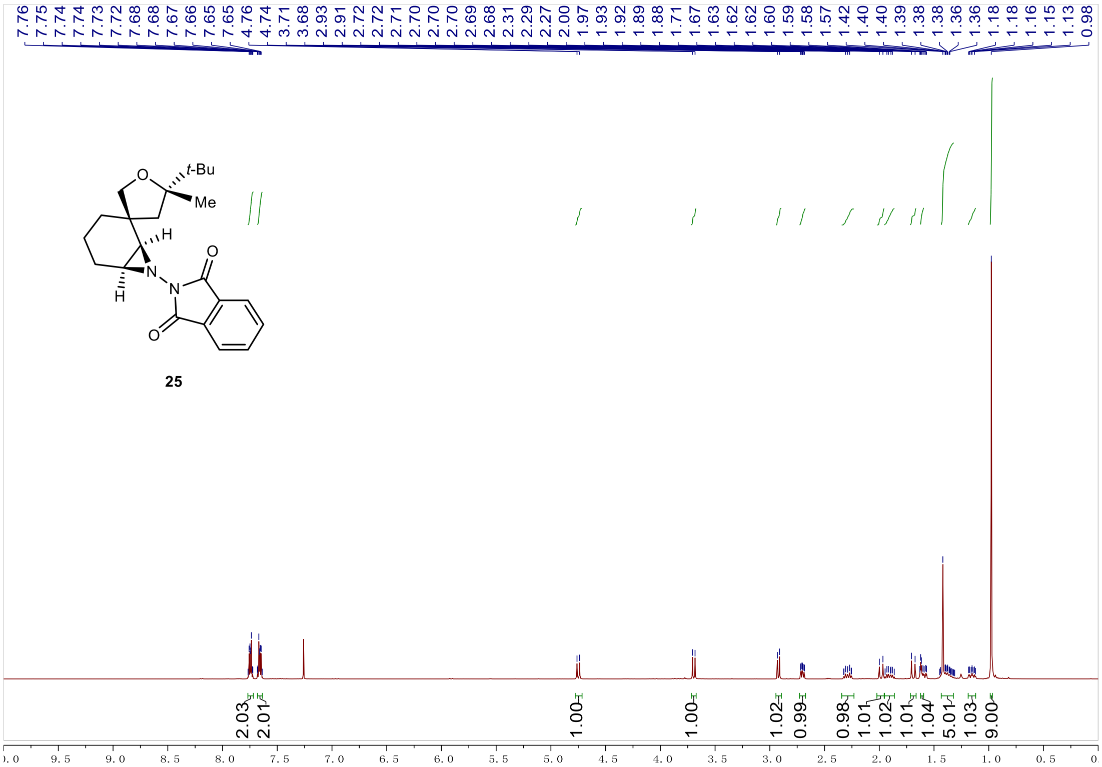


$^{13}C$ NMR (101 MHz, $CDCl_3$)

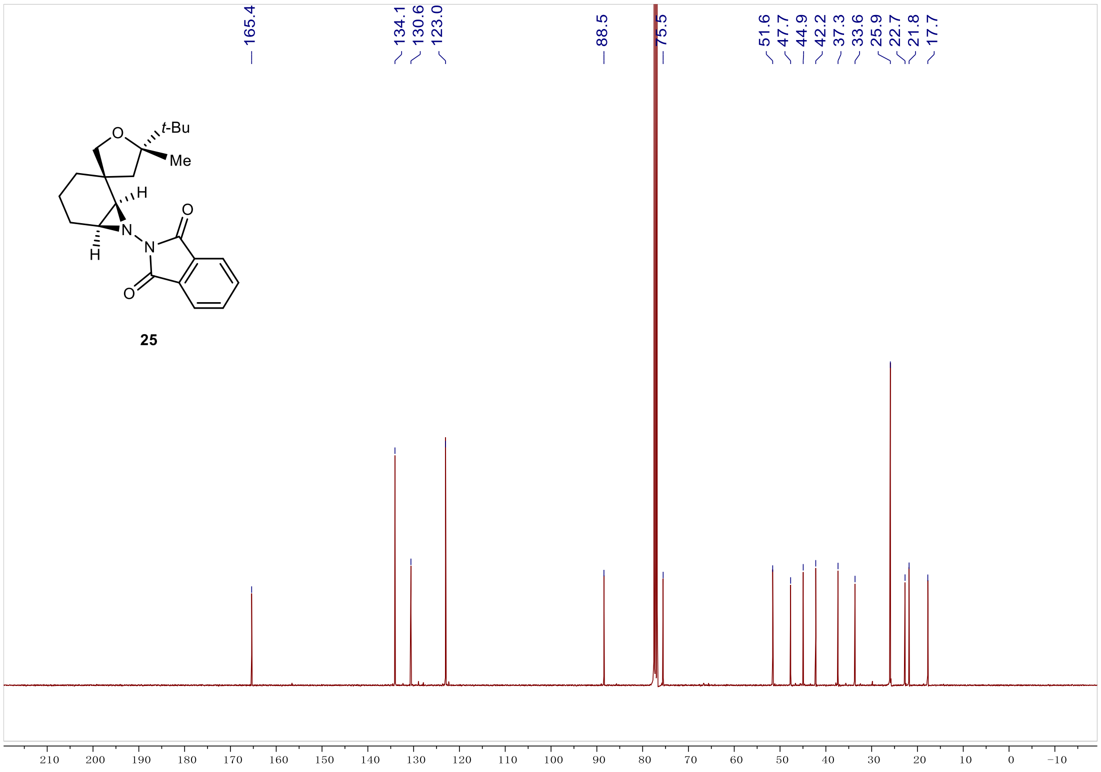

NOESY NMR (400 MHz, $CDCl_3$)

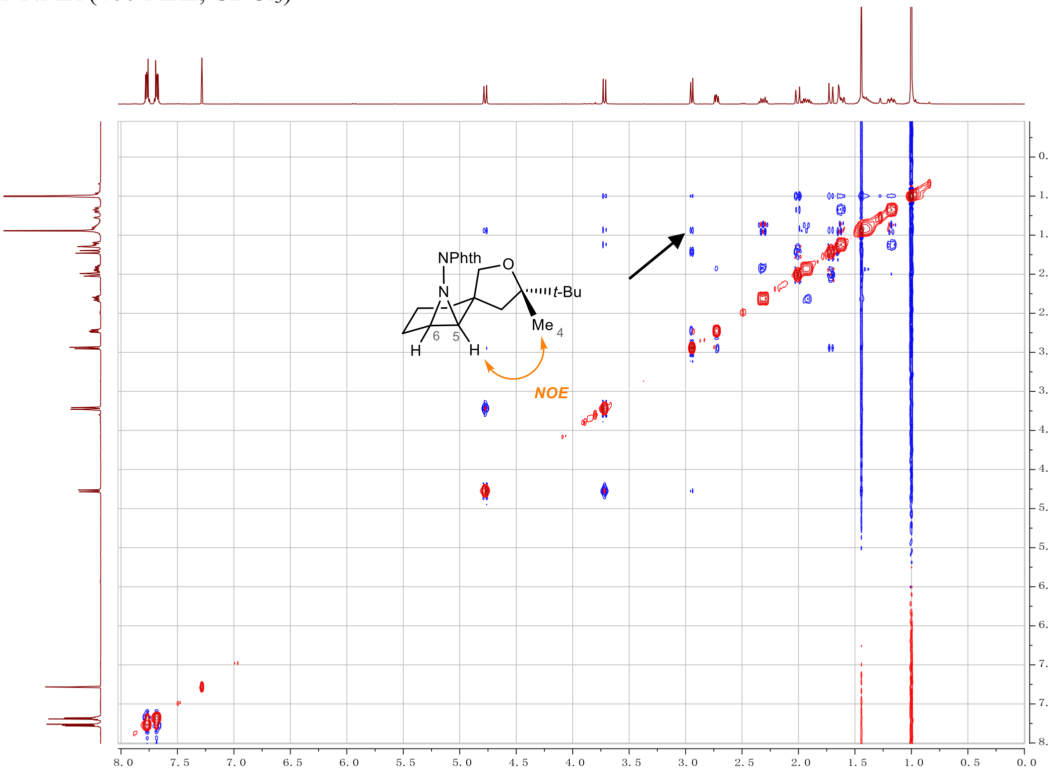

$^{1}$H NMR (400 MHz, $CDCl_3$)

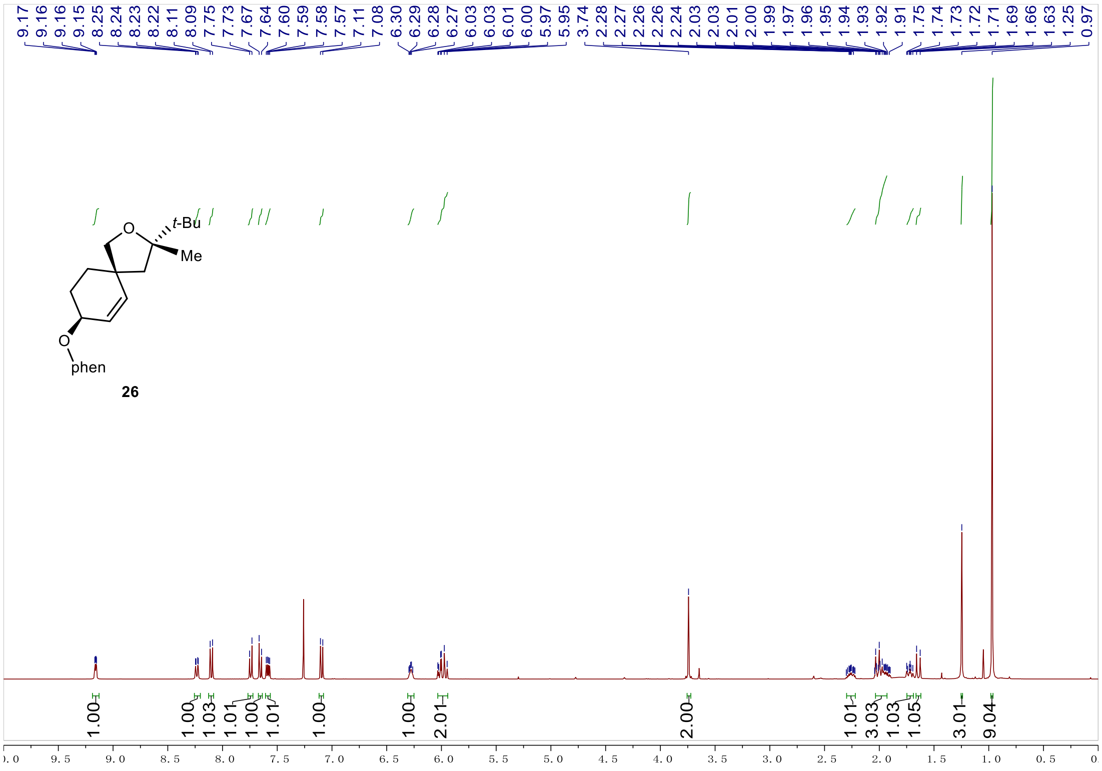


$^{13}$C NMR (101 MHz, $CDCl_3$)

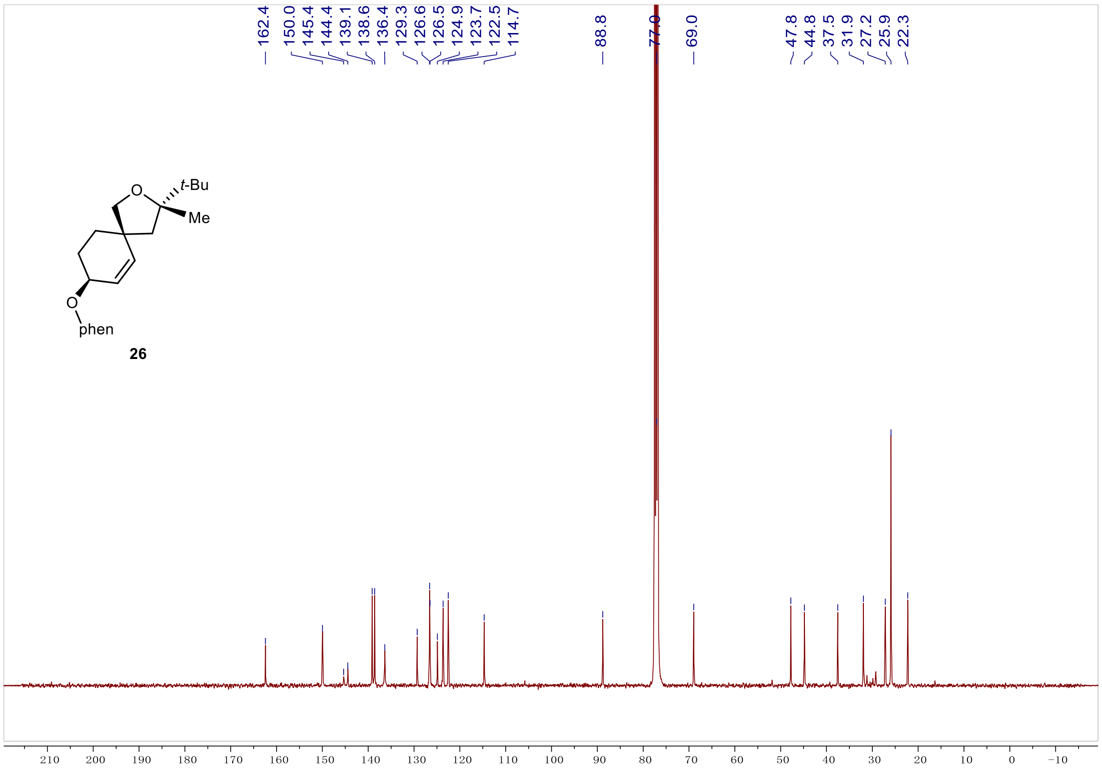

$^1H$ NMR (400 MHz, $CDCl_3$)

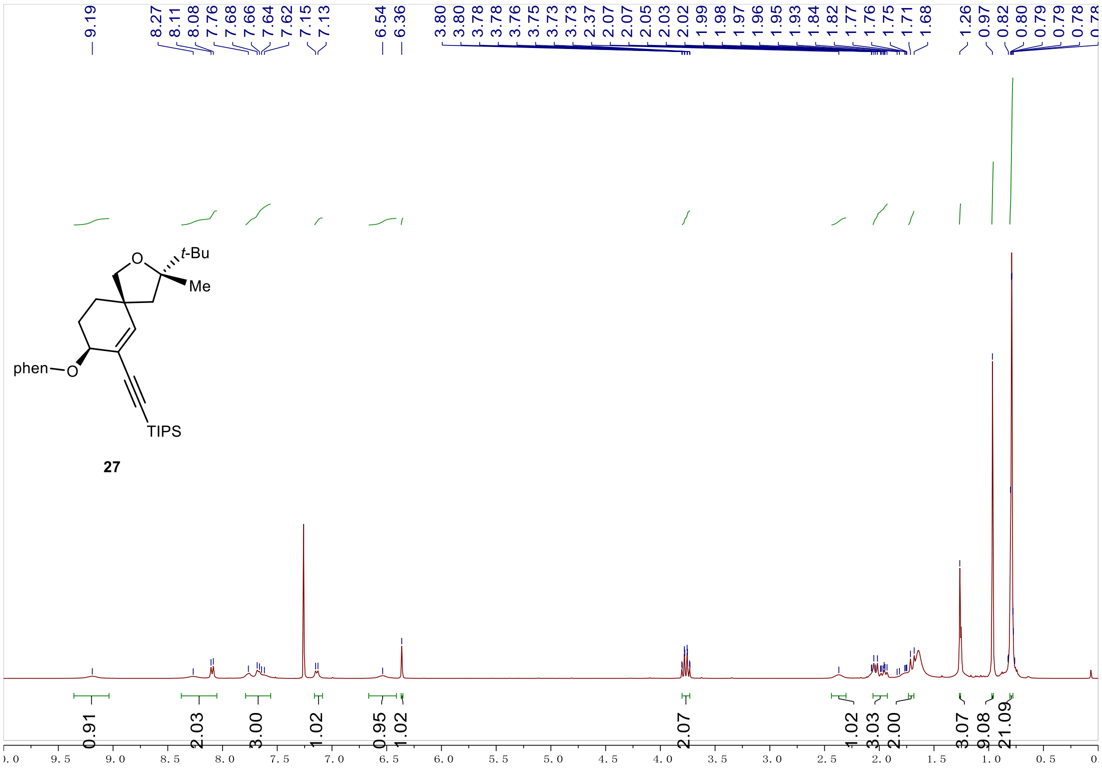

$^{13}C$ NMR (101 MHz, $CDCl_3$)

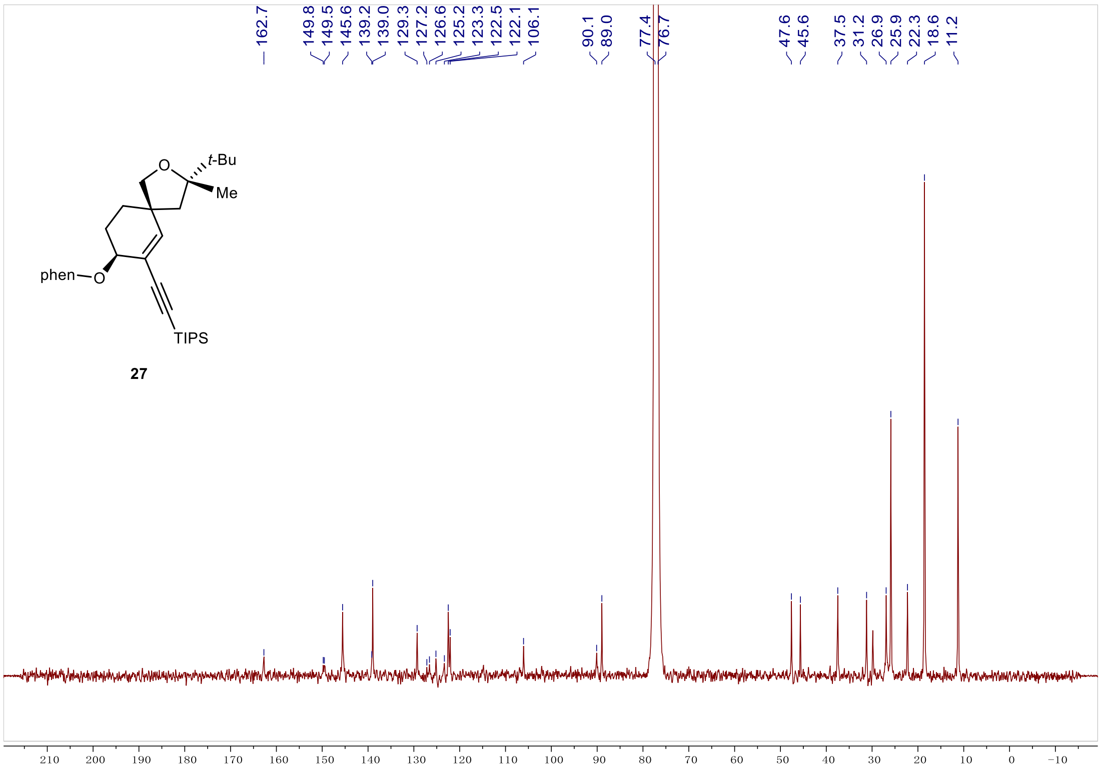

$^{1}H$ NMR (400 MHz, $CDCl_3$)

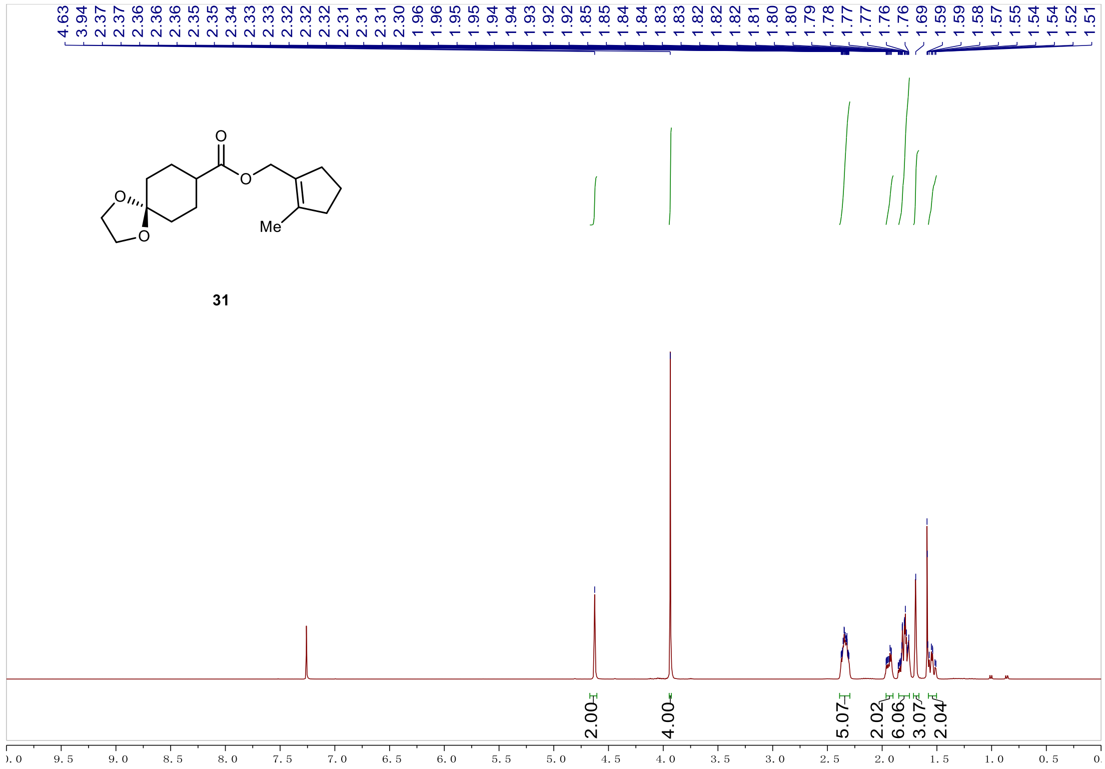

$^{13}C$ NMR (101 MHz, $CDCl_3$)

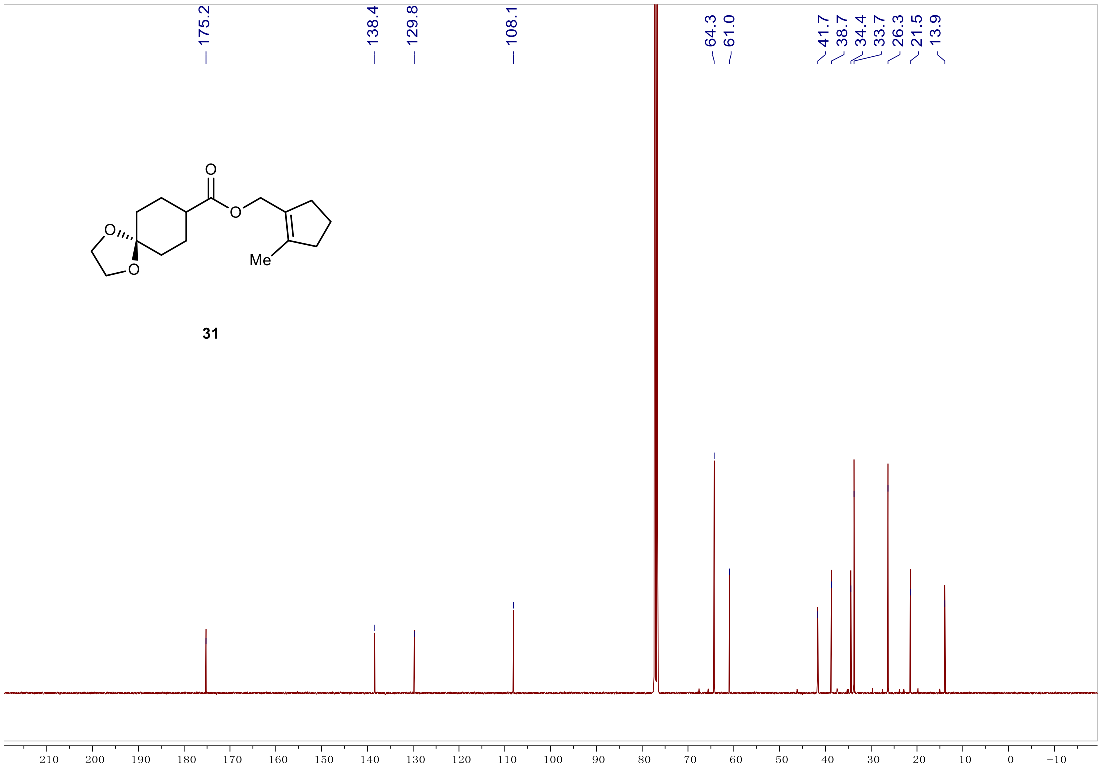

$^{1}H$ NMR (400 MHz, $C_6D_6$)

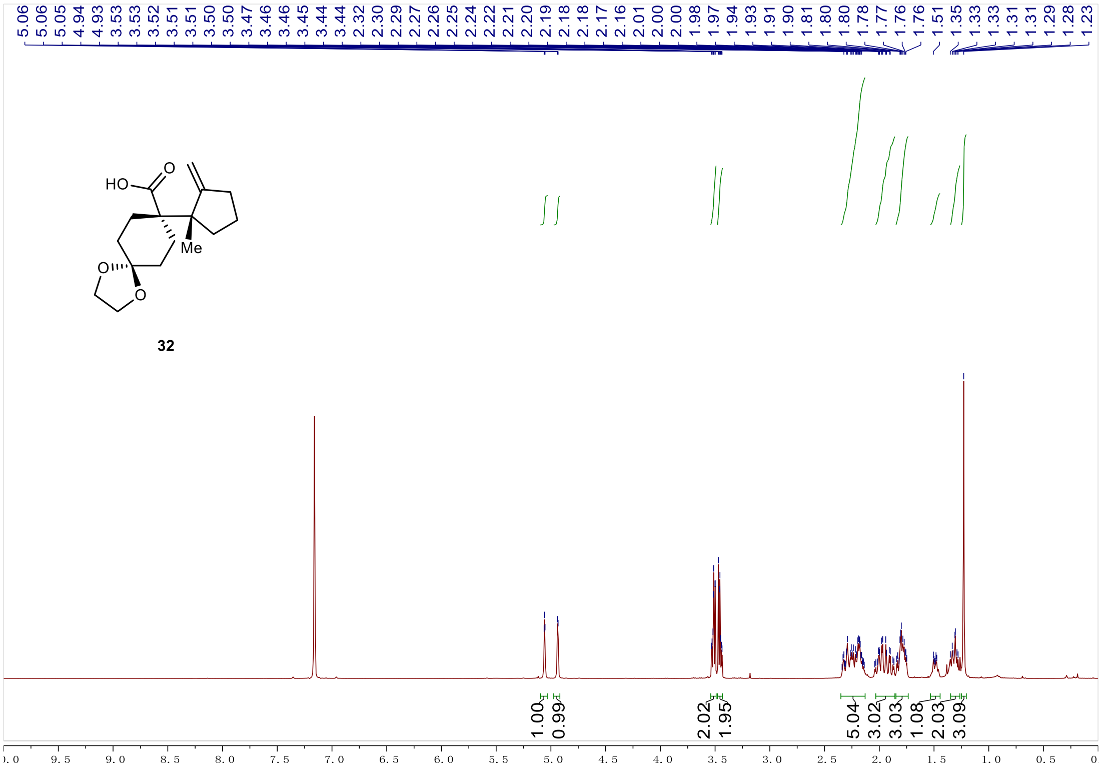

$^{13}C$ NMR (101 MHz, $C_6D_6$)

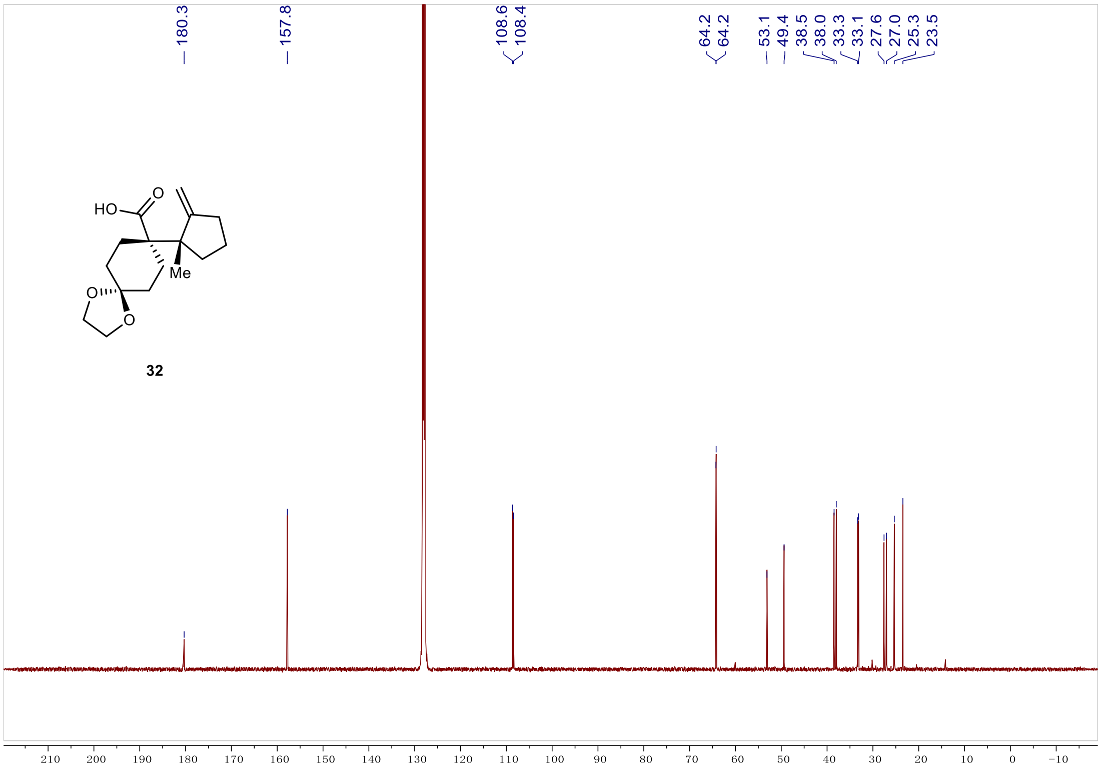

$^{1}H$ NMR (400 MHz, $C_6D_6$)

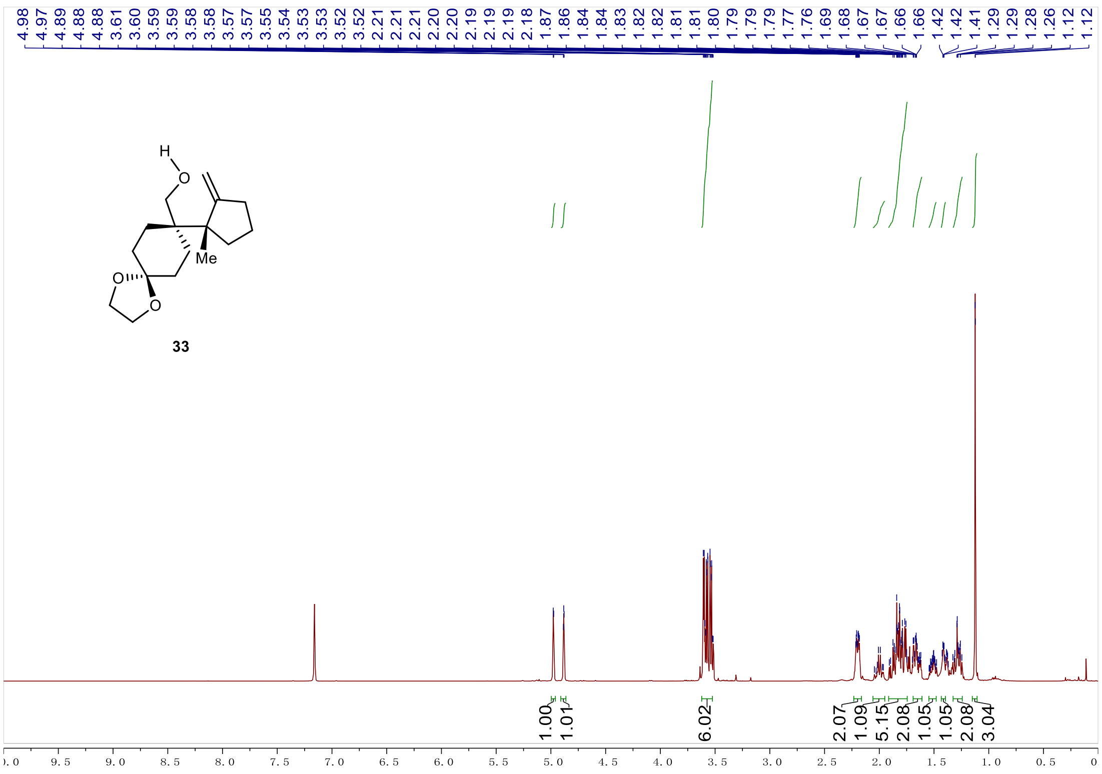

$^{13}C$ NMR (101 MHz, $C_6D_6$)

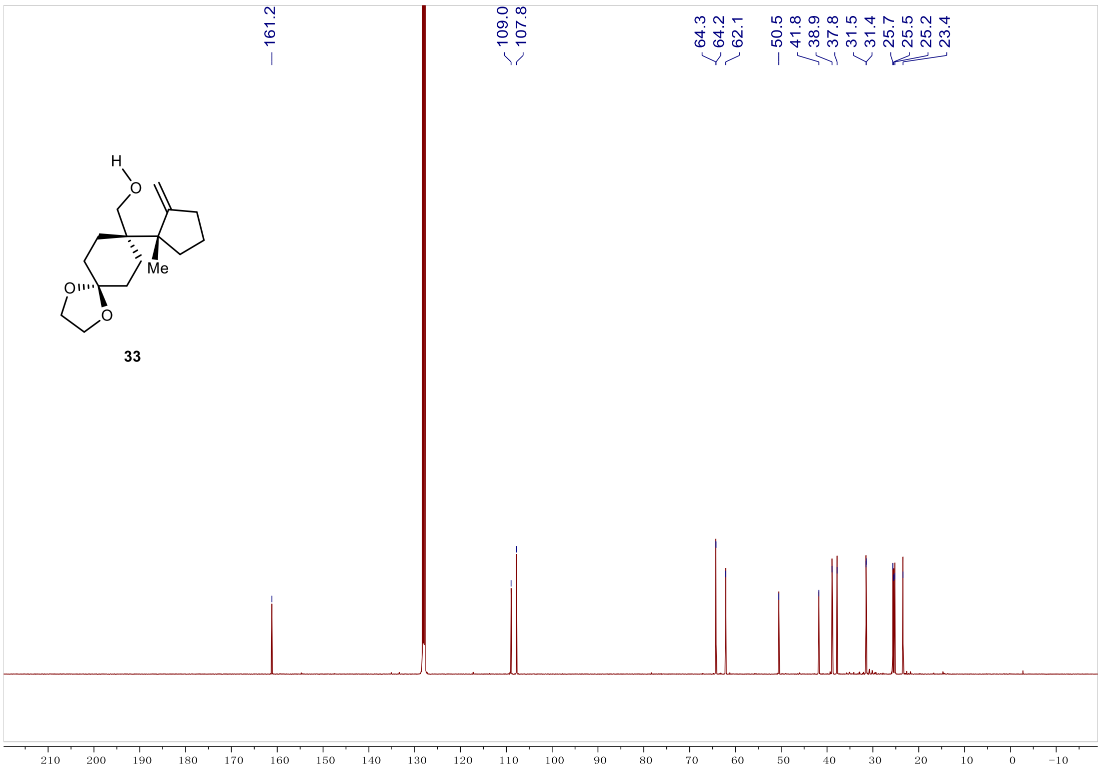

$^{1}H$ NMR (600 MHz, $C_6D_6$)

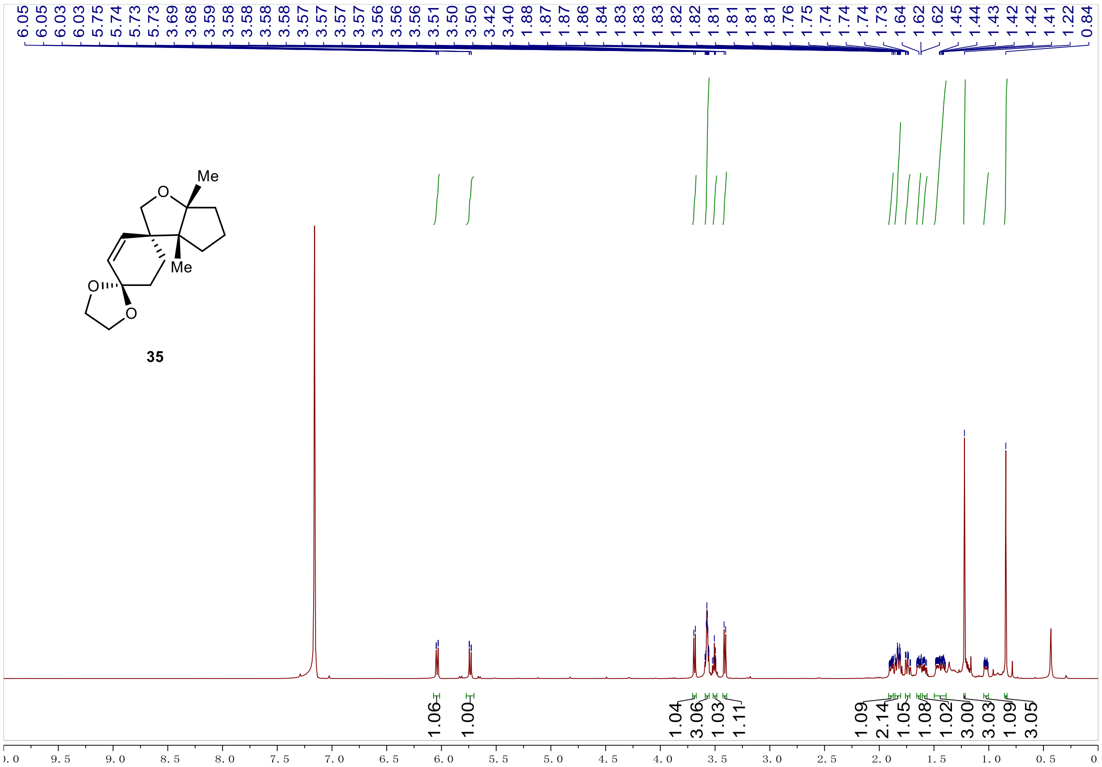


$^{13}C$ NMR (151 MHz, $C_6D_6$)

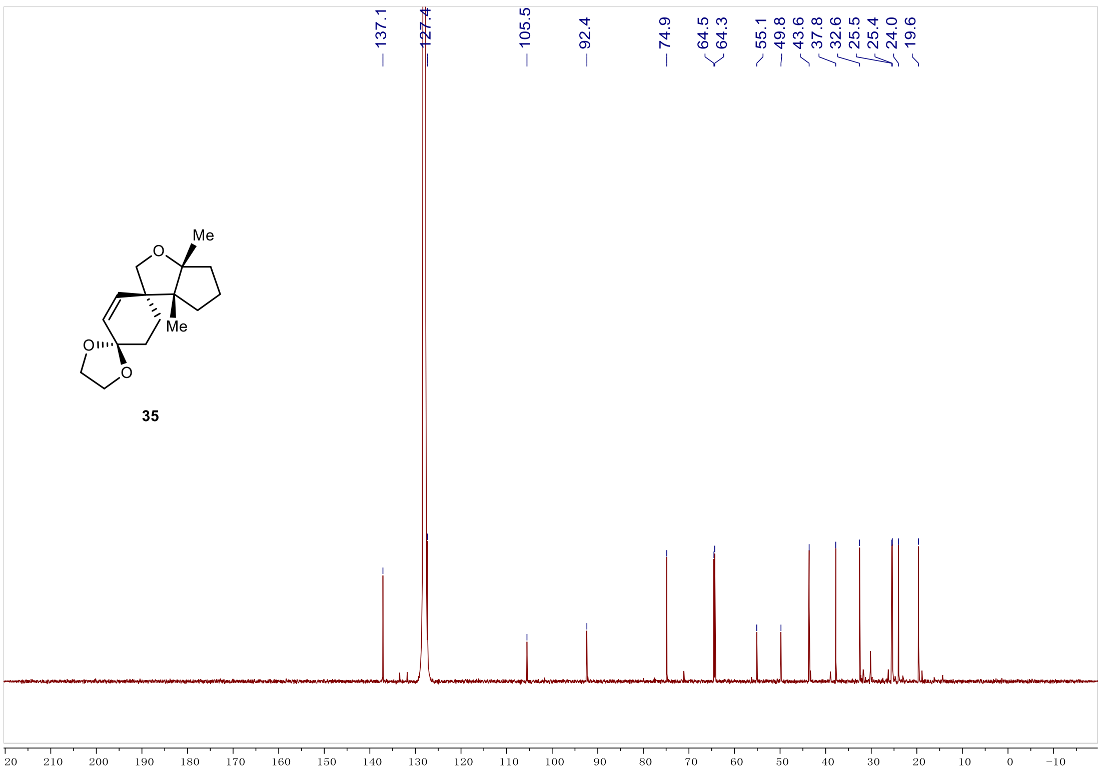

$^1$H NMR (400 MHz, $CDCl_3$)

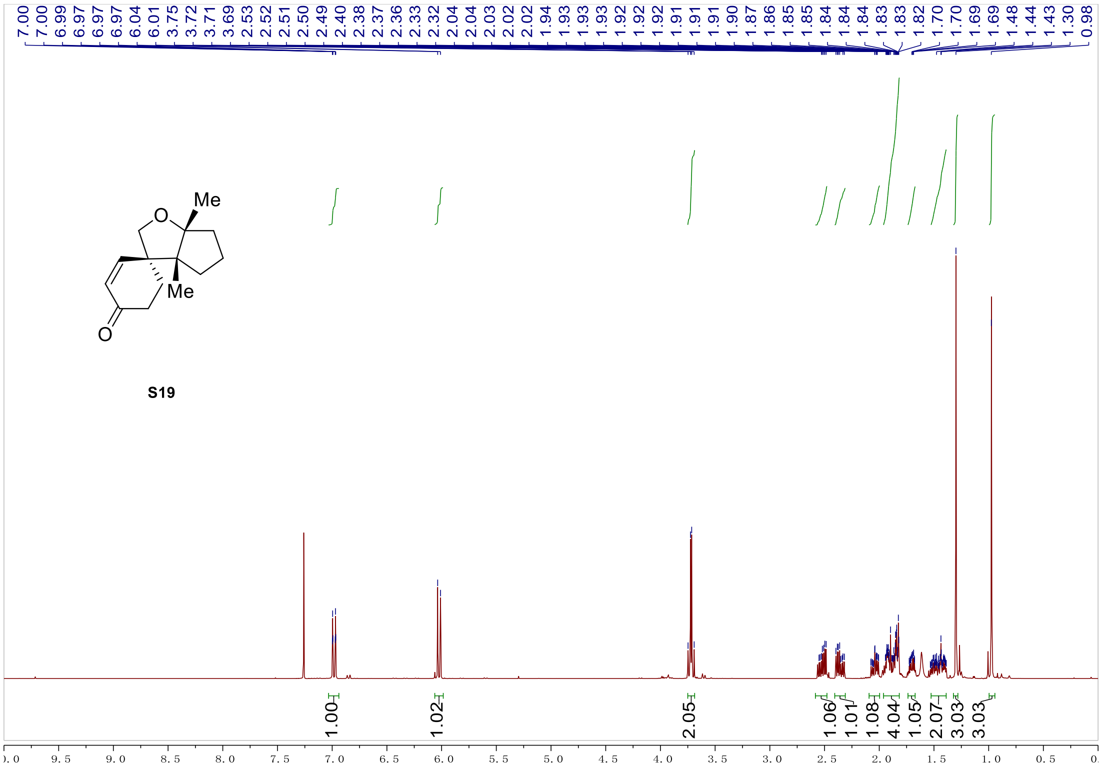


$^{13}$C NMR (101 MHz, $CDCl_3$)

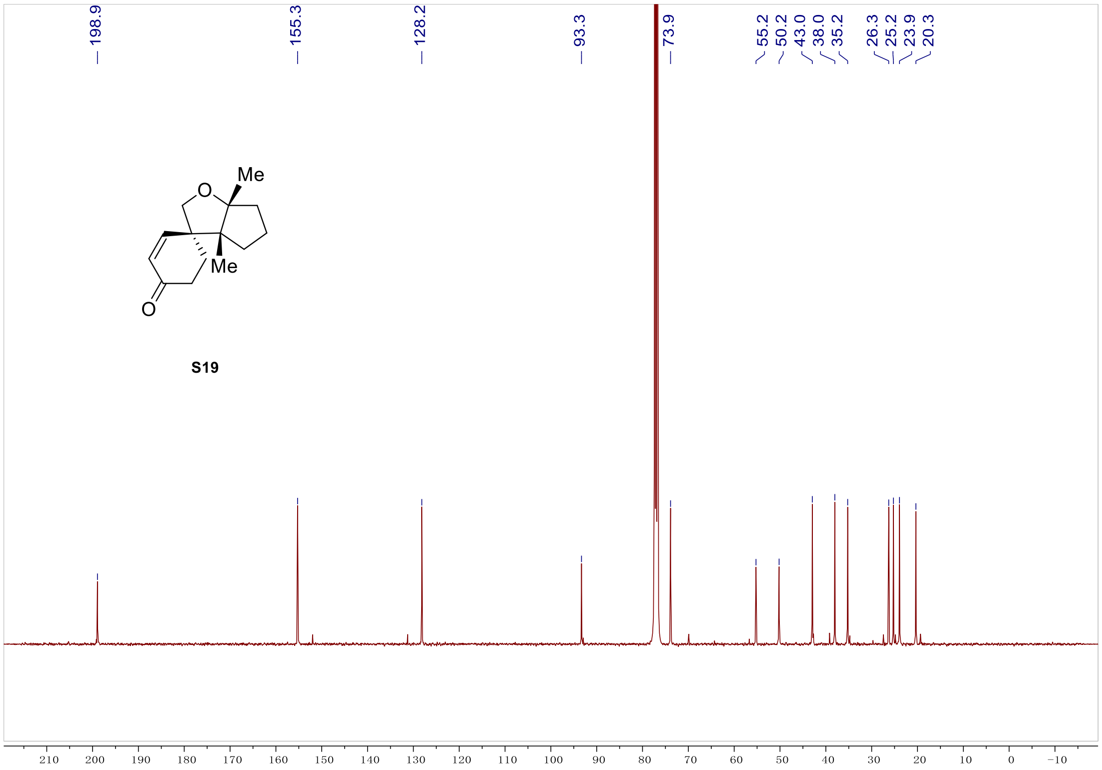

$^{1}H$ NMR (500 MHz, $C_6D_6$)

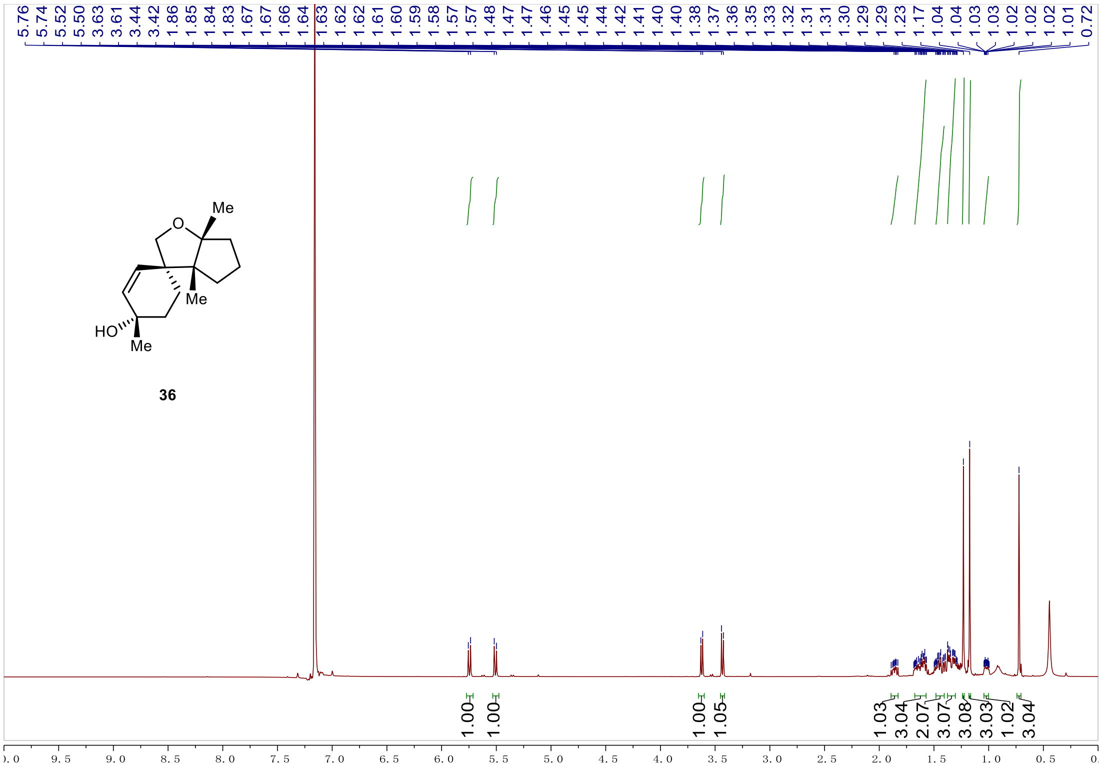


$^{13}C$ NMR (126 MHz, $C_6D_6$)

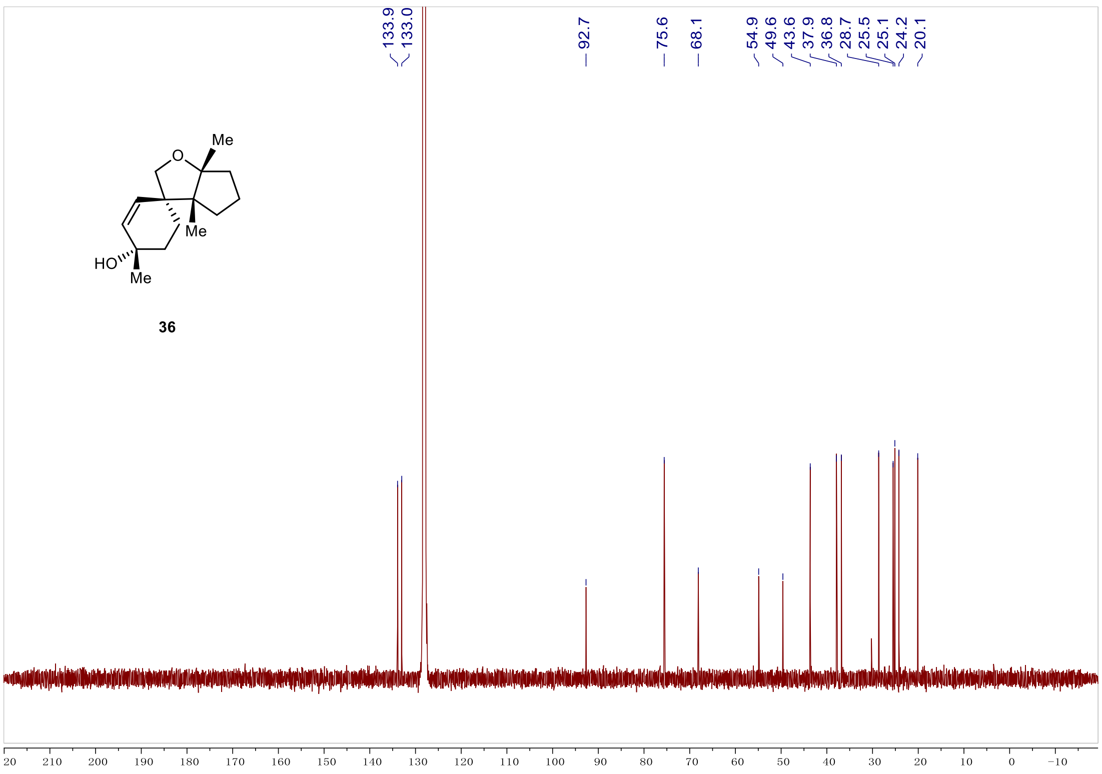

NOESY NMR (600 MHz, $C_6D_6$)

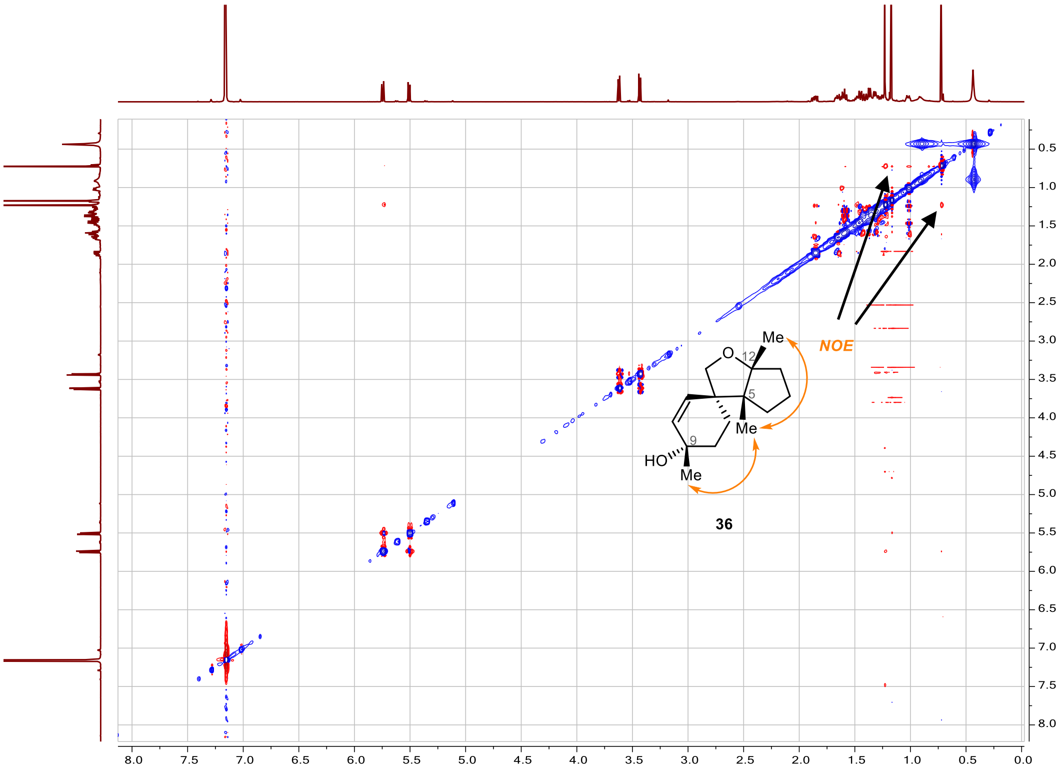

$^{1}$H NMR (400 MHz, $CDCl_3$)

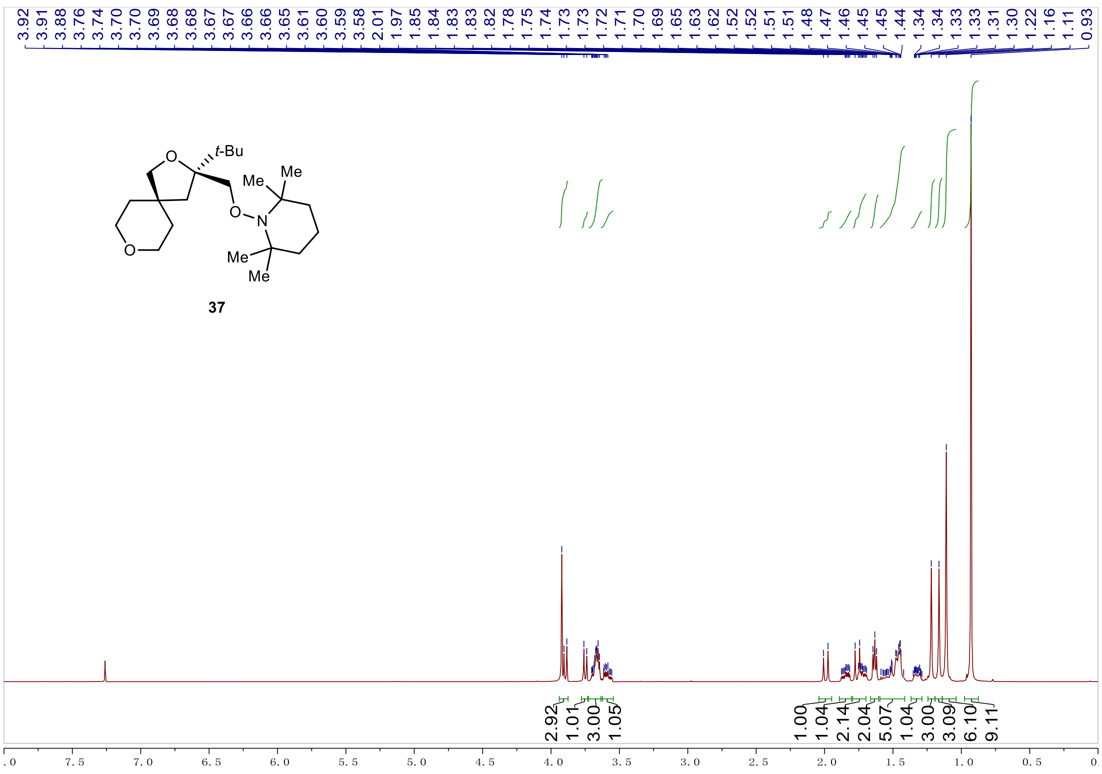


$^{13}$C NMR (101 MHz, $CDCl_3$)

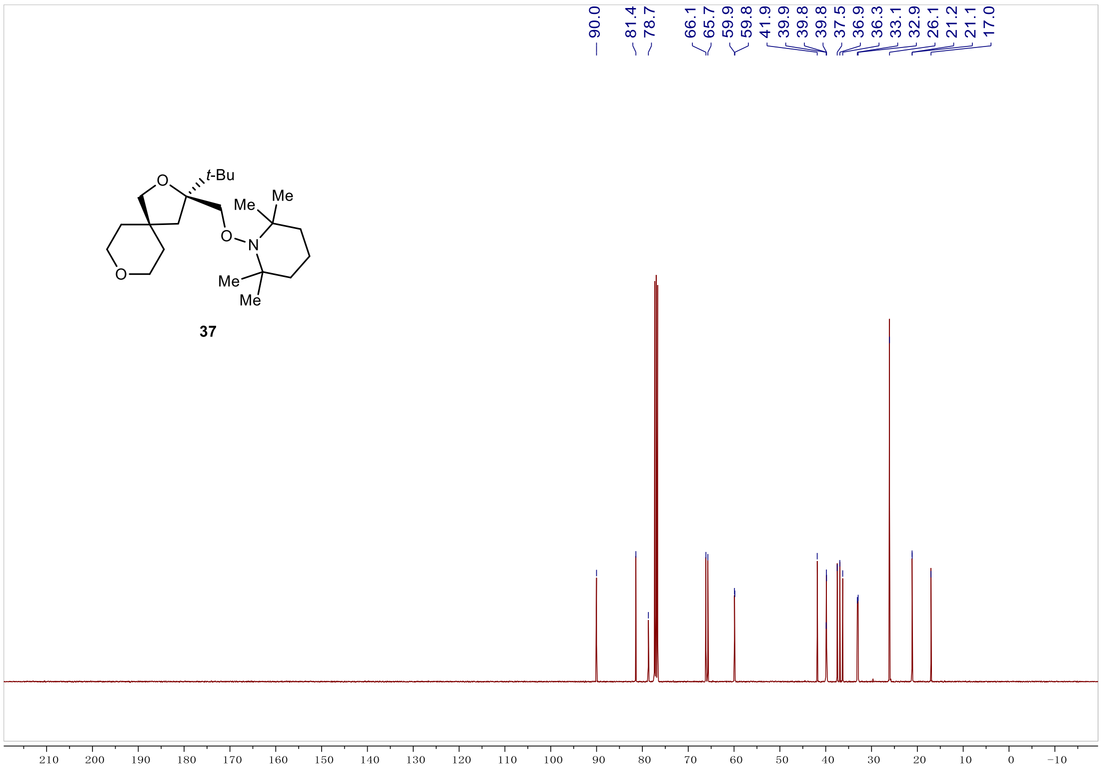

$^{1}H$ NMR (500 MHz, $CDCl_3$)

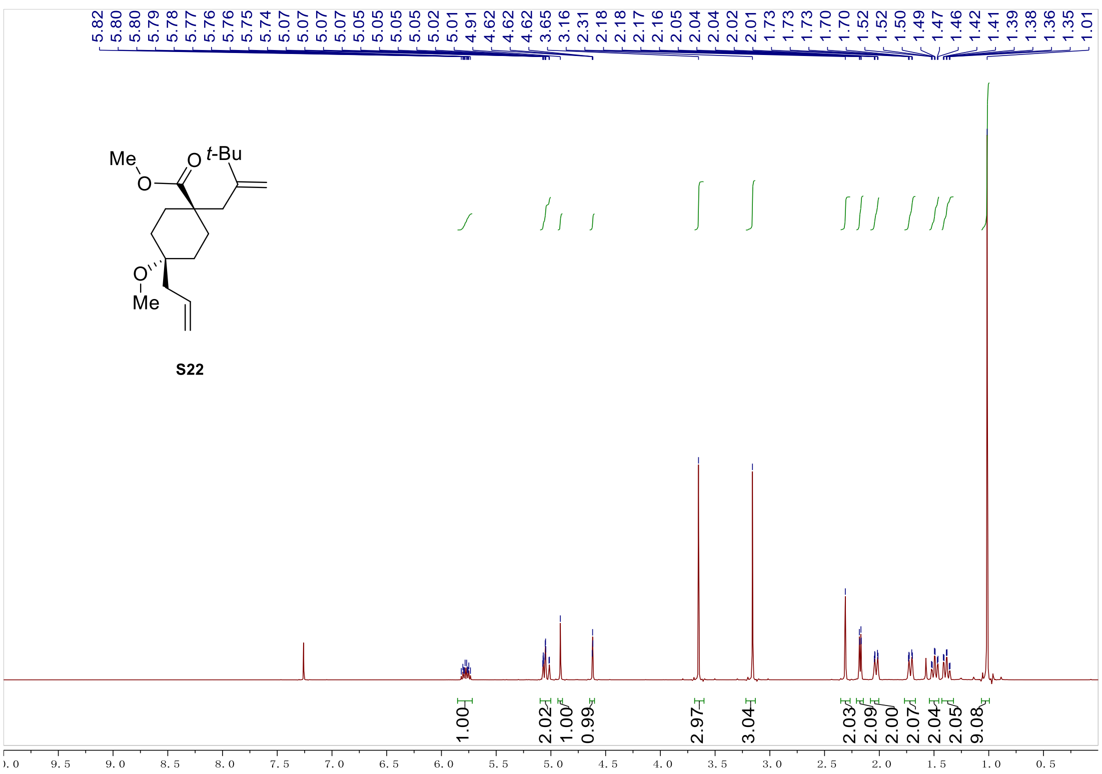

$^{13}C$ NMR (126 MHz, $CDCl_3$)

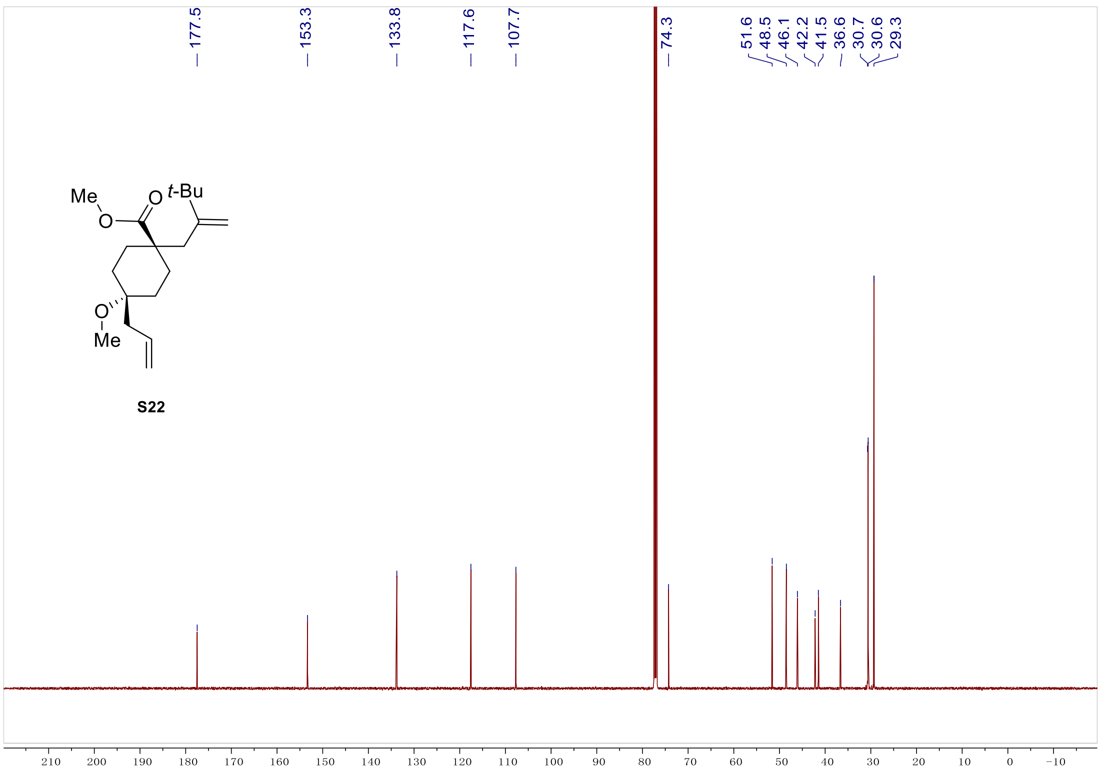

$^{1}H$ NMR (400 MHz, $C_6D_6$)

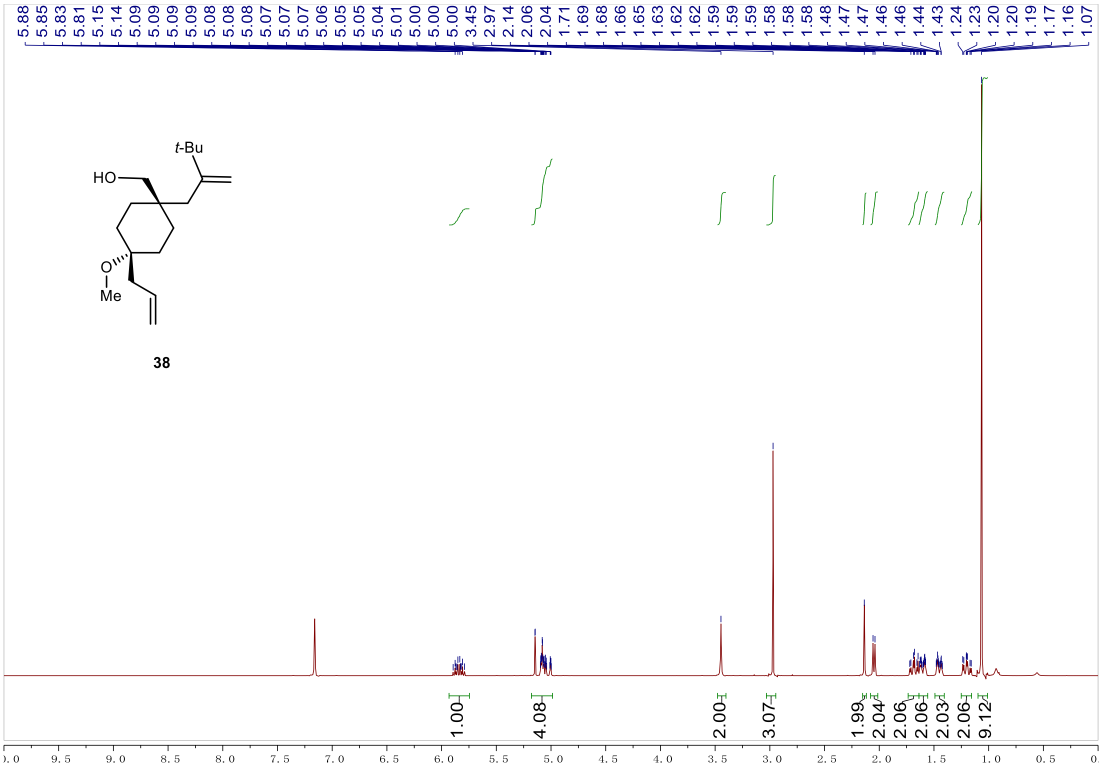

$^{13}C$ NMR (101 MHz, $C_6D_6$)

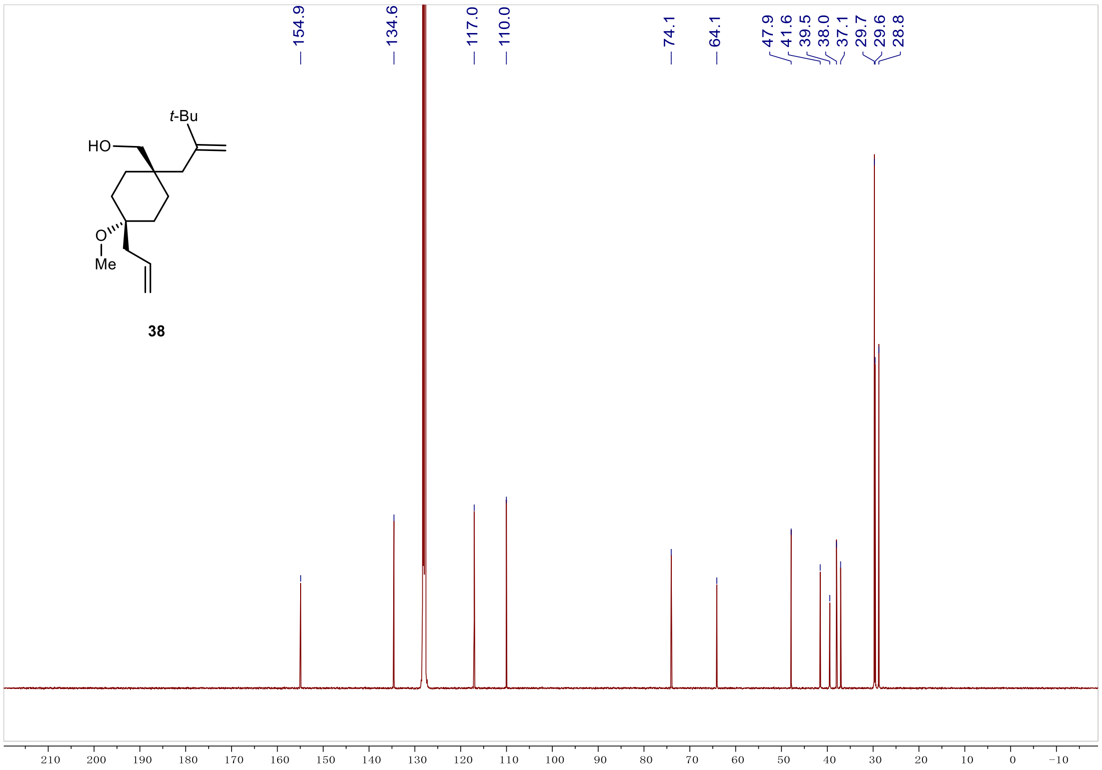

NOESY NMR (400 MHz, $C_6D_6$)

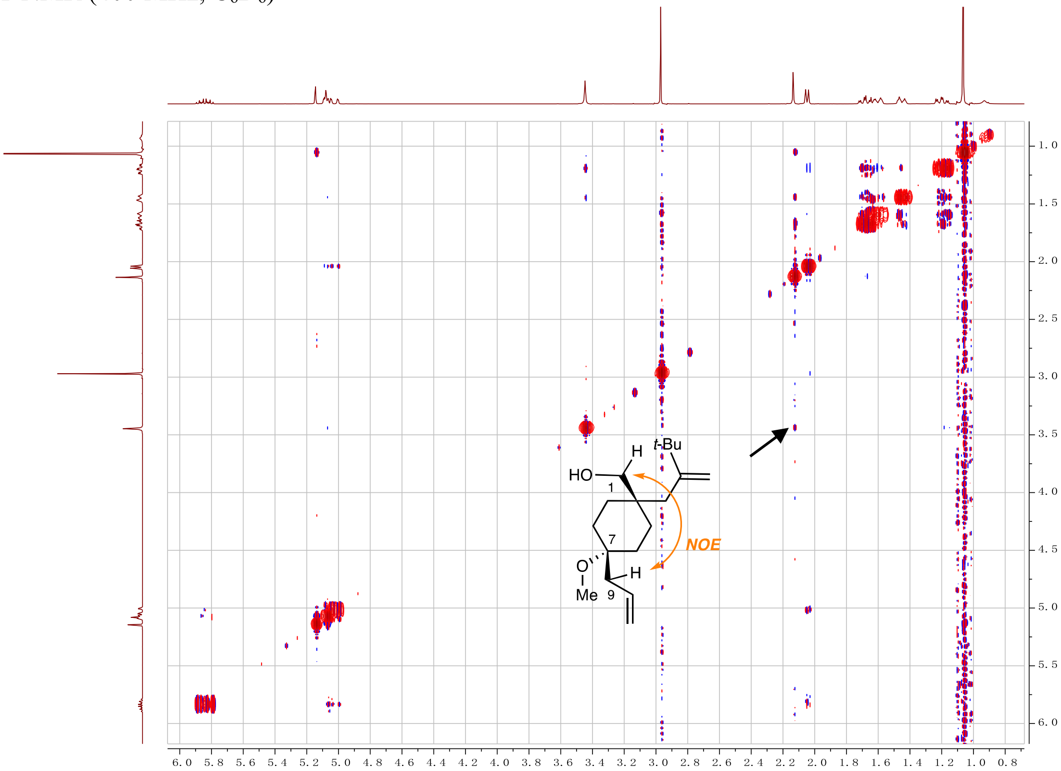

$^{1}H$ NMR (400 MHz, $C_6D_6$)

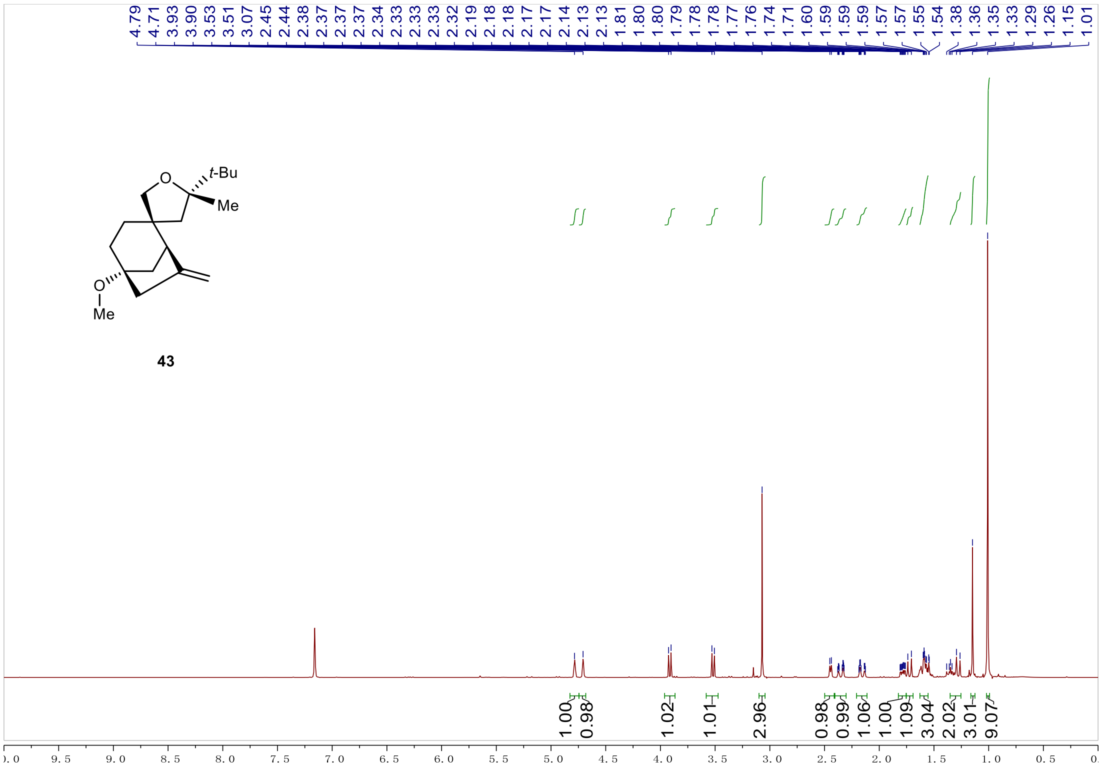

$^{13}C$ NMR (101 MHz, $C_6D_6$)

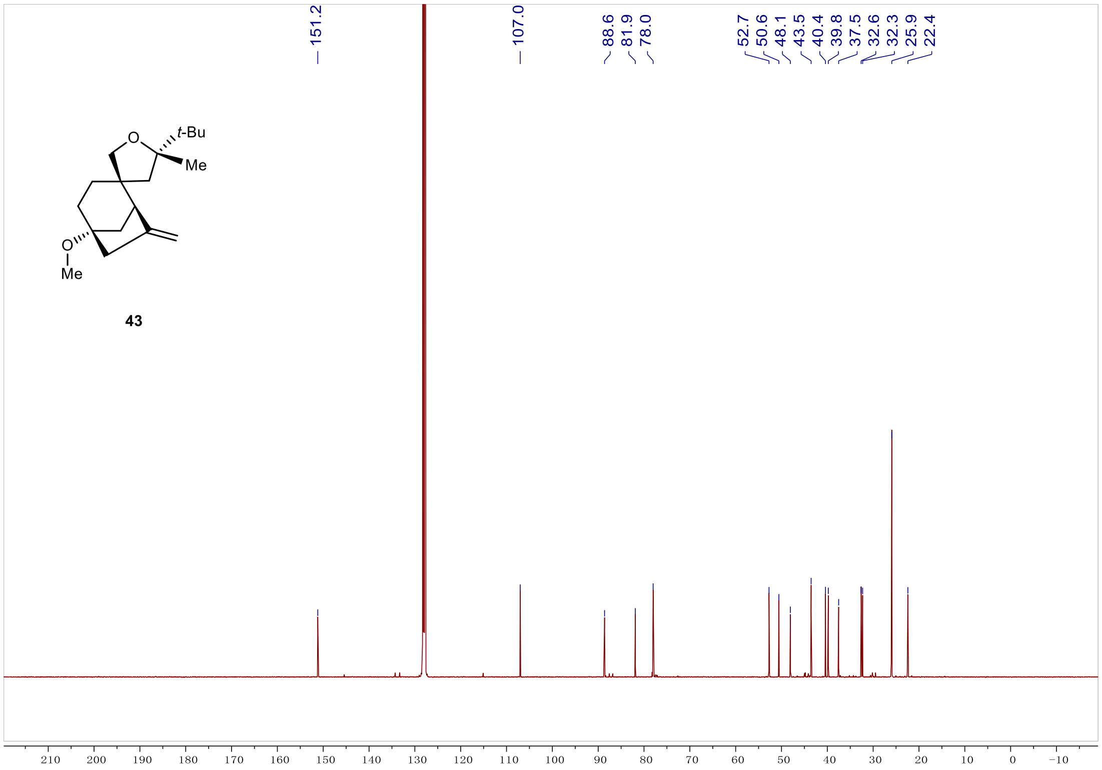

NOESY NMR (400 MHz, $C_6D_6$)

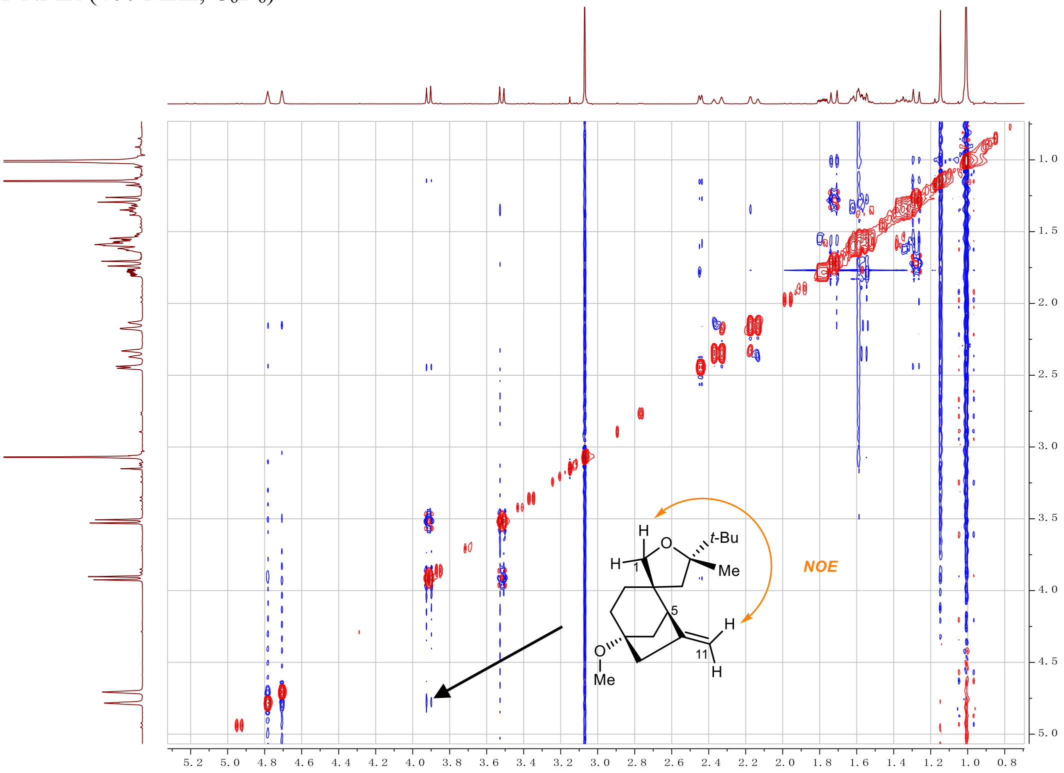

$^{1}$H NMR (500 MHz, $CDCl_3$)

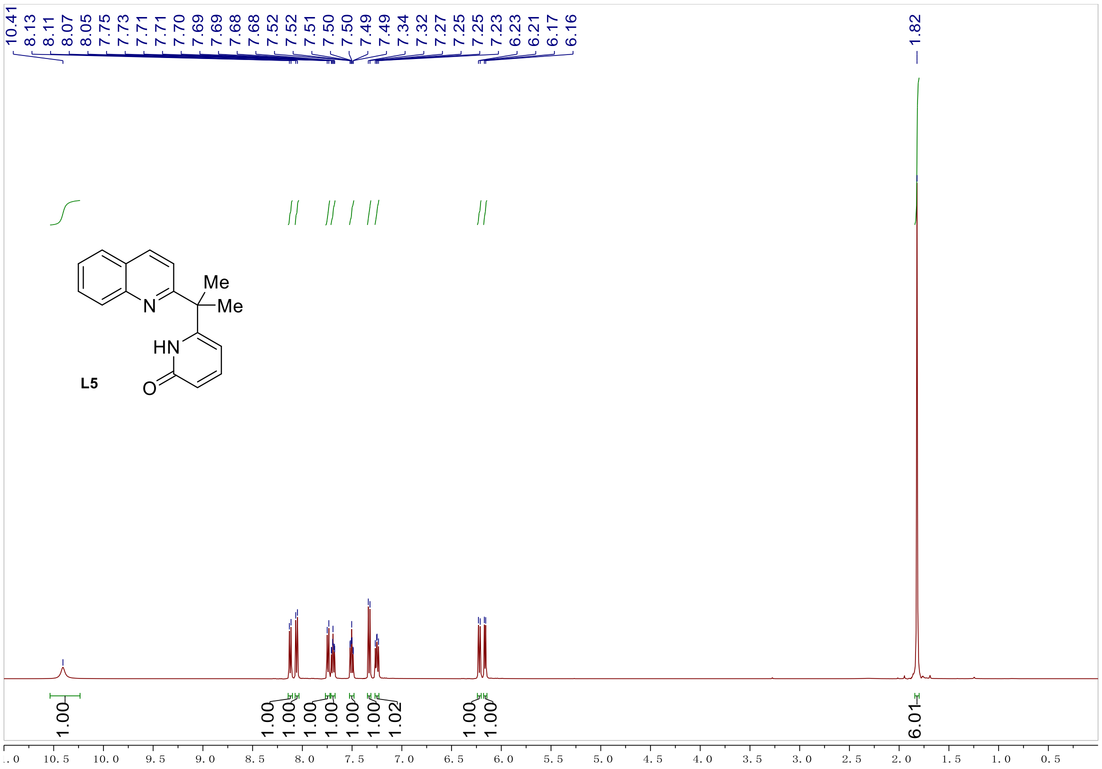


$^{13}$C NMR (126 MHz, $CDCl_3$)

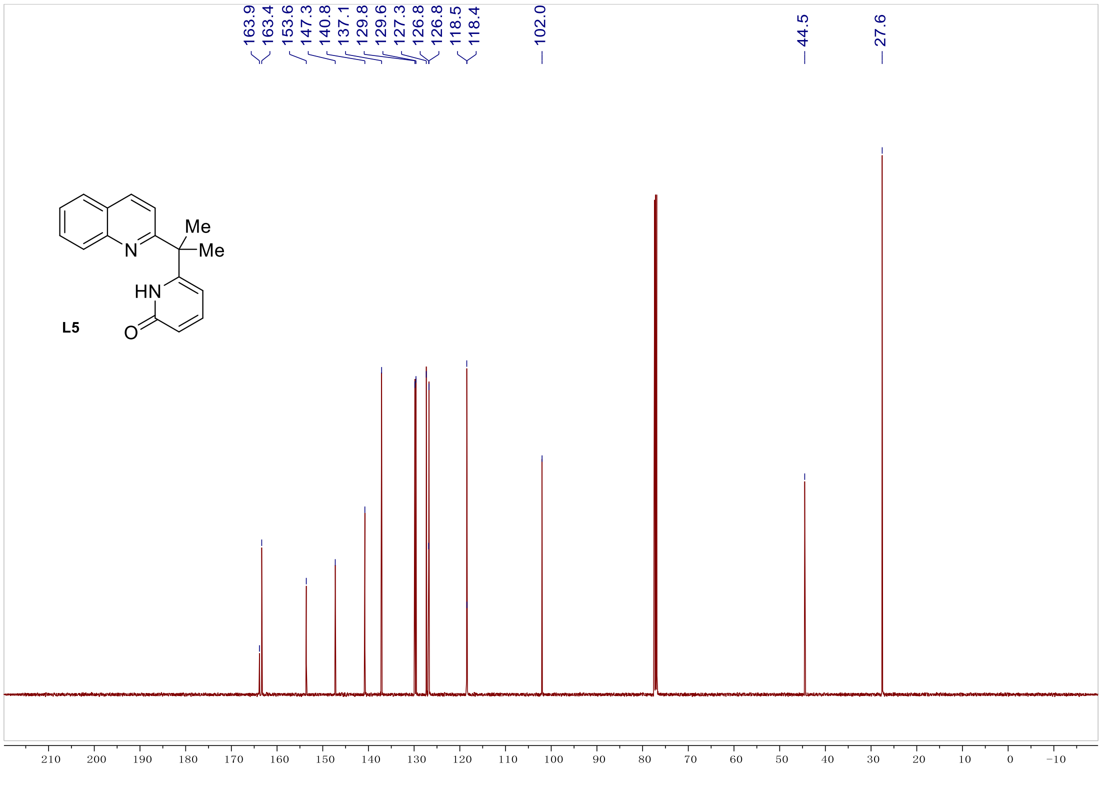

Supplement: Supplementary file 1 — ja4c14418_si_001.pdf [file ja4c14418_si_001.pdf]
